# Synthesis of 4,5-Disubstituted *o*‑Phenylenediamines: An Enabling Platform for Electrochemical Investigations of Interfacial Ion Transfer Reactions

**DOI:** 10.1021/acs.joc.5c00538

**Published:** 2025-12-08

**Authors:** Dennis Tang, Nathaniel Keyes, Jasmin C. Rose, John E. Gonzales, Munho Yang, Marco D. Giles, Yogesh Surendranath, Shane Ardo, Matthew B. Minus

**Affiliations:** †Department of Chemistry, ‡Department of Materials Science and Engineering, §Department of Chemical and Biomolecular Engineering, 8788 University of California Irvine, Irvine, California 92697, United States; ∥ Department of Chemistry, 6717 Prairie View A&M University, 100 University Drive, Prairie View, Texas 77446, United States; ⊥Department of Chemistry, #Department of Chemical Engineering, 2167 Massachusetts Institute of Technology, 77 Massachusetts Ave, 18−163, Cambridge, Massachusetts 02139, United States

## Abstract

*O*-phenylenediamines have emerged as powerful synthons for the installation of molecularly well-defined active sites conjugated to graphitic carbon electrode surfaces. These graphite-conjugated actives sites can serve as rich platforms for the electrochemical investigation of interfacial ion transfer reactions at the molecular level. But widespread utilization of this platform is restricted by the limited synthetic access to *o*-phenylenediamines bearing an array of additional functional groups. Herein, we present three distinct and modular synthetic strategies to symmetric and asymmetric 4,5-*o*-phenylenediamines. We demonstrate the utility of 4,5-*o*-dinitrobenzenes as relatively stable precursors to this class of compounds, as well as a modular route to 4,5-*o*-phenylenediamines in as little as 2 steps from commercial starting materials. We then show, using cyclic voltammetry, that graphitic electrodes modified with molecules obtained from our syntheses exhibit expected electrochemical responses.

## Graphical abstract

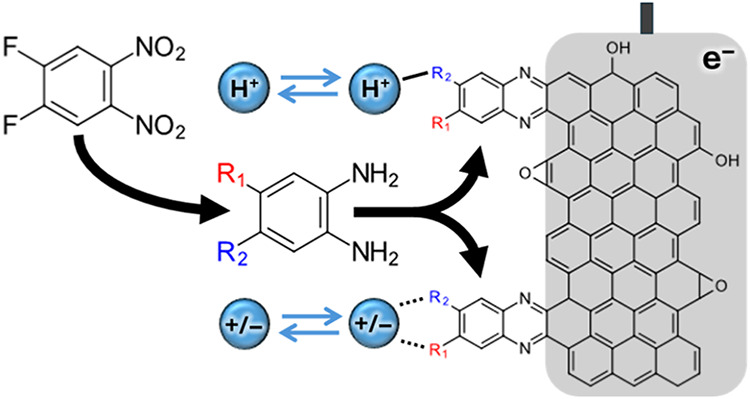

## Introduction

Interfacial ion transfer (IIT) reactions at electrode surfaces play a critical role in electrochemical catalysis, thermochemical catalysis, corrosion, and energy storage.^1–6^ During an IIT reaction the ion crosses the electric double layer, making or breaking bonds with specific surface active sites. This differs significantly from classical electrochemical outer-sphere electron transfer reactions where the electrode simply serves as an inert source or sink of electrons. Given the inherent heterogeneity of most surfaces, the local chemical structure of active sites engaging in IIT is typically unknown, impeding a molecular-level understanding of this ubiquitous class of reactions. Surendranath et al. have developed a platform for studying IIT at the molecular level that consists of conjugating molecularly well-defined active sites onto graphitic carbon surfaces. These graphite-conjugated (GC) sites are prepared by condensation of *o*-phenylenediamines with native *o*-quinone defects on carbon surfaces^7^ (Figure 1). This reaction results in the formation of an aromatic pyrazine linkage connecting the functional groups present on the *o*-phenylenediamine precursor with the rest of the carbon surface. Computational and experimental studies have shown that conjugation serves to position these functional groups within the electric double layer, allowing them to experience the full interfacial electrostatic potential drop.^8–11^ The GC platform has been used to examine the influence of interfacial electrostatics on H_2_ evolution,^12^ CO_2_ reduction,^13^ and O_2_ reduction^7,14,15^ catalysis, exposing fundamentally altered mechanisms relative to analogs dissolved in solution.^8^ When the appended functional groups are acid moieties, the GC active sites serve as molecularly well-defined hosts of interfacial proton-coupled electron transfer (I-PCET), an important subclass of IIT reactions. Using the GC acid platform, Surendranath and coworkers showed that a simple benzoic acid moiety (**COOH**) generated a surface proton donor site that enabled electrochemical characterization of the thermodynamics and kinetics for reversible I-PCET with aqueous electrolytes.^2,8^

**Figure 1:**
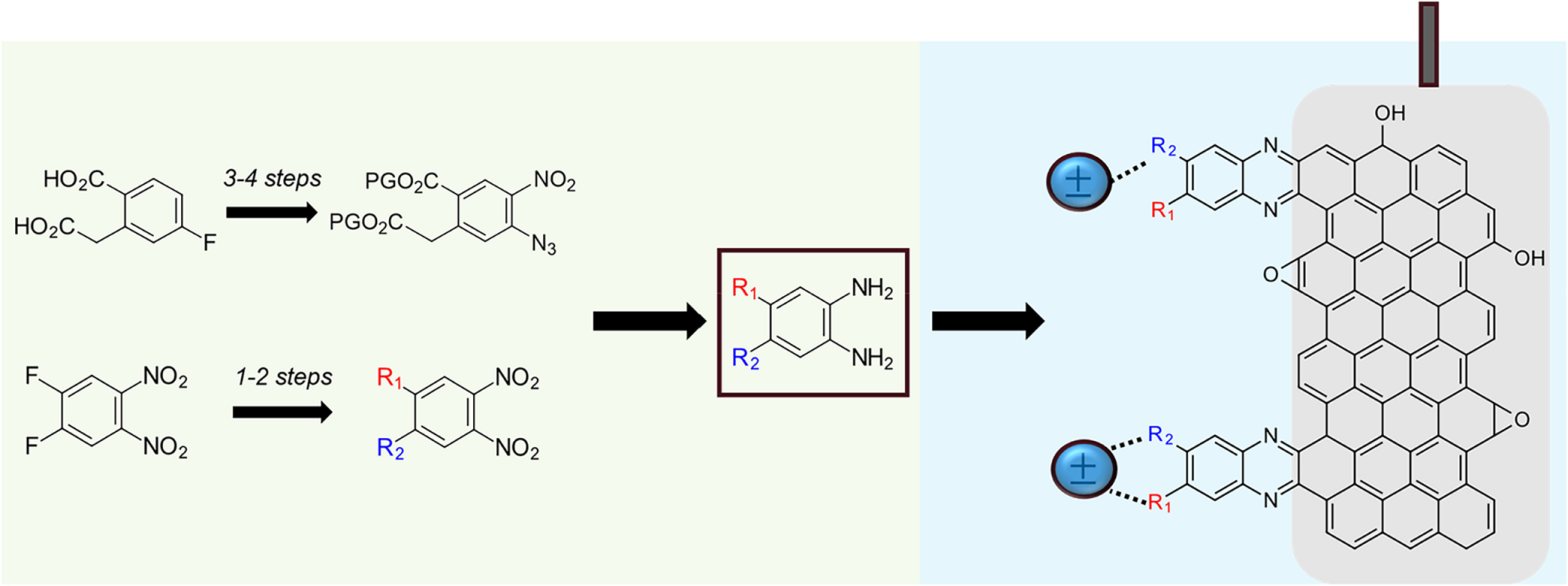
Fundamental electrochemical studies enabled by molecularly modified graphite-conjugated electrodes (blue shaded background on the right)^2,8^ have increased the demand for 4,5-*o*-phenylenediamines that we expand herein to a broader scope of symmetric and asymmetric 4,5-*o*-phenylenediamines.

In principle, the GC platform could be used to examine the factors that promote or inhibit I-PCET across a wide array of moieties. Specific functional groups could be used to construct systems that promote I-PCET via pendant proton relays, akin to strategies employed widely to promote PCET catalysis at molecular transition-metal complexes.^16–18^ Furthermore, the GC platform could serve as an entry point for functionalizing electrodes with coordinating ligands and additional moieties that tune the local environment of surface active sites to assess the effectiveness of specific catalytic reaction pathways.

The foregoing array of investigations is primarily limited by the scarcity of synthetic routes for accessing poly functional *o*-phenylenediamines as precursors to structurally diverse GC sites. This is, in part, due to their oxidative sensitivity. *O*-phenylenediamines are subject to irreversible condensation with carboxylic acids under acidic conditions^19^ and the diamine moiety is subject to irreversible condensation with atmospheric CO_2_,^20^ further complicating synthetic manipulations. While similar syntheses have been reported by Gautrot and Hodge,^21^ their work focused on the use of commercially available, prefunctionalized substrates. In our case, this would have significantly limited the scope of molecules that we intended to access for future studies of IIT using the GC platform. To circumvent these synthetic challenges, herein we report synthetic strategies for accessing a family of *o*-dinitrobenzene derivatives as versatile precursors of *o*-phenylenediamines. These efficient and modular routes only require 2–4 steps from commercial starting molecules to attain desired *o*-phenylenediamines, giving downstream access to GC electrodes primed for studies of I-PCET and other catalytic reactivity.

## Results and Discussion

Our synthetic efforts focused on developing symmetric and asymmetric 4,5-dinitrobenzenes as 4,5-*o*-phenylenediamine precursors. We envisioned that native graphitic carbon surfaces could be modified with symmetric substrates for binding of ionic species in solution, while asymmetric substrates could be used to enhance rates of I-PCET (Figure 1). Higher affinity binding events at the surface of graphitic substrates have the potential to prelocalize cations and anions near the electrode surface, potentially with strong electronic coupling to the graphitic substrate. The preorganization of ions near the electrode surface means that application of a potential bias could drive IIT via polarization of the interface. Asymmetric 4,5-appendages have a different appeal in that the asymmetry could allow one conjugated group to be polarized by the applied potential bias, due to its strong electronic coupling to the graphitic substrate, while the unconjugated functional group would not, due to its weak electronic coupling and therefore dielectric properties. This unique chemical environment of both classes of molecules would allow for distinct testing of surface ion interactions through the examination of thermodynamic and kinetic properties using the tools of electroanalytical chemistry.

### Double EAS Strategy

Our initial synthetic strategies involved using homophthalic acid **1** as a starting material for asymmetric 1,2,4,5 building blocks. It is an inexpensive and readily available compound that already contains the conjugated and unconjugated 4,5 moiety that we were interested in for asymmetric precursors. We first envisioned accessing 4,5-diamino-homophthalic acid **2** through two separate nitration and reduction steps as partially described by Cotelle^22^ et al. (Scheme 1). Nitration of **1** using red-fuming nitric acid afforded the mononitrated acid **3** in reasonable yield. Hydrogenation followed by benzoylation under Schotten-Bauman conditions yielded amide **4**. Unfortunately, installation of the second nitro group proved unsuccessful under various nitrating conditions. We hypothesize that this was due to the deactivated nature of the aromatic ring and the incongruent directing capabilities of the functional groups present in the substrate. Given the difficulties encountered during the second nitration step, we devised a revised synthetic plan that circumvented the hypothesized impediments present in the initial synthesis (Scheme 2). To this end, we first reduced acid **1** to create the para directing groups needed for the 1,2,4,5 substitution pattern. We then protected the resulting diol with acetyl chloride to give the corresponding protected diol **6** in moderate yield. **6** was subjected to various nitration conditions to obtain the dinitro intermediate. While mononitration can take place under gentle conditions (70% nitric acid at room temperature) the dinitration required more aggressive conditions (fuming nitric acid and fuming sulfuric acid at room temperature). While these conditions afforded us dinitro **7**, the efficiency of the reaction varied greatly and was highly dependent on factors such as precise temperature control and rate of substrate addition. Furthermore, the reaction proved not scalable, being more efficient at smaller scales. Interestingly, deprotection of the alcohols occurred concurrently with nitration and so subsequent reactions were instead performed on the unprotected diol S6 with no observable loss in efficiency. With the dinitro intermediate **7** in hand, we chose to oxidize the alcohols to their corresponding acids. Based on a screen of the tolerance of the nitro groups to various oxidation conditions, we hypothesize that the highly electron deficient nature of the substrate likely precluded oxidation under the attempted conditions (Scheme 2).

**Scheme 1:**
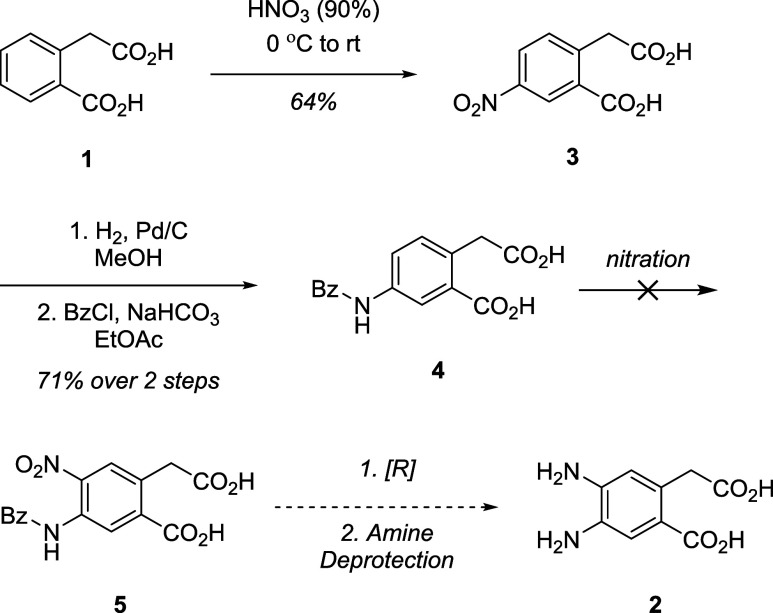
First Synthetic Attempts at Asymmetric Compounds 4,5-*o*-Phenylendiamines

**Scheme 2:**
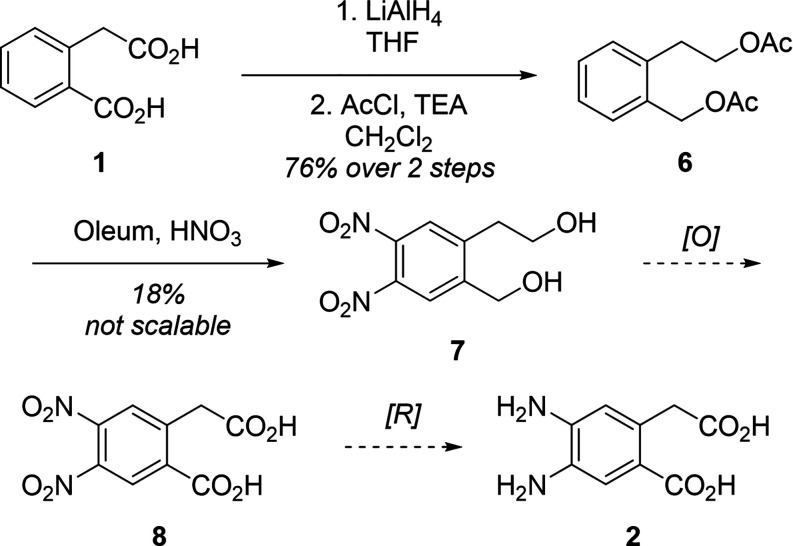
Double Electrophilic Aromatic Substitution Route to 1,2,4,5-*o*-Dinitrobenzenes

### EAS/S_N_Ar Strategy

The withdrawing group effect of the carboxylic acid group in **1** and the ring deactivation that occurs after mononitration forced us to use harsh conditions for the second nitration. These harsh conditions decimated our yields for dinitro **7**, which could be a useful building block for asymmetric 1,2,4,5-phenazines. The poor yields along with the poor scalability of this reaction motivated us to consider alternative modular routes to the 1,2,4,5 asymmetric scaffold. Initial attempts to dihalogenate **1** as part of a Buchwald-Hartwig amination strategy^23^ were also unsuccessful. Literature suggests that the highly electron withdrawn ring system might be more amenable to S_N_Ar reactions than a second harsh electrophilic aromatic substitution (EAS) reaction.^24,25^ Therefore, as a more viable pathway to 1,2,4,5 precursors we decided to bypass the second EAS reaction in favor of an S_N_Ar reaction (Scheme 3). Commercially available 5-fluoro-homophthalic acid **9** was chosen as a suitable starting material to pursue this strategy. Mononitration in a 1:1 v/v solution of concentrated nitric acid and sulfuric acid gratifyingly gave the desired fluoro-nitro aromatic **10** in quantitative yield with no additional purification aside from a standard workup.

**Scheme 3:**
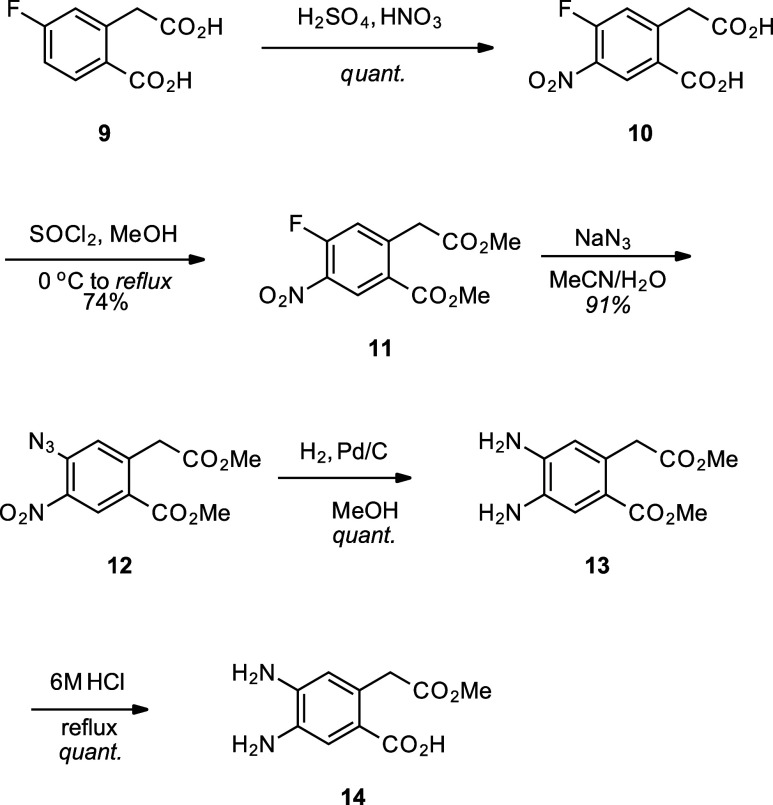
Synthesis of Asymmetric Methyl Ester **14**

We then attempted to esterify molecule **10** in preparation for S_N_Ar azidation and to alleviate difficulties with purifying carboxylic acids. Initial esterification involved attempts using coupling agent diisopropylcarbodiimide (DCC) under mild basic conditions of pyridine in MeCN. Unfortunately, molecule **10** was unstable under basic coupling conditions and so we instead attempted to esterify under acidic conditions. While the monoester was easily made, the diester could only be made in small amounts when ethanol, methanol, or isopropanol was used as the solvent, even after incorporating a Dean–Stark apparatus. We suspect that a trace amount of water prevented the complete conversion to the diester. To evaluate this hypothesis, we attempted the Fischer esterification with *n*-butanol at 110 °C. Ideally water would slowly evaporate in *n*-butanol allowing the equilibrium to shift toward the diester. Gratifyingly, this esterification route in the less volatile *n*-butanol was observed to be more robust, producing the dibutyl ester in good crude yield. However, *n*-butanol was difficult to remove by vacuum. Much of the product was lost during silica gel purification and the high reaction temperatures resulted in undesired byproducts. For these reasons we sought a more efficient esterification process. Refluxing **10** with thionyl chloride in methanol afforded **11** in good yield without the need for further purification. We then attempted to install the azido-group in place of the fluorine so that it could later be reduced along with the nitro group to the 1,2-dianiline target **13**. Initial attempts involved reacting **11** with sodium azide in DMSO, which is known to have robust reactivity in S_N_Ar reactions. Unfortunately, the reaction yielded a plethora of reaction byproducts, and we suspect that the nitro aryl azide **12** was electrophilic enough to keep reacting after it was formed. Thus, we ran the reaction with a small excess of NaN_3_ (1.1 equiv). To our surprise, the NMR spectrum of the reaction products still showed several peaks consistent with a mixture of products and complete consumption of starting materials. To help suppress formation of byproducts, we switched solvent to a mixture of acetonitrile and water. S_N_Ar intermediates are less stable in acetonitrile than in DMSO,^25^ and sodium azide is a weaker nucleophile in protic solvents like water due to ion–dipole interactions stabilizing the azide anion.^26^

We hypothesized that utilizing a dual solvent system of acetonitrile and water would allow for solvation of **11** and a weakening of azide nucleophilicity. Gratifyingly, these conditions resulted in the formation of **12** in good yields after 2 days. The diester **12** was reduced uneventfully to the corresponding *o*-phenylenediamine **13** under hydrogenation conditions with 5% Pd/C. To our surprise, while we were able to convert the benzoic ester to its corresponding acid, the methyl phenylacetate remained after refluxing in acid. Additional attempts to deprotect the resulting methyl ester under basic conditions led to decomposition. Compound **14** would serve as an interesting candidate for determining the potential steric impact on I-PCET reactions when compared to **2**. To further efforts toward **2**, we opted to utilize a benzyl protecting group with the intent to simultaneously reduce the azide and nitro groups and deprotect the ester group in a single step (Scheme 4). Subjecting **10** to thionyl chloride in benzyl alcohol yielded monoprotected benzyl ester **15** at the benzoic position, to our surprise. This, along with our unsuccessful attempts to fully deprotect **13**, suggests a significant difference in reactivity between the benzoic and phenylacetic acid functional groups. Nevertheless, ester **15** underwent S_N_Ar under the previously mentioned conditions to yield **16** after 3 days. It should be noted that the reaction seems to be suppressed by residual benzyl alcohol and therefore care must be taken to remove all benzyl alcohol during the purification process. Alternatively, proton exchange between the azide anion and the phenylacetic acid group could also diminish the nucleophilicity of the azide, slowing the S_N_Ar reaction. Finally, **16** was reduced to our target diacid **2** under the previously mentioned hydrogenation conditions.

**Scheme 4:**
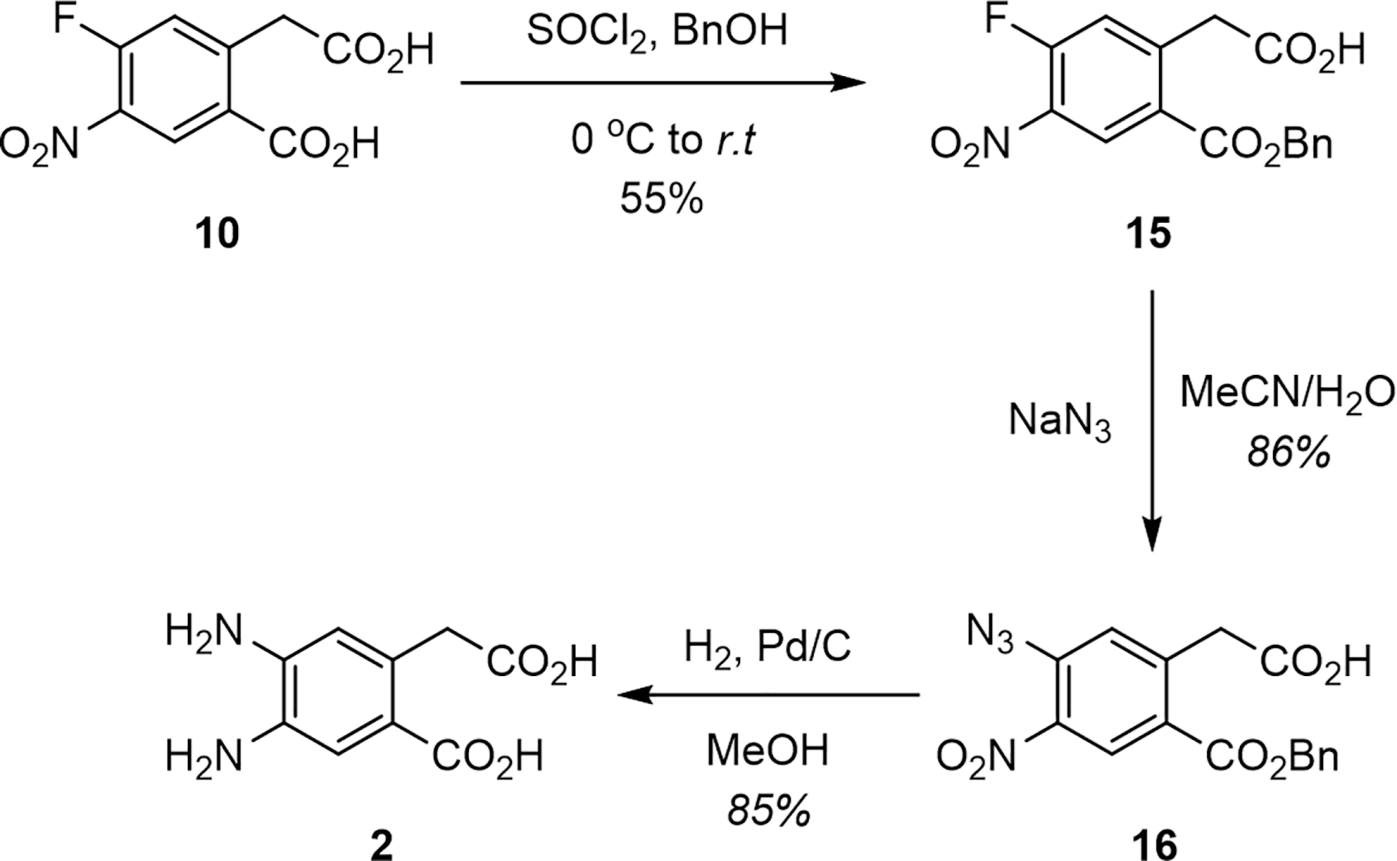
Synthesis of **2** through Benzyl Ester Protection and Deprotection Method

### S_N_Ar/S_N_Ar Strategy

The EAS/S_N_Ar strategy showed that S_N_Ar was a good approach to synthesizing 4,5-*o*-phenylenediamine precursors. However, nitration of a highly electrophilic aromatic ring remained a tedious process. Therefore, we attempted to circumvent nitration completely, opting to azidate a difluorinated compound **S1** (Scheme S1). Initial efforts showed that the benzylic carbon was successfully azidated at room temperature, yielding **S2**. Unfortunately, the aromatic ring remained unreactive even at 70 °C after 24 h with excess NaN_3_ but was found to undergo the second substitution after 1 month. These results suggest that at least one nitro group is necessary for the facile S_N_Ar azidation on these 1,2,4,5-aromatic ring systems. Using lessons learned from our previous synthetic efforts, we proposed a strategy for the modular synthesis of 4,5-*o*-phenylenediamine precursors (Figure 2).

**Figure 2:**
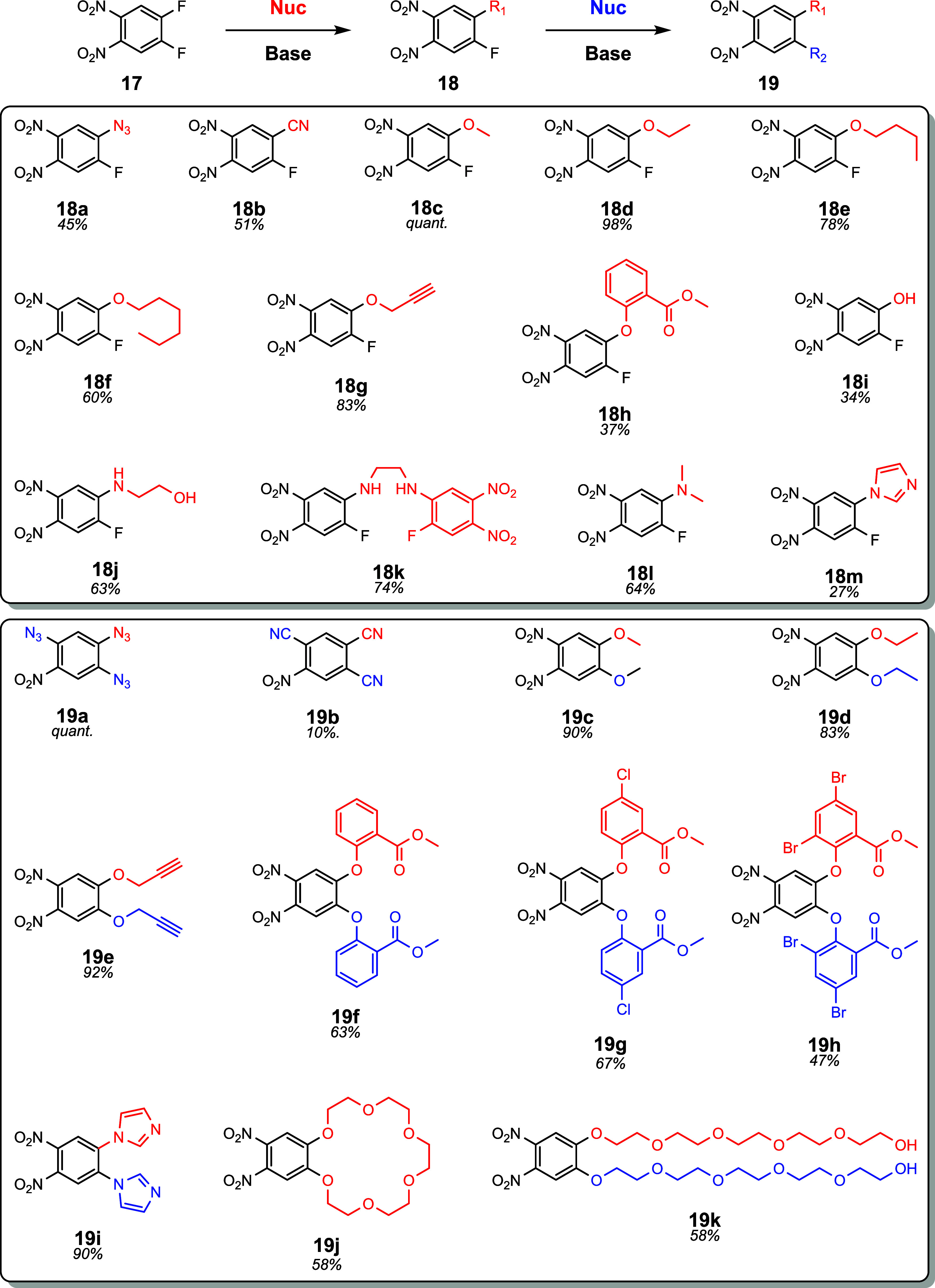
Synthesis of 4,5-*o*-Dinitrobenzenes by S_N_Ar/S_N_Ar Method.

Starting from 1,2-dinitro-4,5-difluorobenzene **17**, we can use S_N_Ar chemistry to install the desired functional groups and avoid less regioselective EAS reactions like nitration. *Ortho-*nitration by EAS can afford 1,2,3,4-*o*-phenylenediamines and would make interesting substrates, but they are less desired for our studies because of the potential substituent interactions with phenazine once coupled to the graphitic carbon surface. Similar work has been done using **17** to functionalize macromolecular systems, like cavitands^27^ and carbon nanotubes,^28^ through S_N_Ar of the aromatic fluorenes followed by further elaboration via the nitro/amine groups. We sought to leverage and expand on this strategy by utilizing stepwise S_N_Ar reactions to enable access to both asymmetric and symmetric *o*-phenylenediamine precursors. With this new strategy in mind, we first pursued azidation of **17**. Azidation allows synthetic access to an iminophosphorane that could be deprotected in the presence of the nitro groups via the Staudinger reaction.^29^ Additionally, the azide precursor **18a** can be used to click alkynes onto the precursor before the reduction to *o*-dianlines. We then attempted S_N_Ar cyanation, which we thought would be a simple way to install a protected nonconjugated amine or a masked conjugated carboxylic acid. Fluorene peak splitting patterns were analyzed to determine the extent of monosubstitution of **18a** (1–2 doublets) vs disubstitution of **19a** (1–2 singlets). Analysis of the product mixture showed that equimolar azide **18a** and cyano **18b** yielded monosubstituted products as well as trisubstituted products **19a and 19b**, both of which were separable by flash chromatography. The robust formation of triazide **19a** is in accordance with recent literature.^21^

S_N_Ar cyanations were attempted in alcohol during the screening process. Interestingly, these attempts resulted in the highly predominant monoalkoxylation. Even with excess alcohol present, methanol and ethanol gave 100:1 and 8:1 preference for monoalkoxylation **18c**–**d** over dialkoxylation **19c**–**d**, respectively. Encouraged by these results, we explored asymmetric alkoxylation for a series of alcohols with different degrees of hydrophobicity: methanol, ethanol, butanol, hexanol, propargyl alcohol, and methyl salicylate, **18c**–**h**. All alcohols except methyl salicylate required heat to react with 1,2-dinitro-4,5-difluorobenzene. Alcohol hydrophobicity was observed to correlate inversely with the extent of monoalkoxylation. Propargyl alcohol monoalkoxylated in good yields while methyl salicylate produced a mixture of starting material **17**, monoalkoxylated **18**, and dialkoxylated products **19**. Interestingly, the S_N_Ar reaction failed to proceed with an excess of hexanol. These results suggest that solvent polarity plays a distinct role in stabilizing the polar intermediate needed to initiate the S_N_Ar reaction. Monosubstituted products **18e** and **18f** required the use of only 1–2 equiv of hydrophobic alcohol in DMSO, along with K_2_CO_3_ as the base. Desired aliphatic ether products were not observed when KHCO_3_ was used as the base in wet DMSO, and instead resulted in the production of hydroxylated **18i**. Based on this result, in some cases we switched to Hunig’s base (DIPEA) as a more selective base.

We hypothesize that the ability to access monosubstituted products **18** is a result of the change in ring electronics after monosubstitution and that the removal of the first fluorine from the ring slightly deactivates the ring for the subsequent S_N_Ar reaction. This deactivation after the first step gives a kinetic preference to the reaction of difluoro **17** over the further reaction of monosubstituted products. In the case of azide and cyanide, this preference for monosubstitution of the aromatic ring is not seen. Instead, the rate of substitution appears to increase after the first azidation of **17** to **18a**, favoring the formation of disubstituted and trisubstituted products.

Next, we examined symmetric dialkoxylation. Methanol, ethanol, butanol, and hexanol were all heated with **18** in excess K_2_CO_3_ and alcohol, resulting in **19c**–**d**. As the alcohols increased in hydrophobicity, we observed a decrease in the rate of alkoxylation. After 14 h at 70 °C, 1-hexanol and 1-butanol would not react in neat solvent and barely reacted even in 0.2 M solutions with 50% DMSO. We suspected that the hydrophobicity of the solvent was the limiting factor. Comparatively, both ethanol and methanol reacted completely in excellent yields after 1 day. We then synthesized a series of methyl salicylates. Salicylates are known to function as ligands for transition-metal cations and could display interesting electrochemical properties after conjugation to the graphitic carbon surface because of their ion binding activities.^30^ Overall, the disalicylate ester products **19f**–**h** were made in excellent yields. The dibromo **19h** was easily separated in that the product did not dissolve in methanol like the methyl dibromo salicylate starting material. All alcohols that underwent monosubstitution could also undergo the second less activated substitution. These results validate that the two nitro groups are withdrawing enough to induce affinity for anionic attack.

We became interested in S_N_Ar aminations because aniline and amines could potentially bind anions instead of cations, giving access to another series of electrochemical experiments. Unlike alkoxylations, monoaminations occur at room temperature and in modest yields to form **18j**–**m**. We hypothesize that this is a result of the more nucleophilic nature of amines in comparison to alcohols. Disubstitution attempts were not as trivial. For example, the reaction with excess amine for 14 h at 70 °C resulted in exclusively monoamination products in the presence of ethylenediamine and ethanolamine. Similarly, cyclization of **18j** and **18k** was not observed under the tested reaction conditions after heating to 70 °C for over 48 h. While most amines did not produce the disubstituted analogs, imidazole yielded disubstituted product at room temperature with just 1.5 equiv of amine. In acetone, excess imidazole almost exclusively yielded the disubstituted product **19i**.

We then explored PEGylation reactions through the S_N_Ar route for their potential to form groups that could potentially bind cations. Synthesis with 1 equiv of pentaethylene glycol resulted in exclusive formation of the monoalkoxylated PEG as evidenced by *in situ* NMR spectroscopy. However, after concentrating and purifying the reaction mixture, crown ether **19j** was recovered in reasonable yields. We suspect that the mono substituted ring can further react when concentrated by heating in the presence of base. Reaction of **17** with excess equivalents of pentaethylene glycol and base resulted in dialkoxylated PEG **19k**. These findings show that **17** can be PEGylated in good yields and cyclization of mono-PEGylated molecules to the corresponding crown ether can occur while concentrating under reduced pressure.

Overall, this S_N_Ar route gave facile access to *o*-phenylenediamines with amine functionality on the opposite side. However, the second substitution step was significantly slower after the first amination. We hypothesize that the electron donating effect of the amine makes the aromatic system more electron rich, deactivating the aromatic ring for the second S_N_Ar reaction. This would explain the robust second amination demonstrated for **19i** due to greater electron delocalization from the imidazole.

### Electrochemical Characterization

To validate that our new 4,5-*o*-phenylenediamine molecules could be conjugated to conductive graphitic carbon and exhibit similar electrochemical properties as commercially available 3,4-diaminobenzoic acid (**COOH**), we modified GC with each of **14** and **COOH** using previously reported procedures.^2^ Electrochemical evaluation using cyclic voltammetry (CV) in a three-electrode configuration revealed three quasi-reversible waves, each assumed to correspond to an I-PCET reaction based on the number of protic sites in **14** and **COOH**. Simultaneous integration of all three redox features at slow scan rate (0.1 V/s) suggested molecular active site surface coverages for the carboxylic acid groups of (2.0 ± 0.6) × 10^–11^ mol/cm^2^ and (2.1 ± 0.5) × 10^–10^ mol/cm^2^, respectively, when assuming a constant capacitive baseline.^2^ We hypothesized that voltametric responses of **GC-14** and **GC-COOH** would be similar because the *o-*methyl ester moiety of 4,5-functionalized **14** would not be electronically coupled to the underlying graphitic substrate due to an intervening methylene. The three quasi-reversible waves were observed for a wide range of aqueous pH values, with half-wave potentials (*E*_1/2_) for **GC-14** of +0.37 V vs RHE, + 0.16 V vs RHE and – 0.01 V vs RHE (Figure 3). When compared to **GC-COOH**, which displayed redox features at +0.33 V vs RHE, + 0.16 V vs RHE and a shoulder at +0.10 V vs RHE, a notable shift in *E*_1/2_ of the most negative redox feature toward even more reductive potentials is observed. This **GC-COOH** feature has previously been attributed to the protonation and deprotonation of one of the pyrazine nitrogen moieties.^7^ All redox waves display an approximately Nernstian slope (∼−60 mV/pH) in *E*_1/2_ suggesting a reversible 1H^+^/1e^–‑^ stoichiometry (Figure S1a,b). The continuity of this Nernstian slope across the pH range suggests that p*K*_a_ values of the surface groups are directly influenced by the electrode potential via charging of the electric double layer. The reversibility of these redox events is further supported by the relatively small peak separation (Δ*E*_peak_) observed at slow scan rates (0.1 V/s) (Figure S1c) and the near unity peak current ratios for each redox feature at each of the 3 pH values tested (Figure S1d). The waves present at the most positive potential scanned are most likely due to I-PCET involving the installed carboxylate/carboxylic acid group. Presumptive hydrogen bond stabilization of the carboxylic acid on **GC-14** by oxygen atoms on the *o*-methyl ester group are consistent with its slightly more positive *E*_1/2_ value.

**Figure 3:**
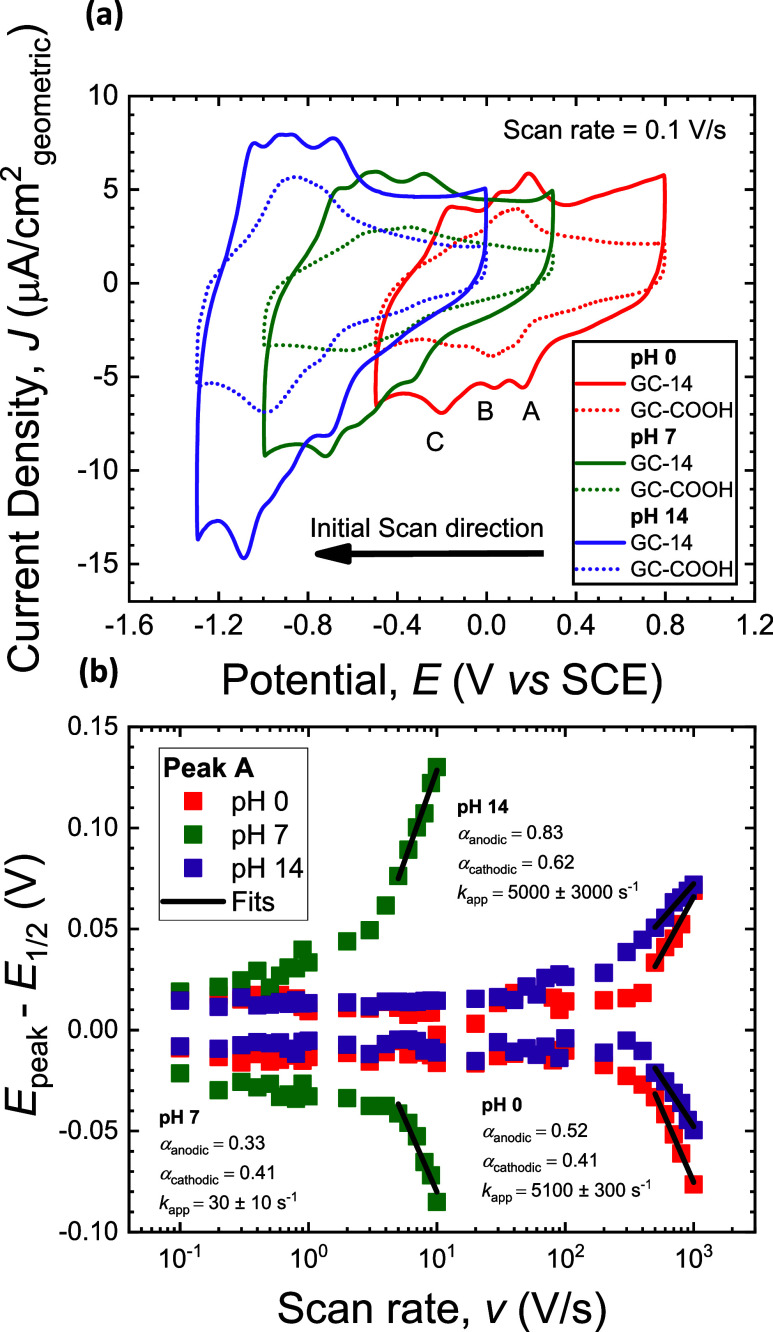
Cyclic voltammetry evaluation of **GC-14** and **GC-COOH** using various aqueous pH conditions. (a) Cyclic voltammograms (IUPAC convention) recorded at 0.1 V/s for two different glassy carbon (GC) working electrodes (initial electrode potentials of 0.8 V vs SCE at pH 0, 0.3 V vs SCE at pH 7, and 0.0 V vs SCE at pH 14), each immersed in room-temperature aqueous pH 0 (1 M HClO_4_), pH 7 (1 M Britton–Robinson buffer as 0.1 M sodium acetate, 0.1 M sodium phosphate dibasic, 0.1 M boric acid, and 0.7 M sodium perchlorate), or pH 14 (1 M NaOH), and whose current densities are normalized to the geometric area of the electrode. The three waves are each ascribed to a reversible I-PCET reaction to each of the two pyrazine nitrogen atoms or one carboxylate. Data for **GC-COOH** were scaled via division by 15 to help visualize current densities on the same scale, an outcome that we suspect is due to a significantly lower surface coverage of carboxylic acid groups on **GC-14**. (b) Trumpet plot analysis of peak A in cyclic voltammograms of **GC-14**. Half-wave potentials (*E*_1/2_) were subtracted from peak potentials (*E*_peak_) and plotted semilogarithmically vs the scan rate (*v*). Nonlinear least-squares best fits to eq 1 are displayed as black lines over the range of scan rates analyzed. The three-electrode configuration consisted of a modified GC working electrode (0.175 cm^2^ geometric surface area), a saturated calomel reference electrode (SCE), and a cylindrical graphite rod counter electrode (2.5 mm diameter). Voltammograms for untreated (as received) glassy carbon electrodes are included as Figure S1 for reference.

Kinetic parameters in the form of apparent rate constants (*k*_app_) and charge transfer coefficients (α) were determined from the dependence of the peak potentials on scan rate (*v*) using the Laviron formalism,^31^ as follows,

$$E_{\mathrm{peak},i}=E_{1/2}\pm (\frac{\mathrm{RT}}{\alpha_{i}\mathrm{nF}})\;\ln\left(\frac{\alpha_{i}\mathrm{nFv}}{k_{\mathrm{app}}\mathrm{RT}}\right)$$ 1

where *i* = anodic (+) or cathodic (–) and we report *k*_app_ values as the mean ± standard deviation for each pH value evaluated. Because the accuracy of this approximate equation benefits from significant peak separation, data for best fitting was restricted to the 5 fastest scan rates reported (5–10 V/s at pH 7 and 500–1000 V/s at pH 0 and 14). For each of pH 0 and pH 14, *k*_app_ for I-PCET involving the carboxylate group of **GC-14** was determined to be on the order of 10^3^ s, while at pH 7, *k*_app_ was observed to be significantly smaller, on the order of 10^1^ s. These values of *k*_app_ are similar to those reported previously for **GC-COOH**,^2^ with the largest difference being at pH 0 where that for **GC-14** was observed to be approximately four times smaller. We hypothesize that this may be a result of steric hindrance and/or increased electric double layer thickness due to the *o*-methyl ester group present in **GC-14**. Our best-fit procedure did not require that charge transfer coefficients sum to one, which in general is a suitable assumption for IIT reactions.^32^ Irrespective, our resulting best-fit α values (Figure 3b) should not be overinterpreted given the general insensitivity of α values deduced from best fits of cyclic voltammetry data under the Laviron formalism.^31^ Different from our recent reports that used this analysis for I-PCET reactions at modified GC electrodes,^2,33^ herein we did not perform an iterative global best fit of simulated trumpet plots to the experimental (*E*_peak_ – *E*_1/2_) data. We determined that this level of rigor is not necessary to establish *k*_app_ values consistent with a ∼100-fold increase in scan rate at which (*E*_peak_ – *E*_1/2_) values deviate from those at slow scan rate for pH extremes of 0 and 14 relative to the intermediate pH value of 7.^31,2^ Collectively, our data validate the suitability of our new synthetic methodology to obtain a versatile substrate scope that can be electronically coupled to graphitic electrodes for electrochemical evaluation. The large family of *o*-phenylenediamine precursors synthesized here provides the basis for evaluating structure–function correlations in IIT reactions in ongoing and future studies.

## Conclusions

Recent advances in graphitic electrode modification have made *o*-phenylenediamines a desirable class of small molecule synthons for functional electrodes. In this work, we explored three strategies for creating a diverse array of *o*-phenylenediamines. Dinitration strategies that involve two EAS reactions were determined to not be functionally tolerant. As a result, few functional groups were shown to be compatible with this strategy. A nitration (EAS) and azidation (S_N_Ar) strategy of a trisubstituted haloaromatic ring was determined to be a feasible route to custom asymmetric 4,5-*o*-phenylenediamines. Routes with two S_N_Ar reactions, utilizing 4,5-difluoro-*o*-dinitrobenzene, yielded a wide variety of 4,5-*o*-phenylenediamine precursors in just 2–4 steps. Electrochemical experiments confirmed that these asymmetric 4,5-*o*-phenylenediamines can be conjugated to graphitic electrodes and facilitate I-PCET when polarized electrochemically. Future electrochemical investigations powered by these synthetic routes will be used to develop structure–function correlations in interfacial ion transfer.

## Experimental Section

### Safety Statement

This work did not contain any unexpected, new, or significant hazards or risks beyond those commonly expected to be present in a laboratory setting.

### Electrode, Electrolyte, and Cell Preparation

Unpolished glassy carbon wafers (1 mm × 25 mm × 25 mm) (>99.9% purity, MSE Supplies, LLC) were scored with a diamond-tipped scribe, cut into shards (0.1 cm × ∼0.25 cm × ∼0.5 cm), functionalized with *o*-phenylenediamine substrates, and characterized electrochemically. The procedure to functionalize glassy carbon and prepare the electrodes follows that reported previously^2^ and is summarized here: glassy carbon shards were functionalized with *o*-phenylenediamines by suspending the desired *o*-phenylenediamine (200 μmol) in 10 mL ethanol in a scintillation vial, placing a freshly cut shard face-up at the bottom of the vial, sealing the vial under an Ar atmosphere, and heating it to 60 °C for 48 h. Following the functionalization, shards were transferred to a separate vial and serially soaked in each of ethanol, aqueous 0.1 M HClO_4_, acetonitrile, and ultrapure water. Each soak was performed for 30 min, followed by a quick rinse with ultrapure water. Subsequently, each modified glassy carbon shard was dried under a stream of Ar, bound to a copper wire using carbon conductive cement (Thermo Fisher Scientific), and dried at 60 °C in an oven overnight. To ensure that only the modified glassy carbon was in contact with the liquid electrolyte solution, the copper wire was bent into a ‘U’ shape and dipped into molten paraffin wax (Royal Oak Enterprises, LLC), coating the middle section of the wire and carbon cement, but allowing both sides of the glassy carbon portion to remain uncovered by the insulating wax. This ensured that only the lower portion of the functionalized glassy carbon wafer (0.03 cm thickness) could be exposed to the electrolyte solution.^2^ After allowing the wax to cool for ∼30 s, the wax-coated wire was straightened out for use as the working electrode in a three-electrode configuration.^2^

All electrochemical measurements were performed using a potentiostat (VSP-300, Biologic), in a flat-bottomed 20 mL scintillation vial that was sealed with a rubber septum and contained 15 mL of room-temperature aqueous electrolyte that was sparged for 30 min prior to experimentation via vigorous bubbling with ultrapure Ar gas (99.999% purity, UHP, Linde). During electrochemical experiments, the Ar flow rate was decreased to minimize effects due to convection of the electrolyte. A three-electrode configuration was used that consisted of a modified graphitic carbon (>99.9% purity, MSE Supplies, LLC) working electrode (0.175 cm^2^ geometric surface area), saturated calomel (fritted and filled with aqueous saturated KCl) (CH Instruments, Inc.) reference electrode (SCE), and unpolished cylindrical graphite rod (99% purity, Alfa Aesar) counter electrode (2.5 mm diameter), each passed through prefabricated holes in the rubber septum. Cyclic voltammetry was performed at a scan rate of 0.1 V/s with an initial potential of 0.8 V vs SCE at pH 0, 0.3 V vs SCE at pH 7, and 0.0 V vs SCE at pH 14. Initial scan directions were negative, with a switching potential that was −1.3 V from the initial potential. Electrode potentials are reported vs SCE or were converted to vs RHE via the following equation: *E* (RHE) = *E* (SCE) + 0.241 V + (0.0592 V × pH). Aqueous alkaline solutions were prepared using NaOH pellets (99.99% purity, Sigma-Aldrich) and aqueous acidic solutions were prepared using perchloric acid (99.999% SC grade, Sigma-Aldrich), each in ultrapure water (Millipore Sigma, Milli-Q Reference Water Purification System). Britton–Robinson buffer solution was prepared using 0.1 M sodium acetate (99.995% purity, Sigma-Aldrich), 0.1 M sodium phosphate dibasic (99.95% purity, Sigma-Aldrich), 0.1 M boric acid (99.999% purity, Sigma-Aldrich), and 0.7 M sodium perchlorate (99.99% purity, Sigma-Aldrich). The desired pH 7 condition was obtained via titration using aqueous acid or base, verified using a calibrated pH probe with reported precision of ±0.001 pH units (meter HI5222 with probe HI1131B, Hanna Instruments, Inc.). Strongly acidic and alkaline aqueous electrolytes used 1 M acid and base, respectively, and are reported as pH 0 and pH 14 herein.

## Supplementary Material



## Data Availability

The data underlying this study are available in the published article and its Supporting Information.

## Notes

Additional electrochemical analyses; synthetic procedures;^1^H and ^13^C­{^1^H} NMR spectra for new compounds; melting point analyses; HRMS; GCMS (PDF)
