# Supplementary material for: Synthesis of 4,5-Disubstituted o‑Phenylenediamines: An Enabling Platform for Electrochemical Investigations of Interfacial Ion Transfer Reactions
Source: J Org Chem. 2025 Dec 8;90(50):17626–34. doi: 10.1021/acs.joc.5c00538 (PMC12723685; doi:10.1021/acs.joc.5c00538)

Supporting Information

Synthesis of 4,5-Disubstituted *o*-Phenylenediamines: An Enabling Platform for Electrochemical Investigations of Interfacial Ion Transfer Reactions

Dennis Tang,[a] Nathaniel Keyes,[a] Jasmin C. Rose,[b] John E. Gonzales,[b] Munho Yang,[c] Marco D. Giles,[b] Yogesh Surendranath,[d,e] Shane Ardo,[a,c,f]* Matthew B. Minus[b]*

[a]Department of Chemistry, [c]Department of Materials Science and Engineering, [f]Department of Chemical and Biomolecular Engineering, University of California Irvine, Irvine, CA 92697 USA

[b]Department of Chemistry, Prairie View A&M University, 100 University Drive, Prairie View, TX 77446 USA

[d]Department of Chemistry, [e]Department of Chemical Engineering, Massachusetts Institute of Technology, 77 Massachusetts Ave, 18–163, Cambridge, MA 02139 USA

maminus@pvamu.edu; ardo@uci.edu

Table of Contents

I. Experimental Information S2

Materials and Methods S2

Cyclic Voltammetry S3

Experimental Procedures and Characterization Data S4

II. References S25

III. NMR Spectroscopy Data S26

I. Experimental Information

Materials and Methods

All reactions were carried out in flame or oven dried glassware open to air unless otherwise noted. Solvents (ACS certified grade or better) for reactions, workup, and chromatography were purchased and used without further purification. 100% HNO_3 was made by distillation from H_2SO_4 and KNO_3. Homophthalic acid (>99.0%) was purchased from TCI Chemicals. Palladium on carbon (5 wt. % loading (dry basis)), benzoyl chloride (ReagentPlus®, ≥99%), lithium aluminum hydride (powder, reagent grade, 95%), acetyl chloride (reagent grade, 98%), triethylamine (reagent grade, 98%), oleum (reagent grade, 20%, as free SO_3), sulfuric acid (ACS reagent, 95.0-98.0%), nitric acid (ACS reagent, 70%), hydrochloric acid (37%, ACS reagent), thionyl chloride (reagent grade, 97%), sodium azide (ReagentPlus®, ≥99.5%), and benzyl alcohol (ReagentPlus®, ≥99%) were purchased from Sigma Aldrich. Potassium cyanide, triethylene glycol, pentaethylene glycol, 4,5-difluoro-1,2-dinitrobenzene, 2-(carboxymethyl)-5-fluorobenzoic acid, methyl salicylate, 4-chloromethyl salicylate, 3,5-dibromo methyl salicylate, propargyl alcohol, butanol, isopropanol, hexanol, ethanol, methanol, potassium carbonate, potassium bicarbonate, potassium phosphate, ethylene diamine, ethanolamine, and potassium hydroxide were purchased from Fischer Scientific. All solvents were purchased from Fisher Chemical and were ACS grade or higher. Analytical thin layer chromatography was performed using silica gel 60 F_{254} on aluminum sheets with ultraviolet light for visualization. Flash column chromatography was performed on Fisher Chemical™ Silica Gel Sorbent (230-400 Mesh, Grade 60) silica gel. Yields refer to chromatographically and spectroscopically homogeneous materials, unless otherwise stated.

NMR spectra were recorded on Bruker Avance 400 MHz, Bruker Avance 500 MHz, and Bruker 600 MHz instruments obtained at 298 K unless otherwise noted and referenced to residual $CDCl_3$ (7.26 ppm, ^1H; 77.16 ppm, ^{13}C), CD_3OD (3.31 ppm, ^1H; 49.00 ppm, ^{13}C), $(CD_3)_2SO$ (2.50 ppm, ^1H; 39.52 ppm, ^{13}C), $(CD_3)_2CO$ (2.05 ppm, ^1H; 29.84 ppm, ^{13}C), or D_2O (4.79 ppm, ^1H). Chemical shifts are reported in ppm with the following abbreviations to explain multiplicities: s = singlet, d = doublet, t = triplet, quin = quintuplet, dd = doublet of doublets, tt = triplet of triplets, td = triplet of doublets, m = multiplet, bs = broad singlet. All coupling constants are apparent J values measured at the indicated field strengths and reported in Hertz (Hz). High-resolution mass spectra (HRMS) were recorded on a Waters LCT Premier spectrometer using ESI-TOF (electrospray ionization-time of flight) and data are reported in the form of (m/z). Elemental Analysis was performed on the Flash*Smart*™ Elemental Analyzer in the TEMPR Lab at UC Irvine. High resolution mass analyses were conducted with the Agilent qToF LC-MS in the Mass Spectrometer Facility at Rice University and the Thermo Scientific QE Focus in the Mass Spectrometer Facility at Texas A&M University.

Cyclic Voltammetry

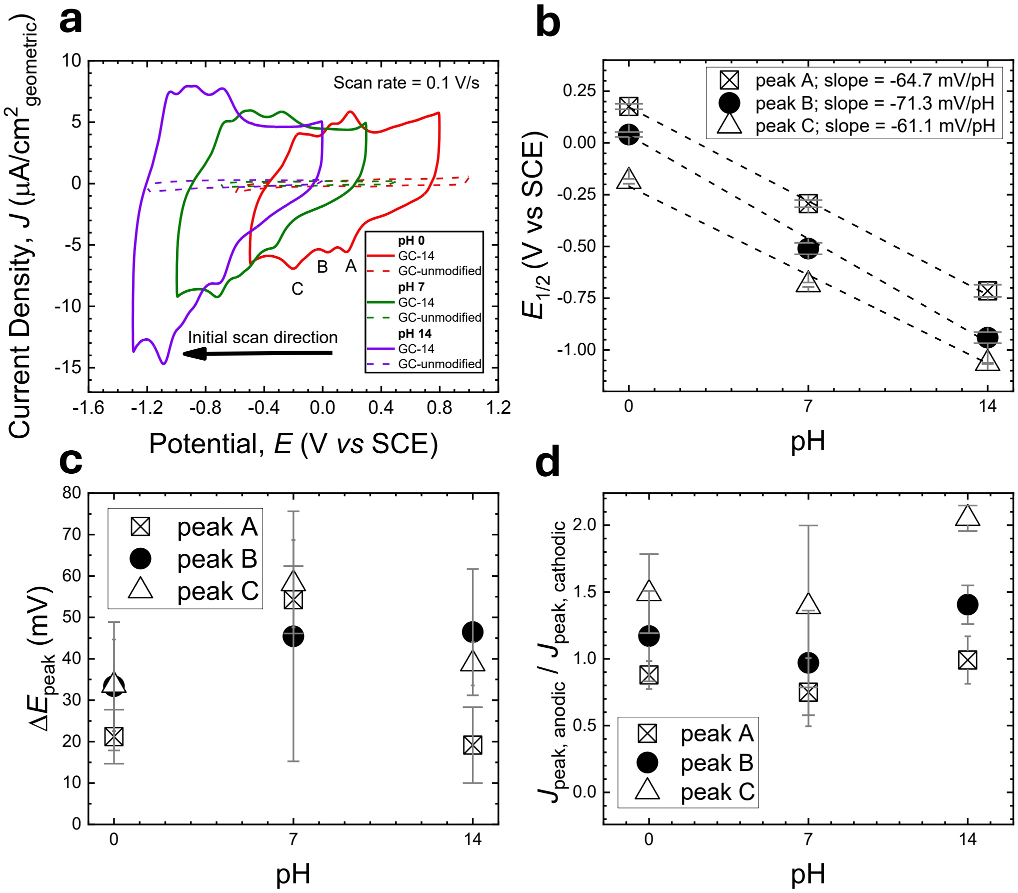

Figure S1. Cyclic voltammetry evaluation of GC-14 and unmodified GC using various aqueous pH conditions and a scan rate of 0.1 V/s. **(a)** Cyclic voltammograms (IUPAC convention) recorded at 0.1 V/s for two different glassy carbon (GC) working electrodes, each immersed in room-temperature aqueous pH 0 (1 M $HClO_4$), pH 7 (1 M Britton–Robinson buffer), or pH 14 (1 M NaOH), and whose current densities are normalized to the geometric area of the electrode. Surface modification of GC with **14** resulted in an approximately order-of-magnitude increase in double-layer capacitive current (35 μF/cm^2 for **GC-14**; 3.4 μF/cm^2 for **GC-unmodified**). **(b)** Plot of half-wave potential ($E_{1/2}$) of the three quasi-reversible redox features present in voltammograms of **GC-14** (labeled A, B and C in **panel a**), where each exhibits a nearly Nernstian pH dependence (i.e. -60 mV/pH) suggesting a redox reaction stoichiometry for H^+ and e^- that is 1:1. Linear least-squares best-fit lines were determined from the data before averaging. **(c)** Plot of peak separation (ΔE_{peak}), calculated as the difference in peak anodic potential and peak cathodic potential, for each redox wave of **GC-14** at each aqueous pH value assessed. Larger peak separations at pH 7 and 14, relative to pH 0 conditions, suggest slower rates of interfacial proton-coupled electron transfer under these conditions, which agree with our previous results.[S1] **(d)** Plot of peak current density ratio for anodic and cathodic peaks ($J_{peak,anodic}$ / $J_{peak,cathodic}$) at each aqueous pH value assessed. Because values are near unity, reaction kinetics are deduced to be approximately reversible. All data are reported as the mean ± standard deviation from five different electrodes, each measured at each indicated aqueous pH value. The three-electrode configuration consisted of a glassy carbon working electrode (0.175 cm^2 geometric surface area), a saturated calomel reference electrode (SCE), and a cylindrical graphite rod counter electrode (2.5 mm diameter).

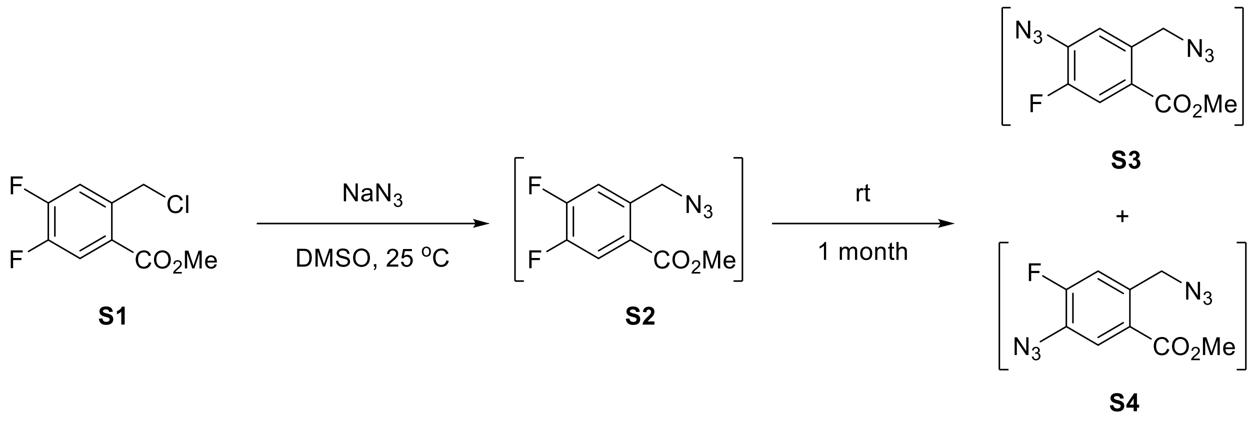

Scheme S1. Azidation of difluoro compound S1

The starting material **S1** (0.05 g, 0.21 mmol) was placed in a 1 dram vial along with sodium azide (0.1 g, 1.53 mmol). DMSO-d_6 (1 mL) was added to the reaction vessel and the reaction was allowed to stir at room temperature. The reaction mixture (600 μL) was pipetted into a standard NMR tube and the reaction was examined using NMR spectroscopy (**Figure S2b**). The NMR sample was recombined with the original reaction mixture and was allowed to stand at room temperature for 1 month. The reaction was then analyzed again using NMR spectroscopy which indicated the formation of **S3** and **S4** (**Figure S2c**).

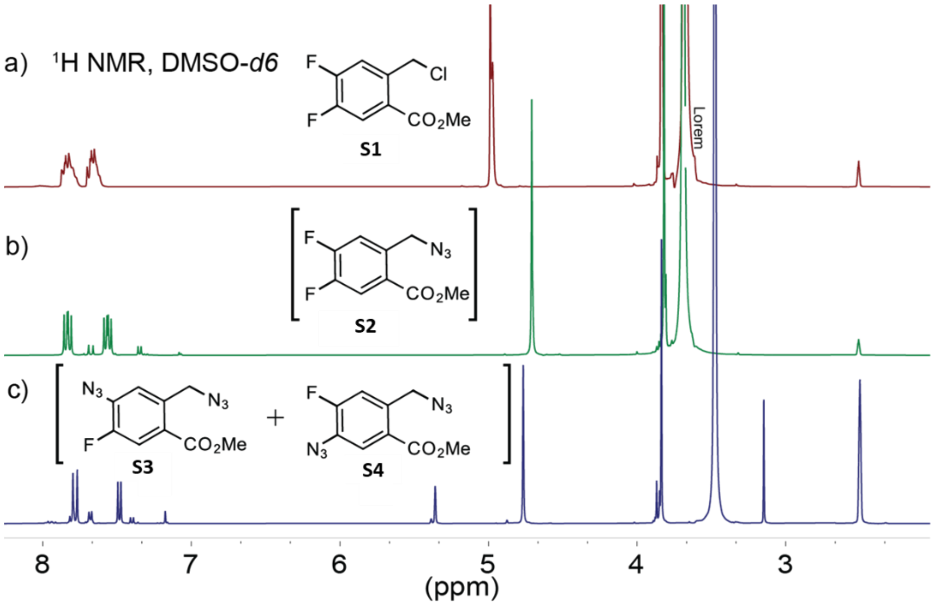

Figure S2. *In situ* NMR spectroscopy associated with **Scheme S1**.

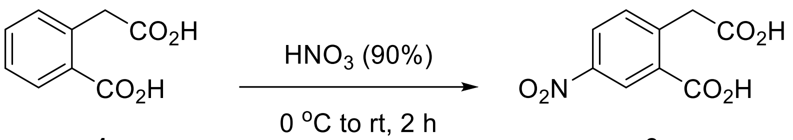

To a 20 mL 3-neck round bottom flask equipped with a stir bar and thermometer was added HNO_3 (90%) (20 mL). The flask was cooled to 0 °C *via* salted ice bath before **homophthalic acid 1** (6.25 g) was added slowly in portions while keeping the temperature > 20 °C and the resulting suspension was allowed to stir at room temperature for 2 h. The suspension was then poured onto ice and vacuum filtered. The solid was subsequently washed with H_2O and allowed to dry on the filter paper under vacuum for 2 h or until dry to yield **nitro-homophthalic acid 3** (5 g, 64%) as a white powder.

Nitro-homophthalic acid 3

^{1}H NMR (500 MHz, CD_3OD) δ 8.82-8.81 (d, 1H, *J* = 2.46 Hz), 8.36-8.34 (dd, 1H, *J* = 8.41, 2.52 Hz), 7.60-7.58 (d, 1H, *J* = 8.46 Hz), 4.17 (s, 2H) ppm; **^{13}C{1H} NMR** (150 MHz, CD_3OD) δ 175.5, 170.4, 137.9, 133.5, 133.3, 132.2, 131.5, 128.3, 41.3 ppm; **M.P.**: 210-215 °C (decomposition); **HRMS (ESI-TOF)** m/z: $[M-H]^-$ Calcd for $C_9H_6NO_6$ 224.0273; Found 224.0270.

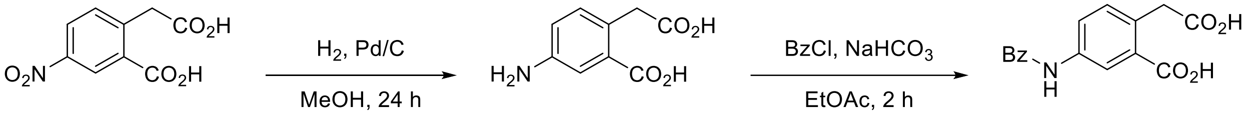

To a flame dried 100 mL 3-neck round bottom flask equipped with a stir bar under $Ar_{(g)}$ atmosphere was added 5% palladium on carbon (0.2 g), followed by a solution of **nitro-homophthalic acid 3** (2.25 g, 10 mmol) in MeOH (10 mL). The $Ar_{(g)}$ atmosphere was then purged 3× with $H_{2(g)}$ *via* balloon and the suspension was allowed to stir at room temperature for 24 h. After 24 h a white precipitate formed. The flask was then opened to the atmosphere and equipped with a reflux condenser. H_2O (25 mL) was then added, and the suspension was then refluxed for 5 min to dissolve the precipitate. While still hot, the suspension was vacuum filtered, and the filtrate was concentrated under reduced pressure to yield **amino-homophthalic acid S5** (1.26 g, 65%) as a yellow powder.

Amino-homophthalic acid S5

^{1}H NMR (500 MHz, $(CD_3)_2SO$) δ 12.96-11.50 (bs, 2H, COO**H**), 7.13 (d, 1H, *J* = 2.49 Hz), 6.92-6.90 (d, 1H, *J* = 8.18 Hz), 6.66-6.64 (dd, 1H, *J* = 8.10, 2.52 Hz), 5.43-4.98 (bs, 2H, N**H**$_2$), 3.69 (s, 2H) ppm; **^{13}C{1H} NMR** (150 MHz, $(CD_3)_2SO$) δ 173.3, 168.8, 147.5, 132.7, 130.9, 123.3, 116.9, 115.8, 39.2 ppm; **M.P.** > 260 °C; **HRMS (ESI-TOF)** m/z: $[M-H]^-$ Calcd for $C_9H_8NO_4$ 194.0531; Found 194.0536.

To a 100 mL round bottom flask equipped with a stir bar was added saturated $NaHCO_{3(aq)}$ (10 mL), EtOAc (10 mL), and **amino-homophthalic acid S5** (1.16 g, 5.94 mmol). The solution was cooled to 0 °C *via* salted ice bath and BzCl (0.69 mL, 5.94 mmol) was added dropwise. The flask was then covered with a watch glass, and the suspension was allowed to warm to room temperature and stir for 2 h. A precipitate formed and additional solids were precipitated by addition of 1 M HCl (5 mL). The suspension was then vacuum filtered and washed with EtOAc. The solid was allowed to dry on the filter paper under vacuum for 2 h or until dry to yield **benzamide 4** (1.48 g, 83%) as an off-white powder.

Benzamide 4

^{1}H NMR (500 MHz, $(CD_3)_2SO$) δ 13.19-11.84 (bs, 1H, COO**H**), 10.38 (s, 1H, N**H**), 8.35-8.34 (d, 1H, *J* = 2.24 Hz), 7.99-7.97 (d, 2H, *J* = 7.13 Hz), 7.95-7.92 (dd, 1H, *J* = 8.28, 2.29 Hz), 7.62-7.59 (tt, 1H, *J* = 7.33, 1.05 Hz) 7.55-7.52 (t, 2H, *J* = 7.40 Hz), 7.30-7.29 (d, 1H, *J* = 8.38 Hz), 3.90 (s, 2H) ppm; **^{13}C{1H} NMR** (150 MHz, $(CD_3)_2SO$) δ 172.9, 170.2, 165.4, 137.6, 134.8, 131.6, 130.7, 129.7, 128.4, 127.7, 122.2, 121.8, 42.2 ppm; **M.P.** 210 °C; **HRMS (ESI-TOF)** m/z: $[M-H]^-$ Calcd for $C_{16}H_{12}NO_5$ 298.0793; Found 298.0790.

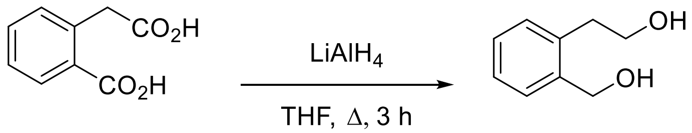

1 S6

To a flame dried 250 mL 3-neck round bottom flask equipped with a stir bar under $Ar_{(g)}$ atmosphere was added $LiAlH_4$ (1.89 g, 49.5 mmol, 3.3 equiv.) and THF (20 mL) slowly. A solution of **homophthalic acid 1** (2.70 g, 15 mmol) in THF (10 mL) was added slowly. The resulting suspension was heated to reflux *via* a silicone oil bath and allowed to stir for 3 h while the reaction progress was monitored *via* TLC (1:1 EtOAc/Hexanes; R_f = 0.17). Upon consumption of the starting material, the reaction was allowed to cool to room temperature before diluting with Et_2O (30 mL). The suspension was then cooled to 0 °C *via* salted ice bath and was quenched sequentially with H_2O (2 mL), 6 M (15%) NaOH (2 mL), and H_2O (2 mL) until bubbling ceased. The suspension was then allowed to stir vigorously for 10 min before $MgSO_4$ was added. The suspension was then vacuum filtered through a pad of Celite and washed with more Et_2O (ca. 15 mL). The solution was then concentrated under reduced pressure to yield **diol S6** (2.3 g, quant.) as a pale-yellow oil.

Diol S6

^{1}H NMR (500 MHz, CD_3OD) δ 7.36-7.65 (d, 1H, *J* = 7.13 Hz), 7.23-7.17 (m, 3H), 4.66 (s, 2H), 3.77-3.74 (t, 2H, *J* = 7.05 Hz), 2.94-2.91 (t, 2H, *J* = 7.05 Hz) ppm; **^{13}C{1H} NMR** (150 MHz, $CDCl_3$) δ 139.4, 138.3, 130.2, 123.0, 128.8, 127.0, 63.6, 63.3, 35.1 ppm; **HRMS (ESI-TOF)** m/z: $[M+Na]^+$ Calcd for $C_9H_{12}O_2Na$ 175.0735; Found 175.0747.

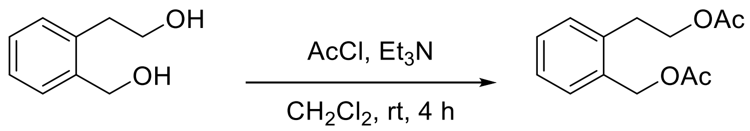

To a flame dried 100 mL 2-neck round bottom flask equipped with a stir bar under $Ar_{(g)}$ atmosphere was added a solution of **diol S6** (0.76 g, 5 mmol) in CH_2Cl_2 (25 mL). The solution was cooled to 0 °C *via* salted ice bath and Et_3N (1.74 mL, 12.5 mmol, 2.5 equiv.) was added. The solution was allowed to stir for 1 h before AcCl (0.89 mL, 12.5 mmol, 2.5 equiv.) was added dropwise. The yellow cloudy suspension was allowed to stir at room temperature for 4 h. The reaction was then quenched with H_2O and extracted with EtOAc. The organic extracts were then combined and washed with brine, dried over Na_2SO_4, and concentrated under reduced pressure. The crude product was purified by flash column chromatography (SiO_2, 1:9 EtOAc/Hexanes, R_f = 0.33) to afford **protected diol 6** as a pale-yellow oil (0.9 g, 76%).

Protected diol 6
^{1}H NMR (500 MHz, $CDCl_3$) δ 7.42-7.28 (m, 4H), 5.22 (s, 2H), 4.33-4.30 (t, 2H, *J* = 7.29 Hz), 3.07-3.04 (t, 2H, *J* = 7.30 Hz), 2.14 (s, 3H), 2.08 (s, 3H) ppm; **^{13}C{1H} NMR** (150 MHz, $CDCl_3$) δ 171.1, 171.0, 136.9, 134.3, 130.32, 130.30, 129.0, 127.2, 64.7, 64.5, 31.8, 21.2, 21.1 ppm; **HRMS (ESI-TOF)** m/z: $[M+Na]^+$ Calcd for $C_{13}H_{16}O_4Na$ 259.0946; Found 259.0950.

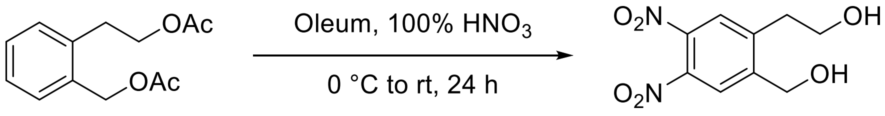

To a 50 mL 2-neck round bottom flask equipped with a stir bar and thermometer was added oleum (20 mL) which was cooled to 0 °C *via* salted ice bath. 100% HNO_3 (3 mL) was added dropwise while keeping the temperature > 15 °C. **Protected diol 6** (0.2 g, 0.85 mmol) was added very slowly, dropwise, while keeping the temperature > 10 °C and solution was then allowed to stir at room temperature for 24 h. The solution was then poured over ice and extracted with EtOAc. The organic extracts were then combined and washed with brine, dried over Na_2SO_4, and concentrated under reduced pressure. The crude product was purified by flash column chromatography (SiO_2, 1:4 EtOAc/Hexanes, R_f = 0.28) to afford **dinitro diol 7** (0.032 g, 18%) as a clear colorless oil. Similar results were obtained when **diol S6** was used as the starting material.

Dinitro diol 7
^{1}H NMR (500 MHz, CD_3OD) δ 8.80 (d, 1H, *J* = 2.39 Hz), 8.66 (d, 1H, *J* = 2.39 Hz), 5.85 (s, 2H), 4.83 (t, 2H, *J* = 6.71 Hz), 3.51 (t, 2H, *J* = 6.72 Hz) ppm; **^{13}C{1H} NMR** (125 MHz, $(CD_3)_2SO$) δ 143.4, 138.8,

129.1, 129.0, 120.4, 112.5, 63.2, 62.5, 17.9 ppm; **HRMS (ESI-TOF)** m/z: $[M+Na]^+$ Calcd for $C_9H_{10}N_2O_6Na$ 265.0505; Found 265.0509.

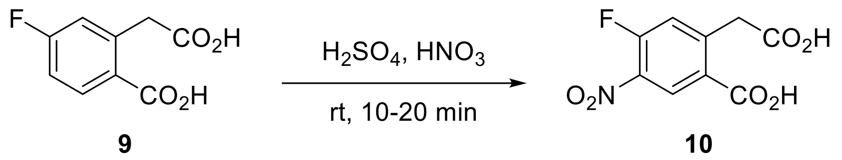

To a 100 mL round bottom flask equipped with a stir bar was added **fluoro-HPA 9** (0.5 g, 2.52 mmol). A 50% (v/v) solution of concentrated H_2SO_4 and HNO_3 was made in a 10 mL graduated cylinder cooled *via* ice bath. The acid solution was added to the flask and stirred at room temperature until the starting material was completely dissolved (ca. 10-20 min). The solution was then poured onto ice and extracted with EtOAc. The combined organic extracts were then washed with brine, dried over Na_2SO_4, and concentrated under reduced pressure to afford **fluoro-nitro-HPA 10** (0.61 g, quant.) as a pale-yellow powder. The crude material was carried forward without further purification.

Fluoro-nitro-HPA 10

^{1}H NMR (400 MHz, CD_3OD) δ 8.74-8.72 (d, 1H, *J* = 7.86 Hz), 7.47-7.45 (d, 1H, *J* = 11.73 Hz) 4.16 (s, 2H) ppm; **^{13}C{1H} NMR** (150 MHz, CD_3OD) δ 173.7, 167.2, 158.8, 157.0, 147.5, 130.2, 128.7, 123.5, 116.8, 40.8 ppm; **M.P.** 198-200 °C; **HRMS (ESI-TOF)** m/z: $[M-H]^-$ Calcd for $C_9H_5FNO_6$ 242.0101; Found 242.0105.

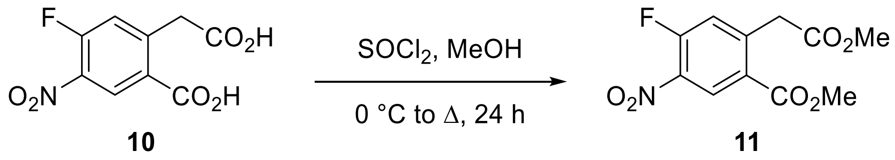

In a 250 mL round bottom flask equipped with a stir bar was added **fluoro-nitro-HPA 10** (0.5 g, 2.06 mmol) and MeOH (20 mL). The solution was cooled to 0 °C *via* salted ice bath. $SOCl_2$ (0.3 mL, 4.32 mmol, 2.1 equiv.) was added dropwise and the solution was heated to reflux *via* silicone oil bath and allowed to stir for 24 h. The solution was then allowed to cool to room temperature, concentrated under reduced pressure and the resulting residue was dissolved in EtOAc and washed with brine. The organic extracts were combined, dried over Na_2SO_4, and concentrated under reduced pressure. The crude product was purified by flash column chromatography (SiO_2, 1:4 EtOAc/Hexanes, R_f = 0.23) to afford **fluoro-nitro-HPE 11** (0.41 g, 74%) as a pale-yellow oil.

Fluoro-nitro-HPE 11

^{1}H NMR (400 MHz, CD_3OD) δ 8.74-8.72 (d, 1H, *J* = 7.76 Hz), 7.53-7.50 (d, 1H, *J* = 11.76 Hz), 4.17 (s, 2H), 3.93 (s, 3H), 3.71 (s, 3H) ppm; **^{13}C{1H} NMR** (100 MHz, CD_3OD) δ 172.3, 166.2, 159.4,

146.8, 130.0, 128.0, 123.7, 117.4, 53.1, 52.6, 40.6 ppm; **HRMS (ESI-TOF)** m/z: $[M+Na]^+$ Calcd for $C_{11}H_{10}FNO_6Na$ 294.0390; Found 294.0393.

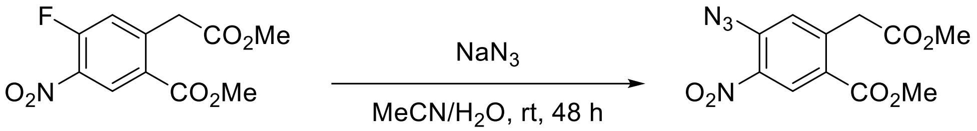

11 12

To a 20 mL scintillation vial equipped with a stir bar was added a solution of **fluoro-nitro-HPE 11** (0.21 g, 0.77 mmol) in MeCN (8 mL). H_2O (2 mL) was then added, followed by NaN_3 (0.2 g, 2.08 mmol, 4 equiv.). The resulting yellow suspension was allowed to stir at room temperature for 48 h or until the suspension turned into a red solution. The solution was then diluted with 1 M HCl and extracted with EtOAc. The organic extracts were combined and washed with brine, dried over Na_2SO_4, and concentrated under reduced pressure. The crude product was purified by flash column chromatography (SiO_2, 1:4 EtOAc/Hexanes, R_f = 0.20) to afford **nitro-azide HPE 12** (0.27 g, 91%) as a yellow-orange solid.

Nitro-azide HPE 12
^{1}H NMR (400 MHz, CD_3OD) δ 8.54 (s, 1H), 7.50 (s, 1H), 4.15 (s, 2H), 3.88 (s, 3H), 3.69 (s, 3H) ppm; **^{13}C{1H} NMR** (100 MHz, $CDCl_3$) δ 170.4, 164.8, 142.9, 138.2, 133.6, 129.4, 126.1, 124.5, 52.7, 52.4, 40.2 ppm; **M.P.** 200 °C (decomposition); **HRMS (ESI-TOF)** m/z: $[M+Na]^+$ Calcd for $C_{11}H_{10}N_4O_6Na$ 317.0498; Found 317.0494.

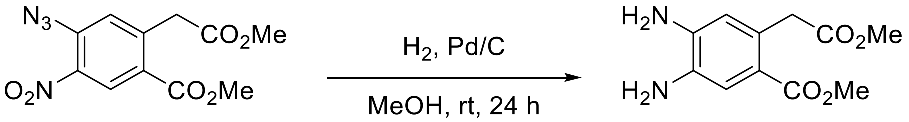

12 13

To a flame dried 3-neck 100 mL round bottom flask equipped with a stir bar under $Ar_{(g)}$ atmosphere was added 5% palladium on carbon (0.02 g), followed by a solution of **nitro-azide HPE 12** (0.21 g, 0.7 mmol) in MeOH (20mL). The $Ar_{(g)}$ atmosphere was then purged 3× with $H_{2(g)}$ *via* balloon and the suspension was allowed to stir at room temperature for 24 h. The suspension was then vacuum filtered through a pad of Celite, adsorbed onto silica under reduced pressure, and purified by flash column chromatography (SiO_2, 3:2 EtOAc/Hexanes, R_f = 0.24) to afford **dimethyl ester 13** (0.165 g, quant.) as a brown-orange powder.

Dimethyl ester 13
^{1}H NMR (400 MHz, CD_3OD) δ 7.43 (s, 1H), 6.52 (s, 1H), 3.82 (s, 2H), 3.77 (s, 3H), 3.67 (s, 3H) ppm; **^{13}C{1H} NMR** (100 MHz, CD_3OD) δ 175.0, 169.3, 142.0, 133.2, 130.3, 120.3, 119.4, 118.7, 52.2,

51.7, 41.1 ppm; **M.P.** 190-195 °C; **HRMS (ESI-TOF)** m/z: $[M+Na]^+$ Calcd for $C_{11}H_{14}N_2O_4Na$ 261.0851; Found 261.0846.

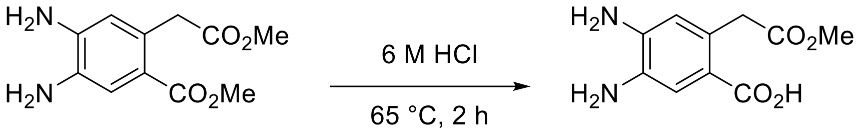

13 **14**

To a 50 mL round bottom flask equipped with a stir bar was added **dimethyl ester 13** (0.05 g, 0.21 mmol) and 6 M HCl (5 mL). The solution was heated to 65 °C *via* silicone oil bath and was allowed to stir for 2 h. The reaction was then cooled to room temperature and neutralized to pH 7 with NaOH. The solution was then triturated with EtOH and vacuum filtered. The solvent was removed under reduced pressure and the process was repeated until no salts remained to afford **methyl ester 14** (0.047 g, quant.) as a brown powder.

Methyl ester 14

^{1}H NMR (400 MHz, CD_3OD) δ 7.37 (s, 1H), 6.54 (s, 1H), 3.77 (s, 3H), 3.70 (s, 2H) ppm; **^{13}C{1H} NMR** (150 MHz, D_2O) δ 181.0, 170.1, 140.0, 132.8, 131.3, 120.2, 119.8, 119.6, 52.0, 43.4 ppm; **M.P.** 205 °C (decomposition); **HRMS (ESI-TOF)** m/z: $[M+Na]^+$ Calcd for $C_{10}H_{12}N_2O_4Na$ 247.0695; Found 247.0694.

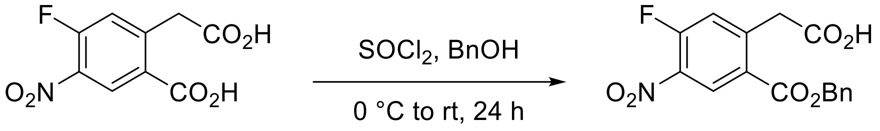

10 **15**

To a 100 mL round bottom flask equipped with a stir bar was added **fluoro-nitro-HPA 10** (0.613 g, 2.52 mmol) and BnOH (5 mL). The solution was cooled to 0 °C *via* salted ice bath and $SOCl_2$ (0.4 mL, 5.54 mmol, 2.2 equiv.) was added dropwise. The solution was allowed to warm to room temperature and stir for 24 h. The solution was diluted with EtOAc, washed with brine, and dried over Na_2SO_4. The organic extracts were then concentrated under reduced pressure and triturated with hexanes 3× to remove residual BnOH. The resulting crude product was then adsorbed onto silica gel under reduced pressure and purified twice by flash column chromatography (SiO_2, 1:4 EtOAc/Hexanes, R_f = 0; then 100% MeOH; SiO_2, 1:19 MeOH/CH_2Cl_2, R_f = 0.33) to afford the monoprotected **benzyl ester 15** (0.461 g, 55%) as a pale-orange solid.

Benzyl ester 15

^{1}H NMR (500 MHz, $(CD_3)_2SO$) δ 8.61-8.59 (d, 1H, *J* = 7.81 Hz), 7.70-7.68 (d, 1H, *J* = 12.02 Hz), 7.36-7.32 (m, 5H), 5.10 (s, 2H), 4.23 (s, 2H) ppm; **^{13}C{1H} NMR** (150 MHz, CD_3OD) δ 169.9, 165.9, 156.6,

154.8, 145.4, 136.0, 135.3, 128.6, 128.5, 128.1, 127.9, 122.4, 65.9, 39.4 ppm; **M.P.** 190 °C (decomposition); **HRMS (ESI-TOF)** m/z: $[M+Na]^+$ Calcd for $C_{16}H_{12}FNO_6Na$ 356.0546; Found 356.0541.

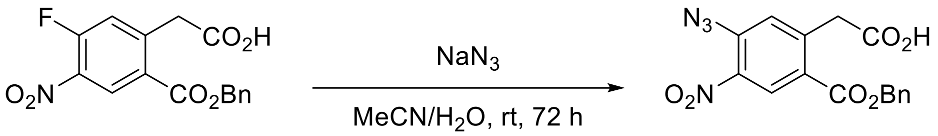

15 **16**

To a 100 mL round bottom flask equipped with a stir bar was added a solution of **benzyl ester 15** (0.46 g, 1.38 mmol) in MeCN (16 mL). H_2O (4 mL) was added, followed by NaN_3 (0.45 g, 6.92 mmol, 5 equiv.). The resulting yellow suspension was allowed to stir at room temperature for 72 h or until the suspension turned into a red solution. The solution was then diluted with 1 M HCl and extracted with EtOAc. The organic extracts were combined and washed with brine, dried over Na_2SO_4, and concentrated under reduced pressure. The crude product was purified by flash column chromatography (SiO_2, 1:9 $MeOH/CH_2Cl_2$, $R_f = 0.35$) to afford **nitro-azide 16** (0.422 g, 86%) as a pale-yellow solid.

Nitro-azide 16
^{1}**H NMR** (500 MHz, CD_3OD) δ 8.55 (s, 1H), 7.45 (s, 1H), 7.33-7.28 (m, 5H), 5.13 (s, 2H), 4.21 (s, 2H) ppm; ^{13}**C{1H} NMR** (150 MHz, CD_3OD) δ 171.7, 167.2, 158.8, 157.0, 146.9, 146.8, 137.3, 137.1, 137.0, 130.2, 129.5, 129.2, 129.1, 123.6, 123.4, 67.8, 41.0 ppm; **M.P.** 190 °C (decomposition); **HRMS (ESI-TOF)** m/z: $[M-H]^-$ Calcd for $C_{16}H_{11}N_4O_6$ 355.0679; Found 355.0668.

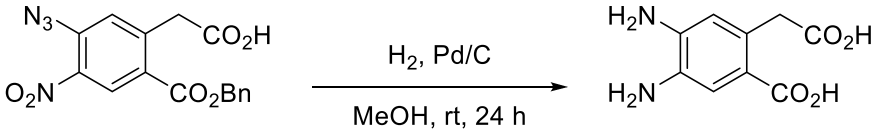

16 **2**

To a flame dried 3-neck 100 mL round bottom flask equipped with a stir bar under $Ar_{(g)}$ atmosphere was added 5% palladium on carbon (0.02 g), followed by a solution of **nitro-azide 16** (0.2 g, 0.7 mmol) in MeOH (20 mL). The $Ar_{(g)}$ atmosphere was then purged 3× with $H_{2(g)}$ *via* balloon and the suspension was allowed to stir at room temperature for 24 h. The suspension was then vacuum filtered through a pad of Celite, adsorbed onto silica under reduced pressure, and purified by flash column chromatography (SiO_2, 3:7 $MeOH/CH_2Cl_2$, $R_f = 0$; then 100% MeOH) to yield **diacid 2** (0.1 g, 85%) as a yellow-brown solid.

Diacid 2

^{1}**H NMR** (500 MHz, D_2O) δ 7.30 (s, 1H), 6.67 (s, 1H), 3.68 (s, 2H) ppm; ^{13}**C{1H} NMR** (150 MHz, D_2O) δ 179.2, 174.0, 138.8, 130.3, 129.1, 124.6, 120.9, 119.3, 41.6 ppm; **M.P.** 180 °C (decomposition); **HRMS (ESI-TOF)** m/z: $[M+Na]^+$ Calcd for $C_9H_{10}N_2O_4Na$ 233.0538; Found 233.0536.

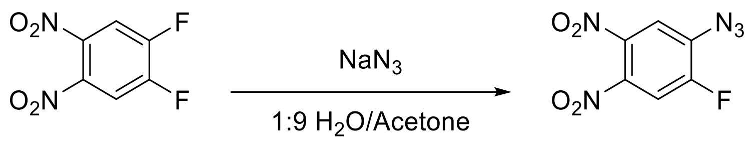

17 **18a**

To a 20 mL scintillation vial equipped with a stir bar was added **1,2-dinitro-4,5-difluorobenzene 17** (0.2 g, 1 mmol) and 10% H_2O in acetone (2.5 mL). NaN_3 (0.098 g, 1.5 mmol) was then added, and the reaction was allowed to stir at room temperature for 14 h. The aqueous layer was extracted 3× with diethyl ether and the organic layer were combined and back extracted with brine. The organic layer was then dried over Na_2SO_4, filtered through cotton, and concentrated under reduced pressure. The crude product was then adsorbed onto silica and purified by flash column chromatography (SiO_2, 5% Et_2O/hexanes) to afford **18a** (0.10 g, 45%) as a light yellow oil.

18a
^{1}**H NMR** (400 MHz, $CDCl_3$) δ 7.78 (d, 1H, *J* = 9.5 Hz), 7.54 (m, 1H) ppm; ^{13}**C{1H} NMR** (100 MHz, $CDCl_3$) δ 156.8, 154.2, 140.7, 138.8, 135.1, 135.0, 118.2, 118.1, 115.1, 114.8 ppm.

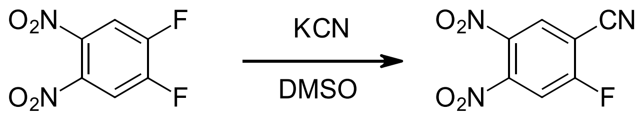

17 **18b**

To a 20 mL scintillation vial equipped with a stir bar was added **1,2-dinitro-4,5-difluorobenzene 17** (0.2 g, 1 mmol) and DMSO (2.5 mL). KCN (0.95 g, 1.5 mmol) was then added, and the reaction was allowed to stir at 65 °C (sand bath) for 14 h. The reaction was then cooled to room temperature and diluted with H_2O and the aqueous layer was extracted 3× with EtOAc. The organic extracts were then dried over Na_2SO_4, filtered through cotton, and concentrated under reduced pressure. The crude product was loaded onto silica with CH_2Cl_2 and purified by flash column chromatography (SiO_2, 50% Et_2O/hexanes) to afford **18b** (0.11 g, 51%), a light brown solid.

18b
^{1}**H NMR** (400 MHz, CD_3OD) δ 8.01 (d, 1H, *J* = 10.1 Hz), 7.44 (d, 1H, *J* = 7.3 Hz) ppm; ^{13}**C{1H} NMR** (100 MHz, CD_3OD) δ 152.7, 151.2, 151.0, 150.2, 141.3, 133.1 ppm; **M.P.** 116-120 °C.

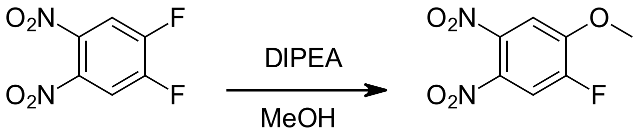

17 **18c**

To a 20 mL scintillation vial equipped with a stir bar **1,2-dinitro-4,5-difluorobenzene 17** (0.2 g, 1 mmol), DIPEA (0.174 mL, 1 mmol), and MeOH (5 mL) were added. The reaction was allowed to stir at 60 °C (sand bath) for 14 h. The reaction was then concentrated under reduced pressure and the resulting residue was taken up with diethyl ether. The ether was washed 2× with 1 M HCl and once with brine. The organic layer was then dried over Na_2SO_4, filtered through cotton, and concentrated under reduced pressure to afford **18c** (0.215 g, quant.) as a faint brown paste.

18c
^{1}H NMR (400 MHz, $(CD_3)_2SO$) δ 8.29 (d, 1H, *J* = 10.5 Hz), 8.02 (d, 1H, *J* = 7.3 Hz), 4.04 (d, 3H, *J* = 1.5 Hz) ppm; **^{13}C{1H} NMR** (100 MHz, $(CD_3)_2SO$) δ 153.2, 152.6, 152.5, 150.6, 141.0, 133.8, 133.7, 114.6, 114.3, 110.9, 110.8, 58.4 ppm.

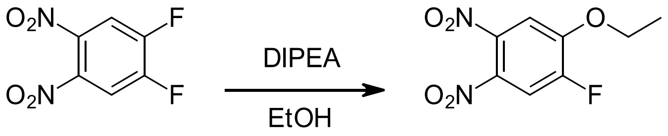

17 **18d**

To a 20 mL scintillation vial equipped with a stir bar was added **1,2-dinitro-4,5-difluorobenzene 17** (0.2 g, 1 mmol), DIPEA (0.174 mL, 1 mmol), and EtOH (5 mL). The reaction was allowed to stir at 60 °C (sand bath) for 14 h. The reaction was then cooled to room temperature and concentrated under reduced pressure, and the resulting residue was taken up with diethyl ether. The ether was washed 2× with 1 M HCl and once with brine. The organic layer was then dried over Na_2SO_4, filtered through cotton, and concentrated under reduced pressure to afford **18d** (0.225 g, 98%) as a yellow solid.

18d
^{1}H NMR (400 MHz, $CDCl_3$) δ 7.80 (d, 1H, *J* = 9.6 Hz), 7.37 (d, 1H, *J* = 6.9 Hz), 4.28 (q, 2H, *J* = 7.0 Hz), 1.59 (t, 3H, *J* = 6.8 Hz) ppm; **^{13}C{1H} NMR** (100 MHz, $CDCl_3$) δ 153.7, 151.3, 151.2, 151.1, 113.9, 113.6, 109.8, 109.8, 66.7, 14.3 ppm; **M.P.** 66-67 °C.

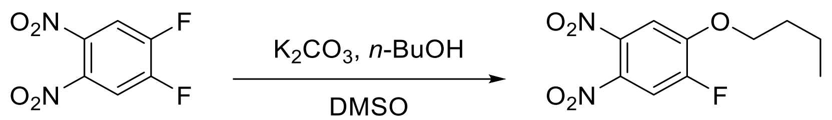

17 **18e**

To a 20 mL scintillation vial equipped with a stir bar was added **1,2-dinitro-4,5-difluorobenzene 17** (0.2 g, 1 mmol), DMSO (5 mL), and *n*-butanol (0.182 mL, 2 mmol). K_2CO_3 (0.138 g, 1 mmol) was then added, and the reaction was allowed to stir at 60 °C (sand bath) for 14 h. The reaction was then cooled to room temperature and diluted with H_2O and the aqueous layer was extracted 3× with diethyl ether. The ether extracts were combined and washed 2× with 1 M K_2CO_3 and once with H_2O. The organic layer was then dried over Na_2SO_4, filtered through cotton, and concentrated under reduced pressure to afford **18e** (0.186 g, 78%) as a brown solid.

18e
^{1}H NMR (400 MHz, $CDCl_3$) δ 7.79 (dd, 1H, *J* = 9.6, 1.5 Hz), 7.38 (dd, 1H, *J* = 7.0, 1.4 Hz), 4.20 (td, 2H, *J* = 6.5, 1.5 Hz), 1.90 (quin, 2H, *J* = 6.8 Hz), 1.63-1.49 (m, 2H), 1.02 (td, 3H, *J* = 7.4, 1.5 Hz) ppm; **^{13}C{1H} NMR** (100 MHz, $CDCl_3$) δ 153.7, 151.6, 151.1, 113.8, 113.6, 109.8, 70.7, 30.6, 19.0, 13.7 ppm; **M.P.** 37-39 °C.

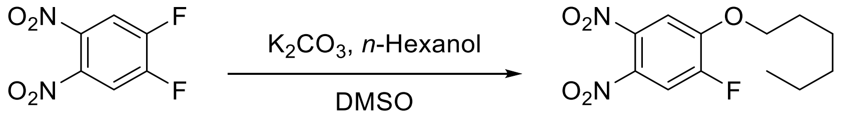

17 **18f**

To a 20 ml scintillation vial equipped with a stir bar was added **1,2-dinitro-4,5-difluorobenzene 17** (0.042 g, 0.2 mmol), K_2CO_3 (0.138 g, 1 mmol), and DMSO (0.5 mL). *n*-Hexanol (0.04 mL, 0.5 mmol) was then added, and the reaction was allowed to stir at 60 °C (sand bath) for 14 h. The reaction was then cooled to room temperature and diluted with H_2O and the aqueous layer was extracted 3× with diethyl ether. The ether extracts were combined and washed 2× with 1 M H_2O and once with brine. The organic layer was then dried over Na_2SO_4 and filtered through cotton. The resulting crude was purified by flash chromatography (SiO_2, Et_2O/Hexanes) to a yield **18f** (0.034 g, 60%) as a viscous orange oil.

18f
^{1}H NMR (400 MHz, $CDCl_3$) δ 7.79 (d, 1H, *J* = 9.6 Hz), 7.37 (d, 1H, *J* = 7.0 Hz), 4.19 (t, 2H, *J* = 6.5 Hz), 1.97-1.85 (m, 2H), 1.51 (t, 2H, *J* = 7.5 Hz), 1.38 (m, 4H, *J* = 3.9 Hz), 0.98-0.88 (m, 3H) ppm; **^{13}C NMR** (100 MHz, $CDCl_3$) δ 153.7, 151.6, 151.5, 151.2, 140.7, 134.5, 113.8, 113.6, 109.8, 109.8, 71.0, 31.3, 28.6, 25.4, 22.5, 13.9 ppm.

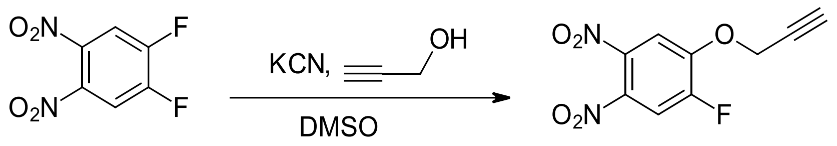

17

18g

To a 20 mL scintillation vial equipped with a stir bar was added **1,2-dinitro-4,5-difluorobenzene 17** (0.2 g, 1 mmol), KCN (0.065 g, 1 mmol), and DMSO (4 mL). Propargyl alcohol (1 mL, 17.3 mmol) was then added, and the reaction was allowed to stir at 60 °C (sand bath) for 14 h. The reaction was then cooled to room temperature and diluted with H_2O and the aqueous layer was extracted 3× with diethyl ether. The ether extracts were combined and washed 2× with 1 M K_2CO_3 and once with H_2O. The organic layer was then dried over Na_2SO_4, filtered through cotton, and concentrated under reduced pressure to afford **18g** (0.194 g, 83%) as a brown oil.

18g

^{1}H NMR (400 MHz, $CDCl_3$) δ 7.80 (d, 1H, *J* = 10.1 Hz), 7.62 (d, 1H, *J* = 10.1 Hz), 4.97 (s, 2H), 2.73 (s, 1H) ppm; **^{13}C{1H} NMR** (100 MHz, $CDCl_3$) δ 154.2, 151.6, 149.4, 149.3, 114.1, 113.9, 111.3, 78.9, 75.4, 58.0 ppm.

Note: 1 equivalent of Hunig's base was a suitable substitute for 1 equivalent of KCN.

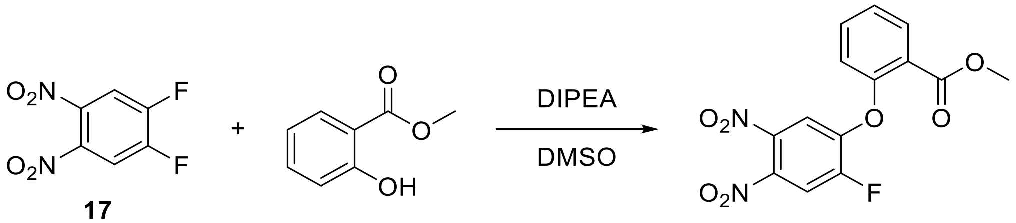

17

18h

To a 20 mL scintillation vial equipped with a stir bar was added **1,2-dinitro-4,5-difluorobenzene 17** (1.02 g, 5 mmol) and methyl salicylate (0.66 mL, 5.1 mmol). DMSO (10 mL) and DIPEA (1 mL, 5.77 mmol) were then added, and the reaction was allowed to stir at 60 °C (sand bath) for 24 h. The reaction was then cooled to room temperature and diluted with H_2O and the aqueous layer was extracted 3× with diethyl ether. The ether extracts were then combined and washed 2× with brine. The organic layer was then dried over Na_2SO_4, filtered through cotton, and concentrated under reduced pressure. The crude product was then adsorbed onto silica and purified by flash column chromatography (SiO_2, EtOAc/hexanes) to yield **18h** (0.63 g, 37%) as a yellow solid.

18h

^{1}H NMR (400 MHz, $CDCl_3$) δ 8.13 (dd, 1H, *J* = 8.0, 2.0 Hz), 7.88 (dd, 1H, *J* = 9.3, 1.9 Hz), 7.72 (td, 1H, *J* = 7.8, 1.8 Hz), 7.53-7.45 (m, 1H), 7.28-7.23 (m, 1H), 7.02 (dd, 1H, *J* = 7.0, 1.8 Hz), 3.88 (s, 3H)

ppm; **$^{13}C\{1H\}$ NMR** (100 MHz, $CDCl_3$) δ 164.2, 153.7, 152.2, 151.1, 151.0, 150.9, 140.2, 136.1, 135.1, 133.0, 127.4, 123.5, 123.1, 114.6, 114.4, 112.4, 112.4, 52.6 ppm; **M.P.** 98-100 °C; **HRMS (ESI-TOF)** m/z: $[M+Na]^+$ Calcd for $C_{14}H_9FN_2O_7Na$ 337.0466; Found 337.0466.

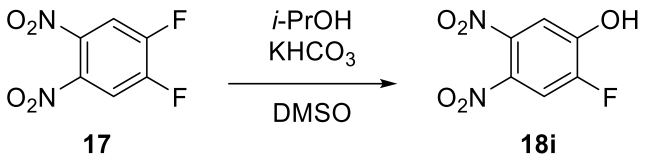

To a 20 mL scintillation vial equipped with a stir bar was added **1,2-dinitro-4,5-difluorobenzene 17** (0.204 g, 1 mmol), $KHCO_3$ (0.100 g, 1 mmol), and DMSO (5 mL). Isopropanol (0.1 mL, 1.31 mmol) was then added, and the reaction was allowed to stir at 60 °C (sand bath) for 14 h. The reaction was then cooled to room temperature and diluted with H_2O and the aqueous layer was extracted 2× with diethyl ether. The remaining aqueous solution was extracted 3× with EtOAc. The extracts were combined, dried with Na_2SO_4, and filtered through cotton. Excess solvent was evaporated by vacuum. CH_2Cl_2 was added to the resulting orange slurry which resulted in the formation of a precipitate, which was washed with solvent to yield **18i** (0.074 g, 37% yield) as a yellow powder.

18i

^1H NMR (400 MHz, $(CD_3)_2CO$) δ 7.62 (d, 1H, *J* = 12.2 Hz), 6.35 (d, 1H, *J* = 7.8 Hz); **$^{13}C\{1H\}$ NMR** (100 MHz, $(CD_3)_2CO$) δ 114.7, 114.6, 111.5, 111.3 ppm; **M.P.** 215-218 °C (decomposition).

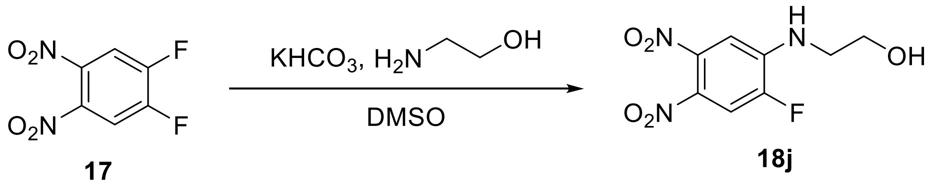

To a 20 mL scintillation vial equipped with a stir bar was added **1,2-dinitro-4,5-difluorobenzene 17** (0.2 g, 1 mmol) and DMSO (5 mL). Ethanolamine (0.121 mL, 2 mmol) and $KHCO_3$ (0.3 g, 3 mmol) were then added, and the reaction was allowed to stir at 30 °C for 4 h. The reaction was then cooled to room temperature and diluted with H_2O, during which a precipitate formed. The mixture was vacuum filtered and the solid was allowed to dry on the filter paper under vacuum for approximately 2 h to afford **18j** (0.154 g, 63%) as a yellow solid.

18j

^1H NMR (400 MHz, $(CD_3)_2SO$) δ 8.04 (d, 1H, *J* = 11.6 Hz), 7.68 (bs, 1H, N**H**), 7.41 (d, 1H, *J* = 7.1 Hz), 4.83 (bs, 1H, O**H**), 3.57 (t, 2H, *J* = 6.3 Hz), 3.35 (t, 2H, *J* = 6.4 Hz) ppm; **$^{13}C\{1H\}$ NMR** (100 MHz,

$(CD_3)_2SO$) δ 149.6, 147.2, 144.5, 144.3, 144.2, 125.3, 125.2, 113.0, 112.7, 106.2, 106.2, 59.9, 45.6 ppm; **M.P.** 144-147 °C; **HRMS (ESI-TOF)** m/z: $[M-H]^-$ Calcd for $C_8H_7FN_3O_5$ 244.0375; Found 244.0371.

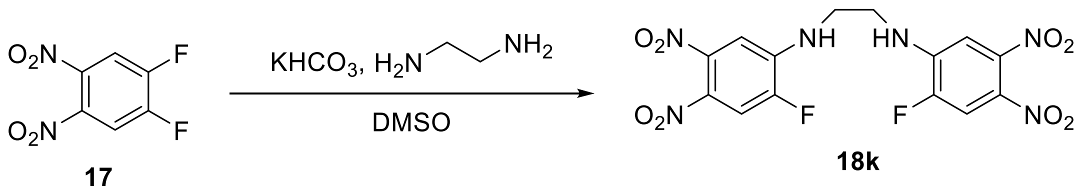

17 → **18k**

To a 20 mL scintillation vial equipped with a stir bar was added **1,2-dinitro-4,5-difluorobenzene 17** (0.4 g, 2 mmol) and DMSO (1 mL). Ethylenediamine (0.066 mL, 1 mmol) and $KHCO_3$ (0.2 g, 2 mmol) were then added, and the reaction was allowed to stir at 30 °C for 4 h. The reaction was then cooled to room temperature and diluted with H_2O, during which a precipitate formed. The mixture was vacuum filtered and the solid was allowed to dry on the filter paper under vacuum for approximately 2 h to afford **18k** (0.317 g, 74%) as a yellow powder.

18k

^{1}H NMR (400 MHz, $(CD_3)_2SO$) δ 8.08 (dd, 2H, *J* = 17.1, 11.5 Hz), 7.43 (d, 2H, *J* = 7.4 Hz), 3.53 (s, 4H) ppm; **^{13}C{1H} NMR** (100 MHz, $(CD_3)_2SO$) δ 149.9, 147.4, 144.0, 144.0, 143.9, 143.7, 125.9, 125.8, 113.1, 112.8, 106.1, 106.1 ppm; **M.P.** 234-38 °C (decomposition); **HRMS (ESI-TOF)** m/z: $[M-H]^-$ Calcd for $C_{14}H_9F_2N_2O_8$ 427.0455; Found 427.0451.

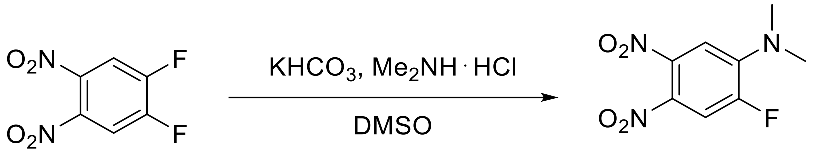

17 → **18l**

To a 20 mL scintillation vial equipped with a stir bar was added **1,2-dinitro-4,5-difluorobenzene 17** (0.2 g, 1 mmol) and DMSO (5 mL). Dimethylamine HCl (0.163 mL, 2 mmol) and $KHCO_3$ (0.3 g, 3 mmol) were then added, and the reaction was allowed to stir at 30 °C for 24 h. The reaction was then cooled to room temperature and diluted with H_2O, during which a precipitate formed. The mixture was vacuum filtered and the solid was allowed to dry on the filter paper under vacuum for approximately 2 h to afford **18l** (0.148 g, 64%) as an orange powder.

18l

^{1}**H NMR** (400 MHz, $(CD_3)_2SO$) δ 8.04 (dd, 1H, *J* = 14.4, 1.5 Hz), 7.38 (d, 1H, *J* = 7.9 Hz), 3.13-3.07 (m, 6H) ppm; ^{13}**C{1H} NMR** (100 MHz, $(CD_3)_2SO$) δ 154.2, 151.6, 149.4, 149.3, 140.1, 135.8, 40.0 ppm; **M.P.** 124-126 °C; **HRMS (ESI-TOF)** m/z: $[M+H]^+$ Calcd for $C_8H_9FN_3O_4$ 230.072; Found 230.068.

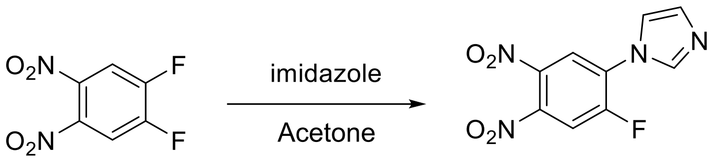

17 **18m**

To a 20 mL scintillation vial equipped with a stir bar was added **1,2-dinitro-4,5-difluorobenzene 17** (0.20 g, 1 mmol) and acetone (5 mL). Imidazole (0.068 g, 1 mmol) was then added, and the reaction was allowed to stir at 50 °C (sand bath) for 14 h. The reaction was evaporated onto silica gel. The solid mixture was eluted through a silica gel chromatography column (5-50% EtOAc/Hex). The second UV active fractions were collected, and the resulting solution was condensed under vacuum to yield **18m** (0.068 g, 27%) as a red-orange solid.

18m
^{1}**H NMR** (400 MHz, $(CD_3)_2SO$) δ 8.71 (d, 1H, *J* = 6.7 Hz), 8.66 (d, 1H, *J* = 10.2 Hz), 8.26 (s, 1H), 7.78 (d, 1H, *J* = 2.2 Hz), 7.21 (s, 1H) ppm; ^{13}**C{1H} NMR** (100 MHz, $(CD_3)_2SO$) δ 156.6, 154.0, 140.5, 139.4, 138.1, 138.1, 130.6, 122.6, 122.6, 120.4, 120.3, 116.5, 116.2 ppm; **M.P.** 119-123 °C.

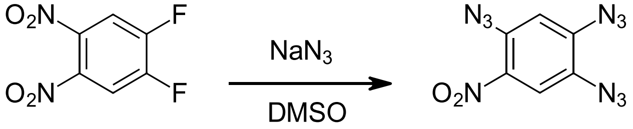

17 **19a**

To a 20 mL scintillation vial equipped with a stir bar was added **1,2-dinitro-4,5-difluorobenzene 17** (0.2 g, 1 mmol) and DMSO (2.5 mL). NaN_3 (0.195 g, 3 mmol) was then added, and the reaction was allowed to stir at 30 °C (sand bath) for 14 h. The reaction was then cooled to room temperature and diluted with H_2O and the aqueous layer was extracted 3× with diethyl ether. The ether extracts were combined and washed 2× with 1 M K_2CO_3 and once with H_2O. The organic layer was then dried over Na_2SO_4, filtered through cotton, and concentrated under reduced pressure to yield **19a**. Characterization was in accordance with a previous report.[S2]

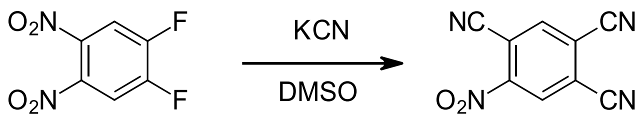

17 **19b**

To a 20 mL scintillation vial equipped with a stir bar was added **1,2-dinitro-4,5-difluorobenzene 17** (0.2 g, 1 mmol), KCN (0.195 g, 3 mmol), and DMSO (2.5 mL), and the reaction was allowed to stir at 60°C (sand bath) for 14 h. The reaction was then cooled to room temperature and diluted with brine, and the aqueous layer was extracted 2× with diethyl ether. The aqueous layer was extracted 3× with EtOAc. The EtOAc extracts were then dried over Na_2SO_4, filtered through cotton, and concentrated under reduced pressure. The crude product was then adsorbed onto silica and purified by flash column chromatography (SiO_2, 50% Et_2O/hexanes) to afford **19b** as a beige paste (0.02 g, 10%).

19b
^{1}H NMR (400 MHz, $(CD_3)_2CO$) δ 8.69 (d, 1H, *J* = 2.4 Hz), 7.64 (s, 1H) ppm; **^{13}C{1H} NMR** (100 MHz, $(CD_3)_2CO$) δ 165.1, 147.5, 132.9, 132.4, 131.6, 126.3, 124.4, 113.2, 112.5, 103.4 ppm.

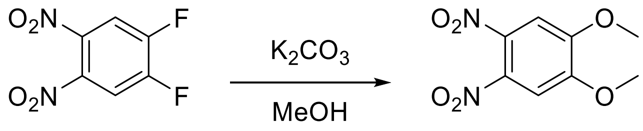

17 **19c**

To a 20 mL scintillation vial equipped with a stir bar was added **1,2-dinitro-4,5-difluorobenzene 17** (0.2 g, 1 mmol), K_2CO_3 (0.276, 2 mmol), and MeOH (5 mL). The reaction was allowed to stir at 60 °C (sand bath) for 14 h. The reaction was then cooled to room temperature and concentrated under reduced pressure, and the resulting residue was taken up with diethyl ether. The ether was washed 3× with water and once with brine. The organic layer was then dried over Na_2SO_4, filtered through cotton, and concentrated under reduced pressure to afford **19c** (0.205 g, 90%) as an orange solid. Characterization was in accordance with a previous report.[S3]

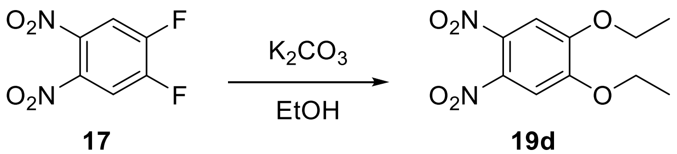

17 **19d**

To a 20 mL scintillation vial equipped with a stir bar was added **1,2-dinitro-4,5-difluorobenzene 17** (0.2 g, 1 mmol), K_2CO_3 (0.276 g, 2 mmol), and EtOH (5 mL). The reaction was allowed to stir at 60 °C (sand bath) for 14 h. The reaction was then cooled to room temperature and concentrated

under reduced pressure, and the resulting residue was taken up with diethyl ether. The ether was washed 3× with water and once with brine. The organic layer was then dried over Na_2SO_4, filtered through cotton, and concentrated under reduced pressure to afford **19d** (0.212 g, 83%) as an orange solid. Characterization was in accordance with a previous report.[S4]

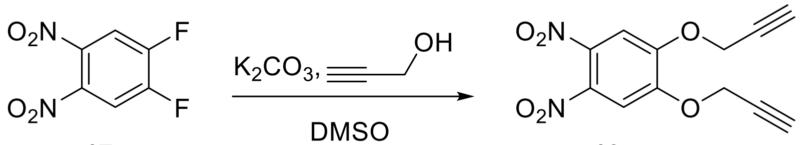

17 **19e**

To a 20 mL scintillation vial equipped with a stir bar was added **1,2-dinitro-4,5-difluorobenzene 17** (0.2 g, 1 mmol), K_2CO_3 (0.414 g, 3 mmol), and DMSO (5 mL). Propargyl alcohol (0.035 mL, 6 mmol) was then added, and the reaction was allowed to stir at 60 °C for 14 h. The reaction was then cooled to room temperature and diluted with H_2O and the aqueous layer was extracted 3× with diethyl ether. The ether extracts were combined and washed 2× with 1 M K_2CO_3 and once with H_2O. The organic layer was then dried over Na_2SO_4, filtered through cotton, and concentrated under reduced pressure. The resulting residue was then washed 3× with hexanes and once with MeOH to afford **19e** (0.253 g, 92%) as a gray solid.

19e
^{1}H NMR (400 MHz, $CDCl_3$) δ 7.58 (s, 2H), 4.92 (s, 4H), 2.69 (s, 2H) ppm; **^{13}C{1H} NMR** (100 MHz, $CDCl_3$) δ 150.0, 137.1, 109.7, 78.4, 75.9, 57.6 ppm; **M.P.** 140-143 °C.

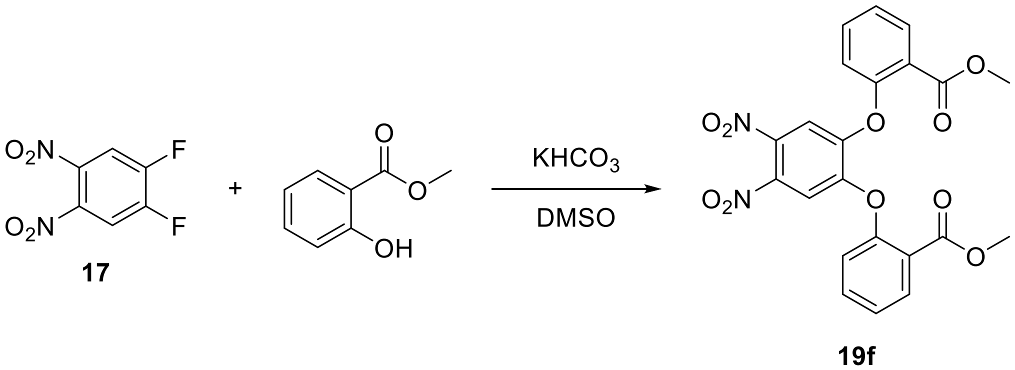

17 **19f**

To a 20 mL scintillation vial equipped with a stir bar was added **1,2-dinitro-4,5-difluorobenzene 17** (0.2 g, 1 mmol), $KHCO_3$ (0.300 g, 3 mmol), and DMSO (5 mL). Methyl salicylate (0.608 g, 4 mmol) was then added, and the reaction was allowed to stir at room temperature for 14 h. The reaction was then diluted with H_2O and the aqueous layer was extracted 3× with diethyl ether. The ether extracts were then combined and washed 2× with brine. The organic extracts were

then dried over Na_2SO_4, filtered through cotton, and concentrated under reduced pressure. The resulting solid was wet loaded onto a silica gel column with CH_2Cl_2 and purified (20-50% Et_2O in Hexanes) to afford **19f** (0.304 g, 65%) as a white powder.

19f
^{1}H NMR (400 MHz, $(CD_3)_2CO$) δ 8.05 (dt, 2H, *J* = 8.1, 1.7 Hz), 7.82-7.73 (m, 2H), 7.56-7.36 (m, 6H), 3.84 (s, 6H) ppm; **^{13}C{1H} NMR** (100 MHz, $(CD_3)_2CO$) δ 164.6, 153.4, 151.2, 137.8, 134.7, 134.7, 132.2, 132.2, 126.2, 126.2, 123.8, 122.4, 122.4, 113.7, 113.7, 51.8, 51.8 ppm; **M.P.** 100-103 °C; **HRMS (ESI-TOF)** m/z: $[M+Na]^+$ Calcd for $C_{22}H_{16}N_2O_{10}Na$ 491.0696; Found 491.0692.

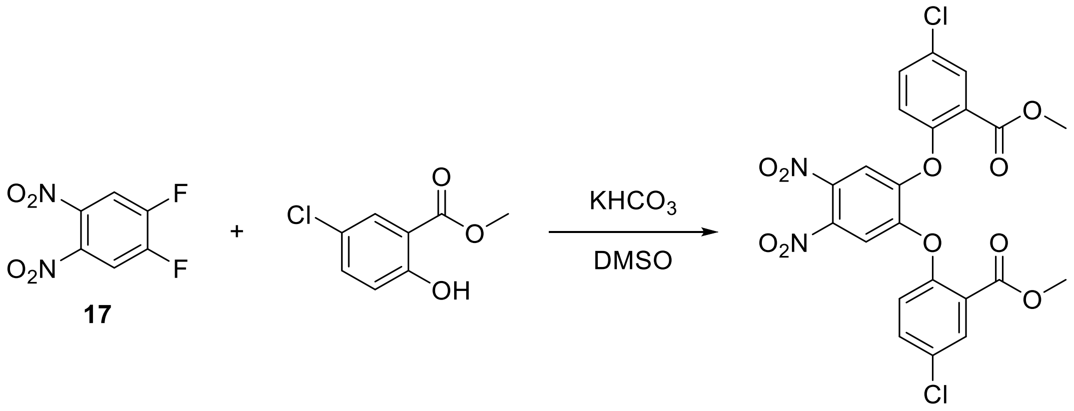

19g

To a 20 mL scintillation vial equipped with a stir bar was added **1,2-dinitro-4,5-difluorobenzene 17** (0.2 g, 1 mmol), $KHCO_3$ (0.138 g, 4 mmol), and DMSO (5 mL). Methyl 5-chlorosalicylate (0.466g, 2.5 mmol) was then added, and the reaction was allowed to stir at room temperature for 14 h. The reaction was then diluted with H_2O. The aqueous solution was extracted 3× with diethyl ether. The extracts were then combined and washed 2× with brine. The organic extracts were dried over Na_2SO_4, filtered through cotton, and concentrated under reduced pressure. The resulting solid was wet loaded onto a silica gel column with CH_2Cl_2 and purified (20-50% Et_2O in Hexanes) to yield **19g** (0.359 g, 67%).

19g
^{1}H NMR (400 MHz, $(CD_3)_2SO$) δ 7.91 (d, 2H, *J* = 2.7 Hz), 7.82 (s, 2H), 7.77 (dd, 2H, *J* = 8.8, 2.7 Hz), 7.36 (d, 2H, *J* = 8.8 Hz), 3.75 (s, 6H) ppm; **^{13}C{1H} NMR** (100 MHz, $(CD_3)_2SO$) δ 163.3, 152.0, 149.8, 137.8, 134.4, 131.2, 129.7, 124.2, 123.4, 115.4, 52.6, 40.1, 39.9, 39.7, 39.5, 39.3, 39.1, 38.9 ppm; **M.P.** 149-151 °C; **HRMS (ESI-TOF)** m/z: $[M+Na]^+$ Calcd for $C_{22}H_{14}Cl_2N_2O_{10}Na$ 558.9917; Found 558.9916.

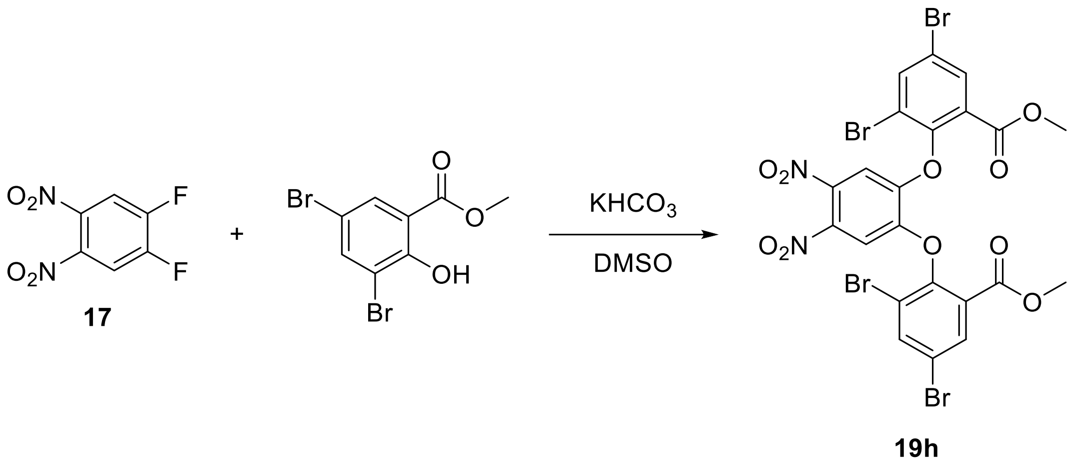

To a 20 mL scintillation vial equipped with a stir bar was added **1,2-dinitro-4,5-difluorobenzene 17** (1.02 g, 5 mmol), $KHCO_3$ (0.138 g, 4 mmol), and DMSO (5 mL). Methyl 3,5-dibromosalicylate (0.775 g, 2.5 mmol) was then added, and the reaction was allowed to stir at room temperature for 14 h. The reaction was then cooled to room temperature and diluted with H_2O and the aqueous layer was extracted 3× with diethyl ether. The ether extracts were then combined and washed 2× with brine. The organic layer was then dried over Na_2SO_4, filtered through cotton, and concentrated under reduced pressure. The crude solid was rinsed 3× with $CDCl_3$ to yield and dried to yield **19h** (0.367 g, 47%) as a faint yellow solid.

19h

^{1}H NMR (400 MHz, $CDCl_3$) δ 8.19 (s, 2H), 8.08 (s, 2H), 6.98 (s, 2H), 3.88 (s, 6H) ppm; **^{13}C{1H} NMR** (100 MHz, $CDCl_3$) δ 162.5, 148.6, 148.3, 140.6, 137.7, 134.6, 127.7, 120.8, 119.5, 110.9, 53.2 ppm; **M.P.** 213-216 °C.

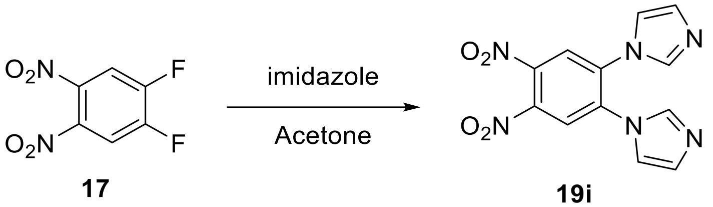

To a 20 mL scintillation vial equipped with a stir bar was added **1,2-dinitro-4,5-difluorobenzene 17** (0.204 g, 1 mmol) and acetone (5 mL). Imidazole (0.136 g, 2 mmol) was then added, and the reaction was allowed to stir at 50 °C (sand bath) for 14 h. Solution was evaporated under vacuum to yield **19i** (0.271 g, 90%) as a dark red solid.

19i

^{1}H NMR (400 MHz, $(CD_3)_2CO$) δ 8.68 (s, 2H), 7.74 (s, 2H), 7.14 (s, 2H), 7.08 (s, 2H) ppm; **^{13}C{1H}**

NMR (100 MHz, $(CD_3)_2CO$) δ 142.7, 138.2, 137.3, 130.9, 125.8, 120.9 ppm; **M.P.** 215-220 °C (decomposition); **HRMS (ESI-TOF)** m/z: $[M+H]^+$ Calcd for $C_{12}H_9N_6O_4$ 301.0680; Found 301.0674.

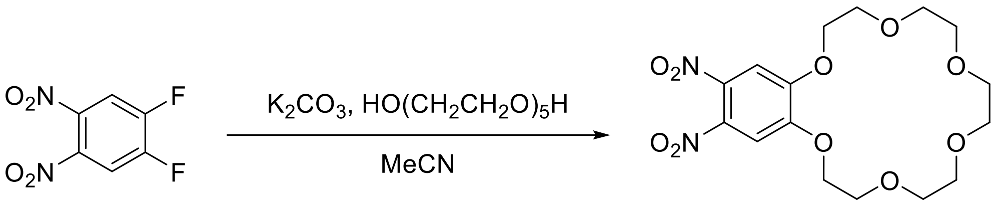

17

19j

To a 20 mL scintillation vial equipped with a stir bar was added **1,2-dinitro-4,5-difluorobenzene 17** (0.2 g, 1 mmol), K_2CO_3 (0.138 g, 4 mmol), and MeCN (4 mL). Pentaethyleneglycol (1 mL, 4.731 mmol) was then added, and the reaction was stirred at 65 °C (sand bath) for 14 h. The reaction was uncapped and condensed at 65 °C. The resulting residue was injected directly and purified by flash column chromatography (SiO_2, MeOH/EtOAc) to afford **19j** (0.371 g, 58%) as a clear pale-yellow oil. Characterization was in accordance with a previous report.[S5]

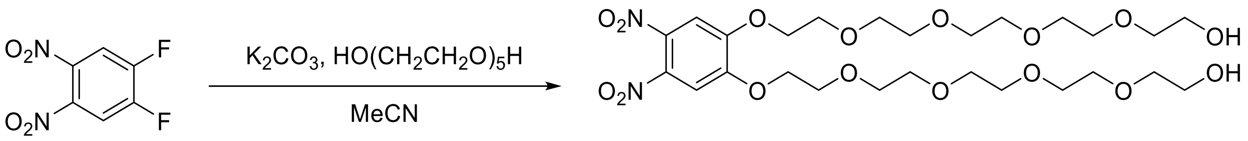

17

19k

To a 20 mL scintillation vial equipped with a stir bar was added **1,2-dinitro-4,5-difluorobenzene 17** (0.2 g, 1 mmol), K_2CO_3 (0.554 g, 4 mmol), and MeCN (4 mL). Pentaethyleneglycol (1 mL, 4.731 mmol) was then added, and the reaction was stirred at 65 °C (sand bath) for 14 h. The reaction was allowed to cool to room temperature and was concentrated under reduced pressure. The resulting residue was taken up with CH_2Cl_2, adsorbed onto silica under reduced pressure, and purified by flash column chromatography (SiO_2, MeOH/EtOAc) to afford **19k** (0.371 g, 58%) as a clear pale-yellow oil.

19k

^{1}H NMR (400 MHz, $CDCl_3$) δ 7.50 (s, 2H), 4.30 (quin, 4H, *J* = 4.3 Hz), 3.89 (t, 4H, *J* = 4.7 Hz), 3.77-3.37 (m, 32H) ppm; **^{13}C{1H} NMR** (100 MHz, $CDCl_3$) δ 151.6, 136.6, 109.3, 72.7, 72.7, 70.95, 70.5, 70.4, 70.4, 70.3, 70.3, 70.1, 70.00, 69.7, 69.3, 61.5, 61.4 ppm; **HRMS (ESI-TOF)** m/z: $[M+H]^+$ Calcd for $C_{26}H_{45}FN_2O_6$ 641.2764; Found 641.2754.

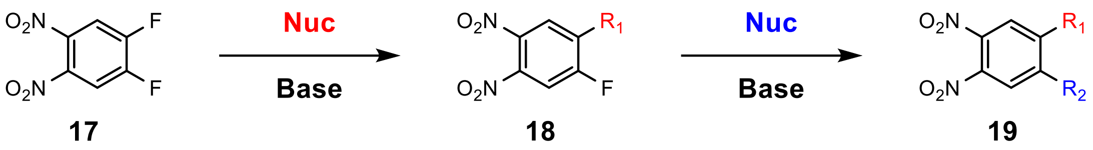

Table S1. Summary of conditions for the S_NAr/S_NAr method.

	Conditions			
**Entry**	**Solvent**	**Base**	**Temperature (°C)**	**Time (h)**
**18a**	H_2O/Acetone	—	rt	14
**18b**	DMSO	—	65	14
**18c**	MeOH	DIPEA	60	14
**18d**	EtOH	DIPEA	60	14
**18e**	DMSO	K_2CO_3	60	14
**18f**	DMSO	K_2CO_3	60	14
**18g**	DMSO	KCN	60	14
**18h**	DMSO	DIPEA	60	24
**18i**	DMSO	$KHCO_3$	60	14
**18j**	DMSO	$KHCO_3$	30	4
**18k**	DMSO	$KHCO_3$	30	4
**18l**	DMSO	$KHCO_3$	30	24
**18m**	Acetone	—	50	14
**19a**	DMSO	—	30	14
**19b**	DMSO	—	60	14
**19c**	MeOH	K_2CO_3	60	14
**19d**	EtOH	K_2CO_3	60	14
**19e**	DMSO	K_2CO_3	60	14
**19f**	DMSO	$KHCO_3$	rt	14
**19g**	DMSO	$KHCO_3$	rt	14
**19h**	DMSO	$KHCO_3$	rt	14
**19i**	Acetone	—	50	14
**19j**	MeCN	K_2CO_3	65	14
**19k**	MeCN	K_2CO_3	65	14

II. References

(S1) Lewis, N. B.; Bisbey, R. P.; Westendorff, K. S.; Soudackov, A. V.; Surendranath, Y. A Molecular-Level Mechanistic Framework for Interfacial Proton-Coupled Electron Transfer Kinetics. *Nat. Chem.* **2024**, *16* (3), 343–352, DOI: 10.1038/s41557-023-01400-0.

(S2) Nikitin, S.; Diness, F. Tuning Green Explosives through S_NAr Chemistry. *Chem. – Asian J.* **2024**, *19* (13), e202400212, DOI: 10.1002/asia.202400212.

(S3) Fan, K. W.; Peterson, M. B.; Ellersdorfer, P.; Granville, A. M. Expanding the Aqueous-Based Redox-Facilitated Self-Polymerization Chemistry of Catecholamines to 5,6-Dihydroxy-1*H*-Benzimidazole and Its 2-Substituted Derivatives. *RSC Adv.* **2016**, *6* (30), 25203–25214, DOI: 10.1039/C5RA25590B.

(S4) Yang, T.; Li, X.; Deng, S.; Qi, X.; Cong, H.; Cheng, H.-G.; Shi, L.; Zhou, Q.; Zhuang, L. From N–H Nitration to Controllable Aromatic Mononitration and Dinitration–The Discovery of a Versatile and Powerful *N*-Nitropyrazole Nitrating Reagent. *JACS Au* **2022**, *2* (9), 2152–2161, DOI: 10.1021/jacsau.2c00413.

(S5) Jradi, F. M.; Al-Sayah, M. H.; Kaafarani, B. R. Synthesis and Metal-Binding Studies of a Novel Pyrene Discotic. *Tetrahedron Lett.* **2008**, *49* (2), 238–242, DOI: 10.1016/j.tetlet.2007.11.078.

III. NMR Spectroscopy Data

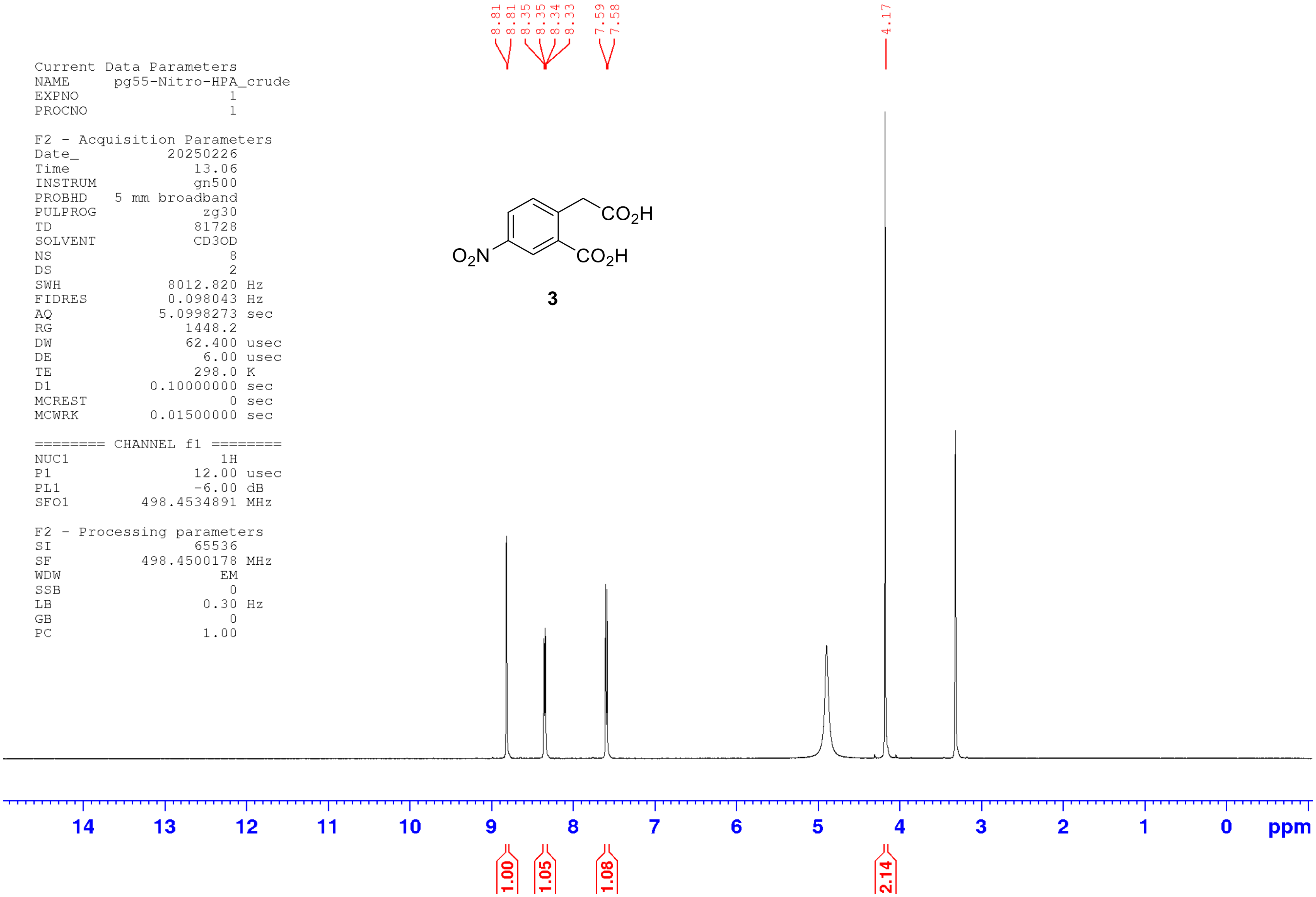

$^{13}C\{1H\}$

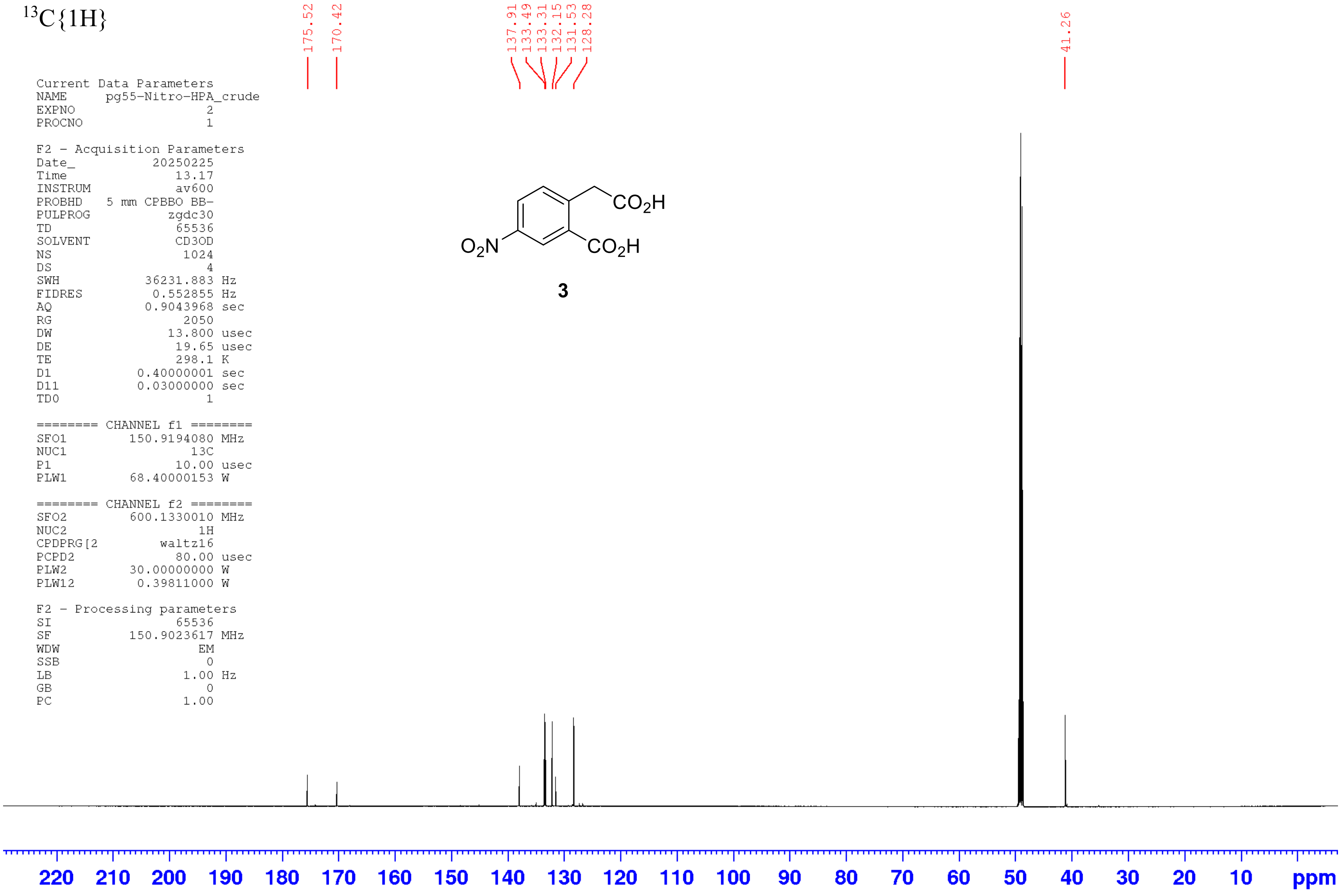
175.52
170.42
137.91
133.49
133.31
132.15
131.53
128.28
41.26
Current Data Parameters
NAME pg55-Nitro-HPA_crude
EXPNO 2
PROCNO 1
F2 - Acquisition Parameters
Date_ 20250225
Time 13.17
INSTRUM av600
PROBHD 5 mm CPBBO BB-
PULPROG zgdc30
TD 65536
SOLVENT CD3OD
NS 1024
DS 4
SWH 36231.883 Hz
FIDRES 0.552855 Hz
AQ 0.9043968 sec
RG 2050
DW 13.800 usec
DE 19.65 usec
TE 298.1 K
D1 0.40000001 sec
D11 0.03000000 sec
TD0 1
======== CHANNEL f1 ========
SFO1 150.9194080 MHz
NUC1 13C
P1 10.00 usec
PLW1 68.40000153 W
======== CHANNEL f2 ========
SFO2 600.1330010 MHz
NUC2 1H
CPDPRG[2 waltz16
PCPD2 80.00 usec
PLW2 30.00000000 W
PLW12 0.39811000 W
F2 - Processing parameters
SI 65536
SF 150.9023617 MHz
WDW EM
SSB 0
LB 1.00 Hz
GB 0
PC 1.00
220 210 200 190 180 170 160 150 140 130 120 110 100 90 80 70 60 50 40 30 20 10 ppm
CO2H
O2N
CO2H

3

```
Current Data Parameters
NAME      pg56-amino-HPA_crude
EXPNO                    1
PROCNO                   1

F2 - Acquisition Parameters
Date_             20230911
Time                 12.56
INSTRUM              gn500
PROBHD   5 mm broadband
PULPROG               zg30
TD                   81728
SOLVENT               DMSO
NS                       8
DS                       2
SWH               8012.820 Hz
FIDRES            0.098043 Hz
AQ               5.0998273 sec
RG                  1824.6
DW                  62.400 usec
DE                    6.00 usec
TE                   298.1 K
D1              0.10000000 sec
MCREST                   0 sec
MCWRK           0.01500000 sec

======== CHANNEL f1 ========
NUC1                    1H
P1                   12.00 usec
PL1                  -6.00 dB
SFO1           498.5534899 MHz

F2 - Processing parameters
SI                   65536
SF             498.5500086 MHz
WDW                     EM
SSB                      0
LB                    0.30 Hz
GB                       0
PC                    1.00
```

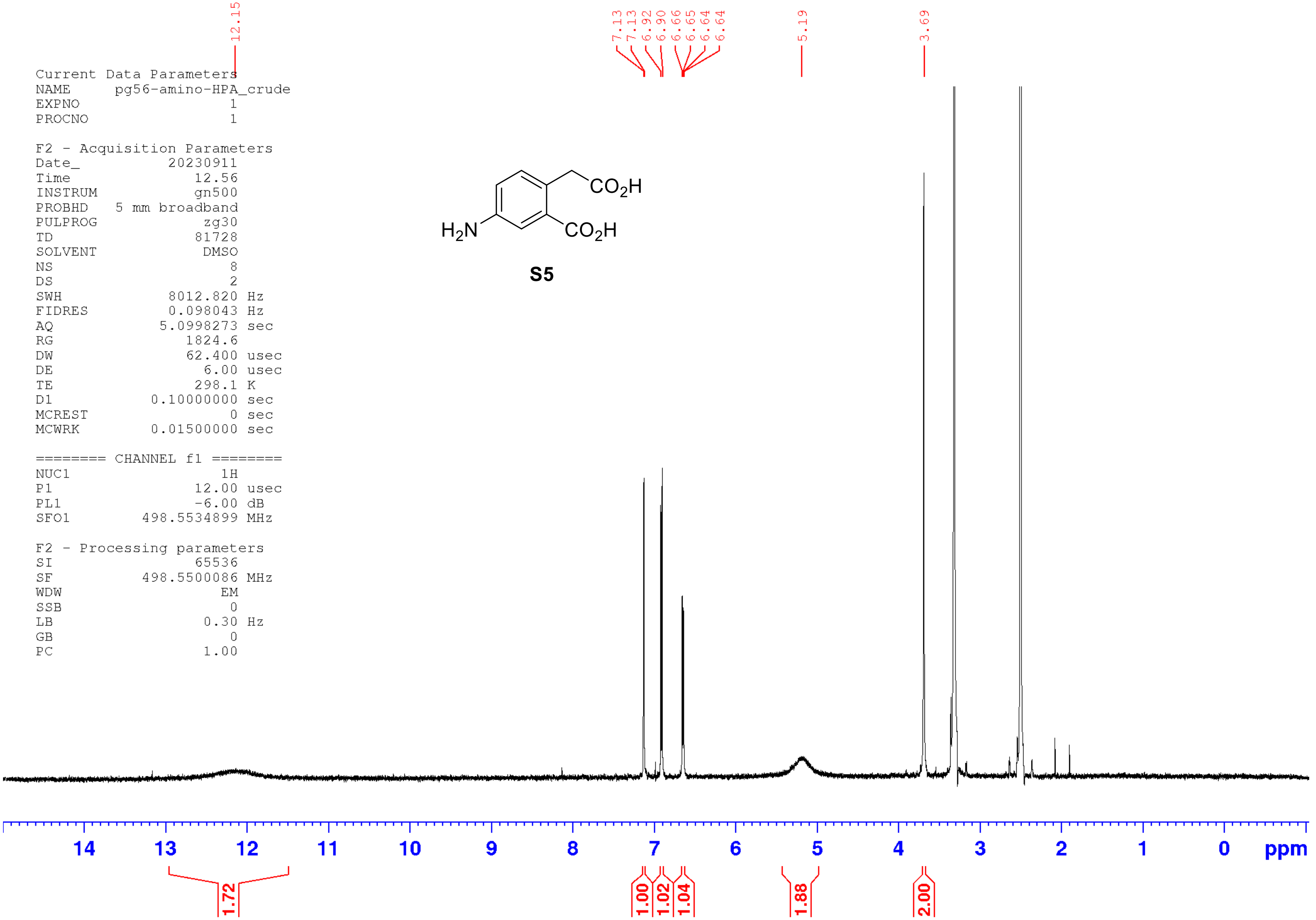

$^{13}C\{1H\}$

```
Current Data Parameters
NAME       pg56-amino-HPA_crude
EXPNO                    2
PROCNO                   1

F2 - Acquisition Parameters
Date_            20250227
Time                16.52
INSTRUM             av600
PROBHD    5 mm CPBBO BB-
PULPROG           zgdc30
TD                 65536
SOLVENT             DMSO
NS                  1024
DS                     4
SWH            36231.883 Hz
FIDRES          0.552855 Hz
AQ             0.9043968 sec
RG                  2050
DW                13.800 usec
DE                 19.65 usec
TE                 298.1 K
D1            0.40000001 sec
D11           0.03000000 sec
TD0                    1

======== CHANNEL f1 ========
SFO1         150.9194080 MHz
NUC1                 13C
P1                 10.00 usec
PLW1         68.40000153 W

======== CHANNEL f2 ========
SFO2         600.1330010 MHz
NUC2                  1H
CPDPRG[2          waltz16
PCPD2              80.00 usec
PLW2         30.00000000 W
PLW12         0.39811000 W

F2 - Processing parameters
SI                 65536
SF           150.9028782 MHz
WDW                   EM
SSB                    0
LB                  1.00 Hz
GB                     0
PC                  1.00
```

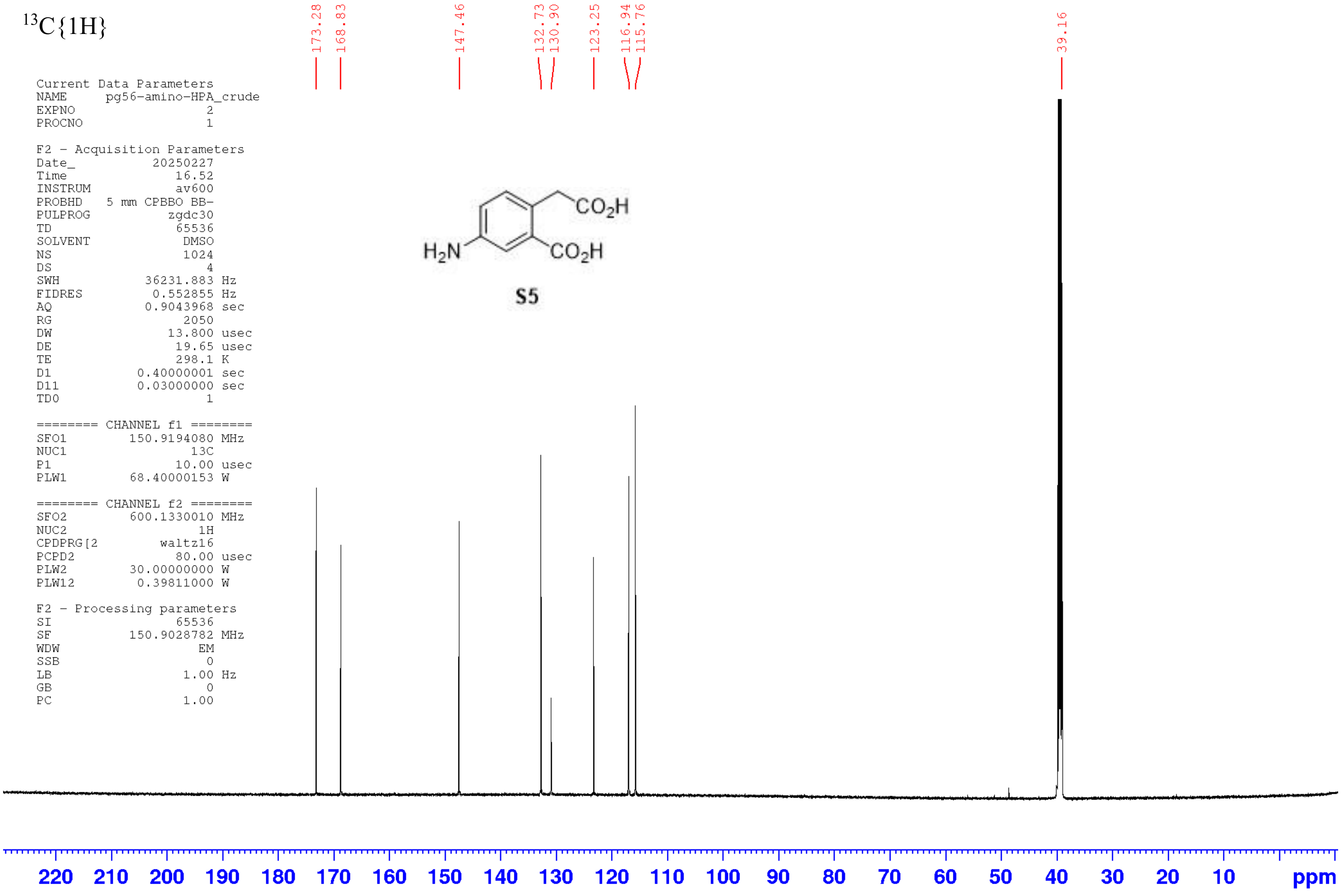

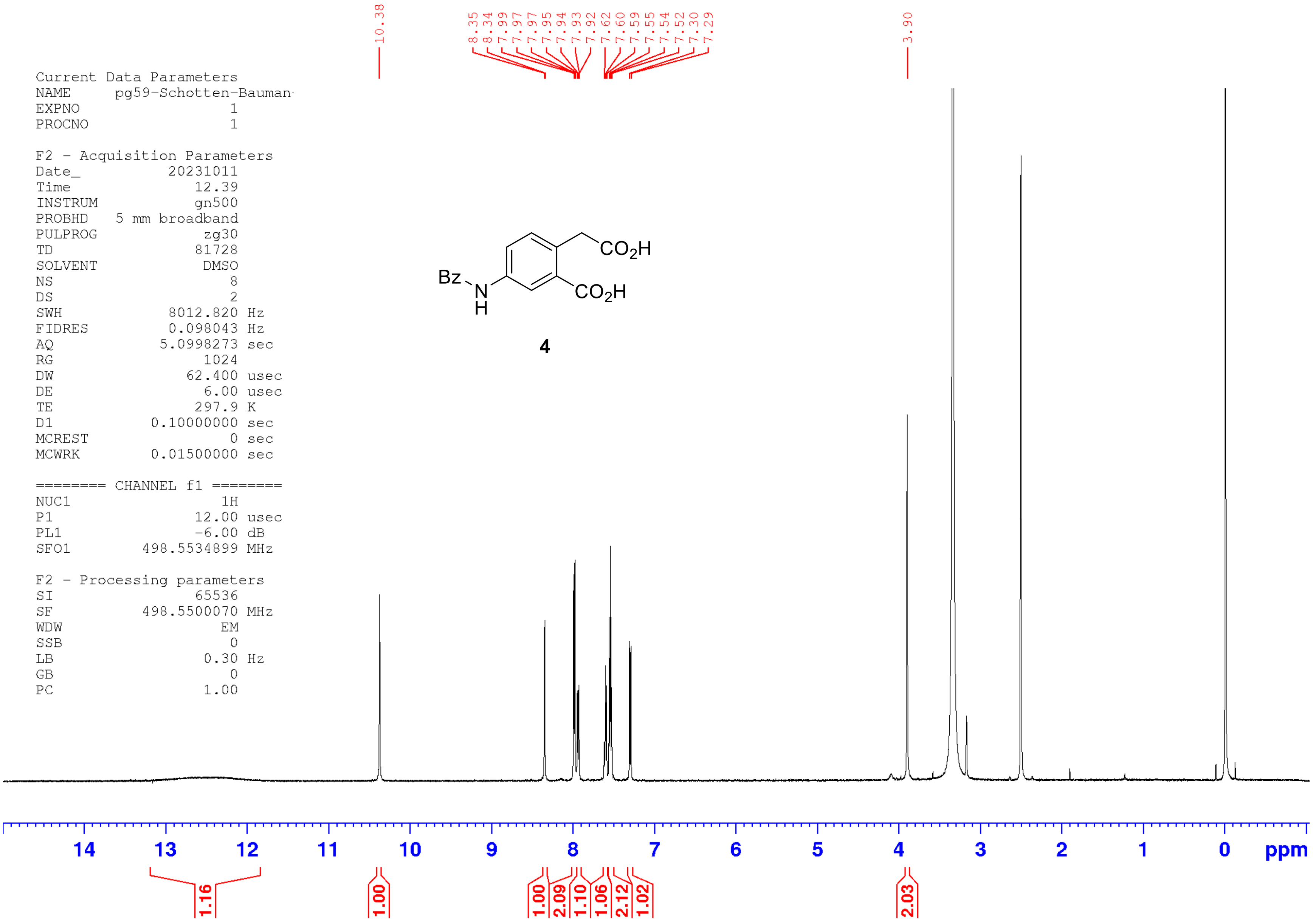
Current Data Parameters
NAME pg59-Schotten-Bauman
EXPNO 1
PROCNO 1
F2 - Acquisition Parameters
Date_ 20231011
Time 12.39
INSTRUM gn500
PROBHD 5 mm broadband
PULPROG zg30
TD 81728
SOLVENT DMSO
NS 8
DS 2
SWH 8012.820 Hz
FIDRES 0.098043 Hz
AQ 5.0998273 sec
RG 1024
DW 62.400 usec
DE 6.00 usec
TE 297.9 K
D1 0.10000000 sec
MCREST 0 sec
MCWRK 0.01500000 sec
======== CHANNEL f1 ========
NUC1 1H
P1 12.00 usec
PL1 -6.00 dB
SFO1 498.5534899 MHz
F2 - Processing parameters
SI 65536
SF 498.5500070 MHz
WDW EM
SSB 0
LB 0.30 Hz
GB 0
PC 1.00
10.38
8.35 8.34 7.99 7.97 7.97 7.95 7.94 7.93 7.92 7.62 7.60 7.59 7.55 7.54 7.52 7.30 7.29
3.90
Bz
N
H
CO2H
CO2H
4
14 13 12 11 10 9 8 7 6 5 4 3 2 1 0 ppm
1.16 1.00 1.00 2.09 1.10 1.06 2.12 1.02 2.03

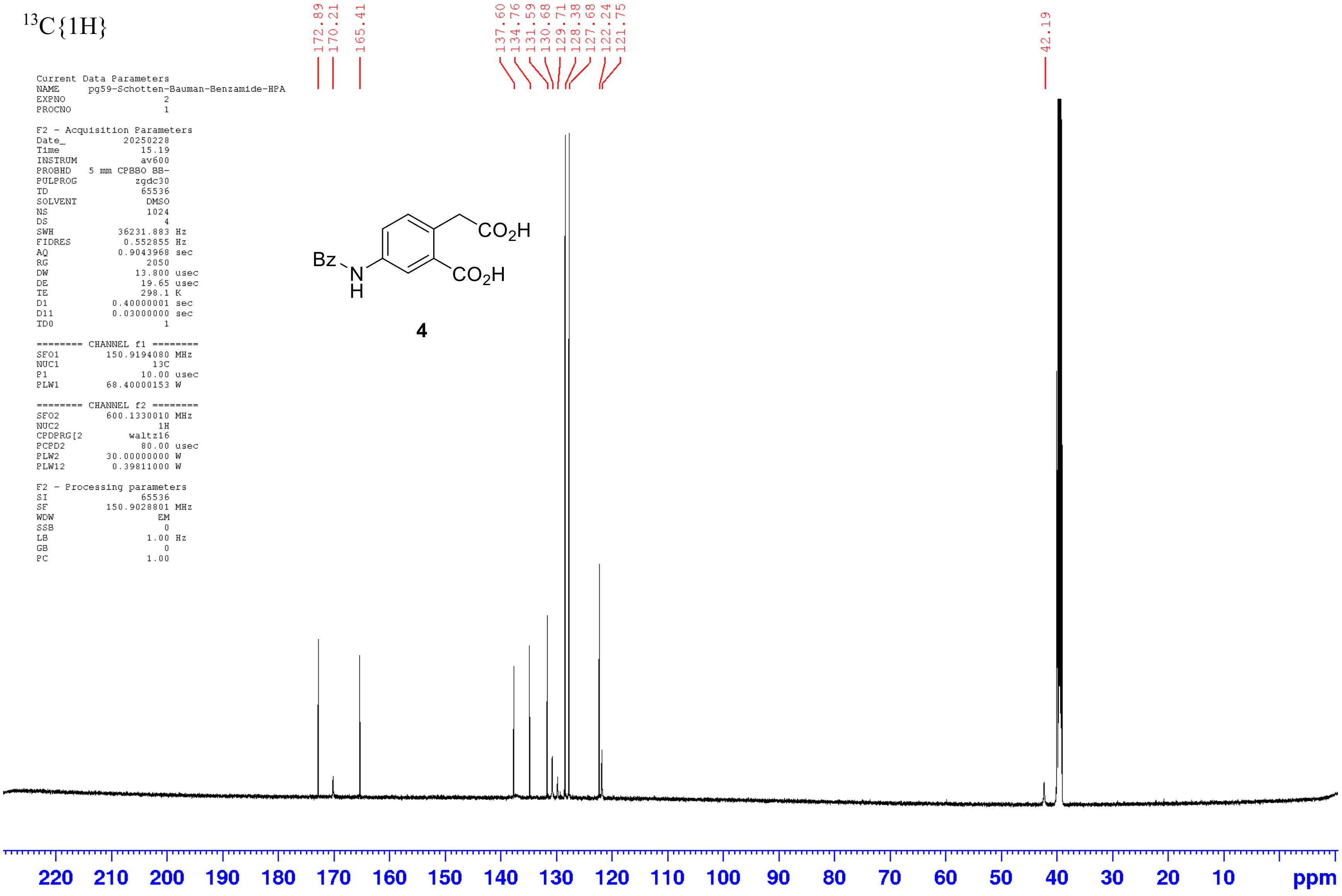

13C{1H}
172.89
170.21
165.41
137.60
134.76
131.59
130.68
129.71
128.38
127.68
122.24
121.75
42.19
Current Data Parameters
NAME pg59-Schotten-Bauman-Benzamide-HPA
EXPNO 2
PROCNO 1
F2 - Acquisition Parameters
Date_ 20250228
Time 15.19
INSTRUM av600
PROBHD 5 mm CPBBO BB-
PULPROG zgdc30
TD 65536
SOLVENT DMSO
NS 1024
DS 4
SWH 36231.883 Hz
FIDRES 0.552855 Hz
AQ 0.9043968 sec
RG 2050
DW 13.800 usec
DE 19.65 usec
TE 298.1 K
D1 0.40000001 sec
D11 0.03000000 sec
TD0 1
======== CHANNEL f1 ========
SFO1 150.9194080 MHz
NUC1 13C
P1 10.00 usec
PLW1 68.40000153 W
======== CHANNEL f2 ========
SFO2 600.1330010 MHz
NUC2 1H
CPDPRG[2 waltz16
PCPD2 80.00 usec
PLW2 30.00000000 W
PLW12 0.39811000 W
F2 - Processing parameters
SI 65536
SF 150.9028801 MHz
WDW EM
SSB 0
LB 1.00 Hz
GB 0
PC 1.00
Bz
N
H
CO2H
CO2H
4
220 210 200 190 180 170 160 150 140 130 120 110 100 90 80 70 60 50 40 30 20 10 ppm

```
Current Data Parameters
NAME                 pg70
EXPNO                   1
PROCNO                  1

F2 - Acquisition Parameters
Date_            20231129
Time                13.32
INSTRUM             gn500
PROBHD   5 mm broadband
PULPROG              zg30
TD                  81728
SOLVENT             CDCl3
NS                      8
DS                      2
SWH              8012.820 Hz
FIDRES           0.098043 Hz
AQ              5.0998273 sec
RG                     64
DW                 62.400 usec
DE                   6.00 usec
TE                  298.2 K
D1             0.10000000 sec
MCREST                  0 sec
MCWRK          0.01500000 sec

======== CHANNEL f1 ========
NUC1                   1H
P1                  12.00 usec
PL1                 -6.00 dB
SFO1          498.5534899 MHz

F2 - Processing parameters
SI                  65536
SF            498.5500247 MHz
WDW                    EM
SSB                     0
LB                   0.30 Hz
GB                      0
PC                   1.00
```

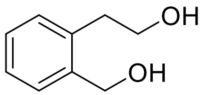

S6

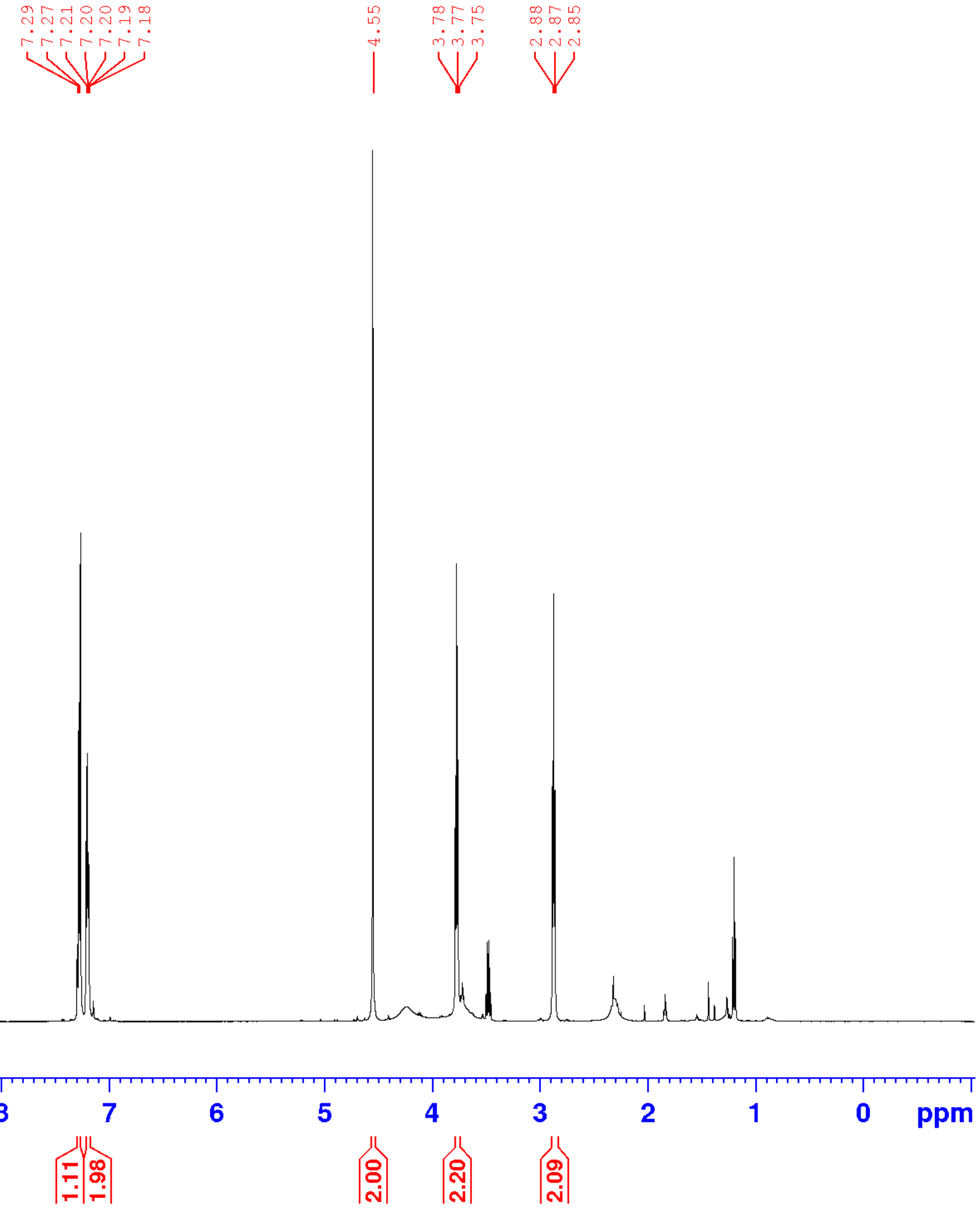

$^{13}C\{1H\}$

```
Current Data Parameters
NAME                 pg70
EXPNO                   2
PROCNO                  1

F2 - Acquisition Parameters
Date_            20241114
Time                14.00
INSTRUM             av600
PROBHD   5 mm CPBBO BB-
PULPROG           zgdc30
TD                  65536
SOLVENT             CDCl3
NS                    850
DS                      4
SWH             36231.883 Hz
FIDRES           0.552855 Hz
AQ              0.9043968 sec
RG                   2050
DW                 13.800 usec
DE                  19.65 usec
TE                  298.0 K
D1             0.40000001 sec
D11            0.03000000 sec
TD0                     1

======== CHANNEL f1 ========
SFO1          150.9194080 MHz
NUC1                  13C
P1                  10.00 usec
PLW1          68.40000153 W

======== CHANNEL f2 ========
SFO2          600.1330010 MHz
NUC2                   1H
CPDPRG[2          waltz16
PCPD2               80.00 usec
PLW2          30.00000000 W
PLW12          0.39811000 W

F2 - Processing parameters
SI                  65536
SF            150.9027959 MHz
WDW                    EM
SSB                     0
LB                   1.00 Hz
GB                      0
PC                   1.00
```

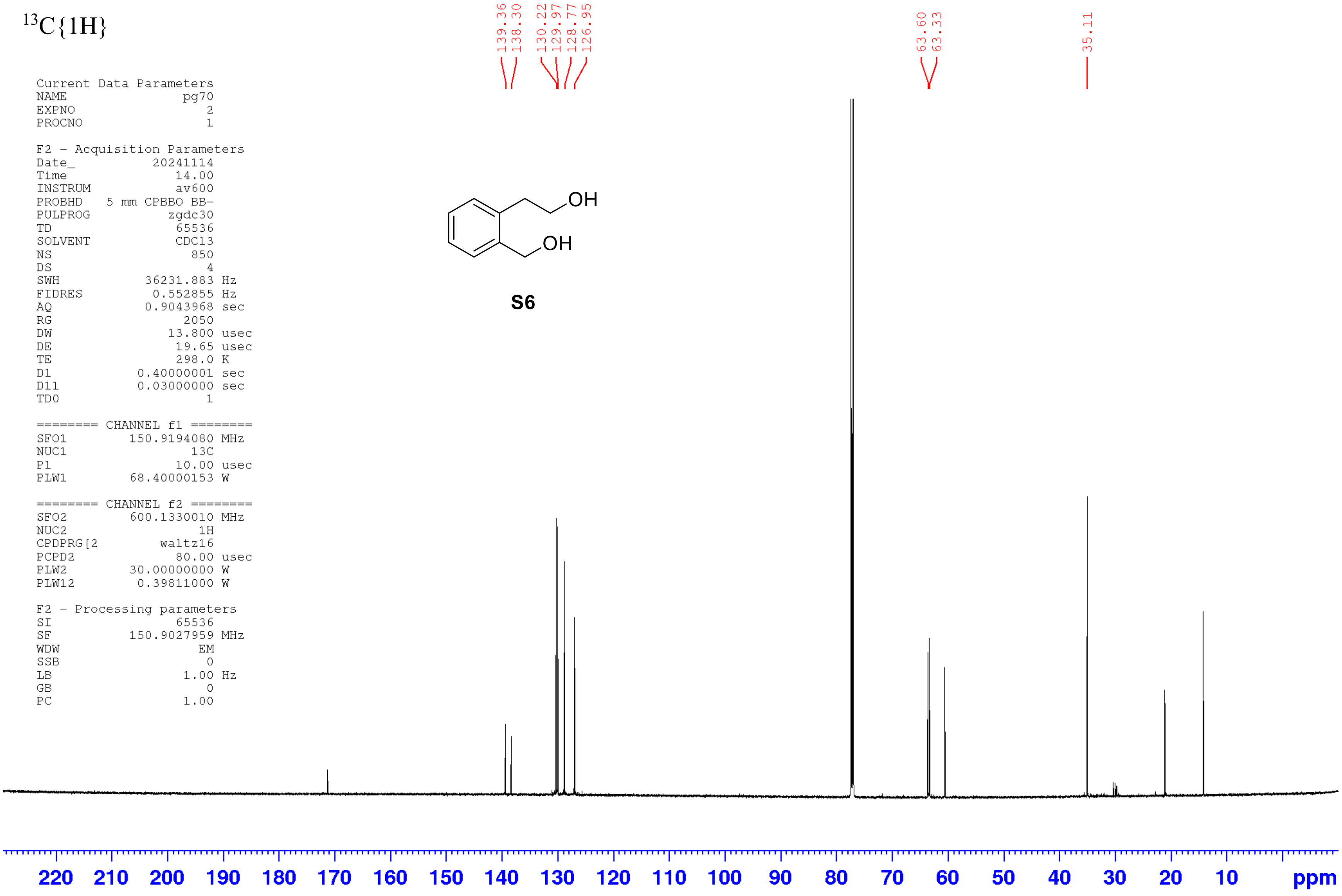

S6

Current Data Parameters
NAME pg71
EXPNO 1
PROCNO 1

F2 - Acquisition Parameters
Date_ 20231201
Time 16.15
INSTRUM gn500
PROBHD 5 mm broadband
PULPROG zg30
TD 81728
SOLVENT CDCl3
NS 8
DS 2
SWH 8012.820 Hz
FIDRES 0.098043 Hz
AQ 5.0998273 sec
RG 80.6
DW 62.400 usec
DE 6.00 usec
TE 298.4 K
D1 0.10000000 sec
MCREST 0 sec
MCWRK 0.01500000 sec

======== CHANNEL f1 ========
NUC1 1H
P1 12.00 usec
PL1 -6.00 dB
SFO1 498.5534899 MHz

F2 - Processing parameters
SI 65536
SF 498.5500000 MHz
WDW EM
SSB 0
LB 0.30 Hz
GB 0
PC 1.00

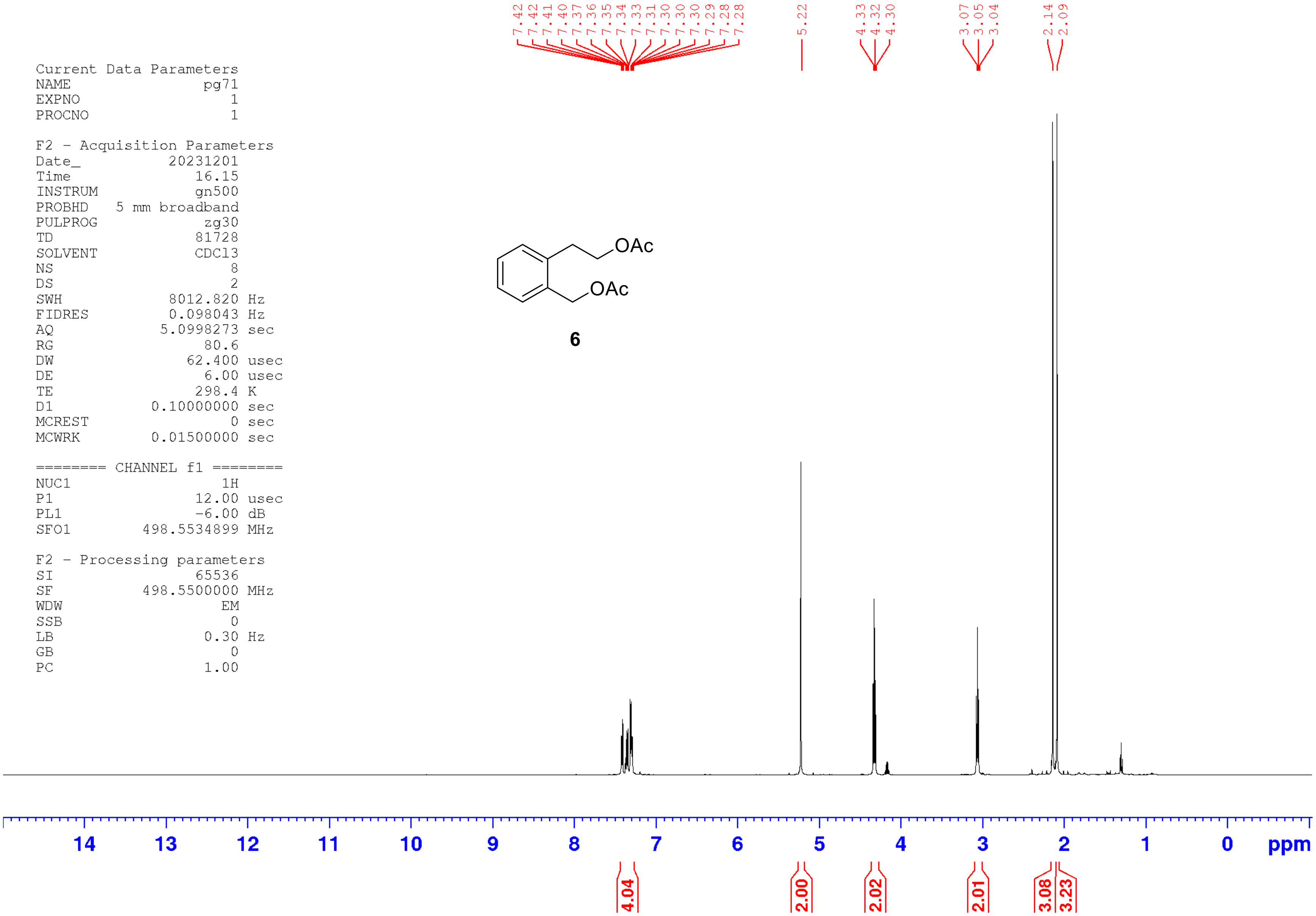

$^{13}C\{1H\}$

```
Current Data Parameters
NAME                 pg71
EXPNO                   2
PROCNO                  1

F2 - Acquisition Parameters
Date_           20250215
Time               17.44
INSTRUM            av600
PROBHD   5 mm CPBBO BB-
PULPROG          zgdc30
TD                 65536
SOLVENT            CDCl3
NS                  1024
DS                     4
SWH            36231.883 Hz
FIDRES          0.552855 Hz
AQ             0.9043968 sec
RG                  2050
DW                13.800 usec
DE                 19.65 usec
TE                 298.1 K
D1            0.40000001 sec
D11           0.03000000 sec
TD0                    1

======== CHANNEL f1 ========
SFO1         150.9194080 MHz
NUC1                 13C
P1                 10.00 usec
PLW1         68.40000153 W

======== CHANNEL f2 ========
SFO2         600.1330010 MHz
NUC2                  1H
CPDPRG[2         waltz16
PCPD2              80.00 usec
PLW2         30.00000000 W
PLW12         0.39811000 W

F2 - Processing parameters
SI                 65536
SF           150.9027959 MHz
WDW                   EM
SSB                    0
LB                  1.00 Hz
GB                     0
PC                  1.00
```

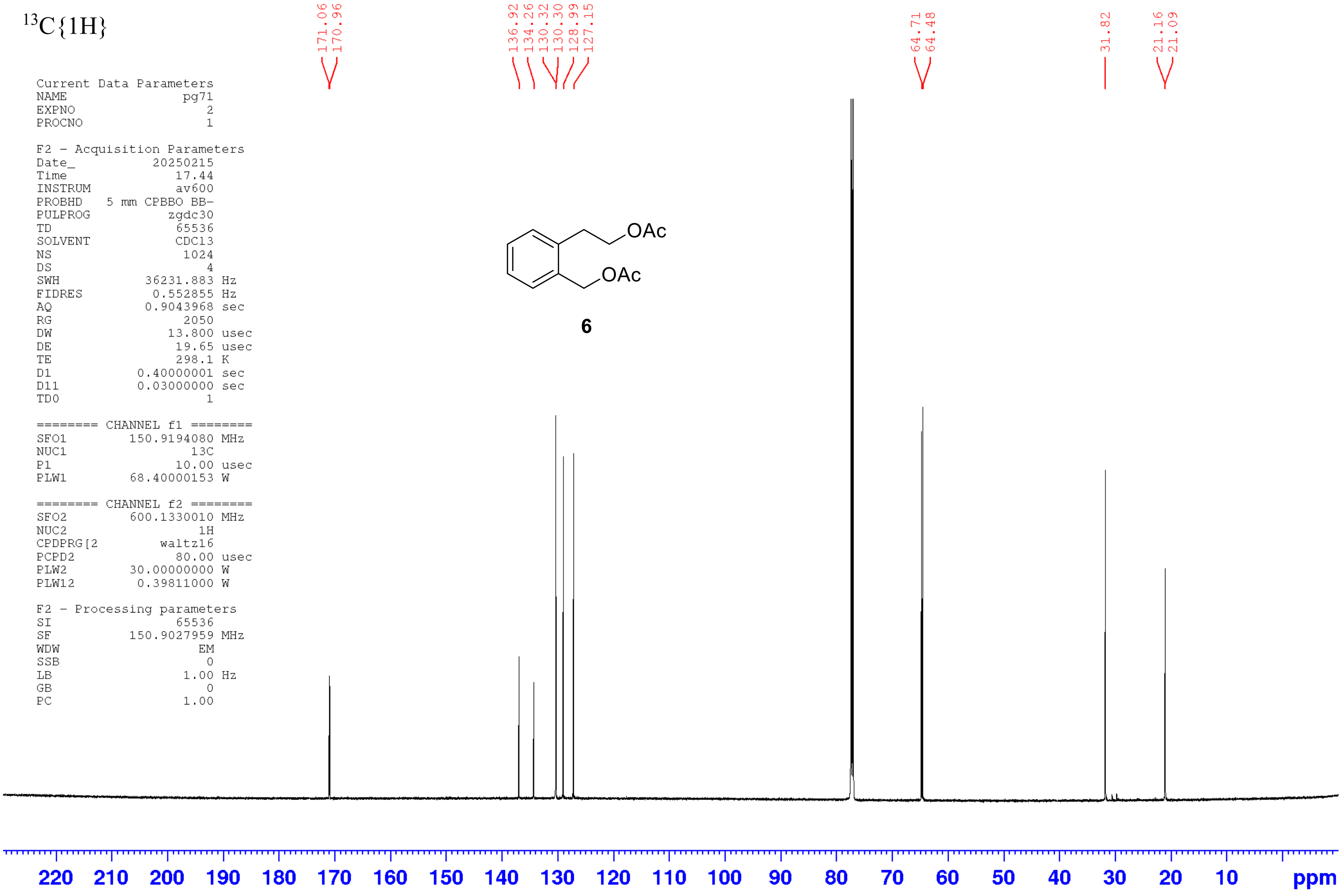

```
Current Data Parameters
NAME                   pg73
EXPNO                     1
PROCNO                    1

F2 - Acquisition Parameters
Date_              20231215
Time                  12.28
INSTRUM               gn500
PROBHD   5 mm broadband
PULPROG                zg30
TD                    81728
SOLVENT               CD3OD
NS                        8
DS                        2
SWH               8012.820 Hz
FIDRES            0.098043 Hz
AQ               5.0998273 sec
RG                   1149.4
DW                   62.400 usec
DE                     6.00 usec
TE                    298.2 K
D1               0.10000000 sec
MCREST                    0 sec
MCWRK            0.01500000 sec

======== CHANNEL f1 ========
NUC1                     1H
P1                    12.00 usec
PL1                   -6.00 dB
SFO1            498.5534899 MHz

F2 - Processing parameters
SI                    65536
SF              498.5500131 MHz
WDW                      EM
SSB                       0
LB                     0.30 Hz
GB                        0
PC                     1.00
```

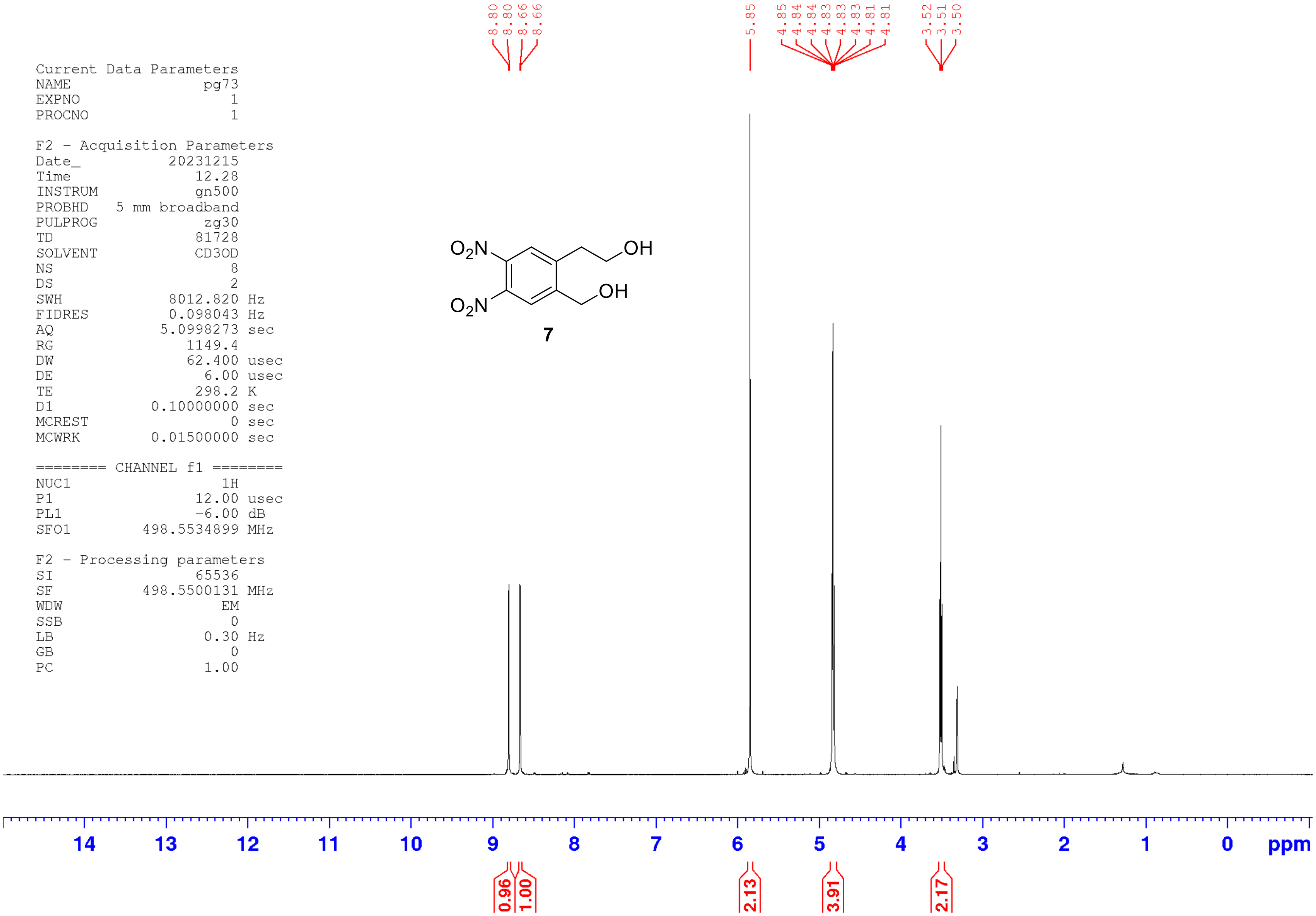

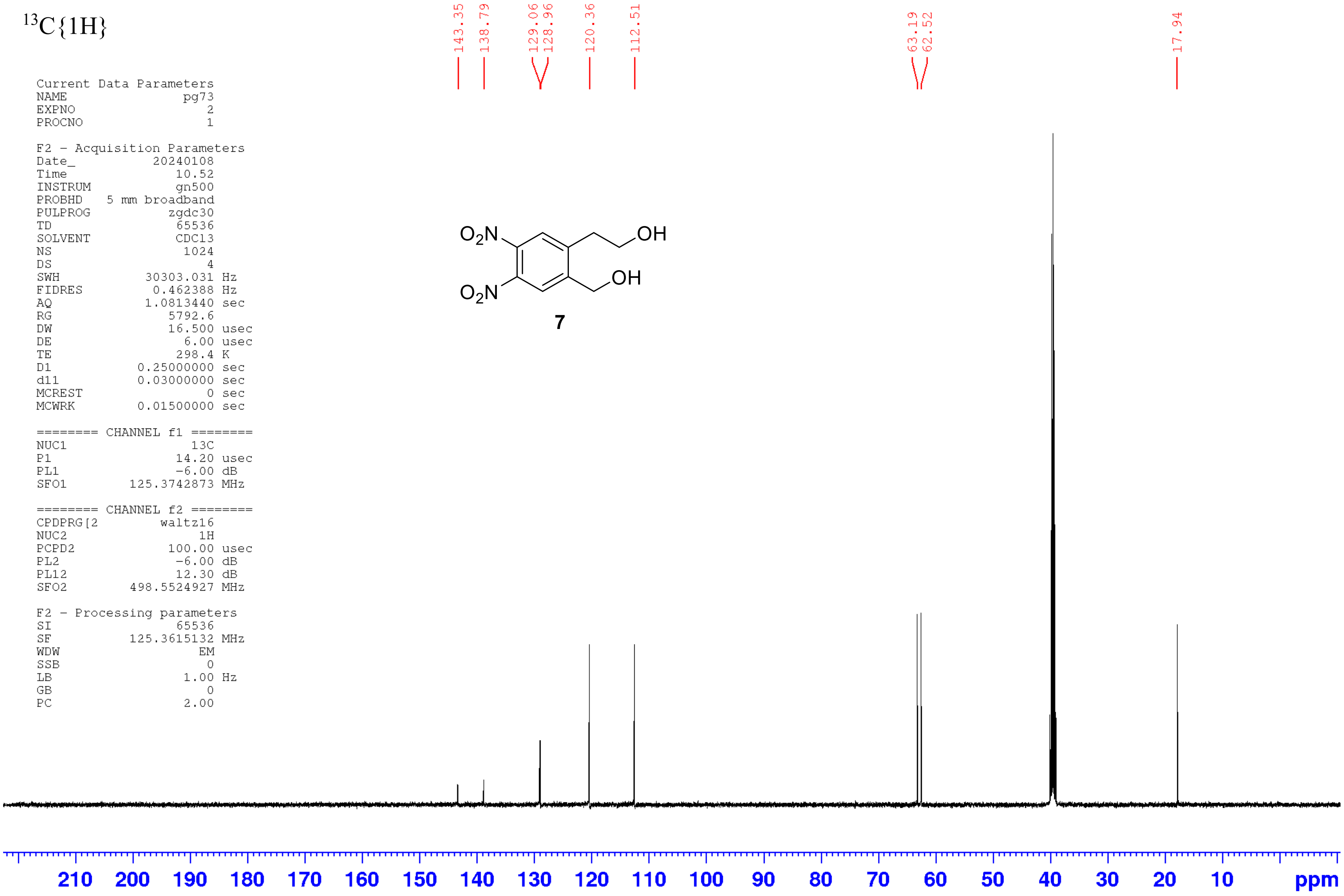
$^{13}C\{1H\}$
Current Data Parameters
NAME pg73
EXPNO 2
PROCNO 1
F2 - Acquisition Parameters
Date_ 20240108
Time 10.52
INSTRUM gn500
PROBHD 5 mm broadband
PULPROG zgdc30
TD 65536
SOLVENT CDCl3
NS 1024
DS 4
SWH 30303.031 Hz
FIDRES 0.462388 Hz
AQ 1.0813440 sec
RG 5792.6
DW 16.500 usec
DE 6.00 usec
TE 298.4 K
D1 0.25000000 sec
d11 0.03000000 sec
MCREST 0 sec
MCWRK 0.01500000 sec
======== CHANNEL f1 ========
NUC1 13C
P1 14.20 usec
PL1 -6.00 dB
SFO1 125.3742873 MHz
======== CHANNEL f2 ========
CPDPRG[2 waltz16
NUC2 1H
PCPD2 100.00 usec
PL2 -6.00 dB
PL12 12.30 dB
SFO2 498.5524927 MHz
F2 - Processing parameters
SI 65536
SF 125.3615132 MHz
WDW EM
SSB 0
LB 1.00 Hz
GB 0
PC 2.00
143.35
138.79
129.06
128.96
120.36
112.51
63.19
62.52
17.94
210 200 190 180 170 160 150 140 130 120 110 100 90 80 70 60 50 40 30 20 10 ppm
O2N
O2N
OH
OH

7

```
Current Data Parameters
NAME                pg109
EXPNO                   1
PROCNO                  1

F2 - Acquisition Parameters
Date_            20240712
Time                 9.56 h
INSTRUM             spect
PROBHD   Z149000_0038 (
PULPROG              zg30
TD                  65536
SOLVENT              MeOD
NS                      8
DS                      2
SWH              6402.049 Hz
FIDRES           0.195375 Hz
AQ              5.1183615 sec
RG                 207.08
DW                 78.100 usec
DE                  13.05 usec
TE                  298.0 K
D1             0.10000000 sec
TD0                     1
SFO1          400.1328009 MHz
NUC1                   1H
P0                   3.79 usec
P1                  11.38 usec
PLW1           7.41450024 W

F2 - Processing parameters
SI                  65536
SF            400.1300078 MHz
WDW                    EM
SSB                     0
LB                   0.30 Hz
GB                      0
PC                   1.00
```

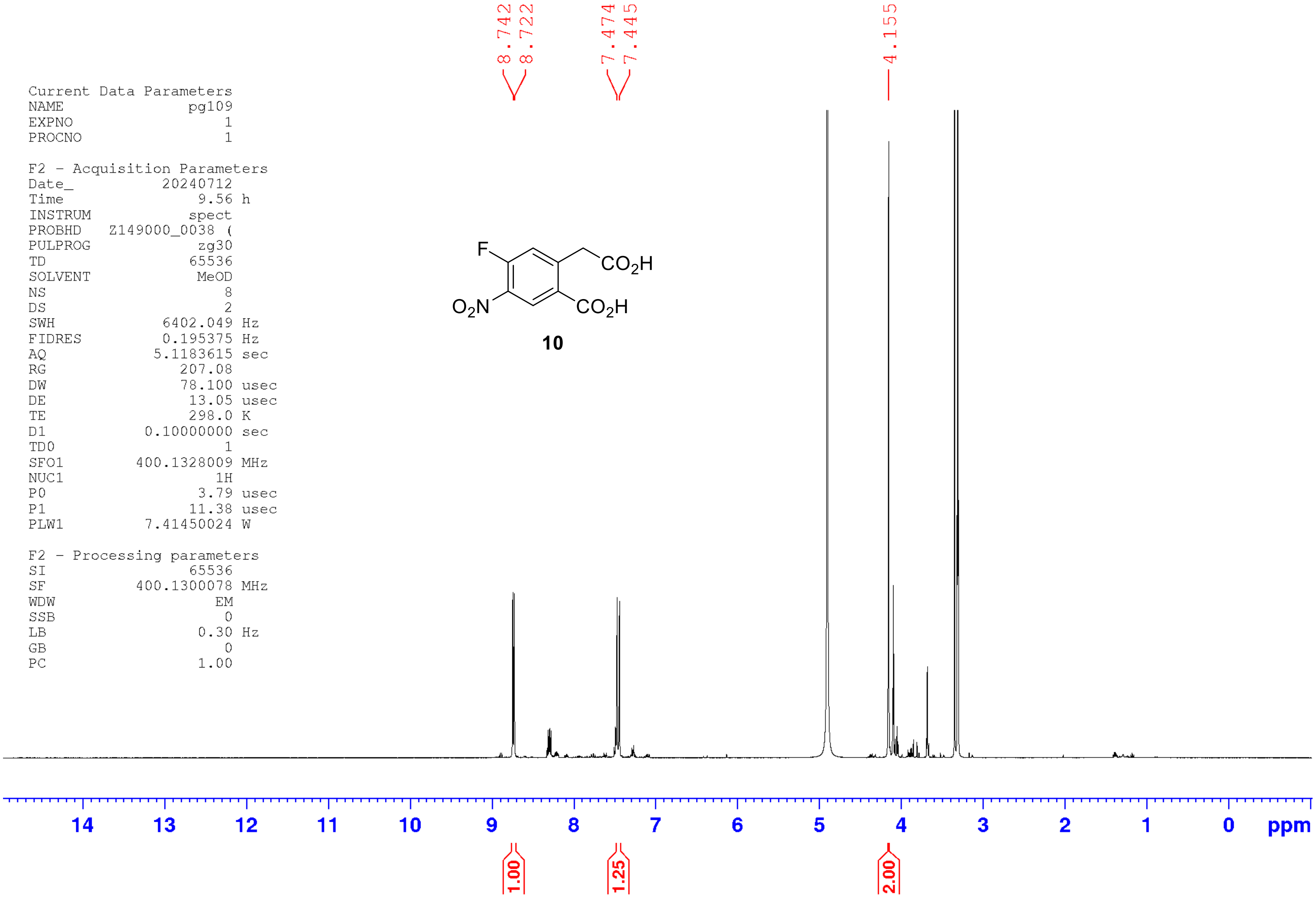

$^{13}C\{1H\}$

```
Current Data Parameters
NAME                  pg109
EXPNO                     2
PROCNO                    1

F2 - Acquisition Parameters
Date_              20241029
Time                  12.29
INSTRUM               av600
PROBHD    5 mm CPBBO BB-
PULPROG             zgdc30
TD                    65536
SOLVENT               CD3OD
NS                     1024
DS                        4
SWH              36231.883 Hz
FIDRES            0.552855 Hz
AQ               0.9043968 sec
RG                     2050
DW                   13.800 usec
DE                    19.65 usec
TE                    297.9 K
D1               0.40000001 sec
D11              0.03000000 sec
TD0                       1

======== CHANNEL f1 ========
SFO1         150.9194080 MHz
NUC1                    13C
P1                    10.00 usec
PLW1         68.40000153 W

======== CHANNEL f2 ========
SFO2         600.1330010 MHz
NUC2                     1H
CPDPRG[2            waltz16
PCPD2                 80.00 usec
PLW2         30.00000000 W
PLW12         0.39811000 W

F2 - Processing parameters
SI                    65536
SF           150.9026009 MHz
WDW                      EM
SSB                       0
LB                     1.00 Hz
GB                        0
PC                     1.00
```

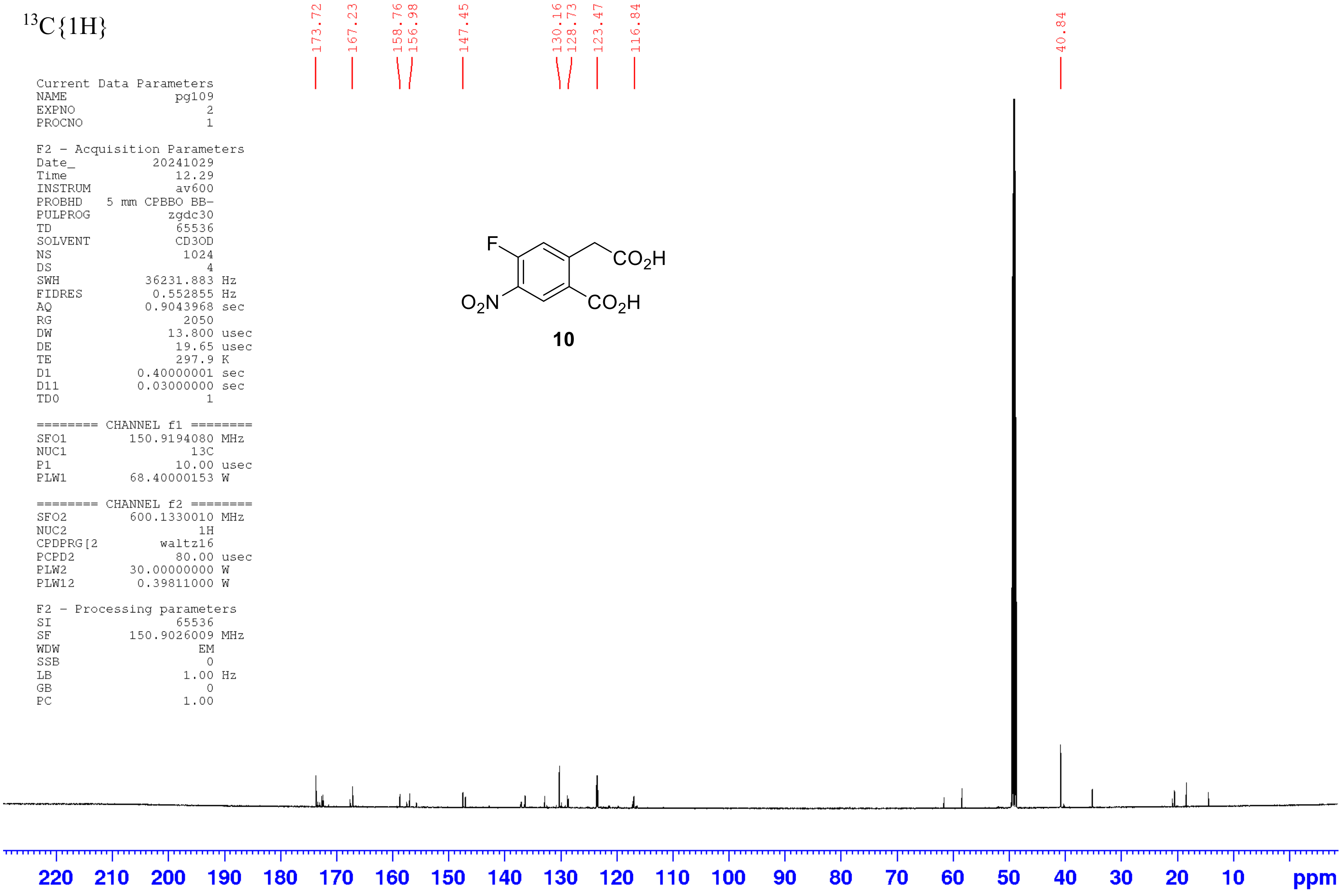

10

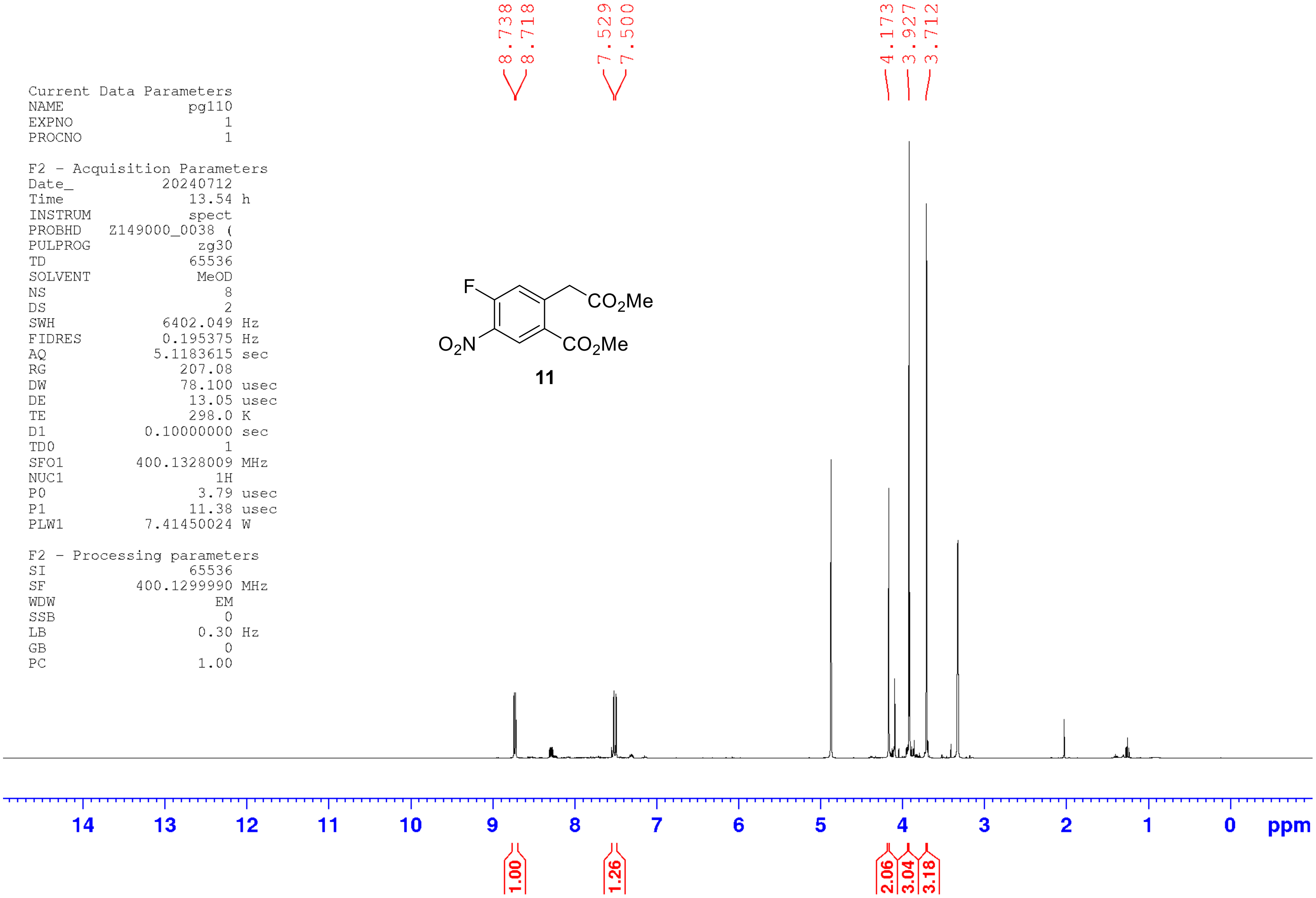
Current Data Parameters
NAME pg110
EXPNO 1
PROCNO 1
F2 - Acquisition Parameters
Date_ 20240712
Time 13.54 h
INSTRUM spect
PROBHD Z149000_0038 (
PULPROG zg30
TD 65536
SOLVENT MeOD
NS 8
DS 2
SWH 6402.049 Hz
FIDRES 0.195375 Hz
AQ 5.1183615 sec
RG 207.08
DW 78.100 usec
DE 13.05 usec
TE 298.0 K
D1 0.10000000 sec
TD0 1
SFO1 400.1328009 MHz
NUC1 1H
P0 3.79 usec
P1 11.38 usec
PLW1 7.41450024 W
F2 - Processing parameters
SI 65536
SF 400.1299990 MHz
WDW EM
SSB 0
LB 0.30 Hz
GB 0
PC 1.00
8.738
8.718
7.529
7.500
4.173
3.927
3.712
F
O_2N
CO_2Me
CO_2Me
11
14 13 12 11 10 9 8 7 6 5 4 3 2 1 0 ppm
1.00
1.26
2.06
3.04
3.18

$^{13}C\{1H\}$

```
Current Data Parameters
NAME                 pg110
EXPNO                    2
PROCNO                   1

F2 - Acquisition Parameters
Date_             20240729
Time                 12.27 h
INSTRUM              spect
PROBHD    Z149000_0038 (
PULPROG            zgpg30
TD                   48074
SOLVENT               MeOD
NS                    1024
DS                       4
SWH              24038.461 Hz
FIDRES            1.000061 Hz
AQ               0.9999392 sec
RG                   48.92
DW                  20.800 usec
DE                   18.00 usec
TE                   298.1 K
D1              0.25000000 sec
D11             0.03000000 sec
TD0                      1
SFO1           100.6238359 MHz
NUC1                   13C
P0                    3.16 usec
P1                    9.49 usec
PLW1           41.29999924 W
SFO2           400.1316005 MHz
NUC2                    1H
CPDPRG[2           waltz65
PCPD2                80.00 usec
PLW2            7.41450024 W
PLW12           0.15003000 W
PLW13           0.07534500 W

F2 - Processing parameters
SI                   65536
SF             100.6126278 MHz
WDW                     EM
SSB                      0
LB                    1.00 Hz
GB                       0
PC                    1.40
```

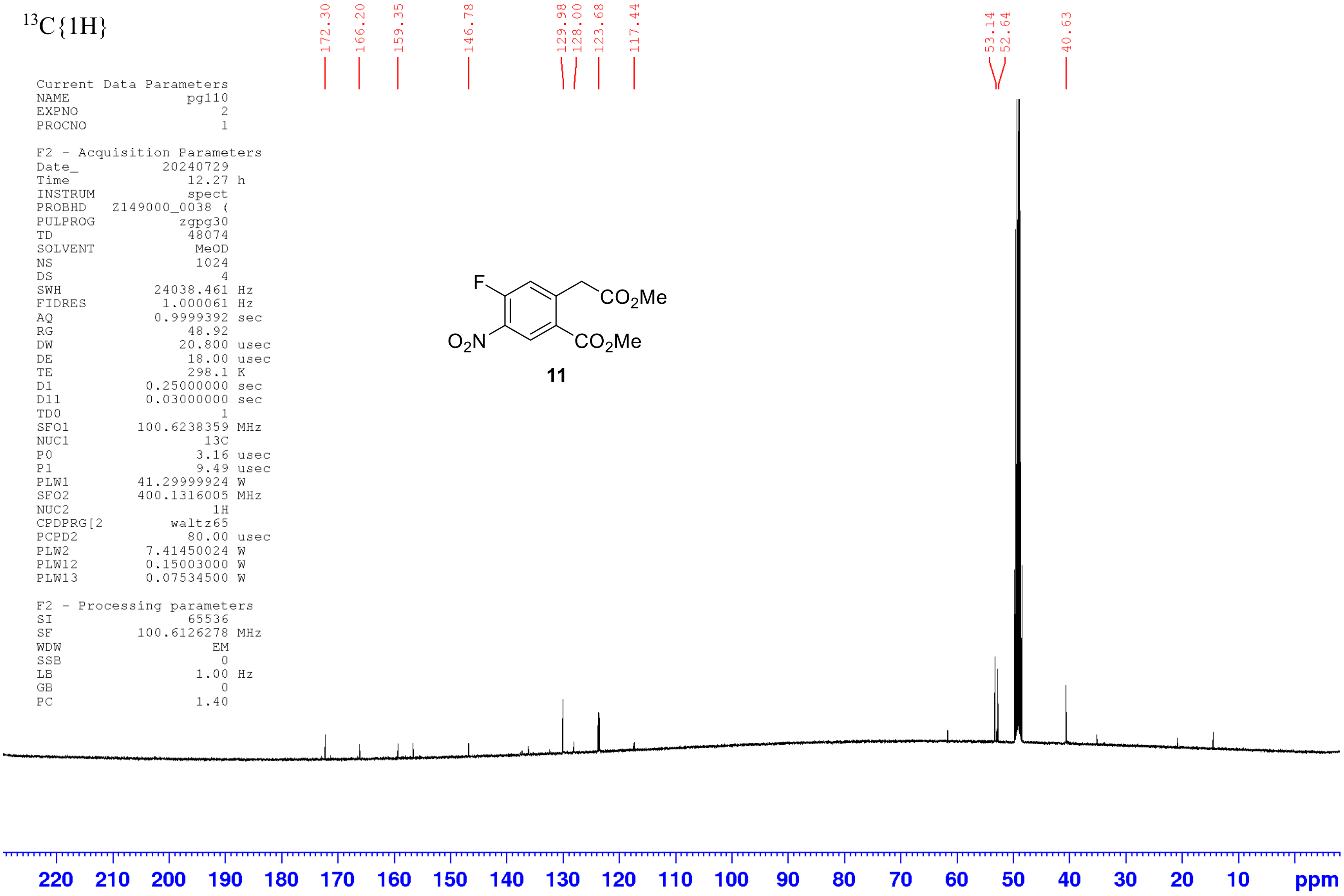

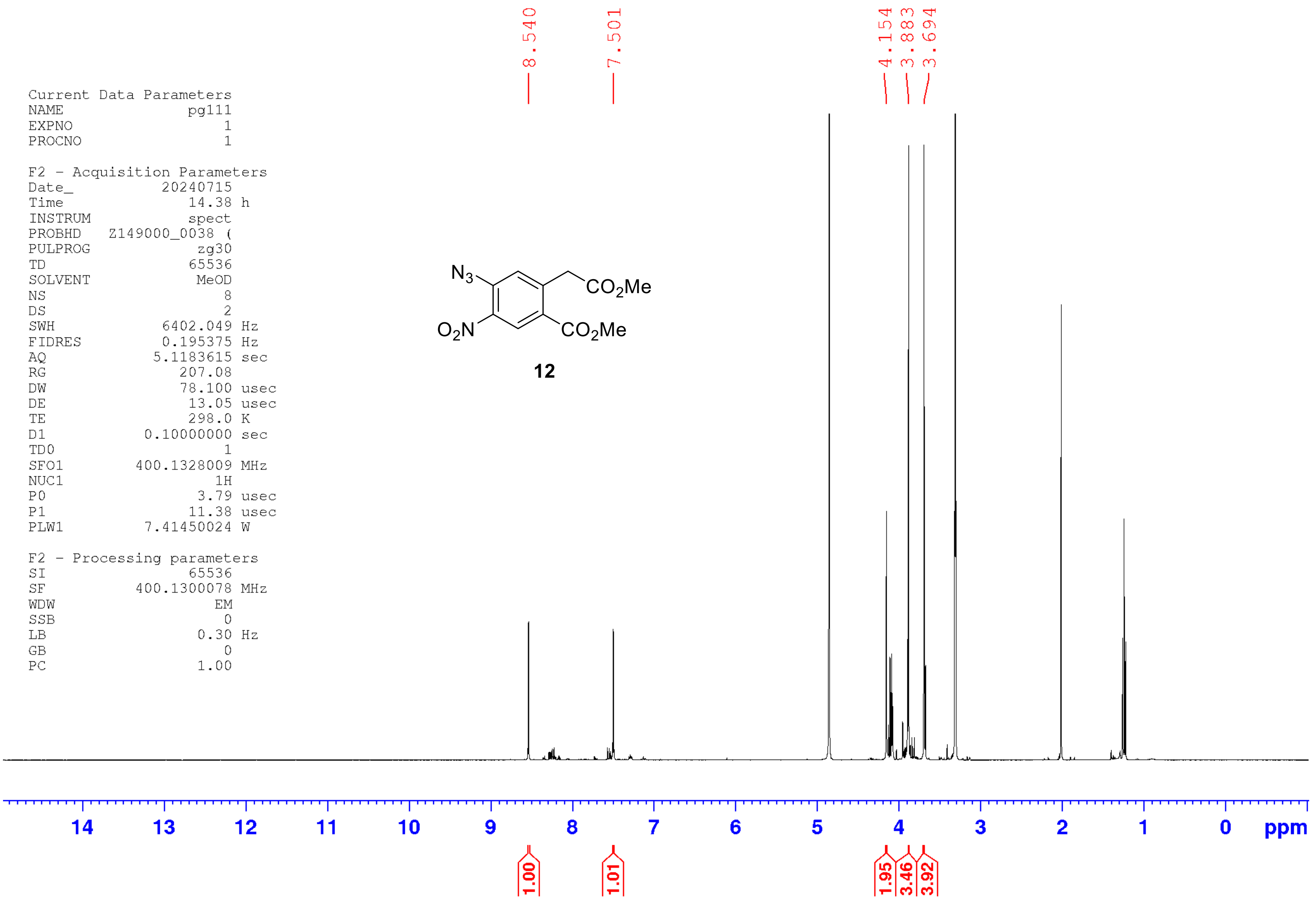
Current Data Parameters
NAME pg111
EXPNO 1
PROCNO 1
F2 - Acquisition Parameters
Date_ 20240715
Time 14.38 h
INSTRUM spect
PROBHD Z149000_0038 (
PULPROG zg30
TD 65536
SOLVENT MeOD
NS 8
DS 2
SWH 6402.049 Hz
FIDRES 0.195375 Hz
AQ 5.1183615 sec
RG 207.08
DW 78.100 usec
DE 13.05 usec
TE 298.0 K
D1 0.10000000 sec
TD0 1
SFO1 400.1328009 MHz
NUC1 1H
P0 3.79 usec
P1 11.38 usec
PLW1 7.41450024 W
F2 - Processing parameters
SI 65536
SF 400.1300078 MHz
WDW EM
SSB 0
LB 0.30 Hz
GB 0
PC 1.00
8.540
7.501
4.154
3.883
3.694
N3
CO2Me
O2N
CO2Me
12
14 13 12 11 10 9 8 7 6 5 4 3 2 1 0 ppm
1.00
1.01
1.95
3.46
3.92

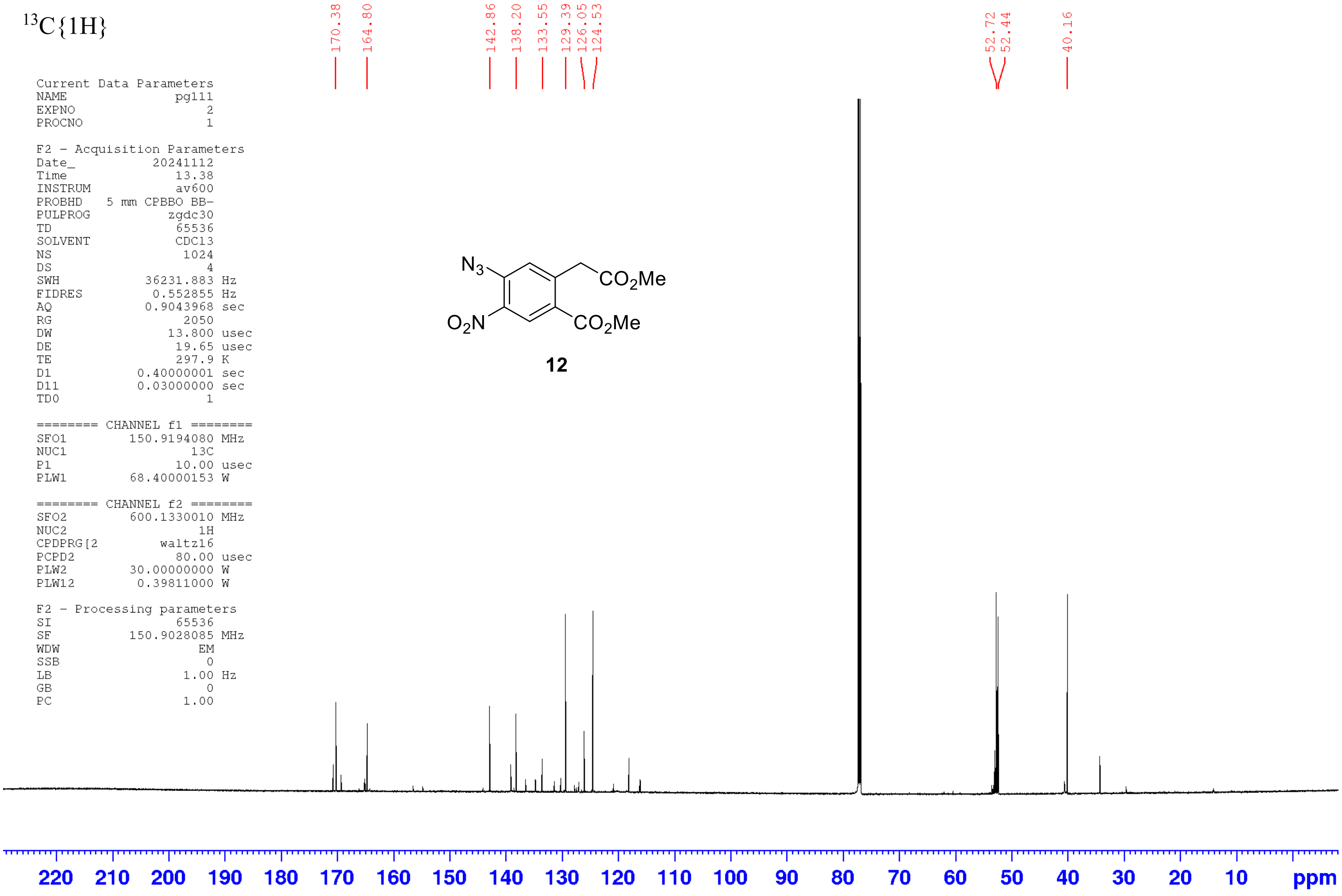
13C{1H}
Current Data Parameters
NAME pg111
EXPNO 2
PROCNO 1
F2 - Acquisition Parameters
Date_ 20241112
Time 13.38
INSTRUM av600
PROBHD 5 mm CPBBO BB-
PULPROG zgdc30
TD 65536
SOLVENT CDCl3
NS 1024
DS 4
SWH 36231.883 Hz
FIDRES 0.552855 Hz
AQ 0.9043968 sec
RG 2050
DW 13.800 usec
DE 19.65 usec
TE 297.9 K
D1 0.40000001 sec
D11 0.03000000 sec
TD0 1
======== CHANNEL f1 ========
SFO1 150.9194080 MHz
NUC1 13C
P1 10.00 usec
PLW1 68.40000153 W
======== CHANNEL f2 ========
SFO2 600.1330010 MHz
NUC2 1H
CPDPRG[2 waltz16
PCPD2 80.00 usec
PLW2 30.00000000 W
PLW12 0.39811000 W
F2 - Processing parameters
SI 65536
SF 150.9028085 MHz
WDW EM
SSB 0
LB 1.00 Hz
GB 0
PC 1.00
170.38
164.80
142.86
138.20
133.55
129.39
126.05
124.53
52.72
52.44
40.16
N3
CO2Me
O2N
CO2Me
12
220 210 200 190 180 170 160 150 140 130 120 110 100 90 80 70 60 50 40 30 20 10 ppm

```
Current Data Parameters
NAME                pg112
EXPNO                   1
PROCNO                  1

F2 - Acquisition Parameters
Date_            20240716
Time                16.33 h
INSTRUM             spect
PROBHD   Z149000_0038 (
PULPROG              zg30
TD                  65536
SOLVENT              MeOD
NS                      8
DS                      2
SWH              6402.049 Hz
FIDRES           0.195375 Hz
AQ              5.1183615 sec
RG                 207.08
DW                 78.100 usec
DE                  13.05 usec
TE                  298.0 K
D1             0.10000000 sec
TD0                     1
SFO1          400.1328009 MHz
NUC1                   1H
P0                   3.79 usec
P1                  11.38 usec
PLW1           7.41450024 W

F2 - Processing parameters
SI                  65536
SF            400.1299990 MHz
WDW                    EM
SSB                     0
LB                   0.30 Hz
GB                      0
PC                   1.00
```

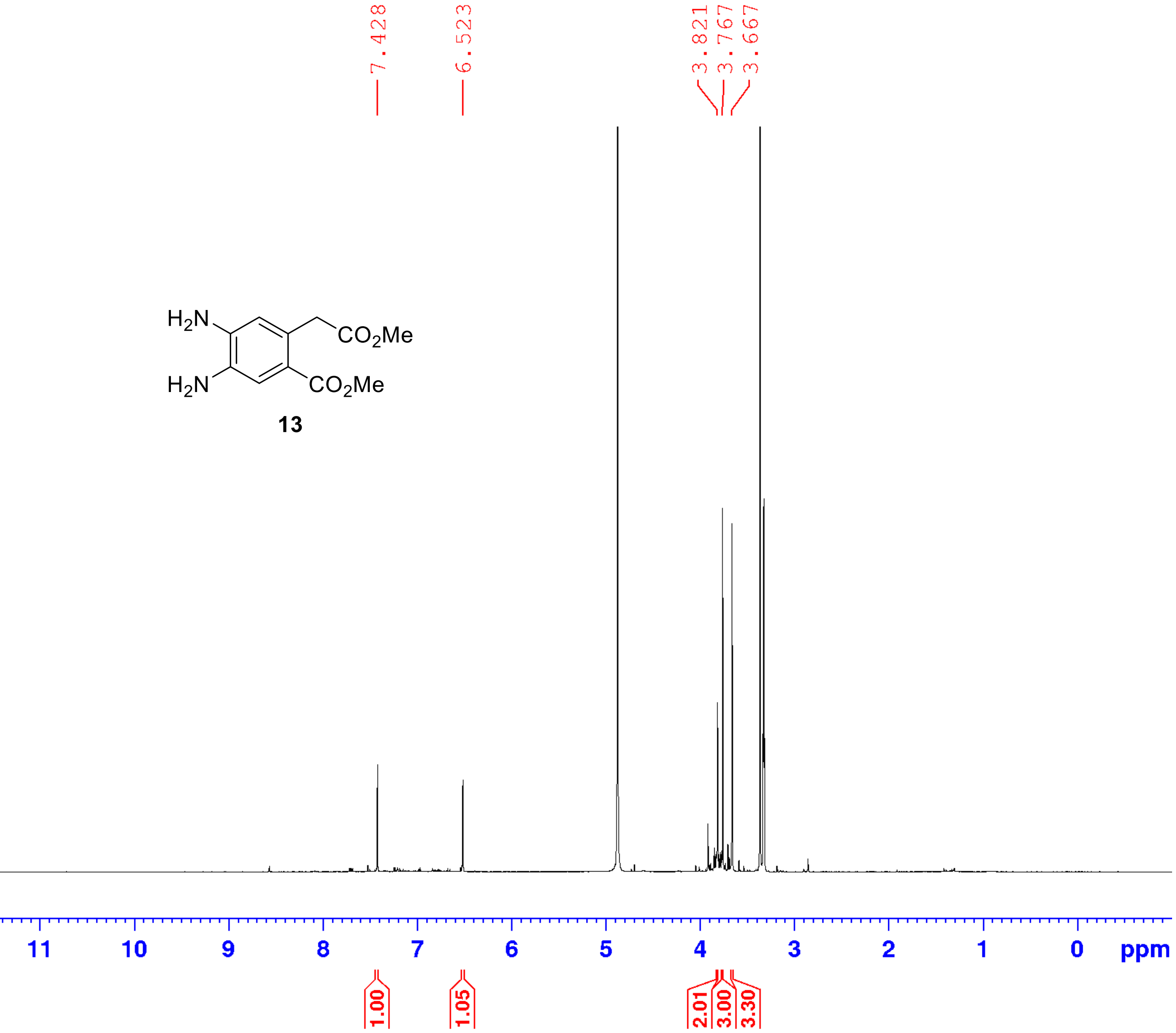

$^{13}C\{1H\}$

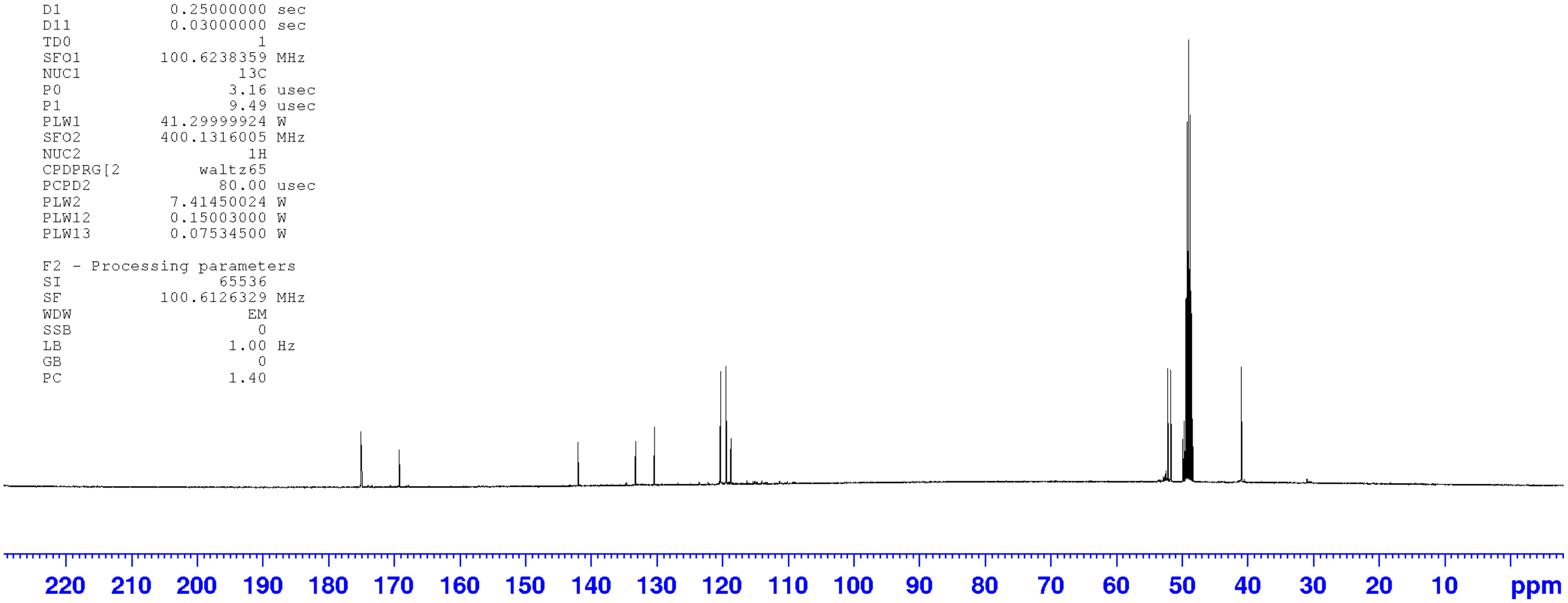


```
Current Data Parameters
NAME                 pg112
EXPNO                    2
PROCNO                   1

F2 - Acquisition Parameters
Date_             20240812
Time                 12.29 h
INSTRUM              spect
PROBHD    Z149000_0038 (
PULPROG            zgpg30
TD                   48074
SOLVENT               MeOD
NS                    1024
DS                       4
SWH              24038.461 Hz
FIDRES            1.000061 Hz
AQ               0.9999392 sec
RG                   48.92
DW                  20.800 usec
DE                   18.00 usec
TE                   298.1 K
D1              0.25000000 sec
D11             0.03000000 sec
TD0                      1
SFO1           100.6238359 MHz
NUC1                   13C
P0                    3.16 usec
P1                    9.49 usec
PLW1           41.29999924 W
SFO2           400.1316005 MHz
NUC2                    1H
CPDPRG[2           waltz65
PCPD2                80.00 usec
PLW2            7.41450024 W
PLW12           0.15003000 W
PLW13           0.07534500 W

F2 - Processing parameters
SI                   65536
SF             100.6126329 MHz
WDW                     EM
SSB                      0
LB                    1.00 Hz
GB                       0
PC                    1.40
```

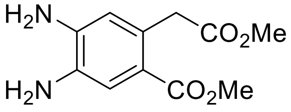

13

Current Data Parameters
NAME pg119
EXPNO 1
PROCNO 1

F2 - Acquisition Parameters
Date_ 20240930
Time 13.18 h
INSTRUM spect
PROBHD Z149000_0038 (
PULPROG zg30
TD 65536
SOLVENT MeOD
NS 8
DS 2
SWH 6402.049 Hz
FIDRES 0.195375 Hz
AQ 5.1183615 sec
RG 70.92
DW 78.100 usec
DE 13.05 usec
TE 298.1 K
D1 0.10000000 sec
TD0 1
SFO1 400.1328009 MHz
NUC1 1H
P0 3.79 usec
P1 11.38 usec
PLW1 7.41450024 W

F2 - Processing parameters
SI 65536
SF 400.1300074 MHz
WDW EM
SSB 0
LB 0.30 Hz
GB 0
PC 1.00

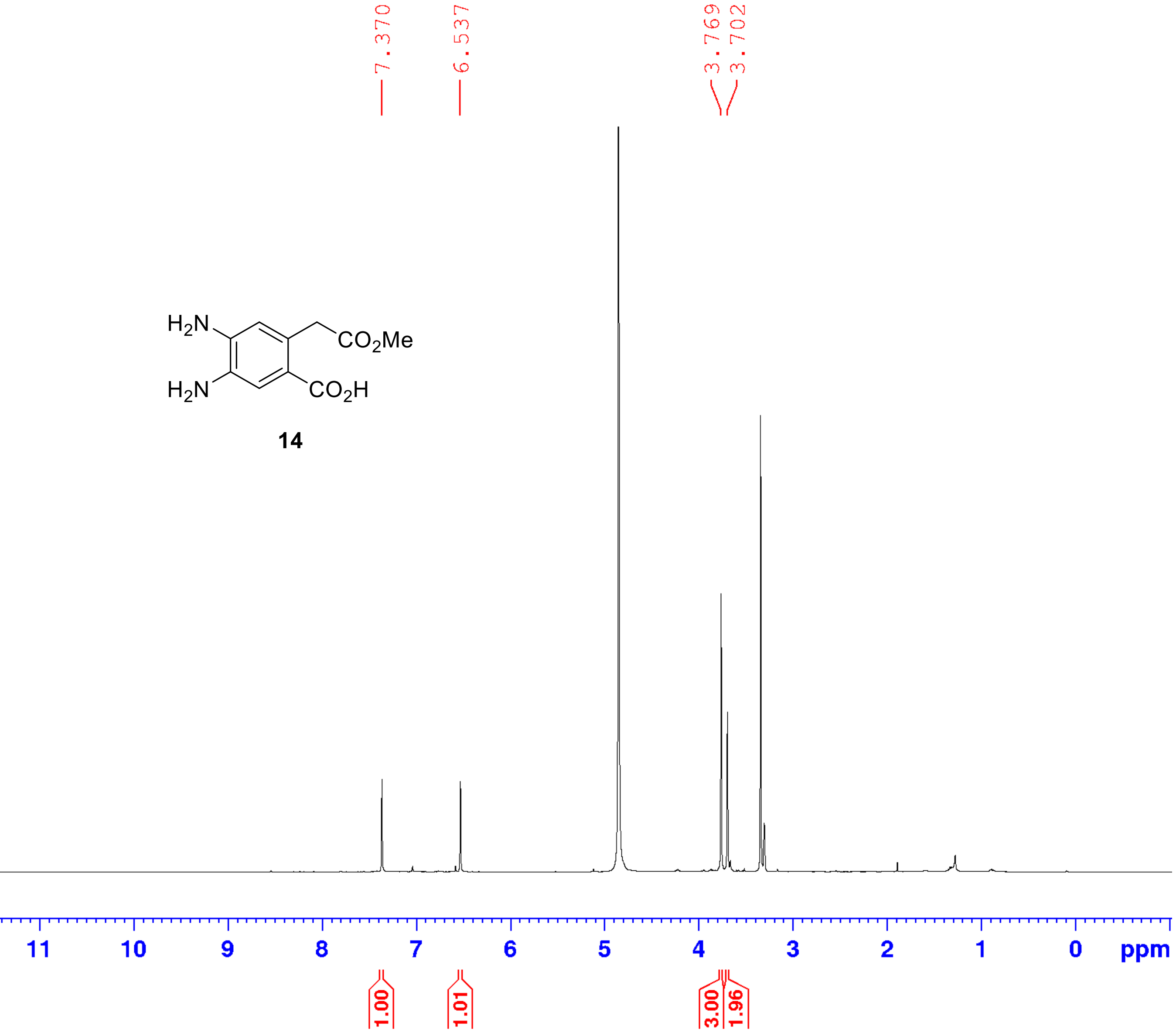

$^{13}C\{1H\}$

Current Data Parameters
NAME pg119
EXPNO 2
PROCNO 1

F2 - Acquisition Parameters
Date_ 20241107
Time 15.20
INSTRUM av600
PROBHD 5 mm CPBBO BB-
PULPROG zgdc30
TD 65536
SOLVENT D2O
NS 1016
DS 4
SWH 36231.883 Hz
FIDRES 0.552855 Hz
AQ 0.9043968 sec
RG 2050
DW 13.800 usec
DE 19.65 usec
TE 298.0 K
D1 0.40000001 sec
D11 0.03000000 sec
TD0 1

======== CHANNEL f1 ========
SFO1 150.9194080 MHz
NUC1 13C
P1 10.00 usec
PLW1 68.40000153 W

======== CHANNEL f2 ========
SFO2 600.1330010 MHz
NUC2 1H
CPDPRG[2 waltz16
PCPD2 80.00 usec
PLW2 30.00000000 W
PLW12 0.39811000 W

F2 - Processing parameters
SI 65536
SF 150.9028085 MHz
WDW EM
SSB 0
LB 1.00 Hz
GB 0
PC 1.00

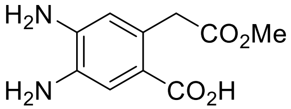

14

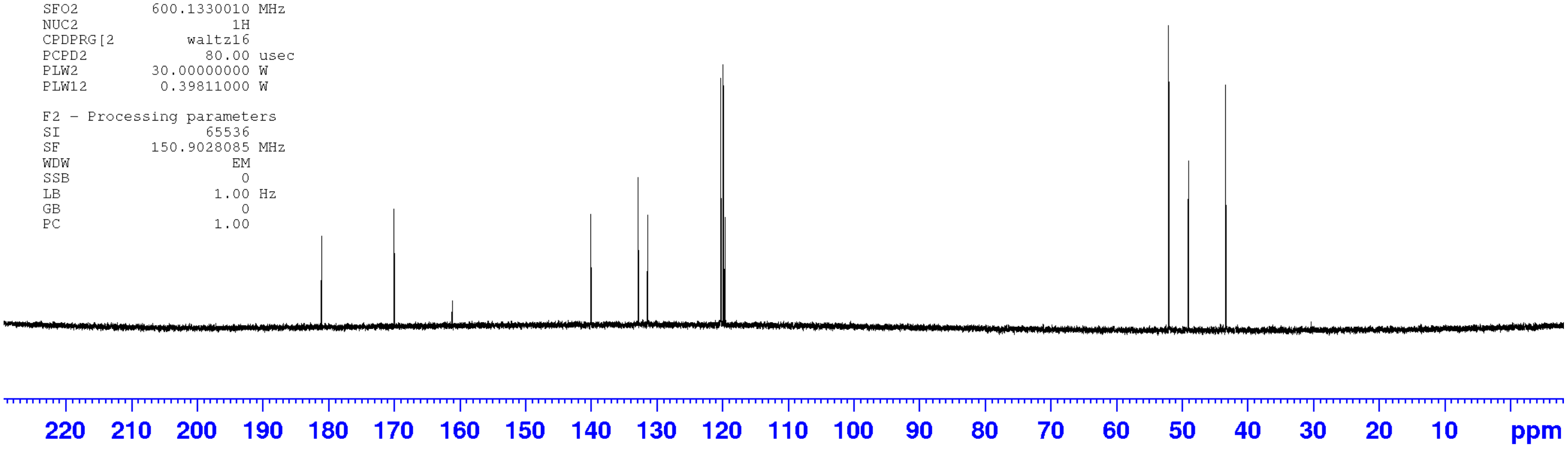

```
Current Data Parameters
NAME                 pg116
EXPNO                    1
PROCNO                   1

F2 - Acquisition Parameters
Date_             20250121
Time                 12.07
INSTRUM              av600
PROBHD    5 mm CPBBO BB-
PULPROG               zg30
TD                   98074
SOLVENT               DMSO
NS                       8
DS                       2
SWH               9615.385 Hz
FIDRES            0.098042 Hz
AQ               5.0998478 sec
RG                    80.6
DW                  52.000 usec
DE                   53.12 usec
TE                   298.0 K
D1              0.10000000 sec
TD0                      1

======== CHANNEL f1 ========
SFO1          600.1342009 MHz
NUC1                    1H
P1                   10.00 usec
PLW1          30.00000000 W

F2 - Processing parameters
SI                   65536
SF            600.1300132 MHz
WDW                     EM
SSB                      0
LB                    0.30 Hz
GB                       0
PC                    1.00
```

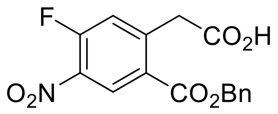

15

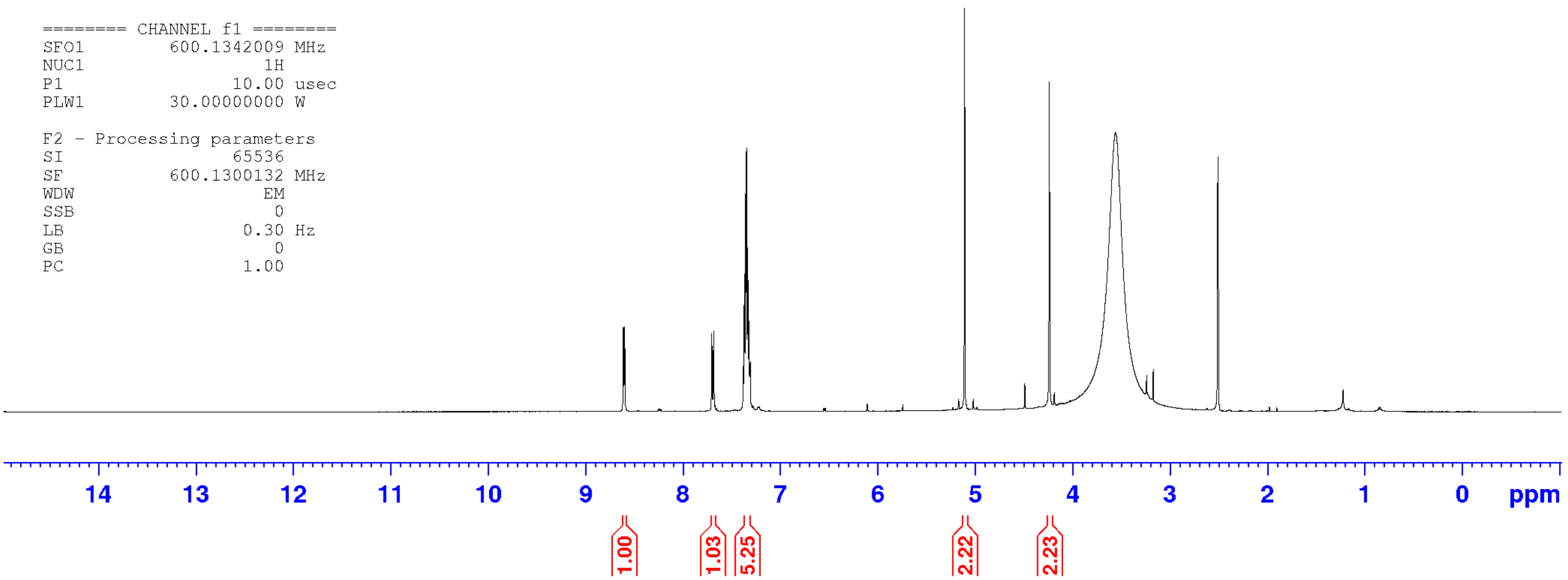

$^{13}C\{1H\}$

Current Data Parameters
NAME pg116
EXPNO 2
PROCNO 1

F2 - Acquisition Parameters
Date_ 20250121
Time 12.34
INSTRUM av600
PROBHD 5 mm CPBBO BB-
PULPROG zgdc30
TD 65536
SOLVENT DMSO
NS 1024
DS 4
SWH 36231.883 Hz
FIDRES 0.552855 Hz
AQ 0.9043968 sec
RG 2050
DW 13.800 usec
DE 19.65 usec
TE 298.0 K
D1 0.40000001 sec
D11 0.03000000 sec
TD0 1

======== CHANNEL f1 ========
SFO1 150.9194080 MHz
NUC1 13C
P1 10.00 usec
PLW1 68.40000153 W

======== CHANNEL f2 ========
SFO2 600.1330010 MHz
NUC2 1H
CPDPRG[2 waltz16
PCPD2 80.00 usec
PLW2 30.00000000 W
PLW12 0.39811000 W

F2 - Processing parameters
SI 65536
SF 150.9028682 MHz
WDW EM
SSB 0
LB 1.00 Hz
GB 0
PC 1.00

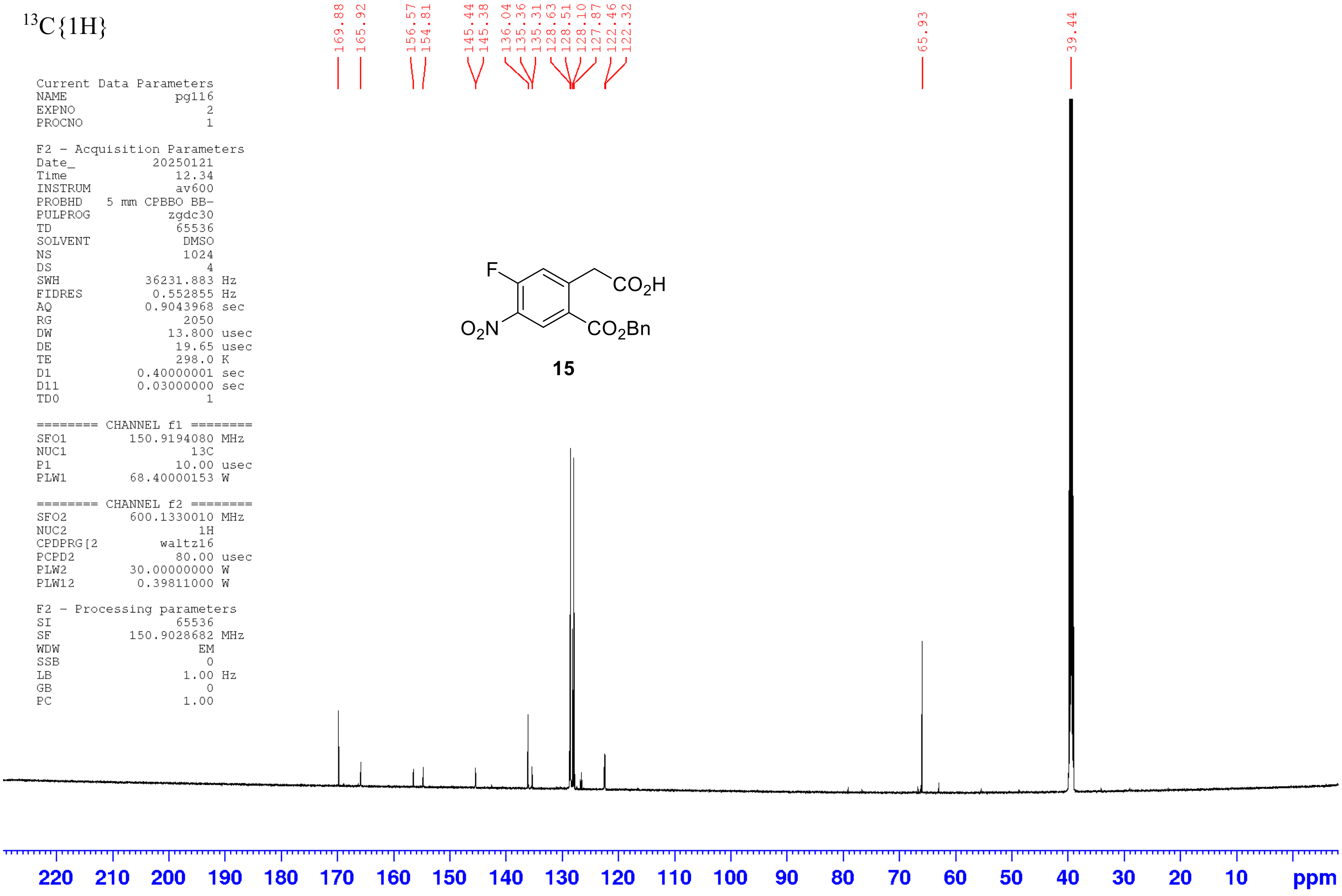

```
Current Data Parameters
NAME                 pg117
EXPNO                    1
PROCNO                   1

F2 - Acquisition Parameters
Date_             20241211
Time                 14.36
INSTRUM              gn500
PROBHD    5 mm broadband
PULPROG               zg30
TD                   81728
SOLVENT              CD3OD
NS                       8
DS                       2
SWH               8012.820 Hz
FIDRES            0.098043 Hz
AQ               5.0998273 sec
RG                  1149.4
DW                  62.400 usec
DE                    6.00 usec
TE                   298.0 K
D1              0.10000000 sec
MCREST                   0 sec
MCWRK           0.01500000 sec

======== CHANNEL f1 ========
NUC1                    1H
P1                   12.00 usec
PL1                  -6.00 dB
SFO1           498.4534891 MHz

F2 - Processing parameters
SI                   65536
SF             498.4500176 MHz
WDW                     EM
SSB                      0
LB                    0.30 Hz
GB                       0
PC                    1.00
```

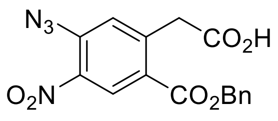

16

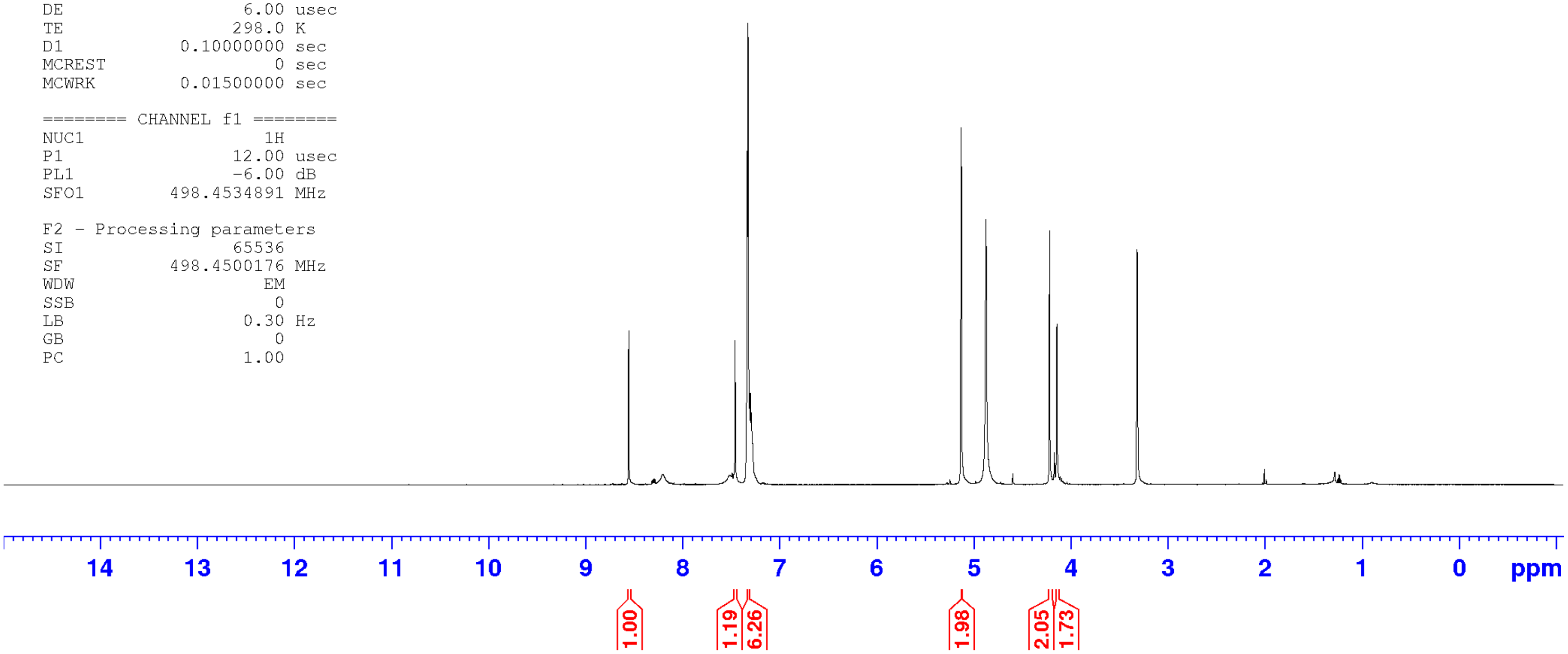

$^{13}C\{1H\}$

```
Current Data Parameters
NAME                pg117
EXPNO                   2
PROCNO                  1

F2 - Acquisition Parameters
Date_            20241207
Time                15.22
INSTRUM             av600
PROBHD   5 mm CPBBO BB-
PULPROG           zgdc30
TD                  65536
SOLVENT              CD3OD
NS                   1024
DS                      4
SWH            36231.883 Hz
FIDRES          0.552855 Hz
AQ             0.9043968 sec
RG                   2050
DW                 13.800 usec
DE                  19.65 usec
TE                  297.9 K
D1             0.40000001 sec
D11            0.03000000 sec
TD0                     1

======== CHANNEL f1 ========
SFO1         150.9194080 MHz
NUC1                  13C
P1                  10.00 usec
PLW1          68.40000153 W

======== CHANNEL f2 ========
SFO2         600.1330010 MHz
NUC2                   1H
CPDPRG[2          waltz16
PCPD2               80.00 usec
PLW2          30.00000000 W
PLW12          0.39811000 W

F2 - Processing parameters
SI                  65536
SF           150.9026017 MHz
WDW                    EM
SSB                     0
LB                   1.00 Hz
GB                      0
PC                   1.00
```

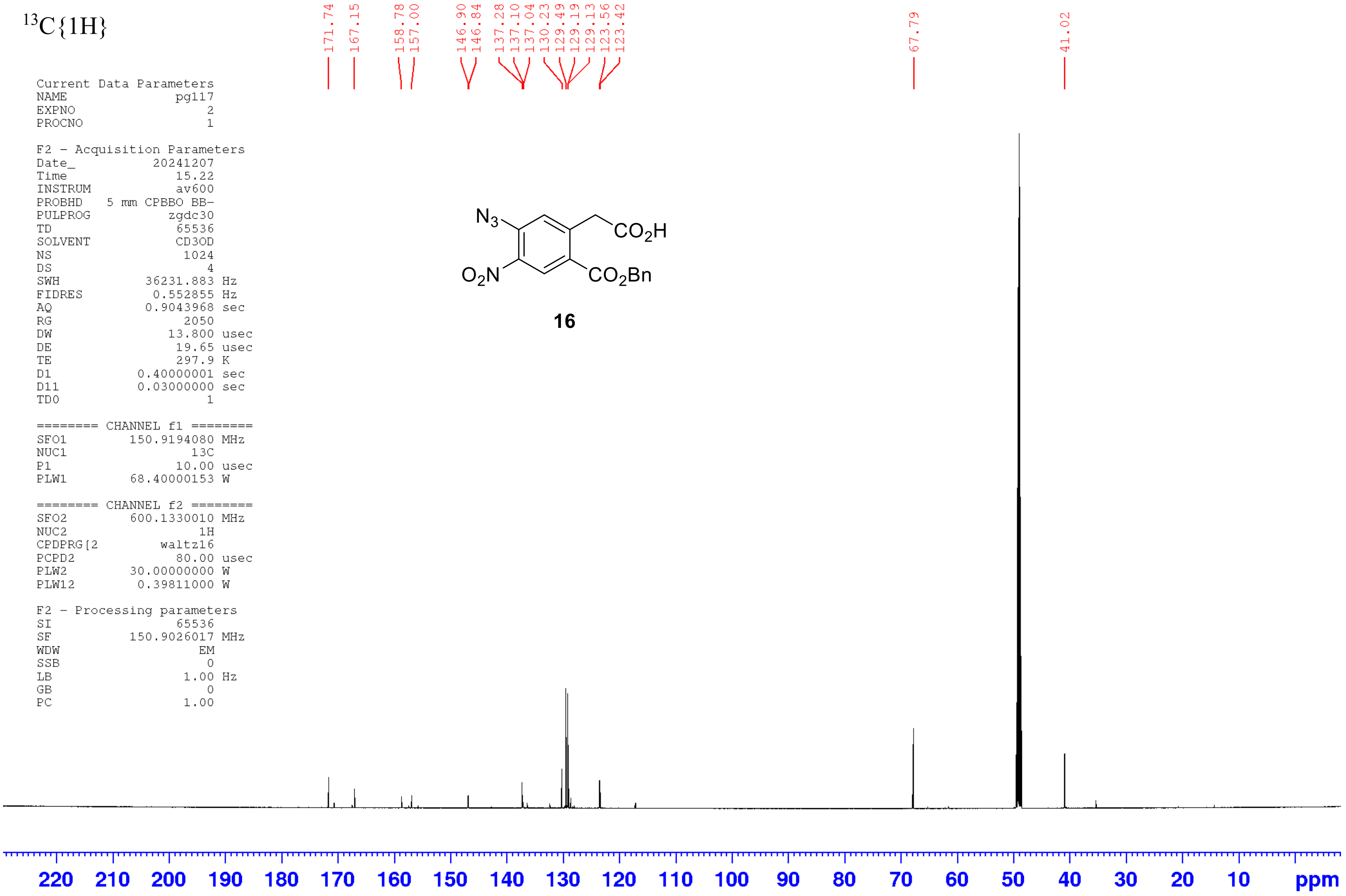

16

Current Data Parameters
NAME pg120
EXPNO 1
PROCNO 1

F2 - Acquisition Parameters
Date_ 20250124
Time 12.12
INSTRUM gn500
PROBHD 5 mm broadband
PULPROG zg30
TD 81728
SOLVENT D2O
NS 8
DS 2
SWH 8012.820 Hz
FIDRES 0.098043 Hz
AQ 5.0998273 sec
RG 1290.2
DW 62.400 usec
DE 6.00 usec
TE 298.0 K
D1 0.10000000 sec
MCREST 0 sec
MCWRK 0.01500000 sec

======== CHANNEL f1 ========
NUC1 1H
P1 12.00 usec
PL1 -6.00 dB
SFO1 498.4534891 MHz

F2 - Processing parameters
SI 65536
SF 498.4500088 MHz
WDW EM
SSB 0
LB 0.30 Hz
GB 0
PC 1.00

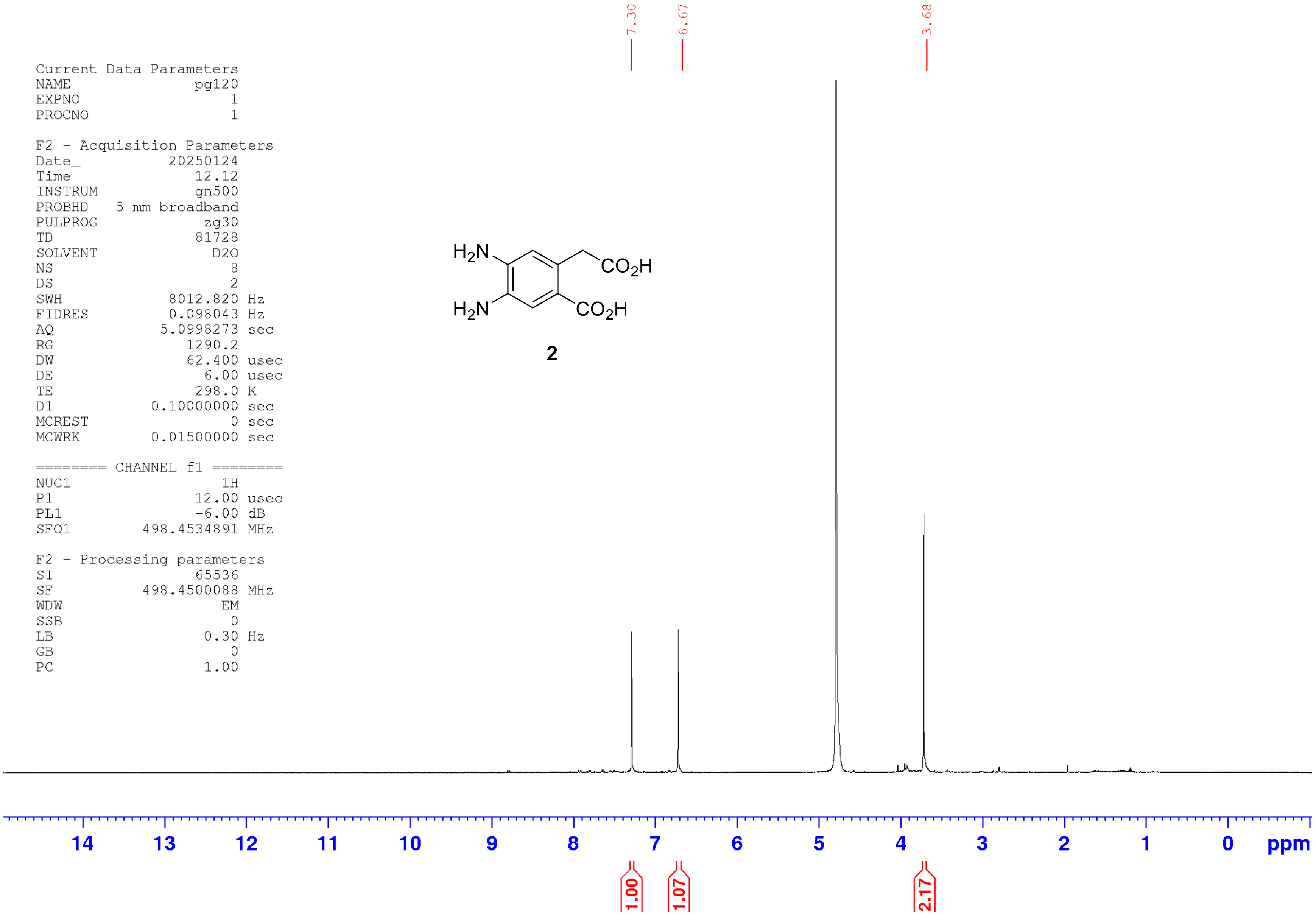

$^{13}C\{1H\}$

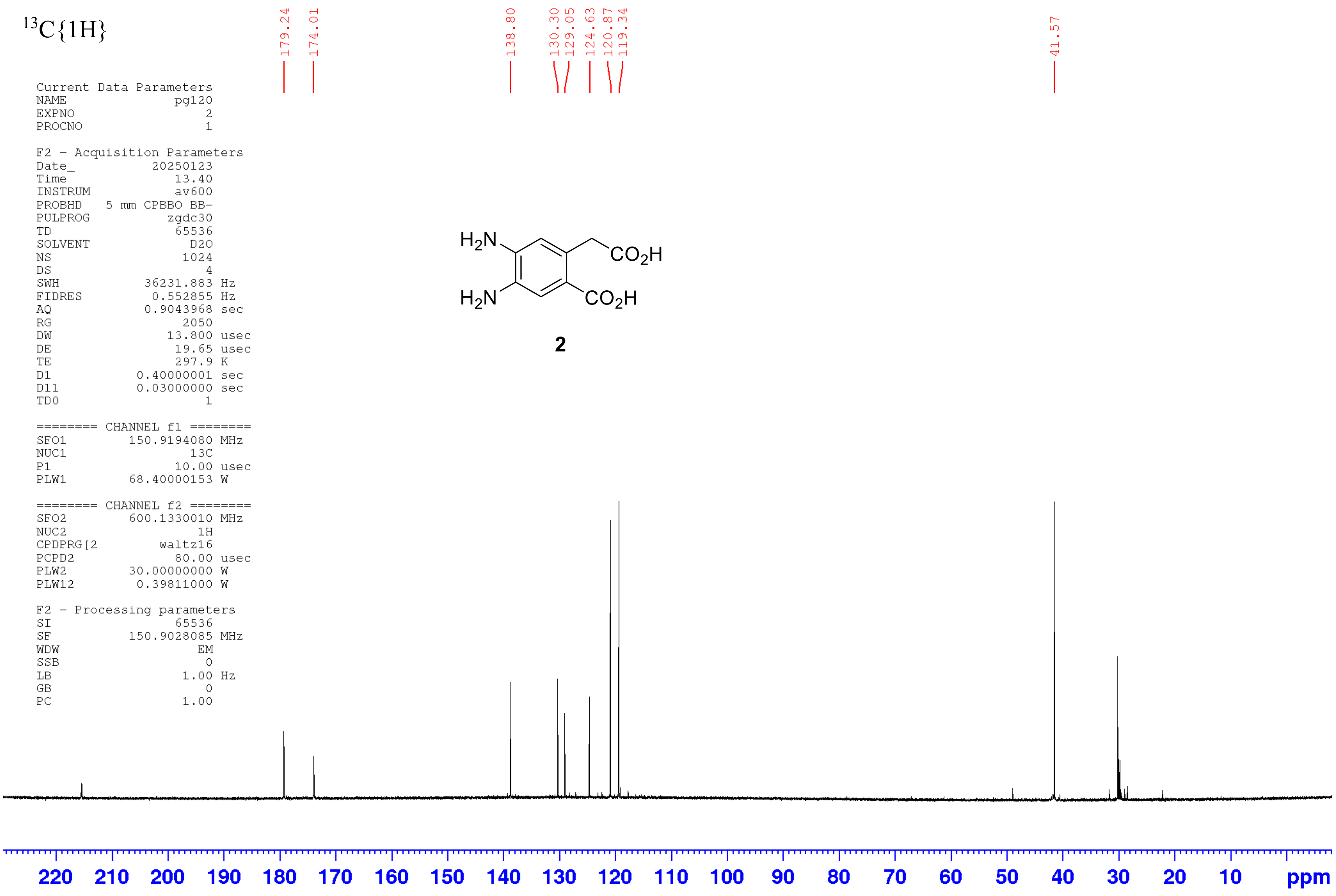
Current Data Parameters
NAME pg120
EXPNO 2
PROCNO 1
F2 - Acquisition Parameters
Date_ 20250123
Time 13.40
INSTRUM av600
PROBHD 5 mm CPBBO BB-
PULPROG zgdc30
TD 65536
SOLVENT D2O
NS 1024
DS 4
SWH 36231.883 Hz
FIDRES 0.552855 Hz
AQ 0.9043968 sec
RG 2050
DW 13.800 usec
DE 19.65 usec
TE 297.9 K
D1 0.40000001 sec
D11 0.03000000 sec
TD0 1
======== CHANNEL f1 ========
SFO1 150.9194080 MHz
NUC1 13C
P1 10.00 usec
PLW1 68.40000153 W
======== CHANNEL f2 ========
SFO2 600.1330010 MHz
NUC2 1H
CPDPRG[2 waltz16
PCPD2 80.00 usec
PLW2 30.00000000 W
PLW12 0.39811000 W
F2 - Processing parameters
SI 65536
SF 150.9028085 MHz
WDW EM
SSB 0
LB 1.00 Hz
GB 0
PC 1.00
179.24
174.01
138.80
130.30
129.05
124.63
120.87
119.34
41.57
H2N
H2N
CO2H
CO2H
2
220 210 200 190 180 170 160 150 140 130 120 110 100 90 80 70 60 50 40 30 20 10 ppm

^{1}H NMR (400 MHz, ($CDCl_3$)

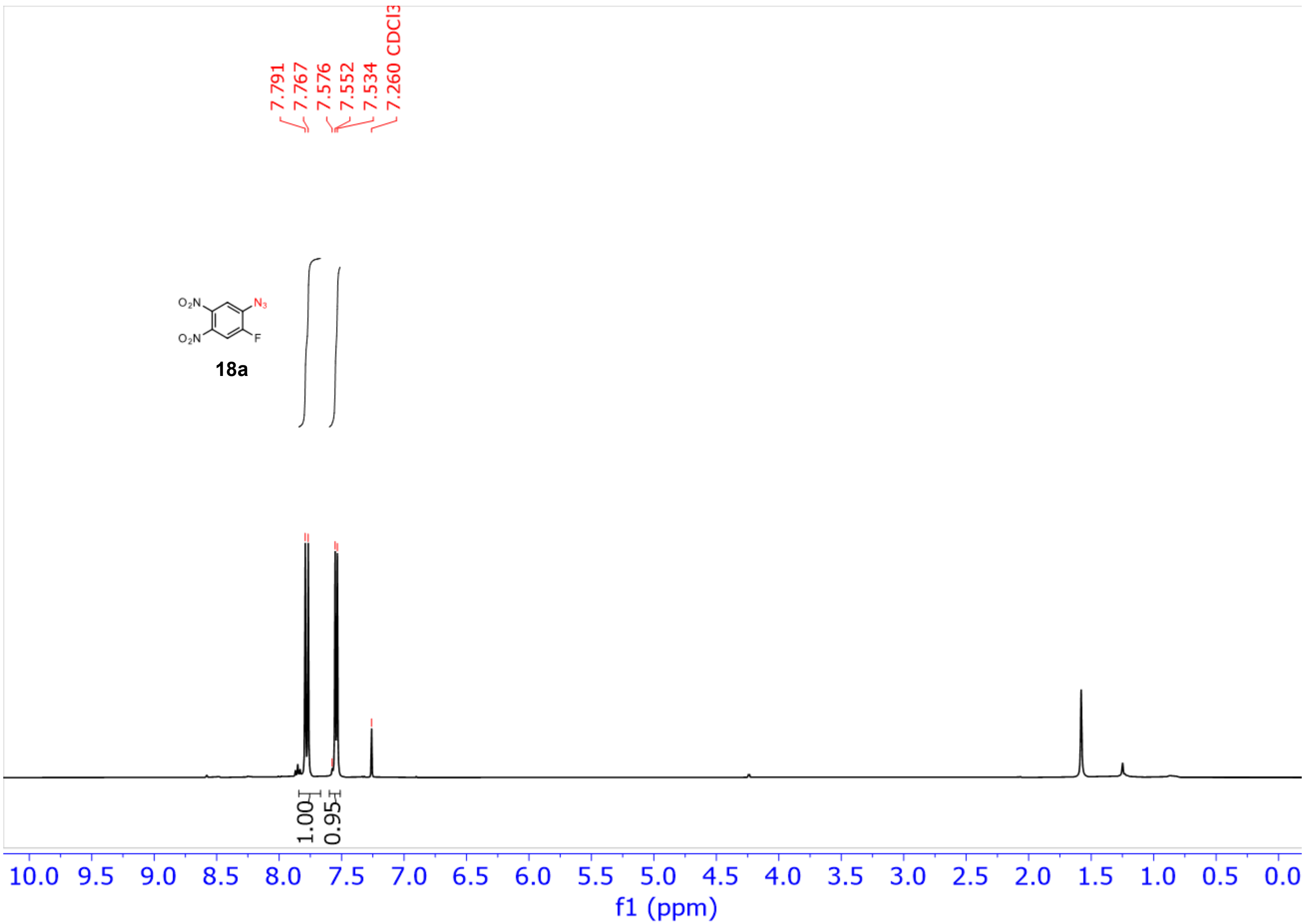

$^{13}C\{^1H\}$ NMR (100 MHz, $CDCl_3$)

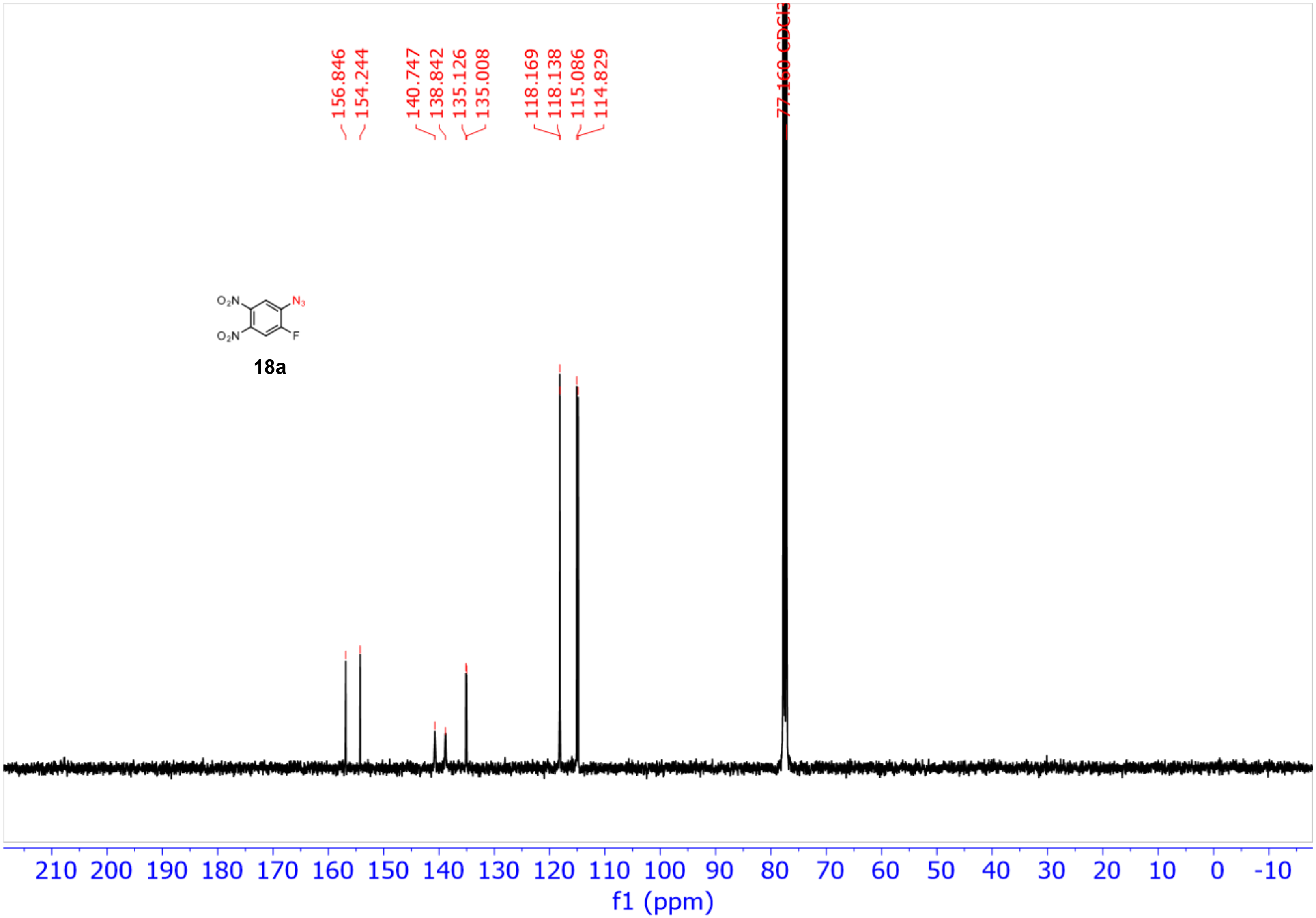

^1H NMR (400 MHz, CD_3OD)

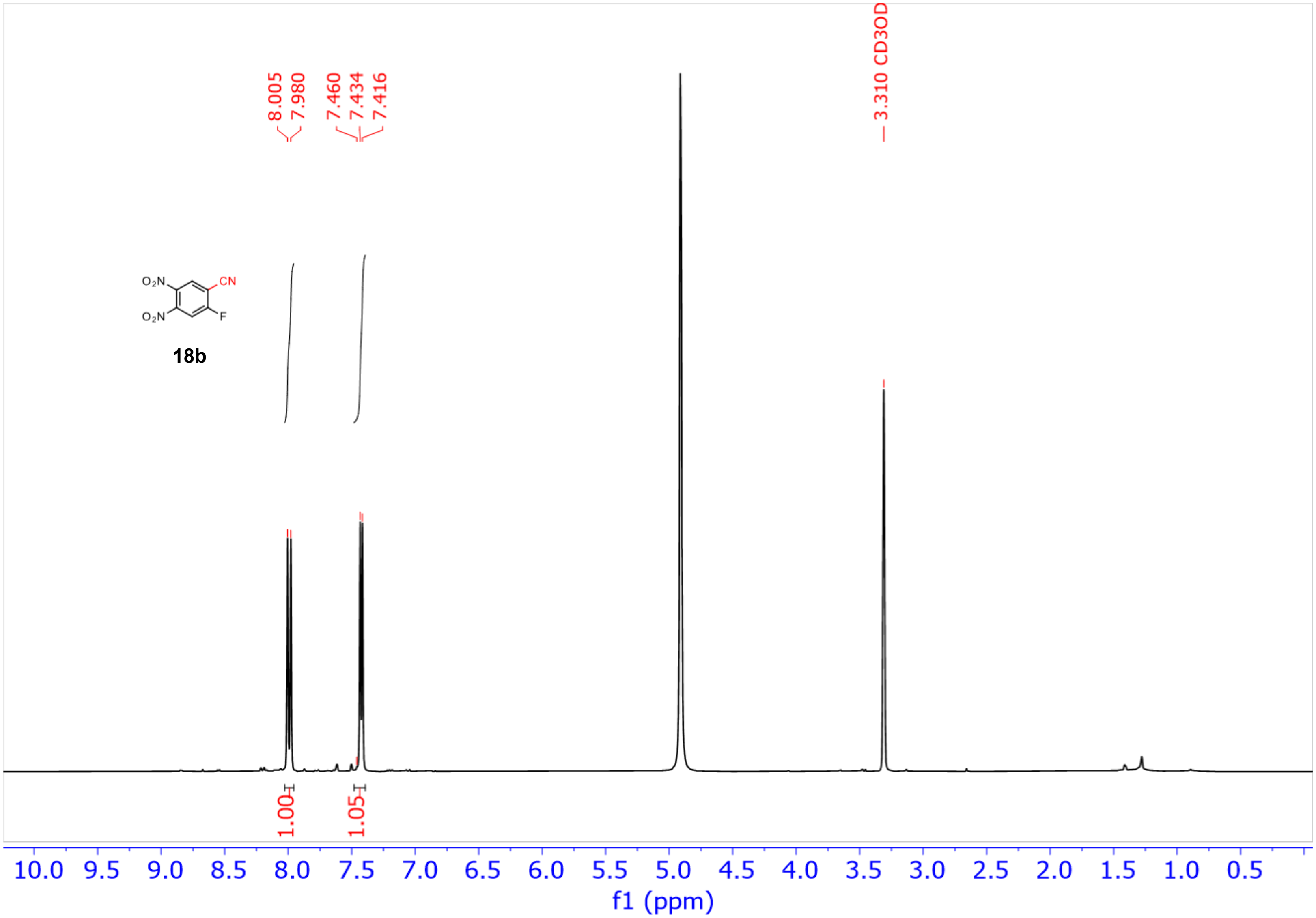

$^{13}C\{1H\}$ NMR (100 MHz, CD_3OD)

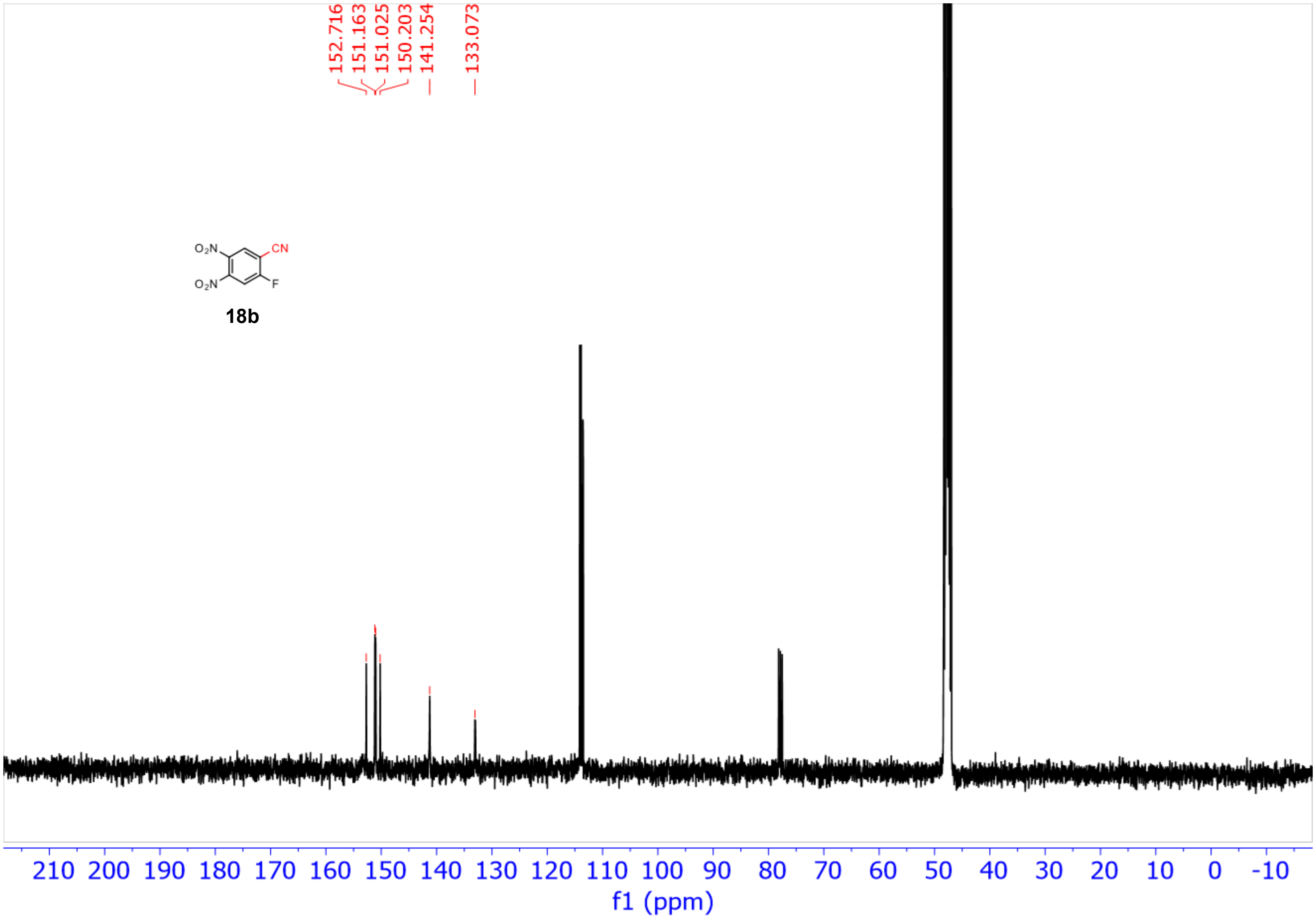

^1H NMR (400 MHz, $(CD_3)_2SO$)

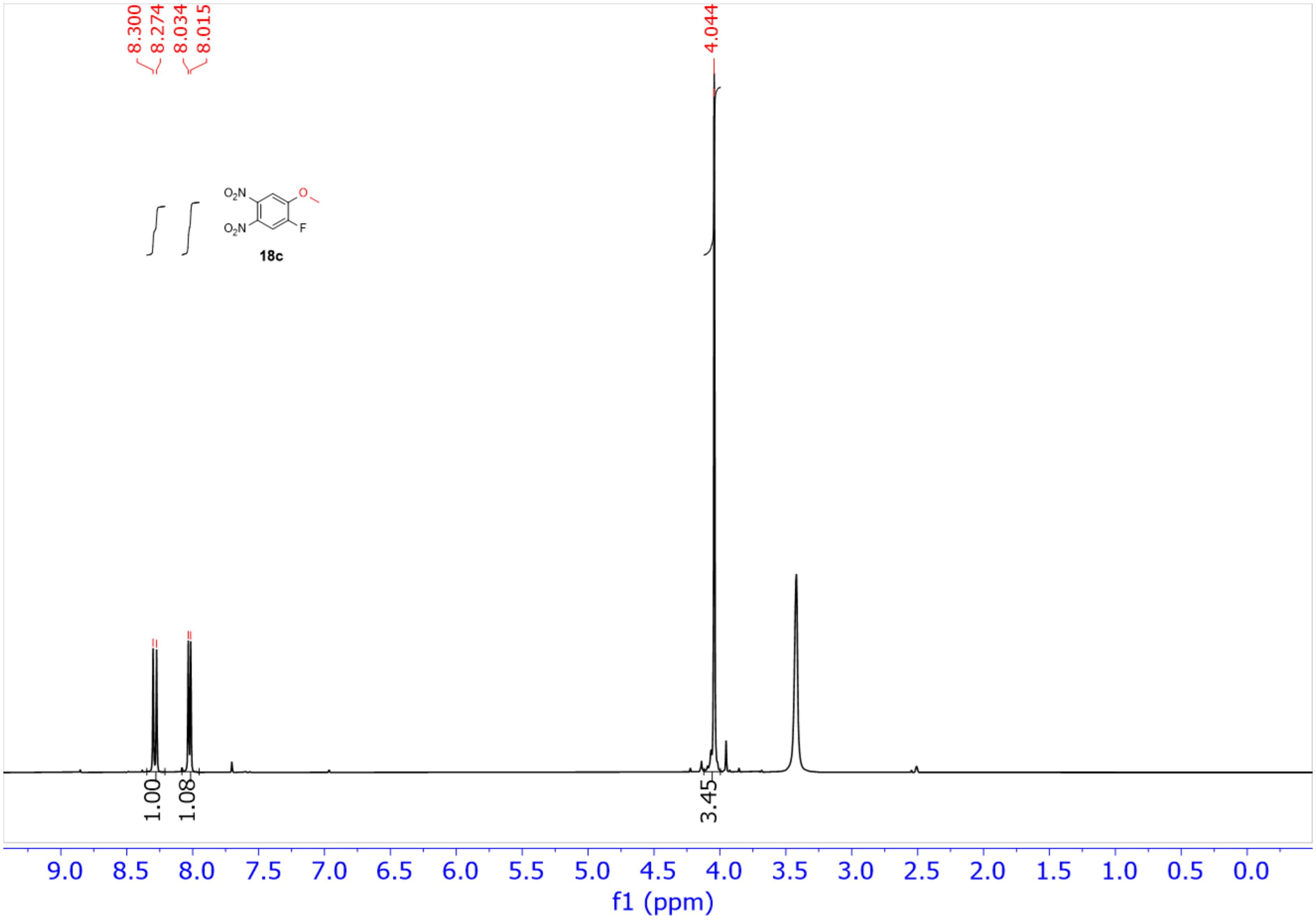

^{13}C {1H} NMR (100 MHz, $(CD_3)_2SO$)

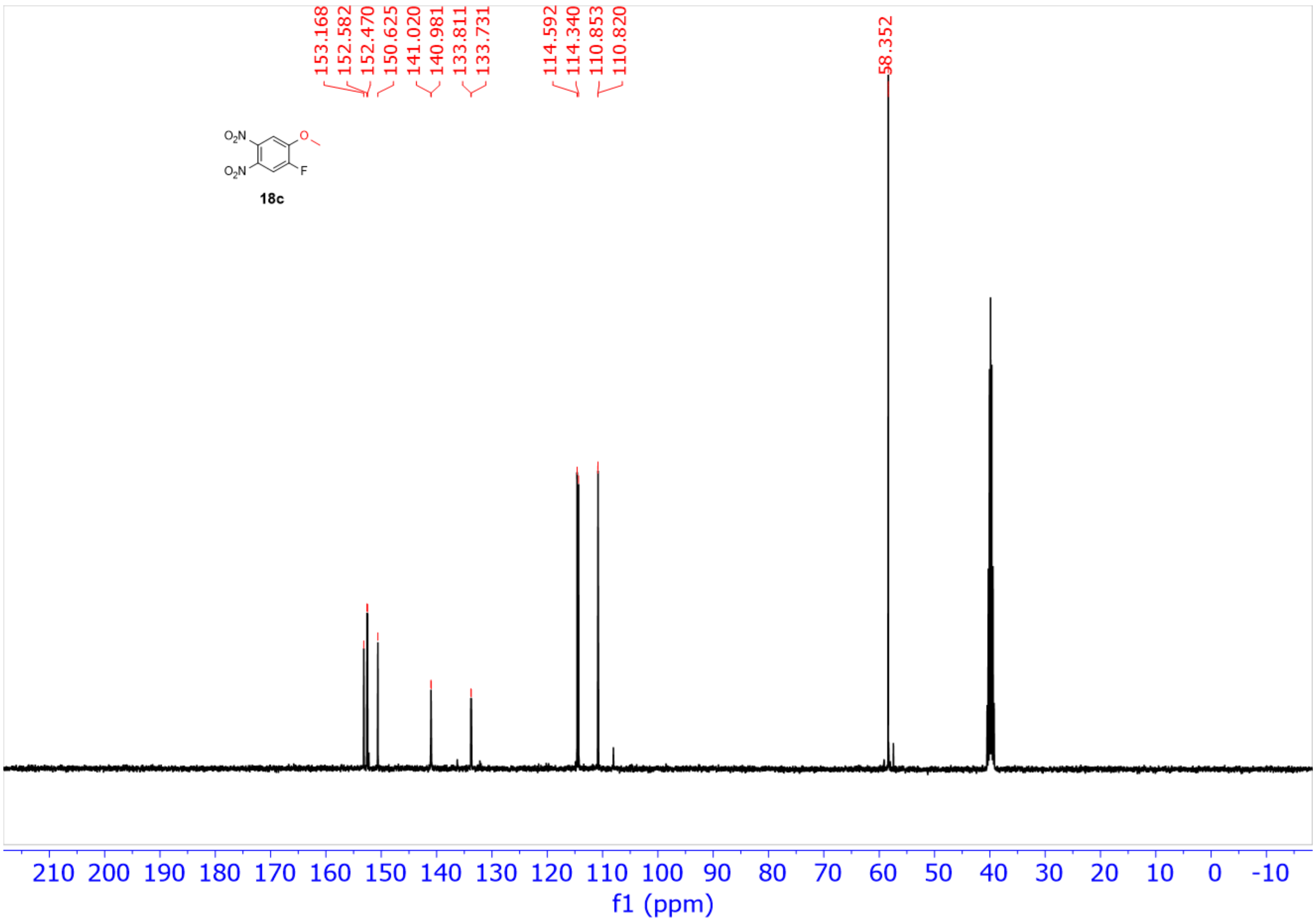

^{1}H NMR (400 MHz, $CDCl_3$)

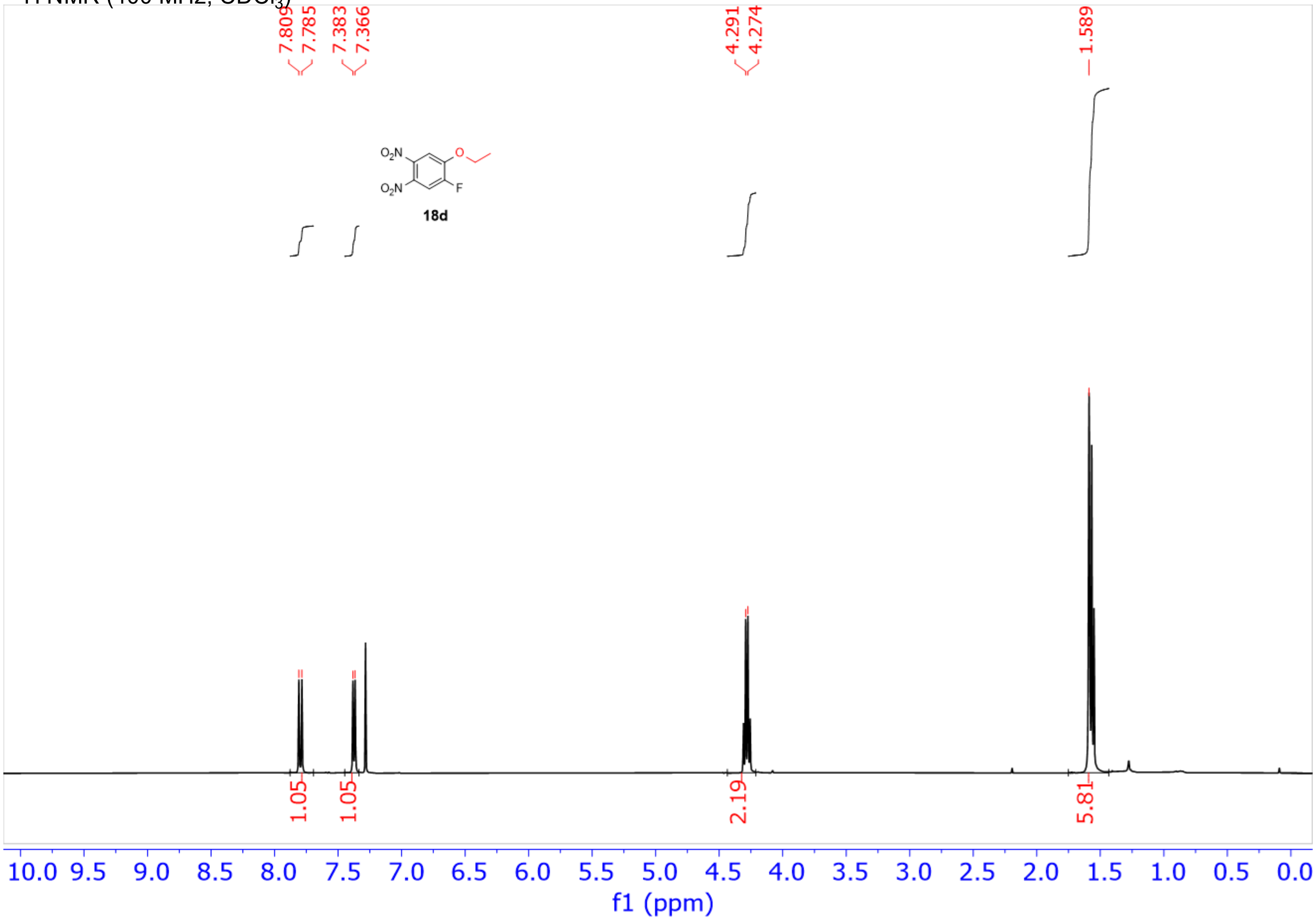

$^{13}C\{1H\}$ NMR (100 MHz, $CDCl_3$)

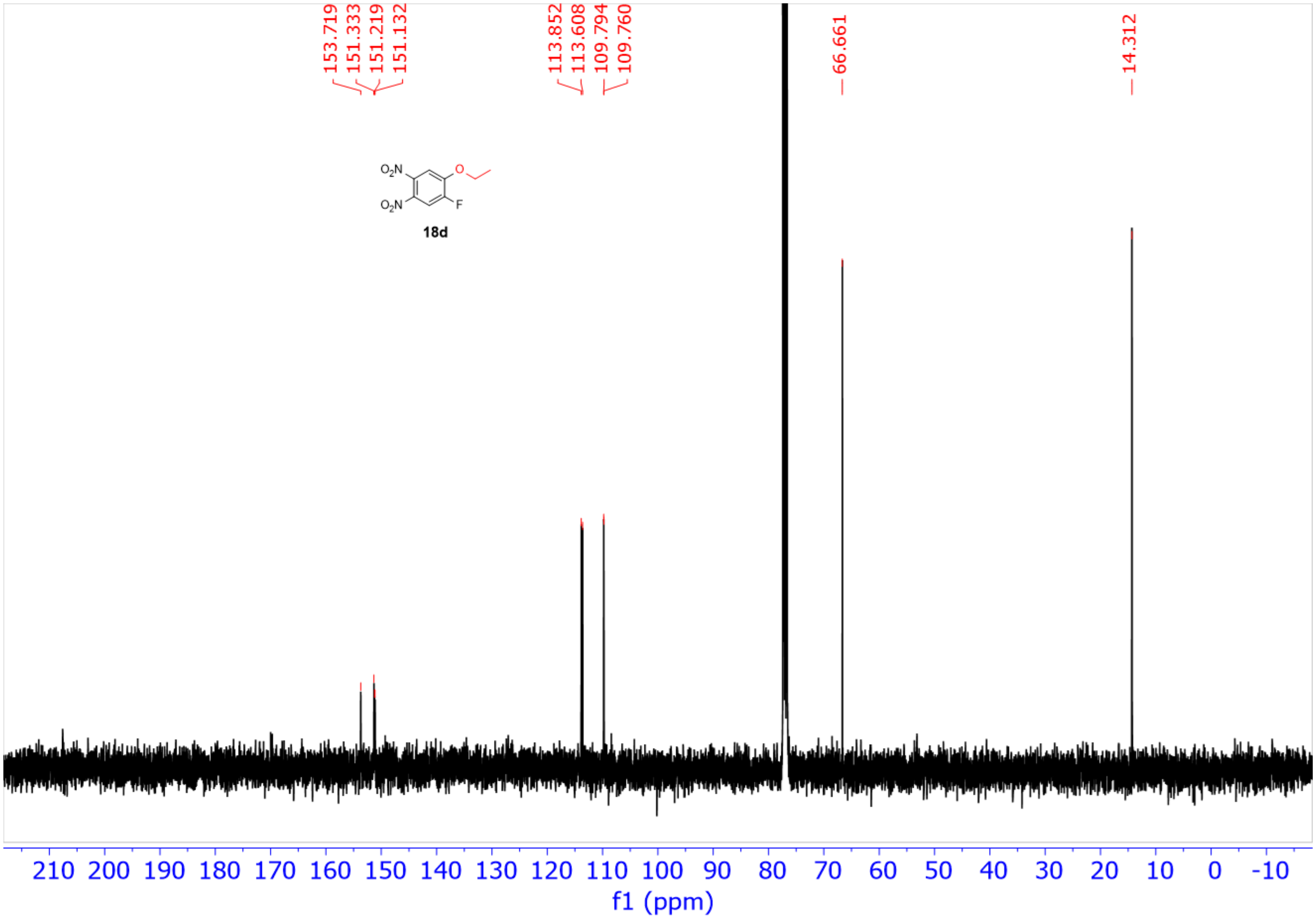

^{1}H NMR (400 MHz, $CDCl_3$)

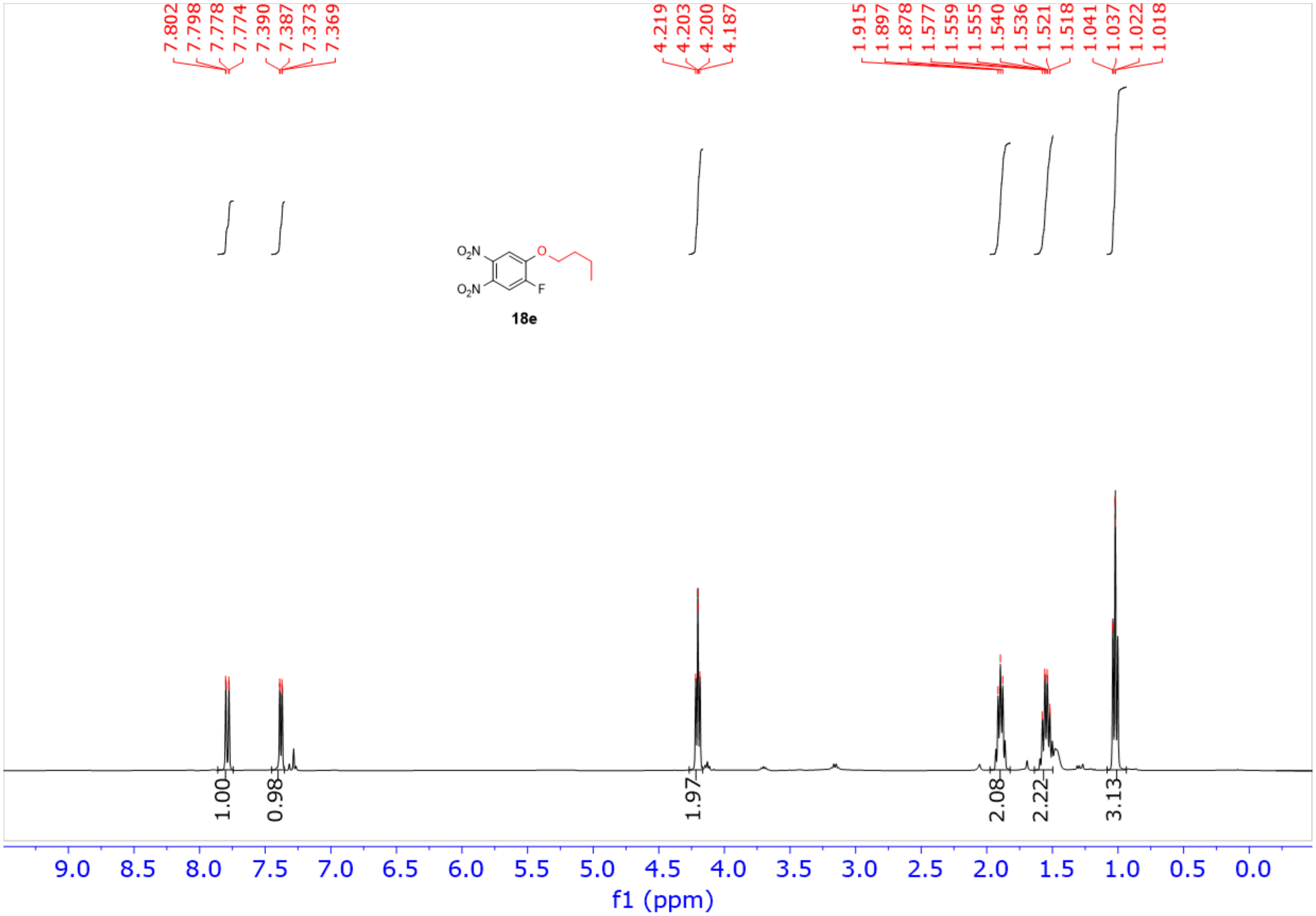

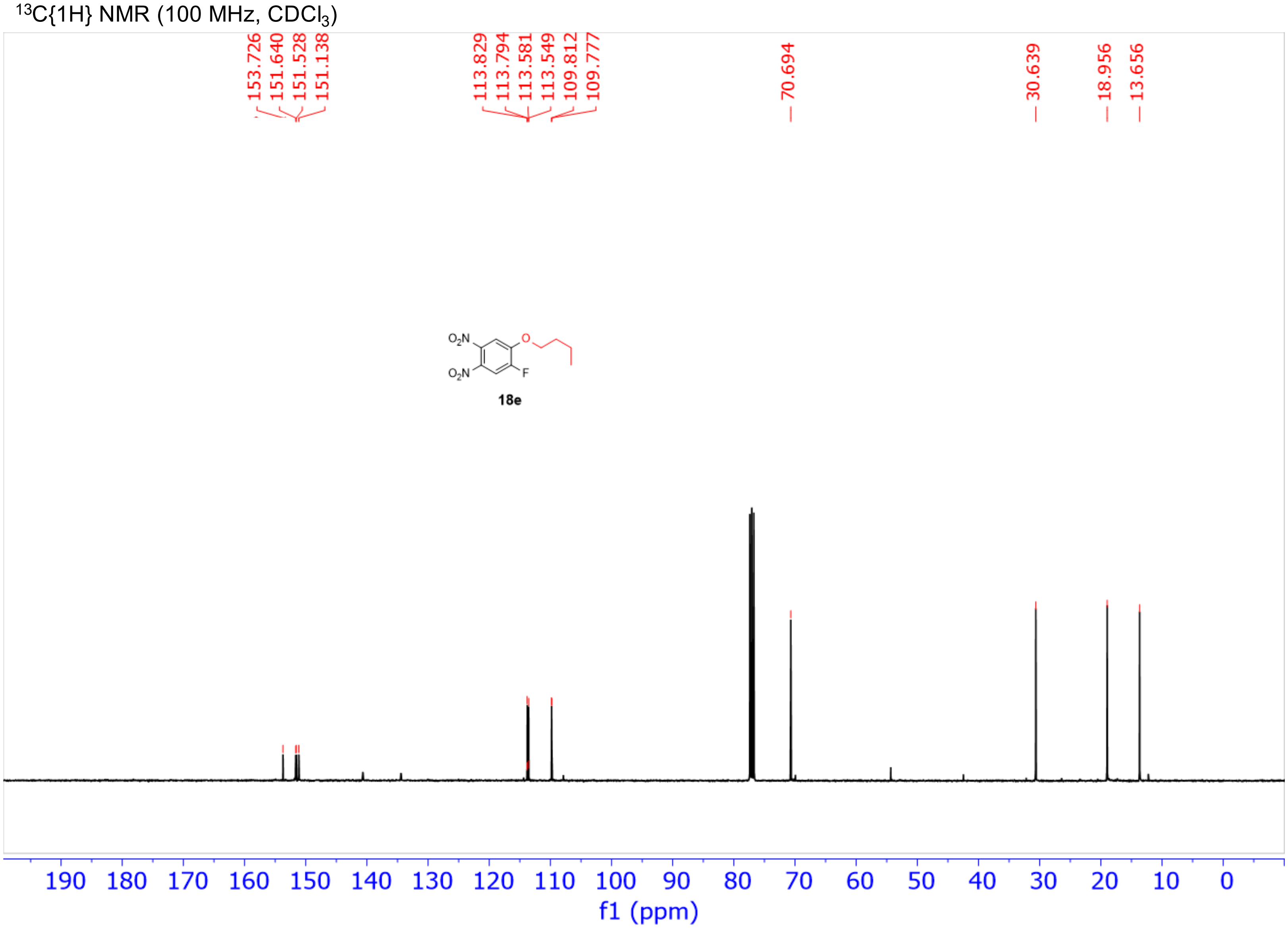

13C{1H} NMR (100 MHz, CDCl3)
153.726
151.640
151.528
151.138
113.829
113.794
113.581
113.549
109.812
109.777
70.694
30.639
18.956
13.656
O2N
O2N
O
F
18e
190 180 170 160 150 140 130 120 110 100 90 80 70 60 50 40 30 20 10 0
f1 (ppm)

^{1}H NMR (400 MHz, $CDCl_3$)

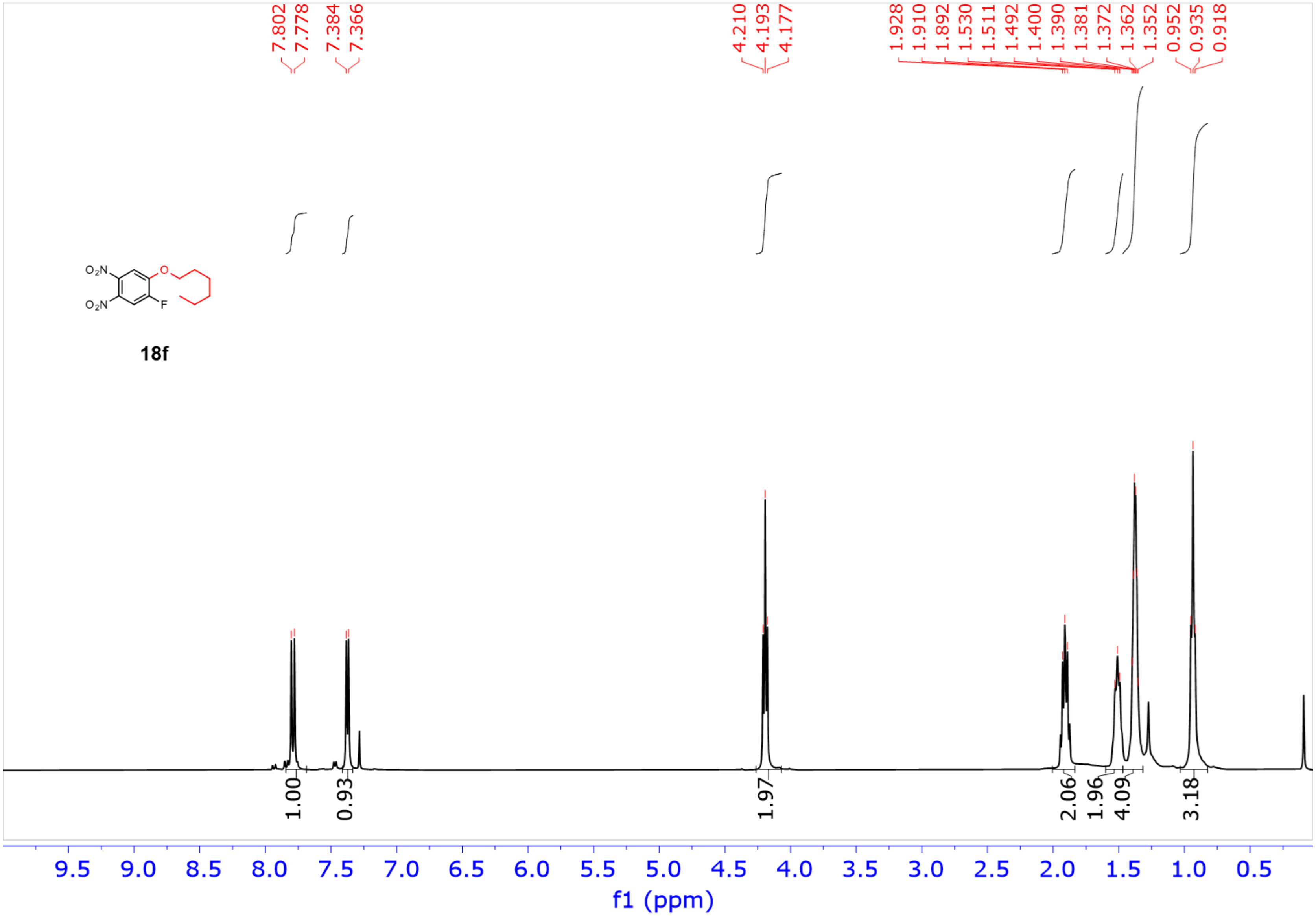

$^{13}C\{1H\}$ NMR (100 MHz, $CDCl_3$)

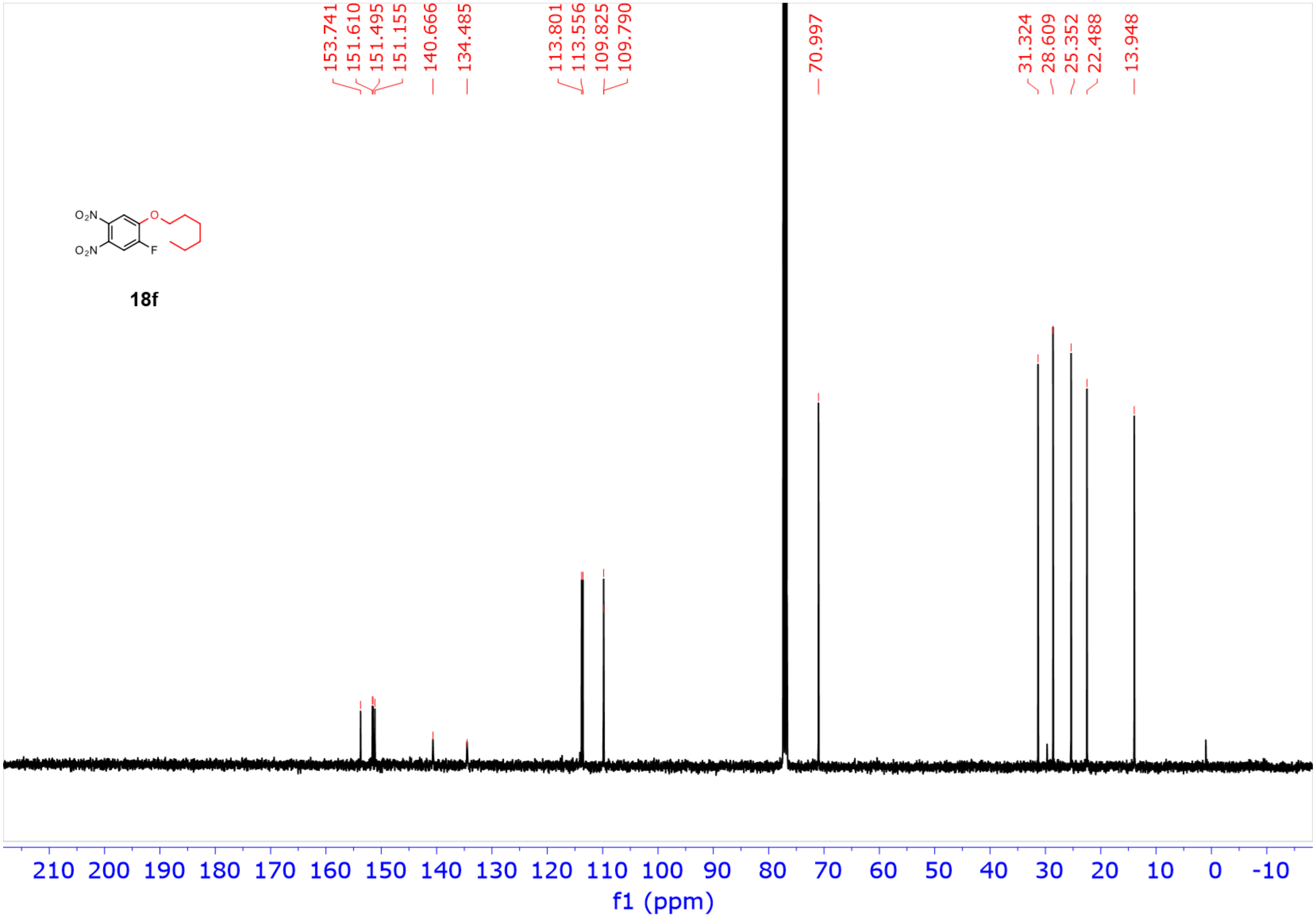

^1H NMR (400 MHz, $CDCl_3$)

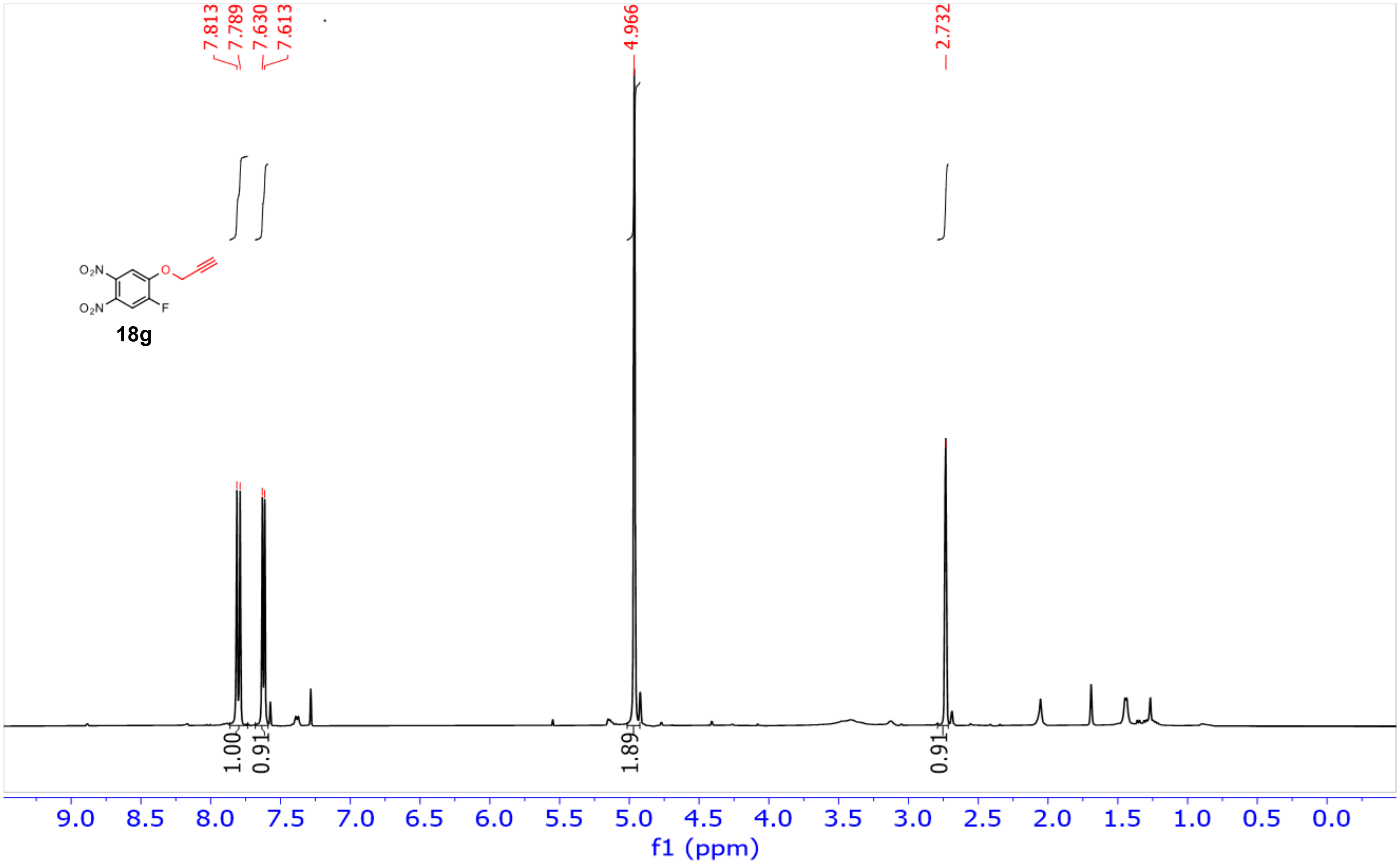

$^{13}C\{1H\}$ NMR (100 MHz, $CDCl_3$)

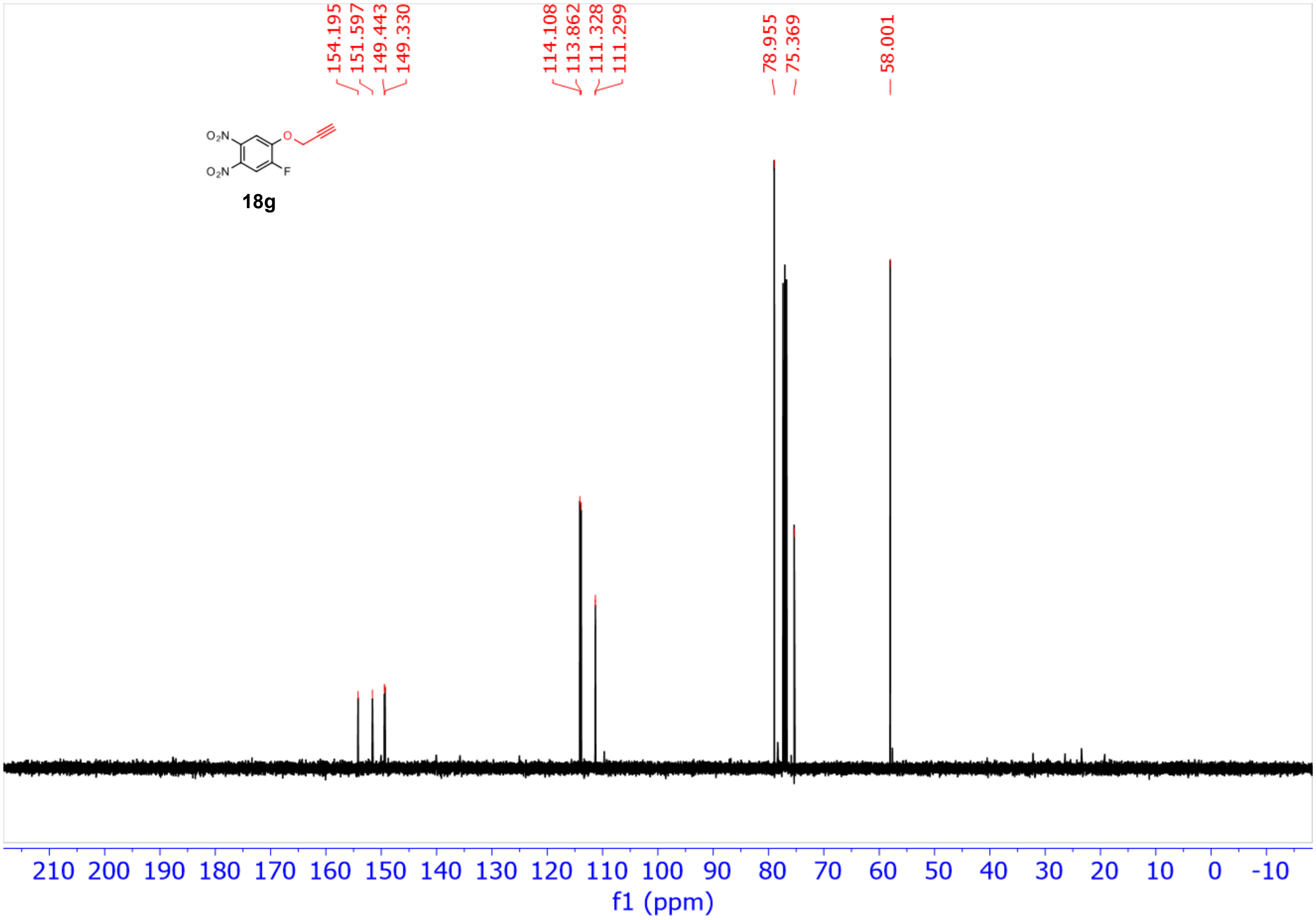

18g

^1H NMR (400 MHz, $CDCl_3$)

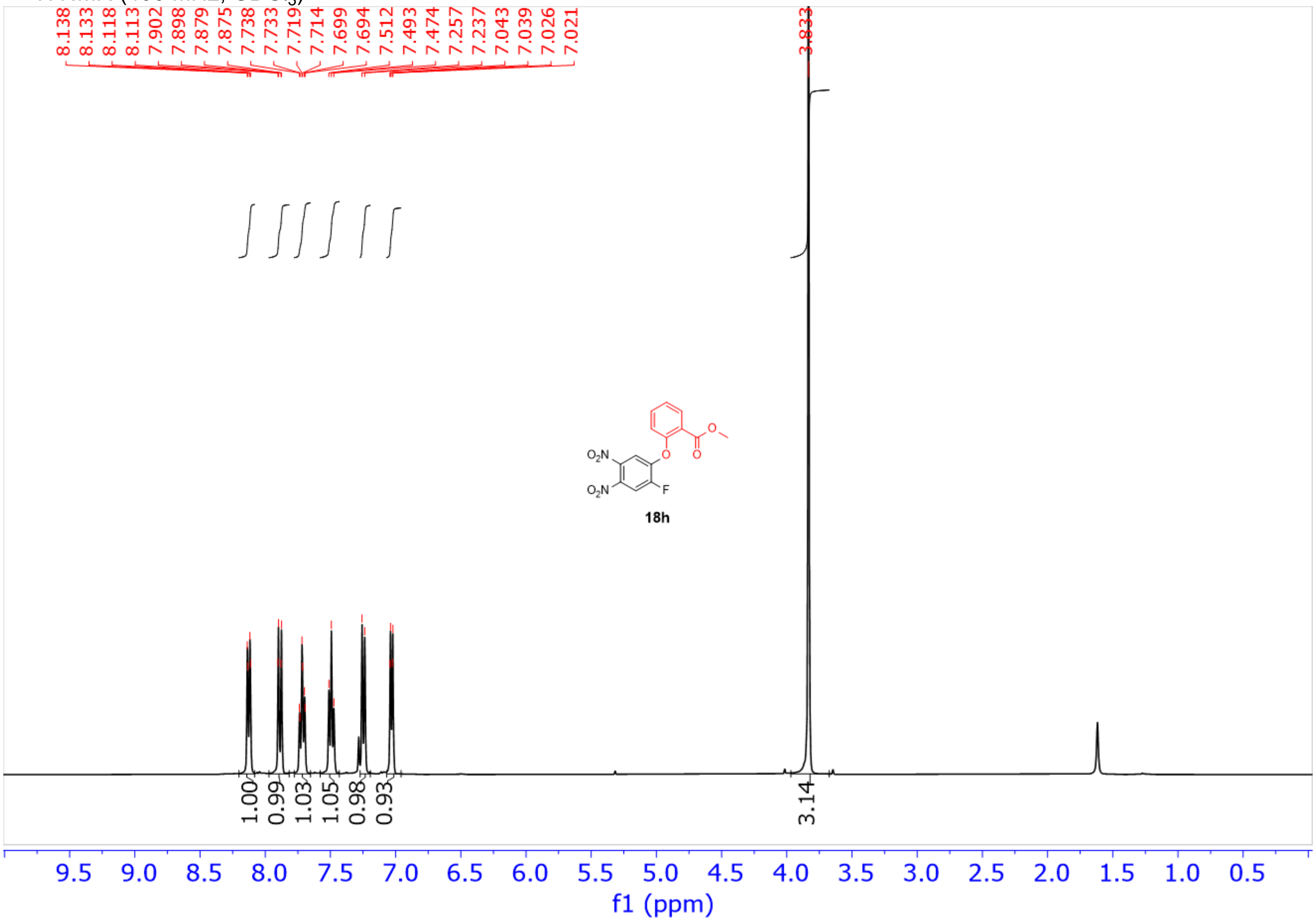

$^{13}C\{1H\}$ NMR (100 MHz, $CDCl_3$)

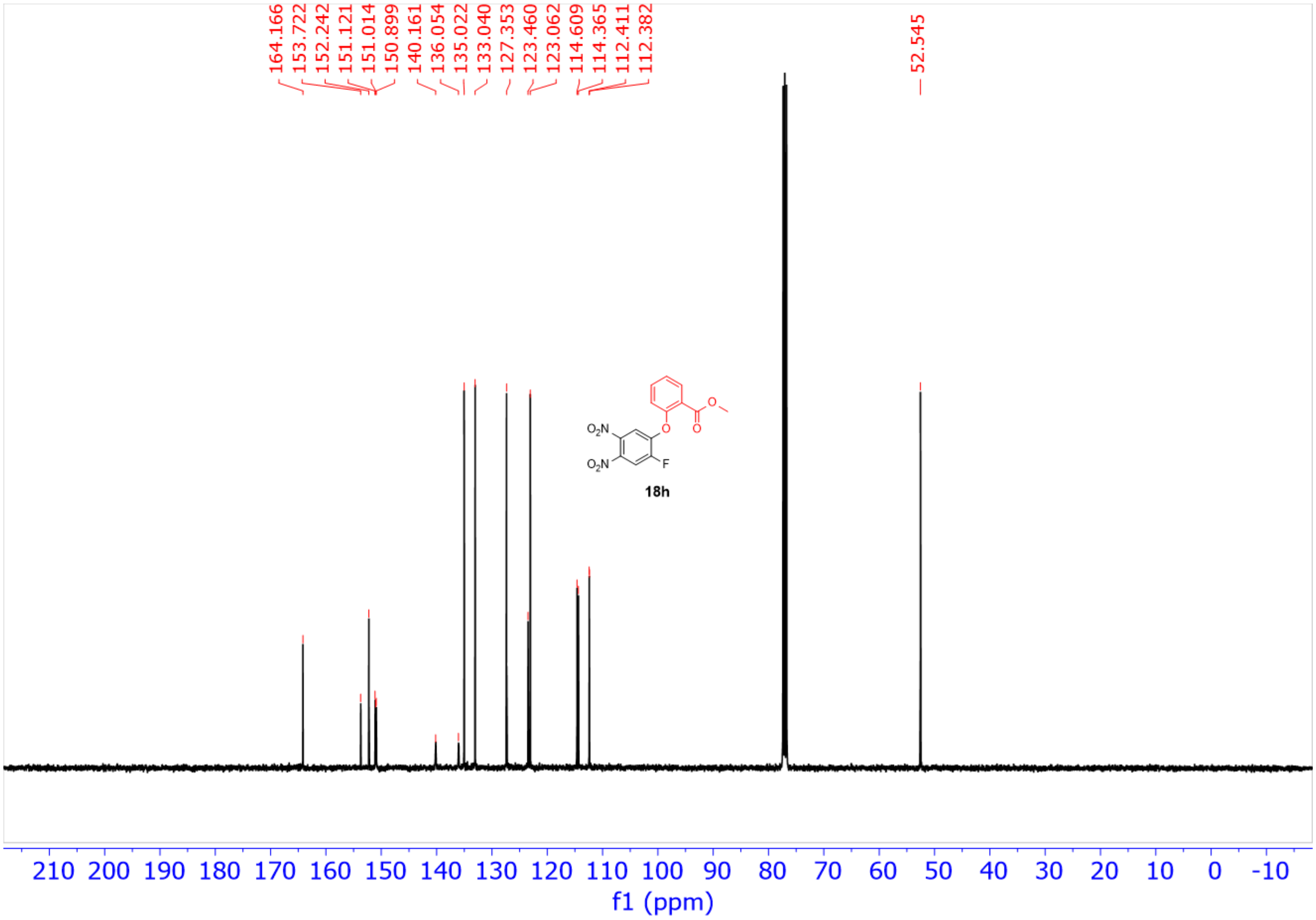

^1H NMR (400 MHz, $(CD_3)_2CO$)

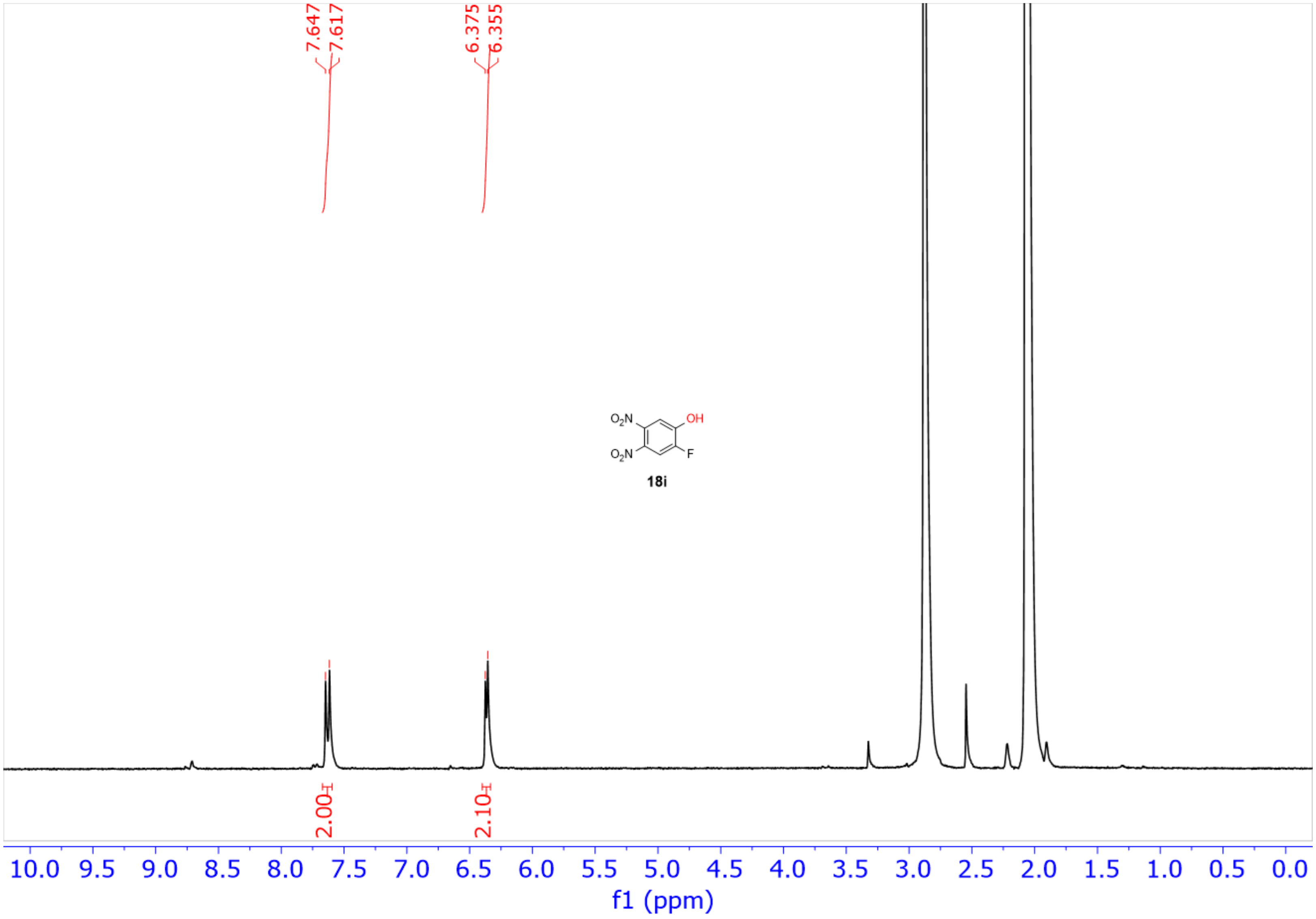

$^{13}C\{^1H\}$ NMR (100 MHz, $(CD_3)_2CO$)

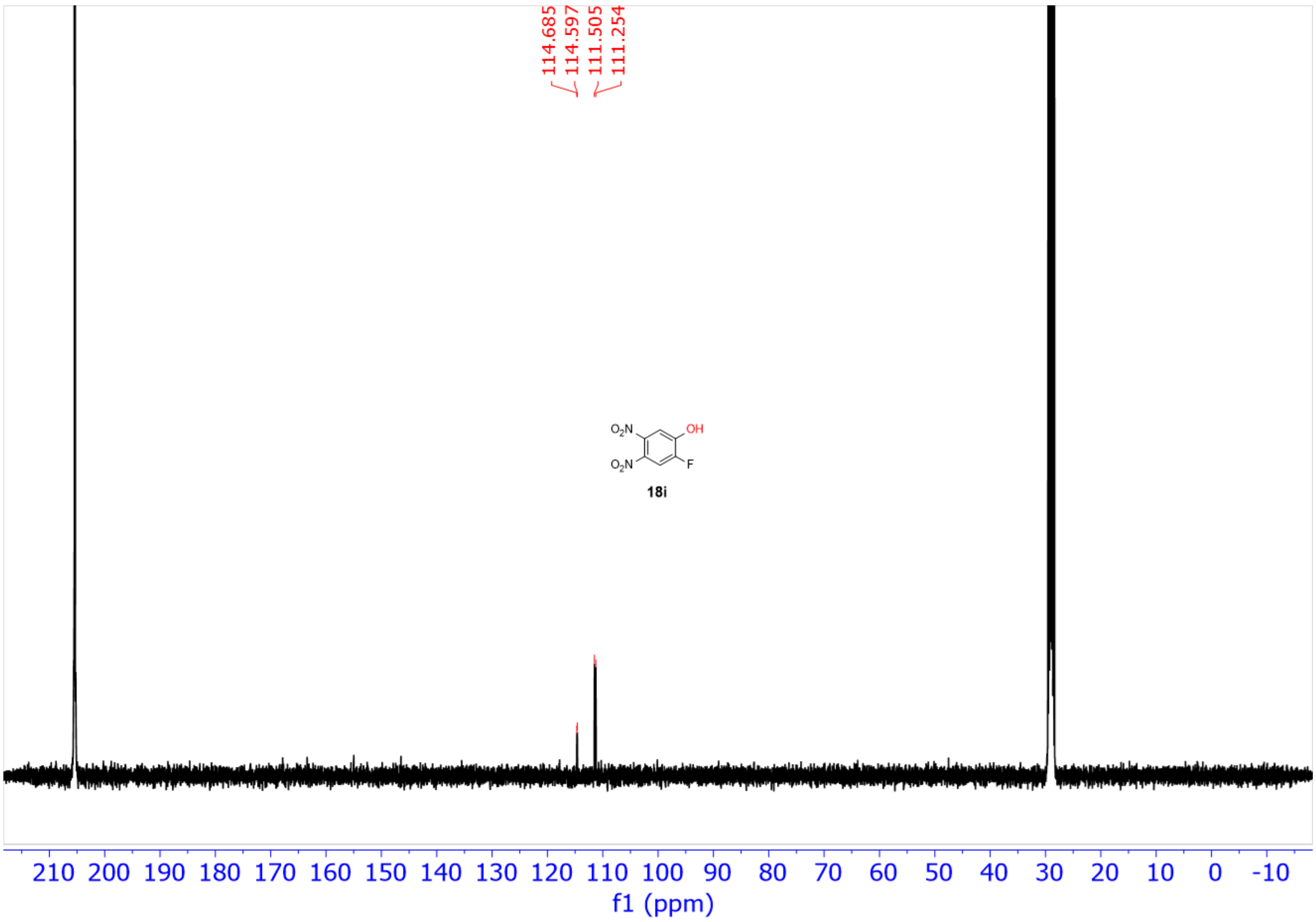

^1H NMR (400 MHz, $(CD_3)_2SO$)

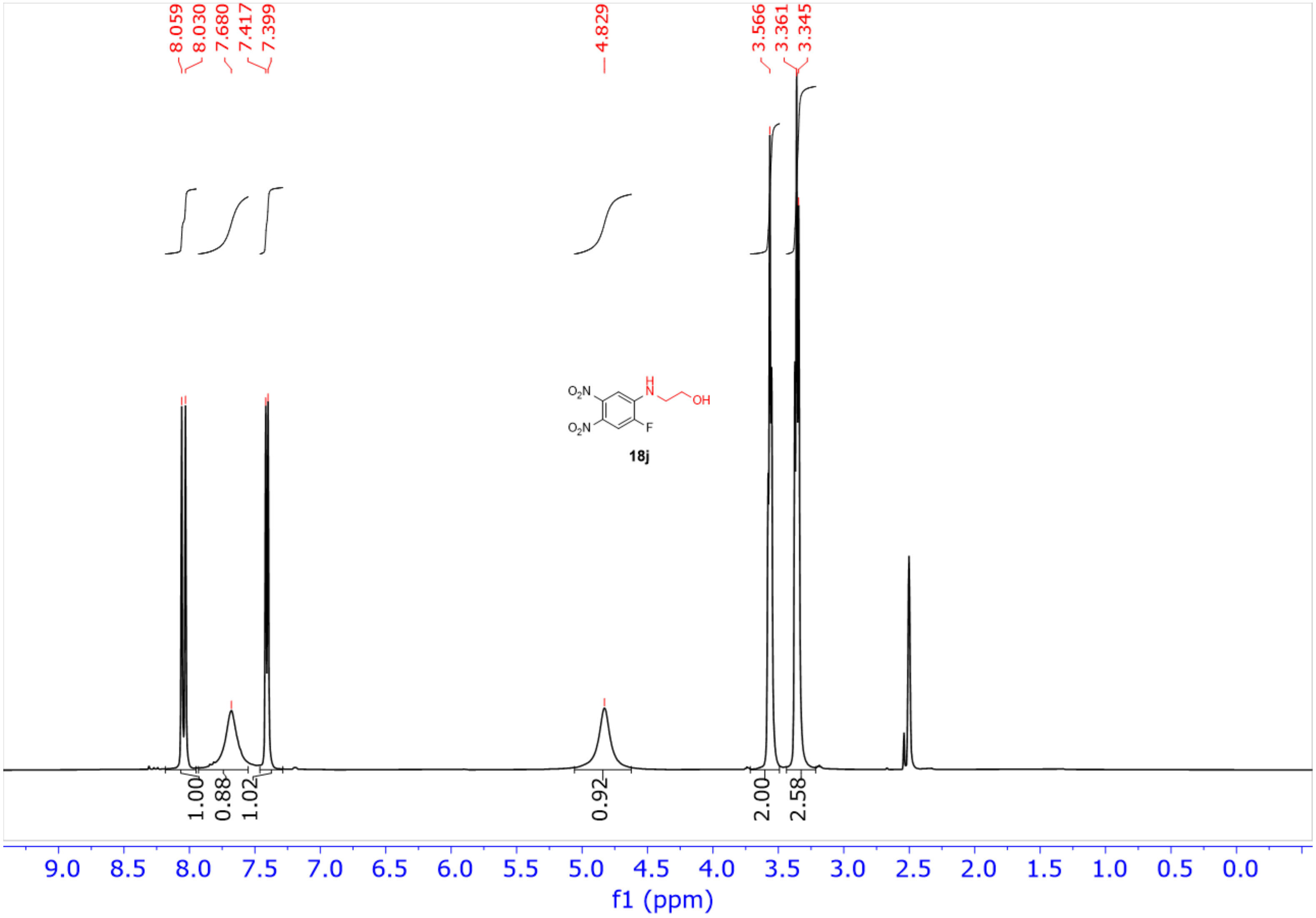

$^{13}C\{1H\}$ NMR (100 MHz, $(CD_3)_2SO$)

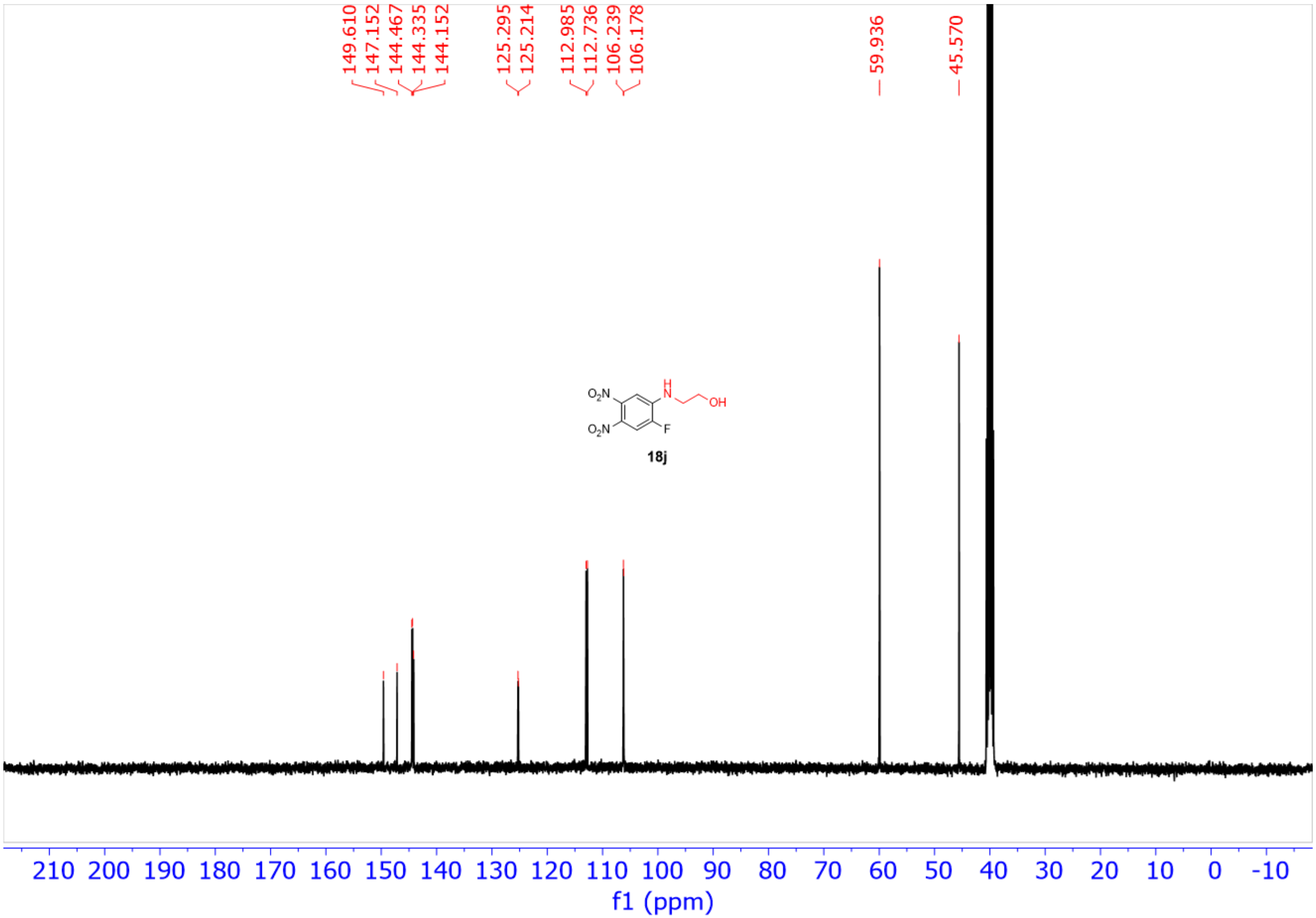

^{1}H NMR (400 MHz, $(CD_3)_2SO$)

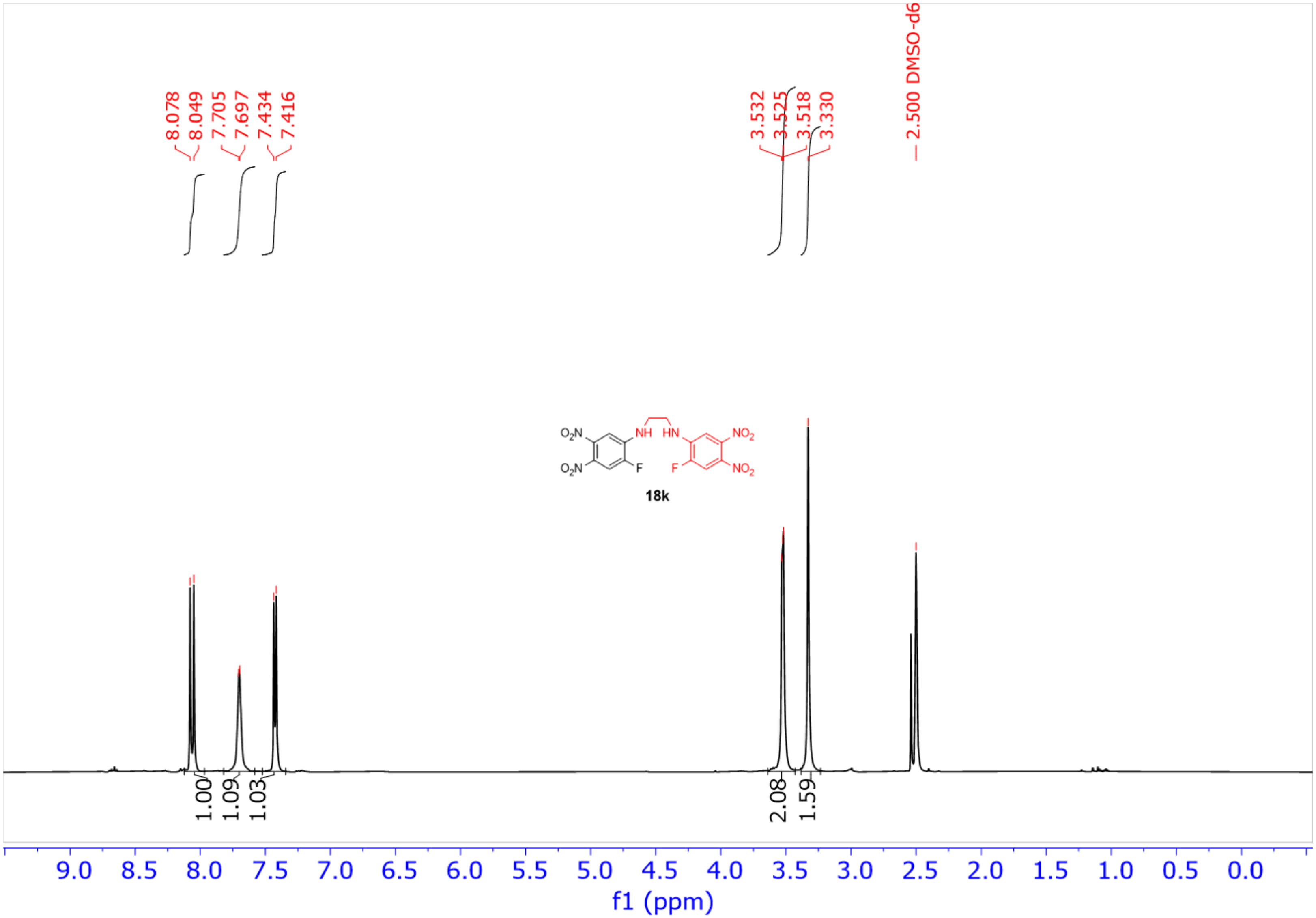

$^{13}C\{1H\}$ NMR (100 MHz, $(CD_3)_2SO$)

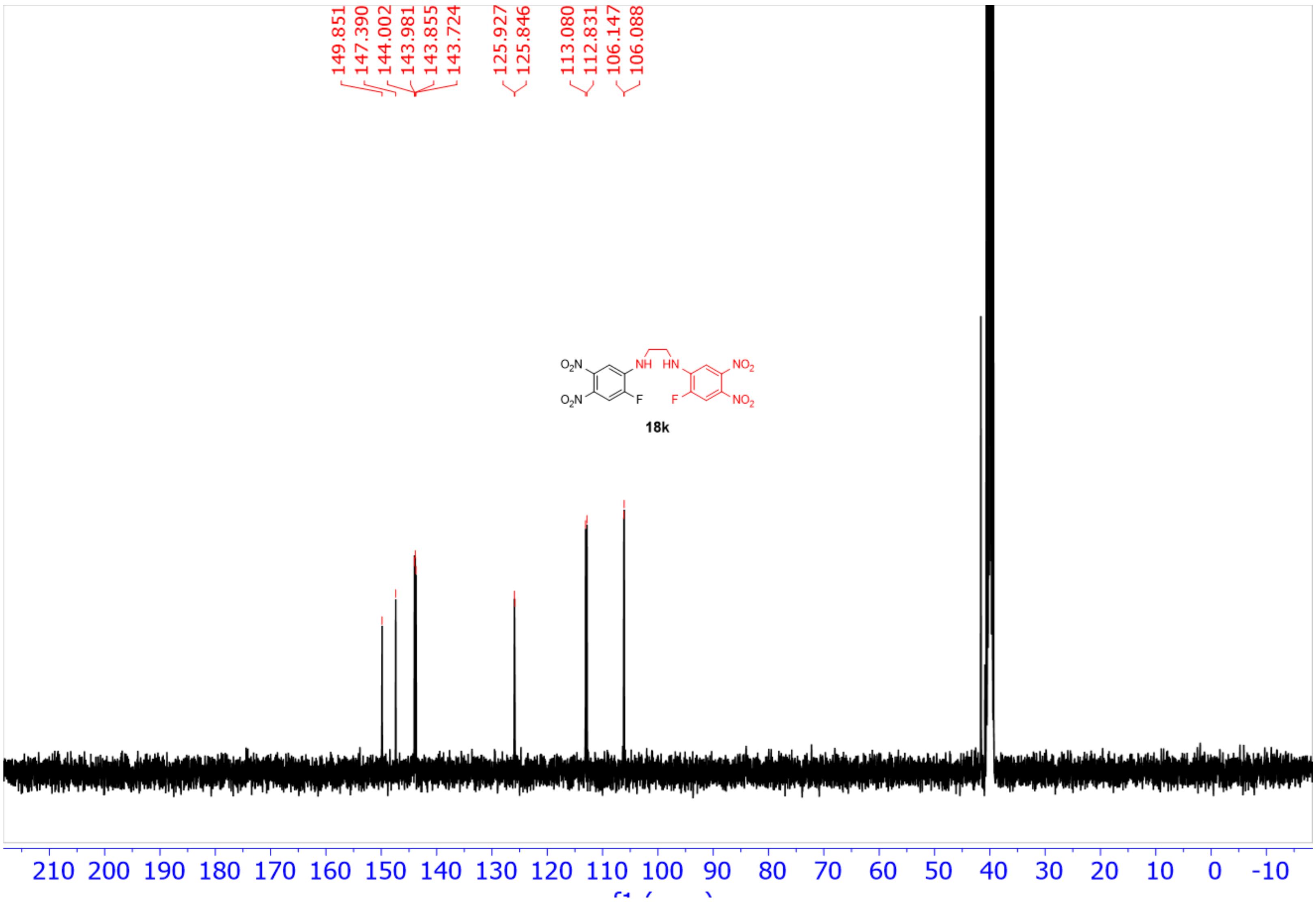

18k

^1H NMR (400 MHz, $(CD_3)_2SO$)

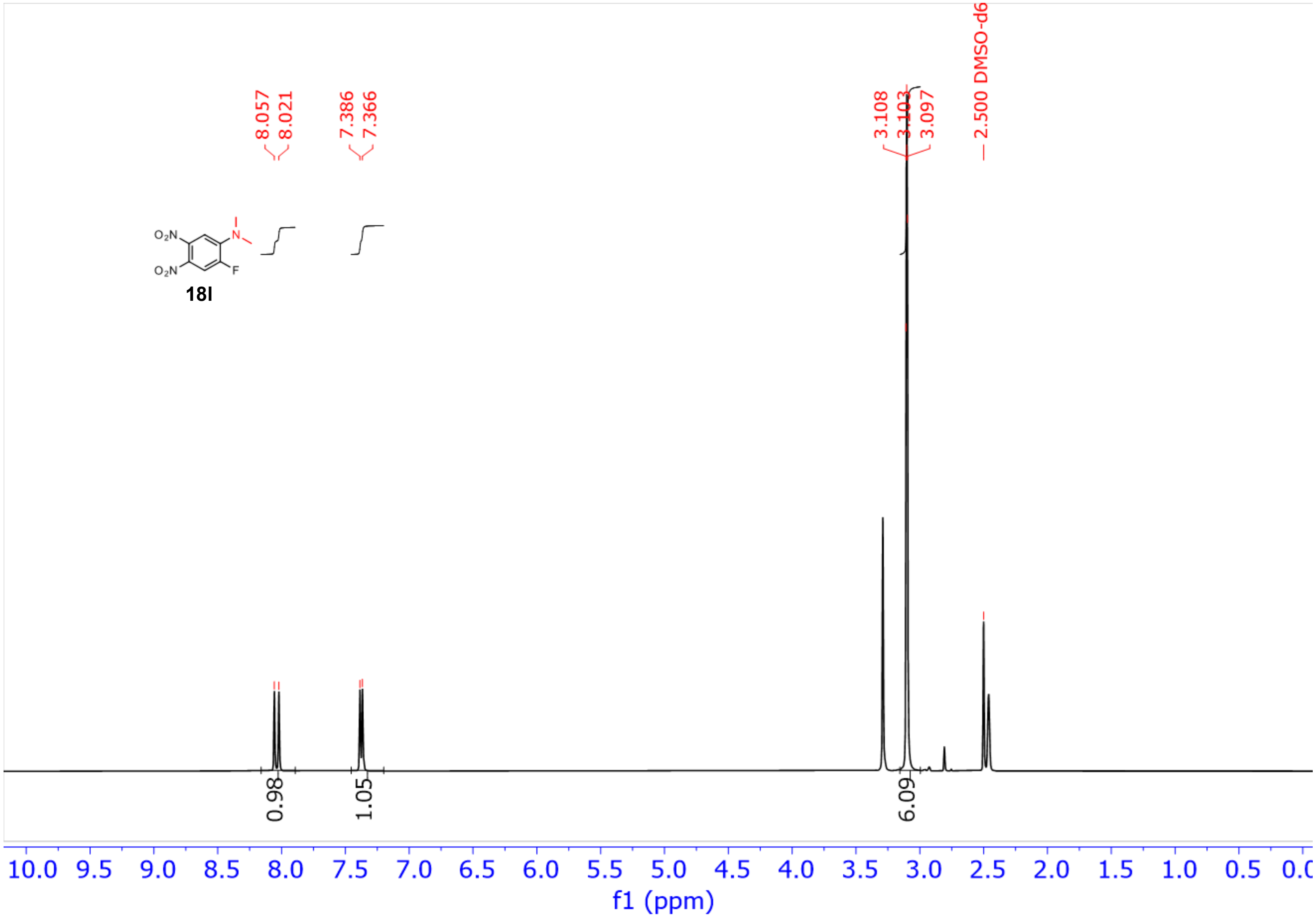

$^{13}C\{^{1}H\}$ NMR (100 MHz, $(CD_3)_2SO$)

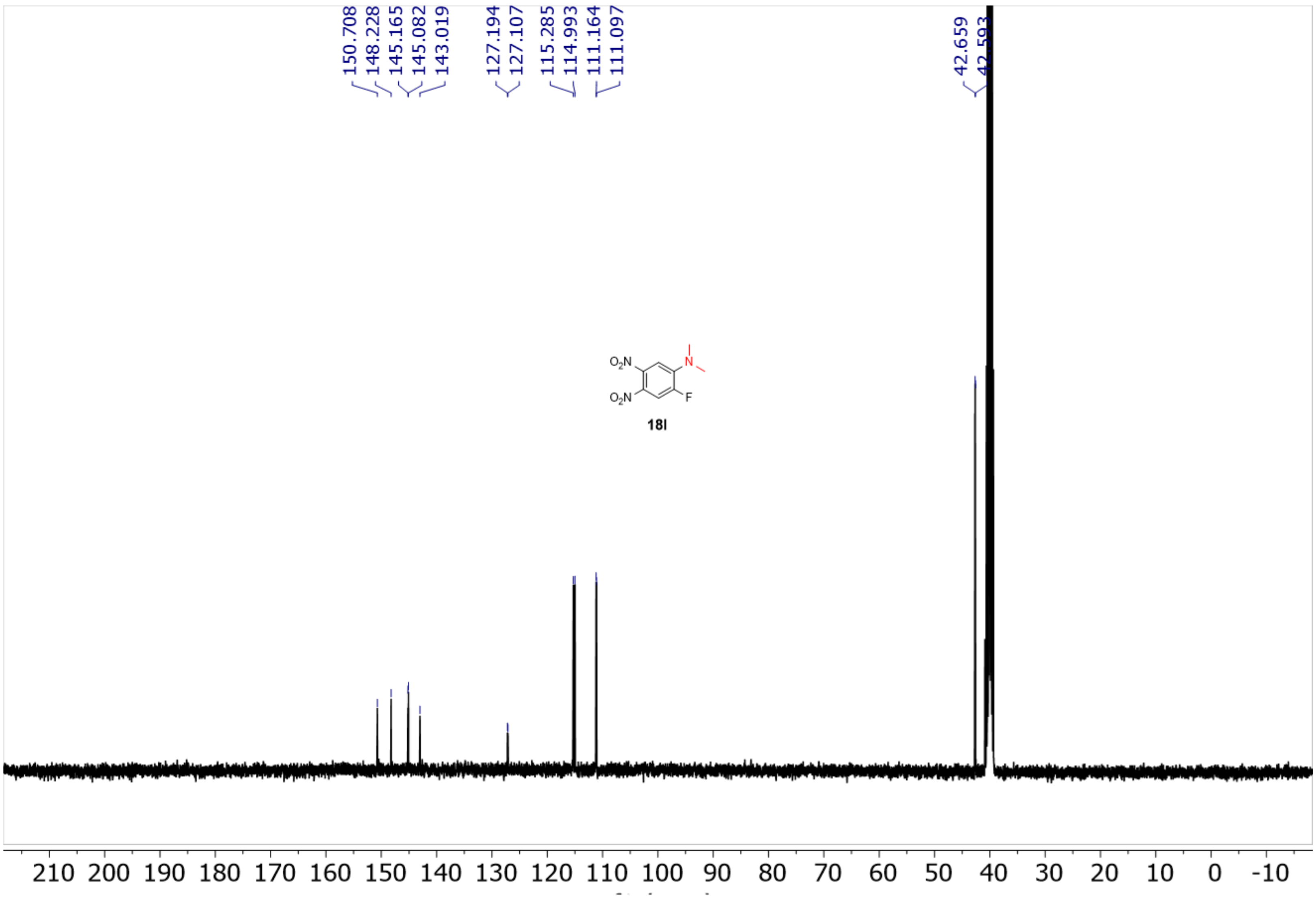

^{1}H NMR (400 MHz, $(CD_3)_2SO$)

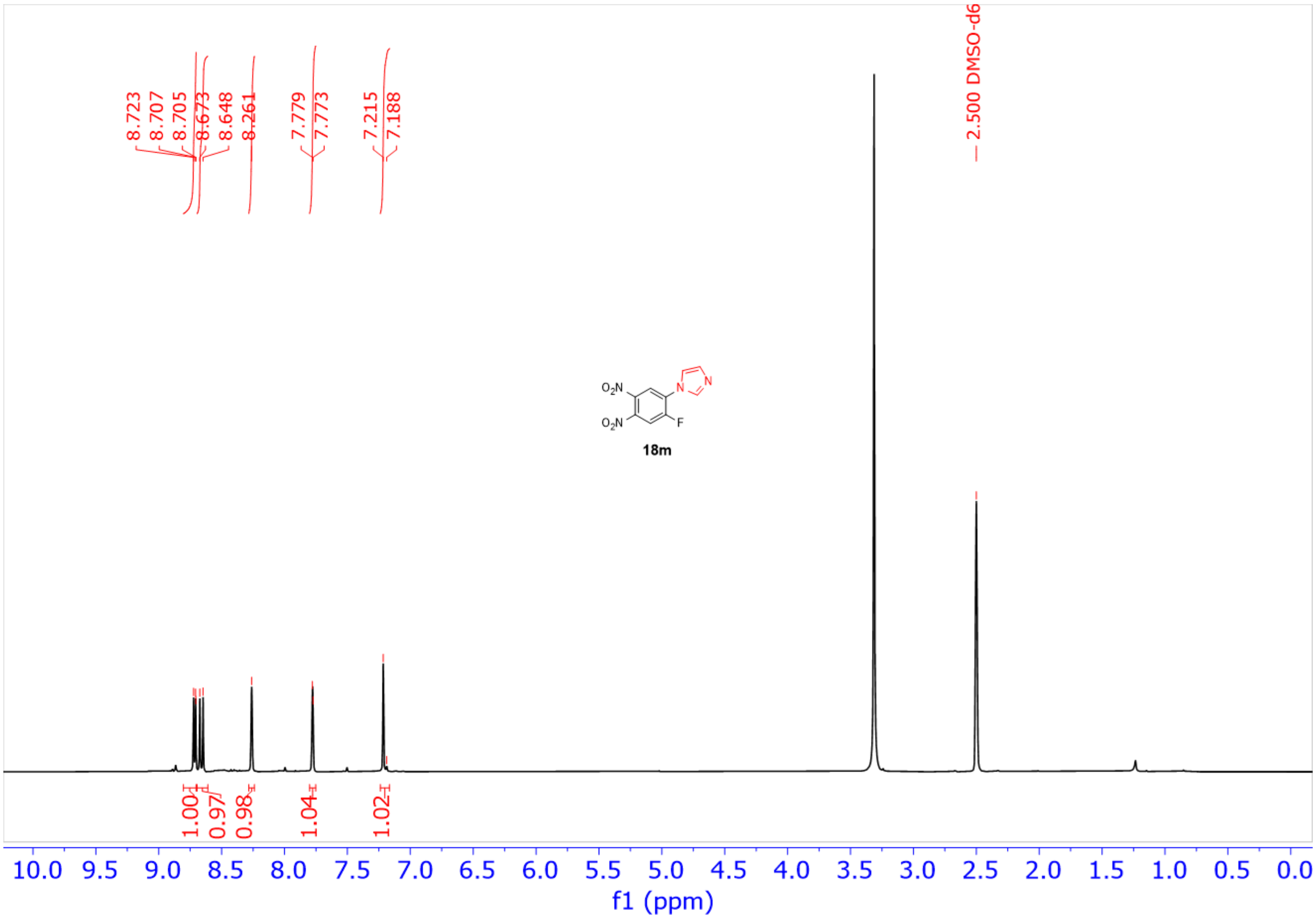

$^{13}C\{1H\}$ NMR (100 MHz, $(CD_3)_2SO$)

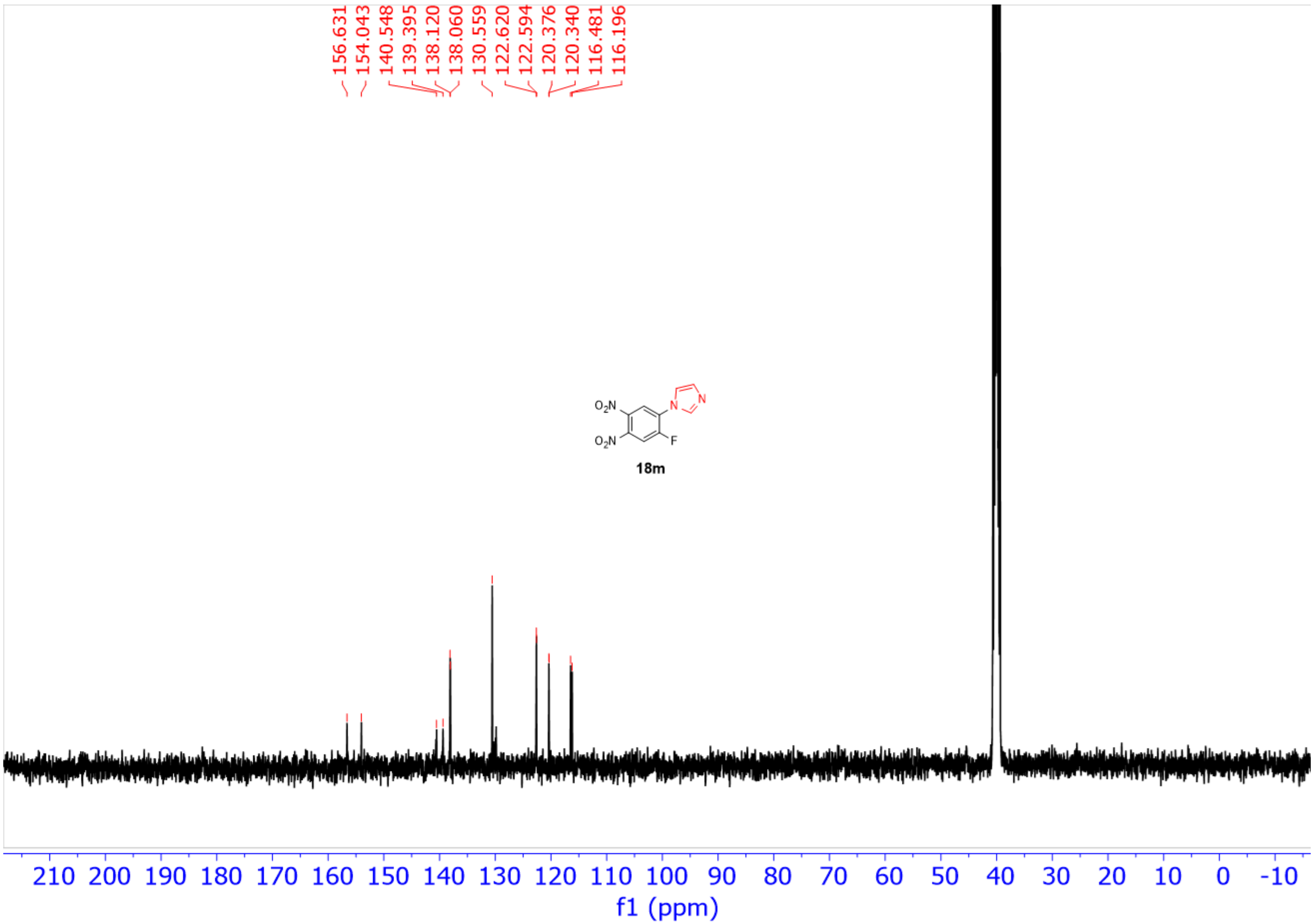

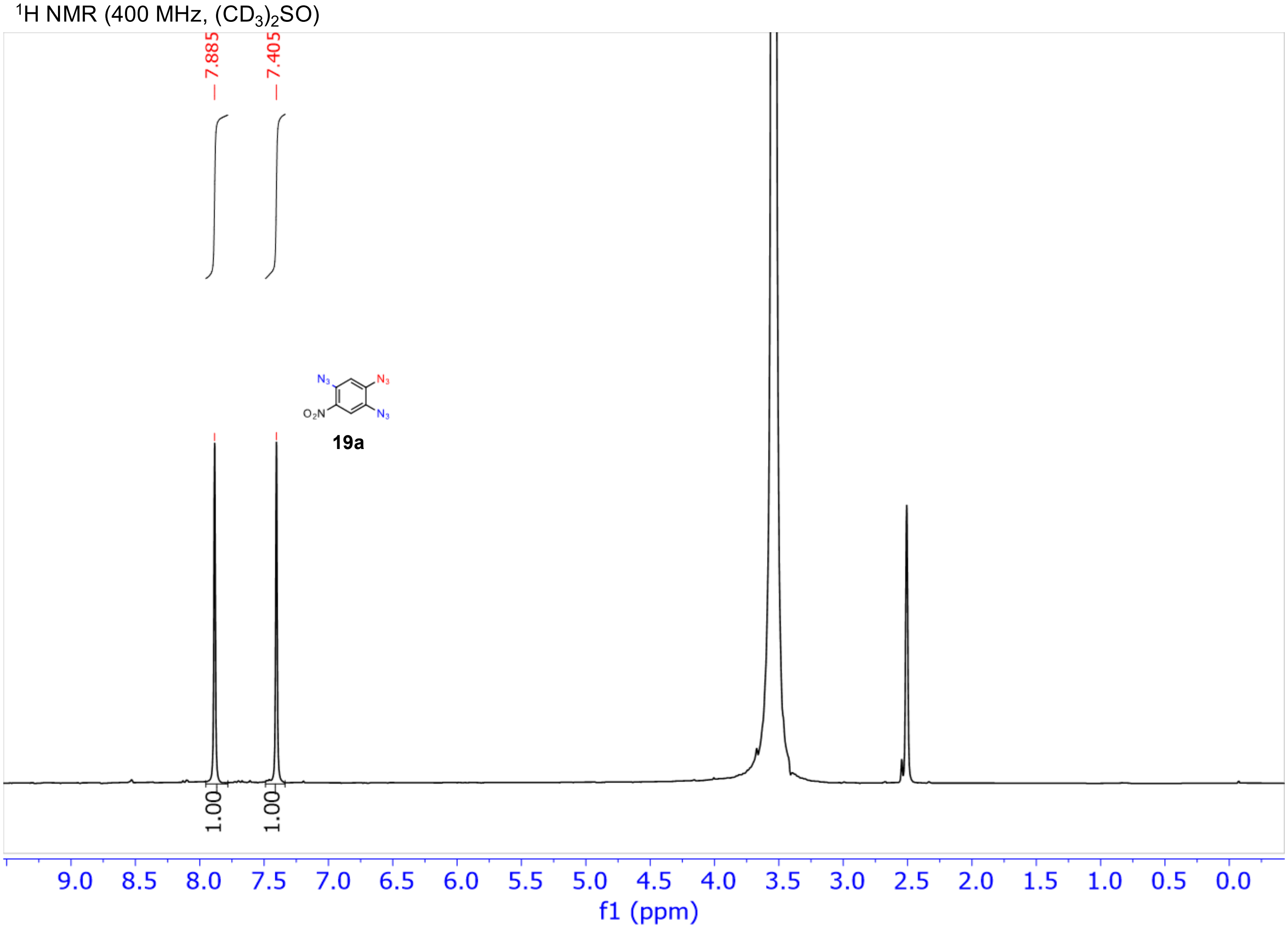

^{1}H NMR (400 MHz, $(CD_3)_2SO$)
7.885
7.405
19a
1.00
1.00
9.0 8.5 8.0 7.5 7.0 6.5 6.0 5.5 5.0 4.5 4.0 3.5 3.0 2.5 2.0 1.5 1.0 0.5 0.0
f1 (ppm)

^{1}H NMR (400 MHz, $(CD_3)_2CO$)

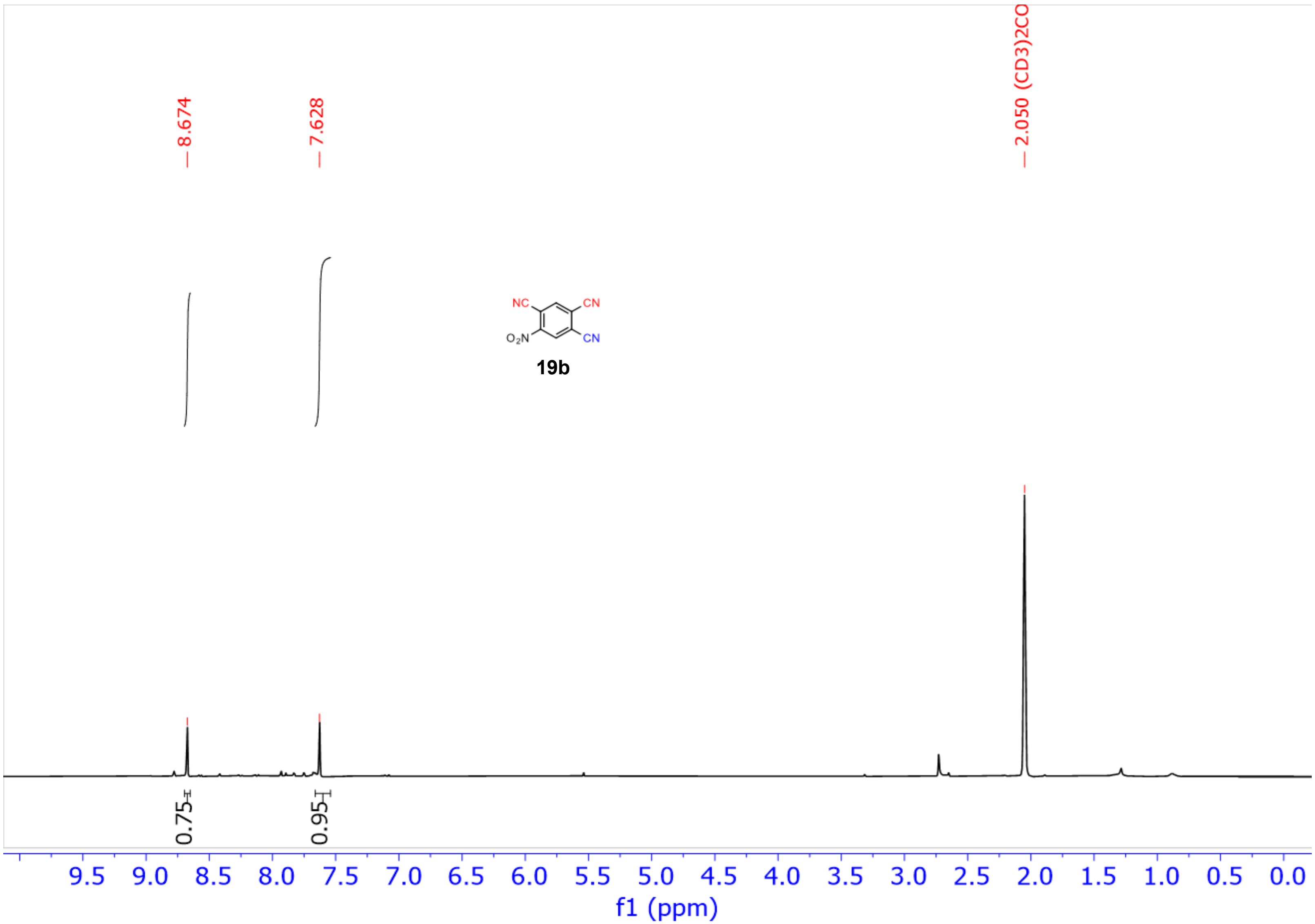

$^{13}C\{1H\}$ NMR (100 MHz, $(CD_3)_2CO$)

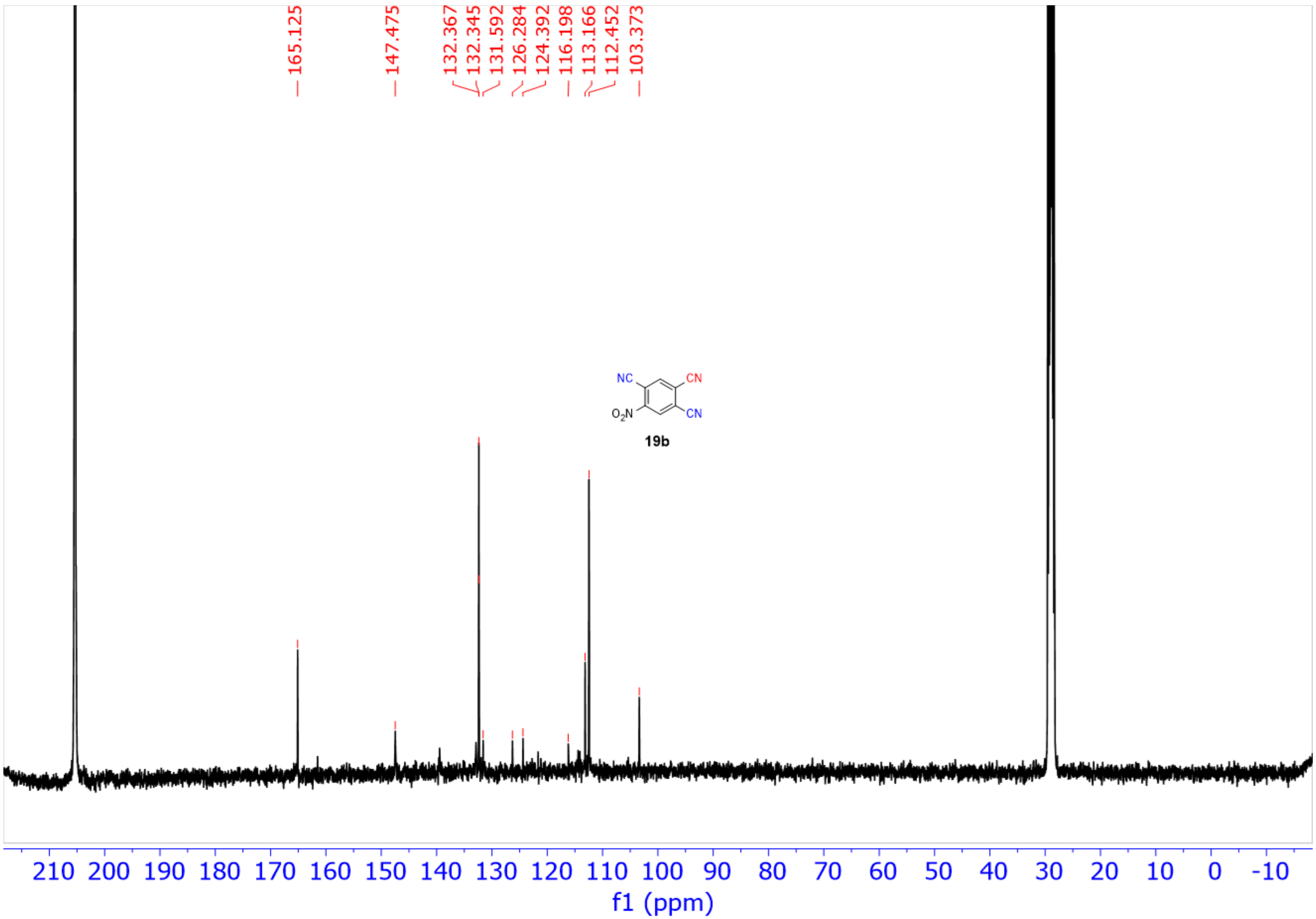

^{1}H NMR (400 MHz, $CDCl_3$)

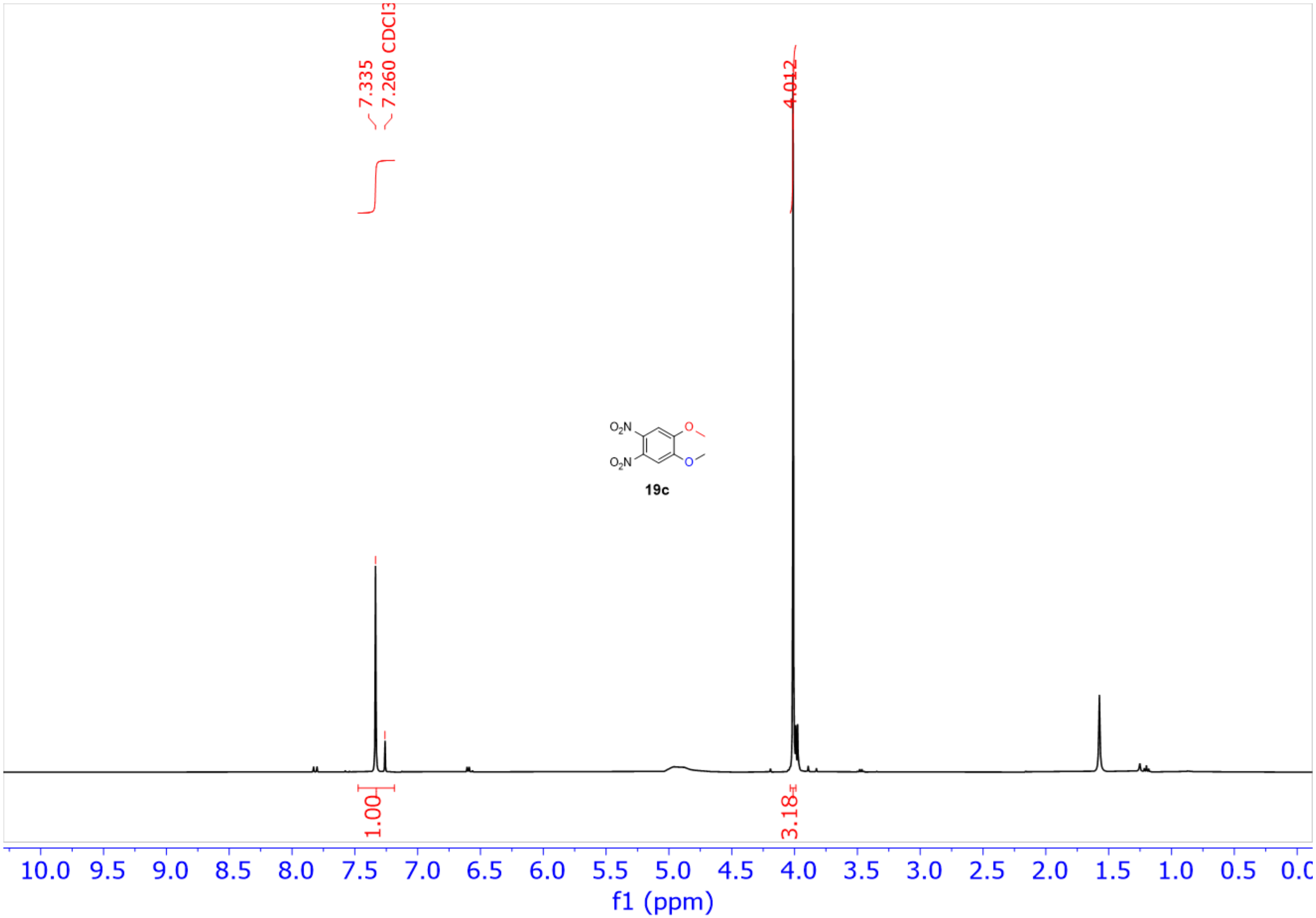

^1H NMR (400 MHz, $CDCl_3$)

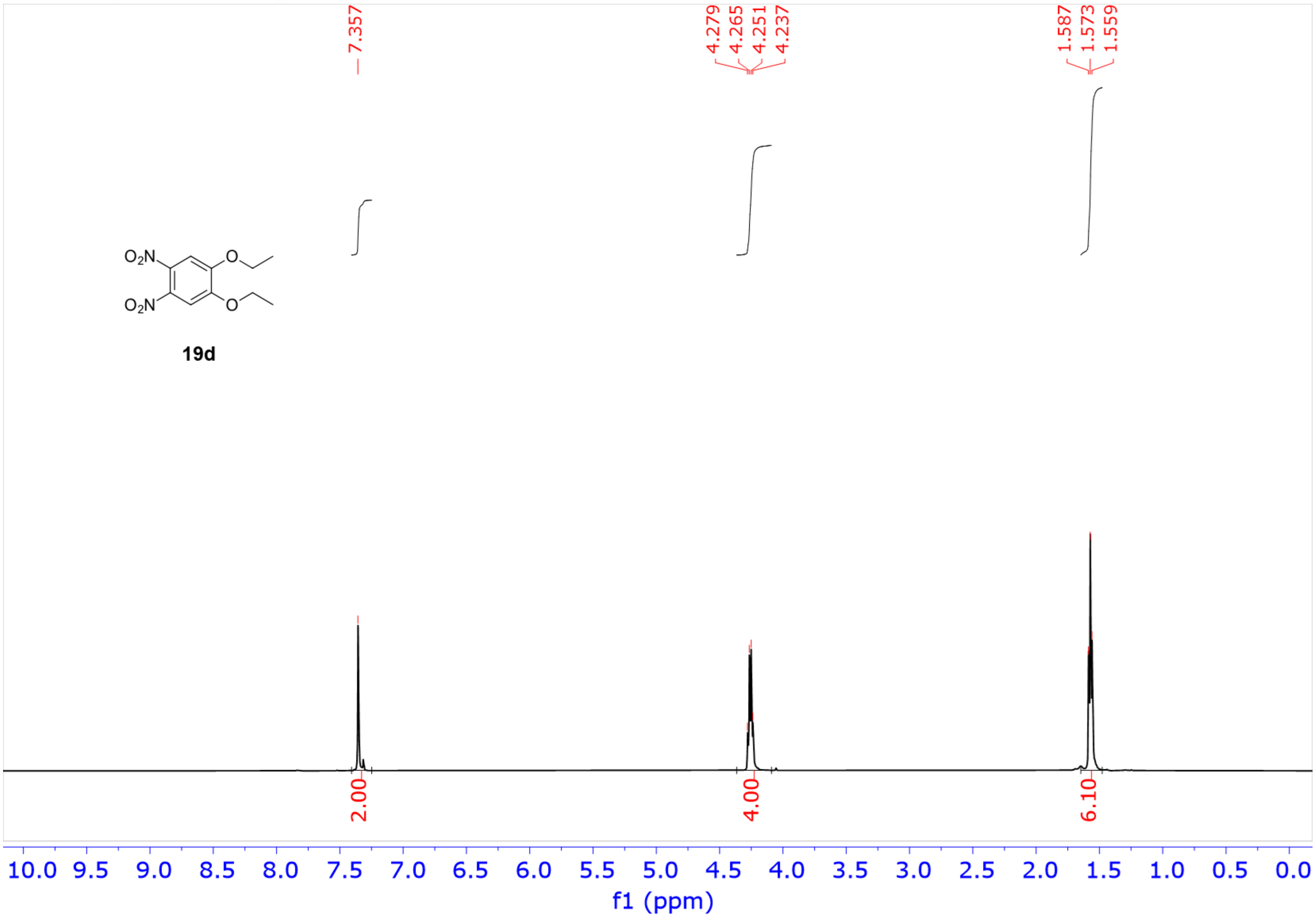

^{1}H NMR (400 MHz, $CDCl_3$)

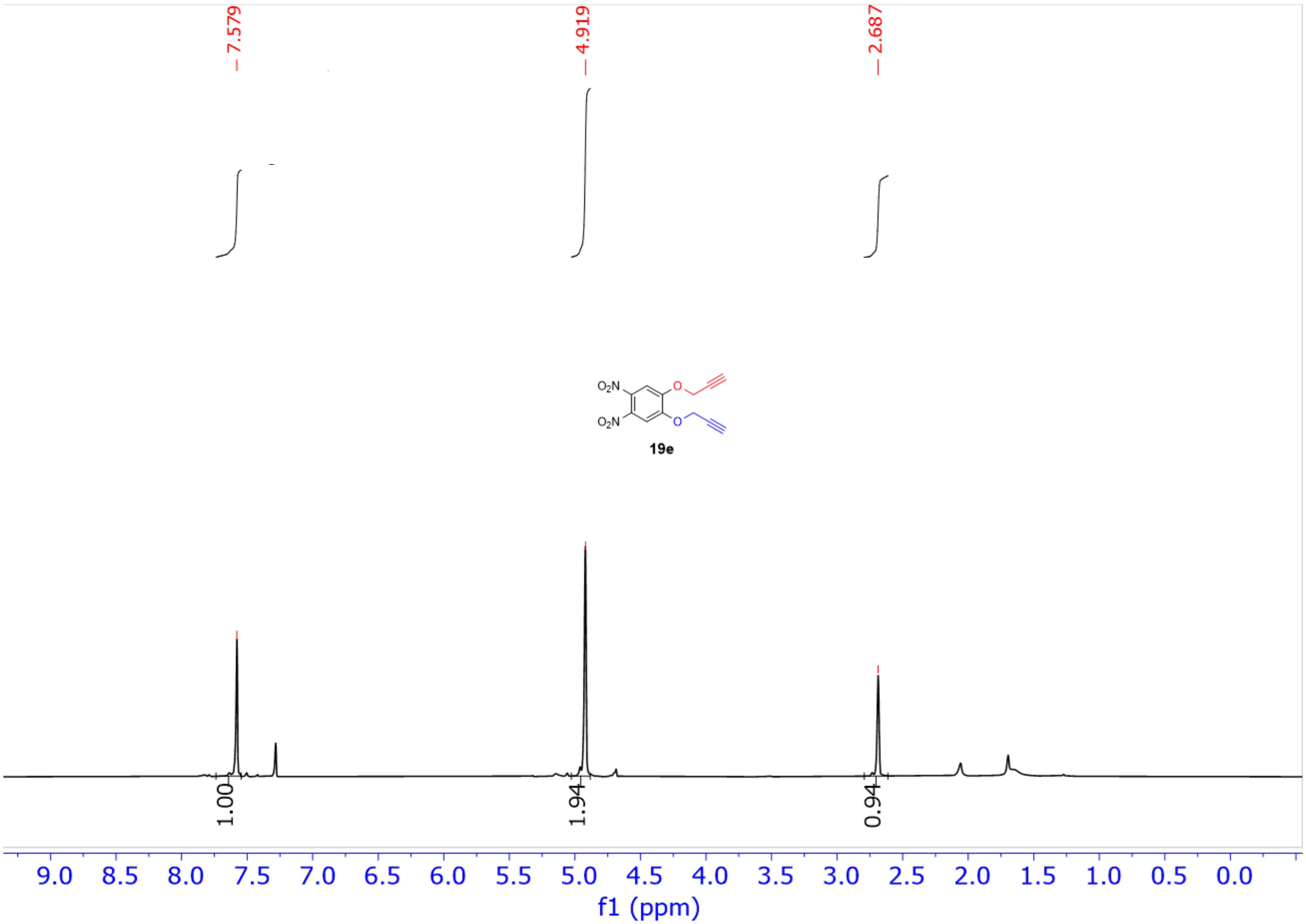

$^{13}C\{1H\}$ NMR (100 MHz, $CDCl_3$)

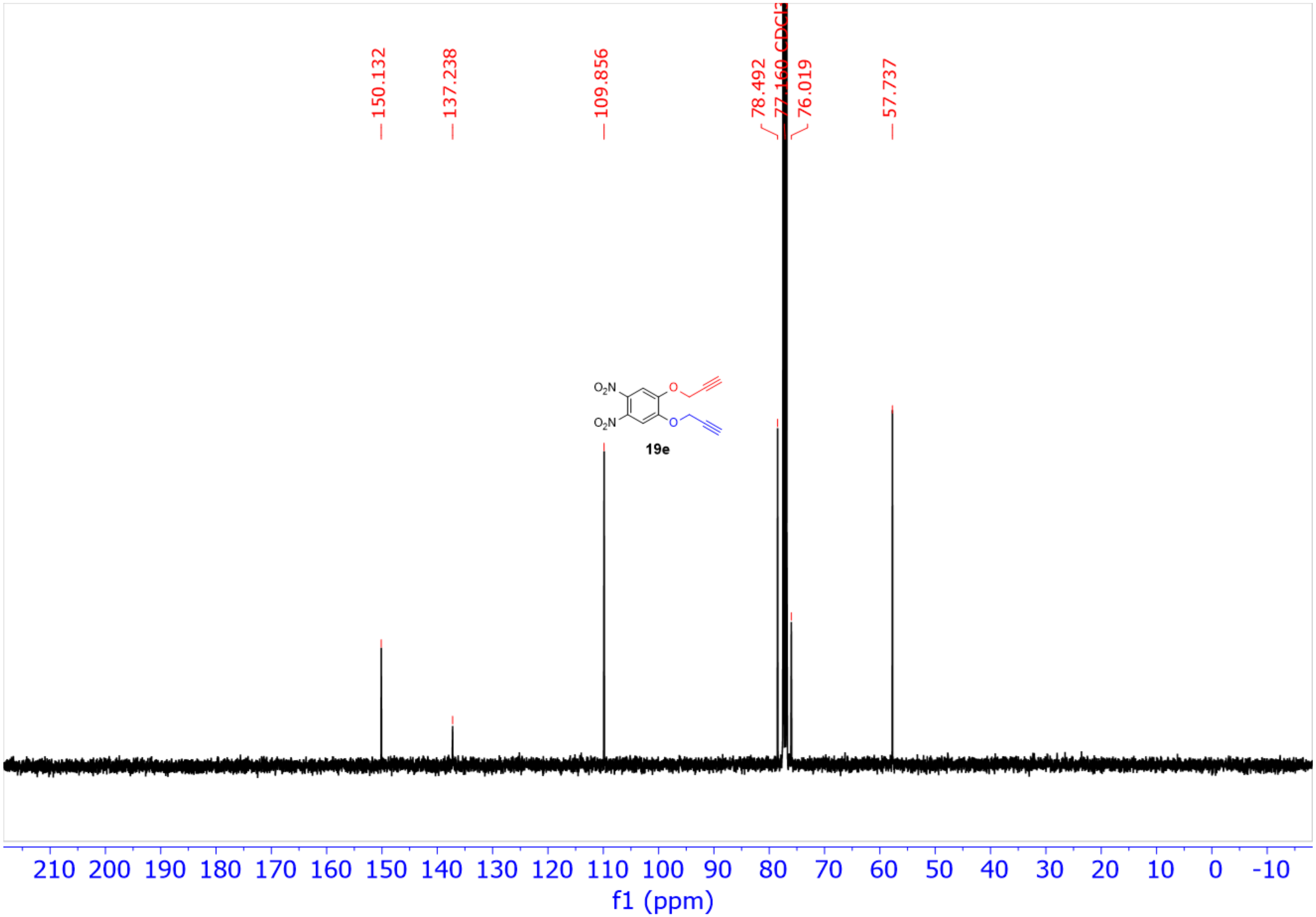

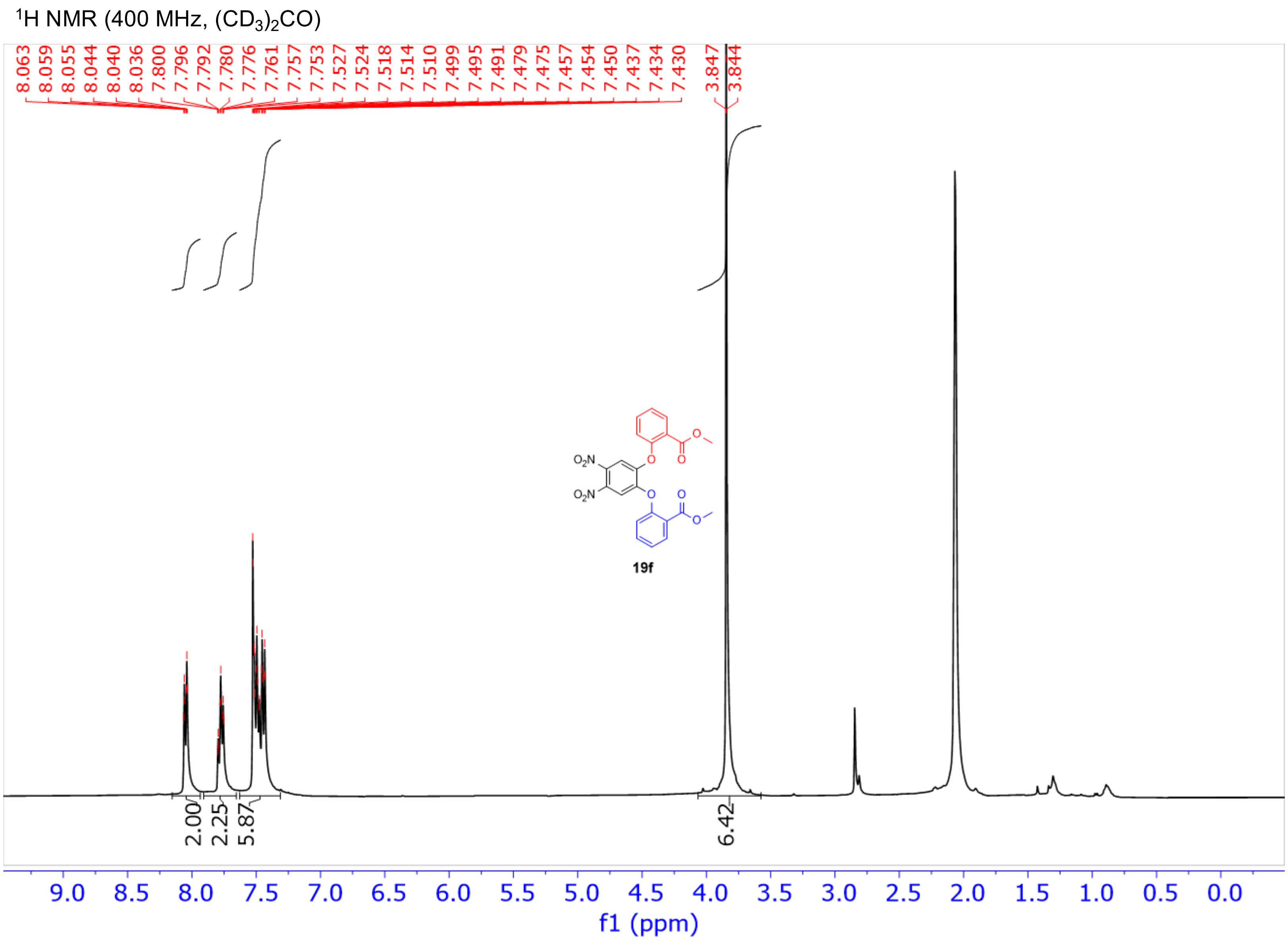

1H NMR (400 MHz, (CD3)2CO)
8.063
8.059
8.055
8.044
8.040
8.036
7.800
7.796
7.792
7.780
7.776
7.761
7.757
7.753
7.527
7.524
7.518
7.514
7.510
7.499
7.495
7.491
7.479
7.475
7.457
7.454
7.450
7.437
7.434
7.430
3.847
3.844
O2N
O2N
O
O
O
O
O
O
19f
2.00
2.25
5.87
6.42
9.0
8.5
8.0
7.5
7.0
6.5
6.0
5.5
5.0
4.5
4.0
3.5
3.0
2.5
2.0
1.5
1.0
0.5
0.0
f1 (ppm)

$^{13}C\{1H\}$ NMR (100 MHz, $(CD_3)_2CO$)

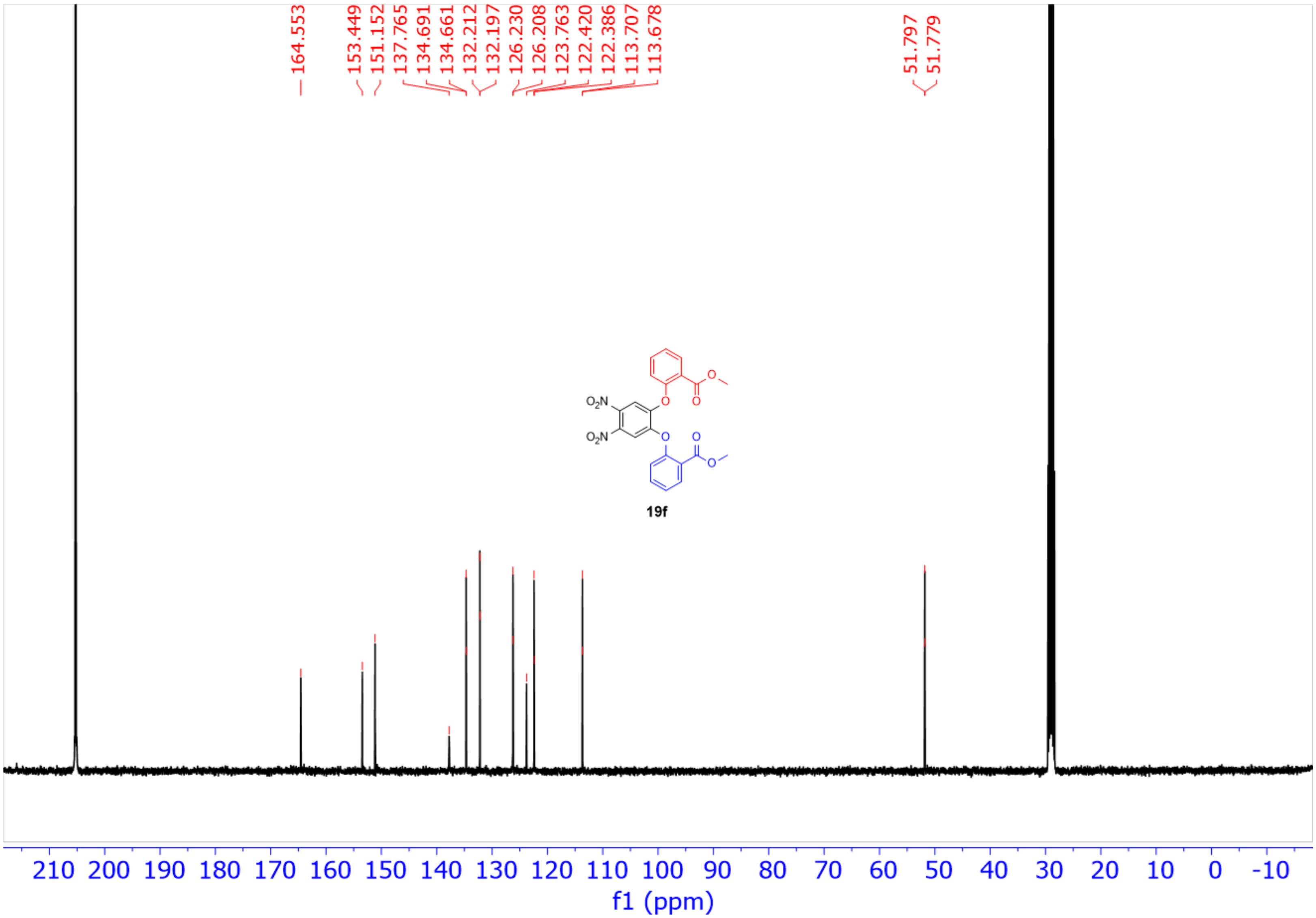

^1H NMR (400 MHz, $(CD_3)_2SO$)

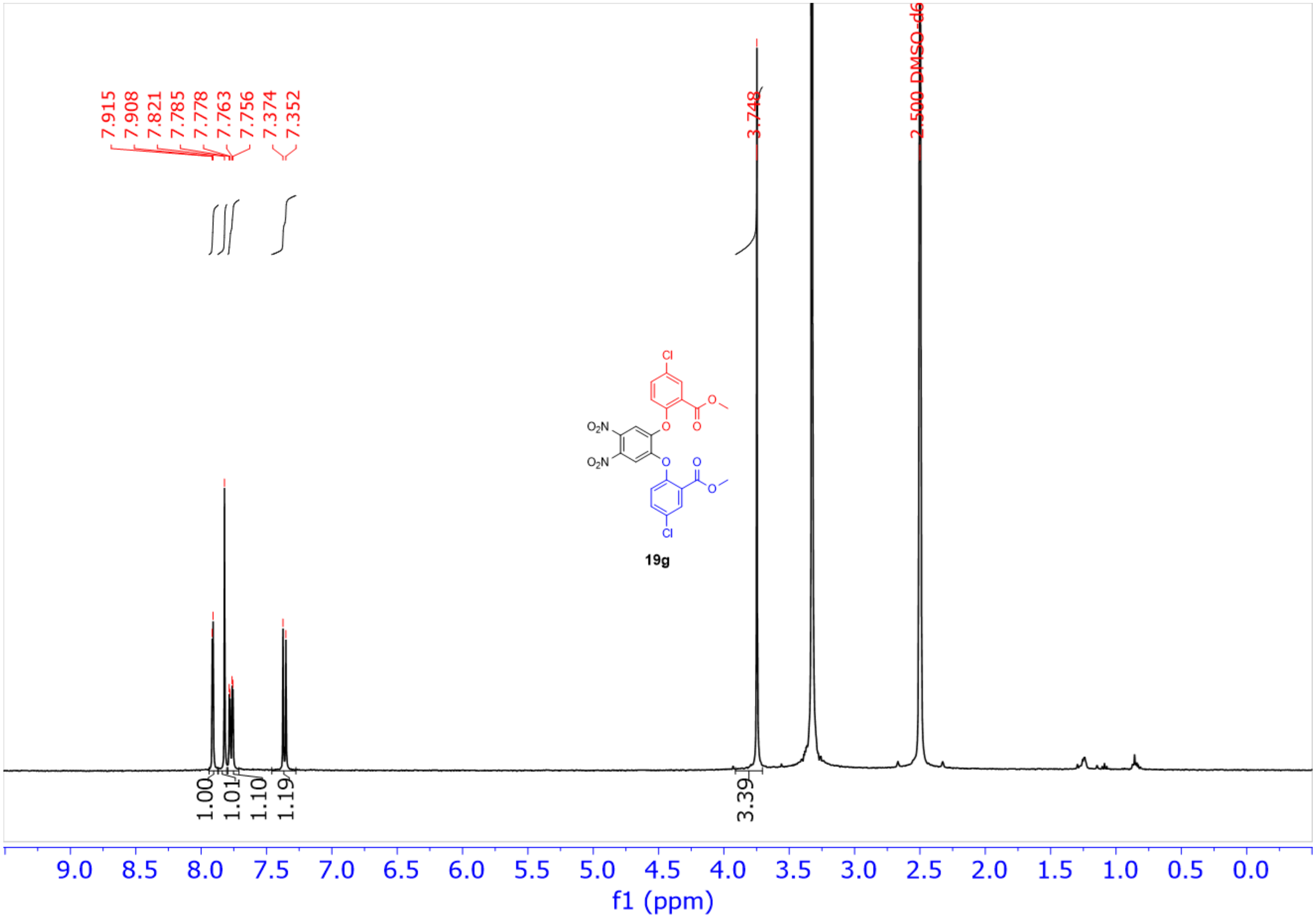

$^{13}C\{1H\}$ NMR (100 MHz, $(CD_3)_2SO$)

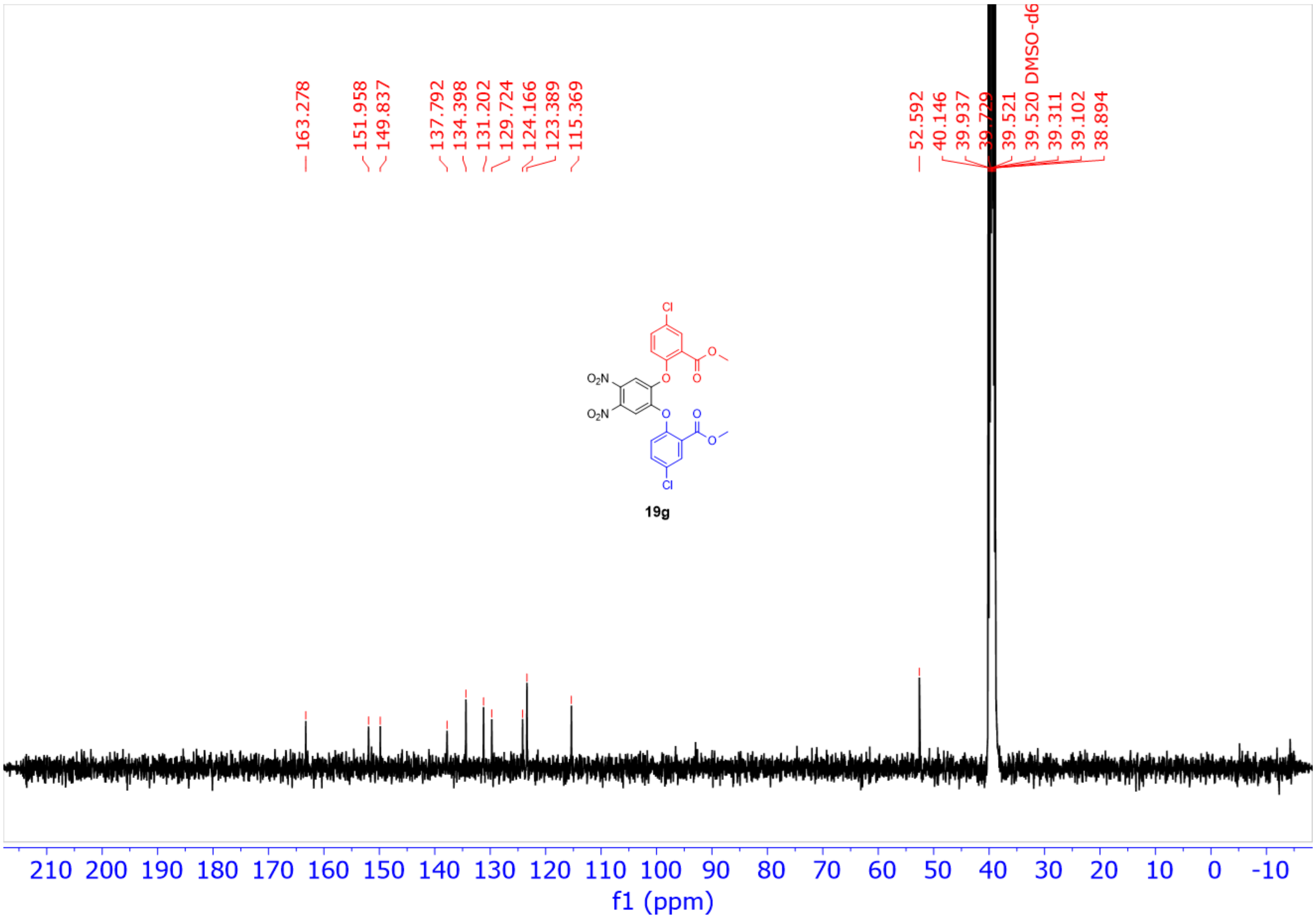

^{1}H NMR (400 MHz, $CDCl_3$)

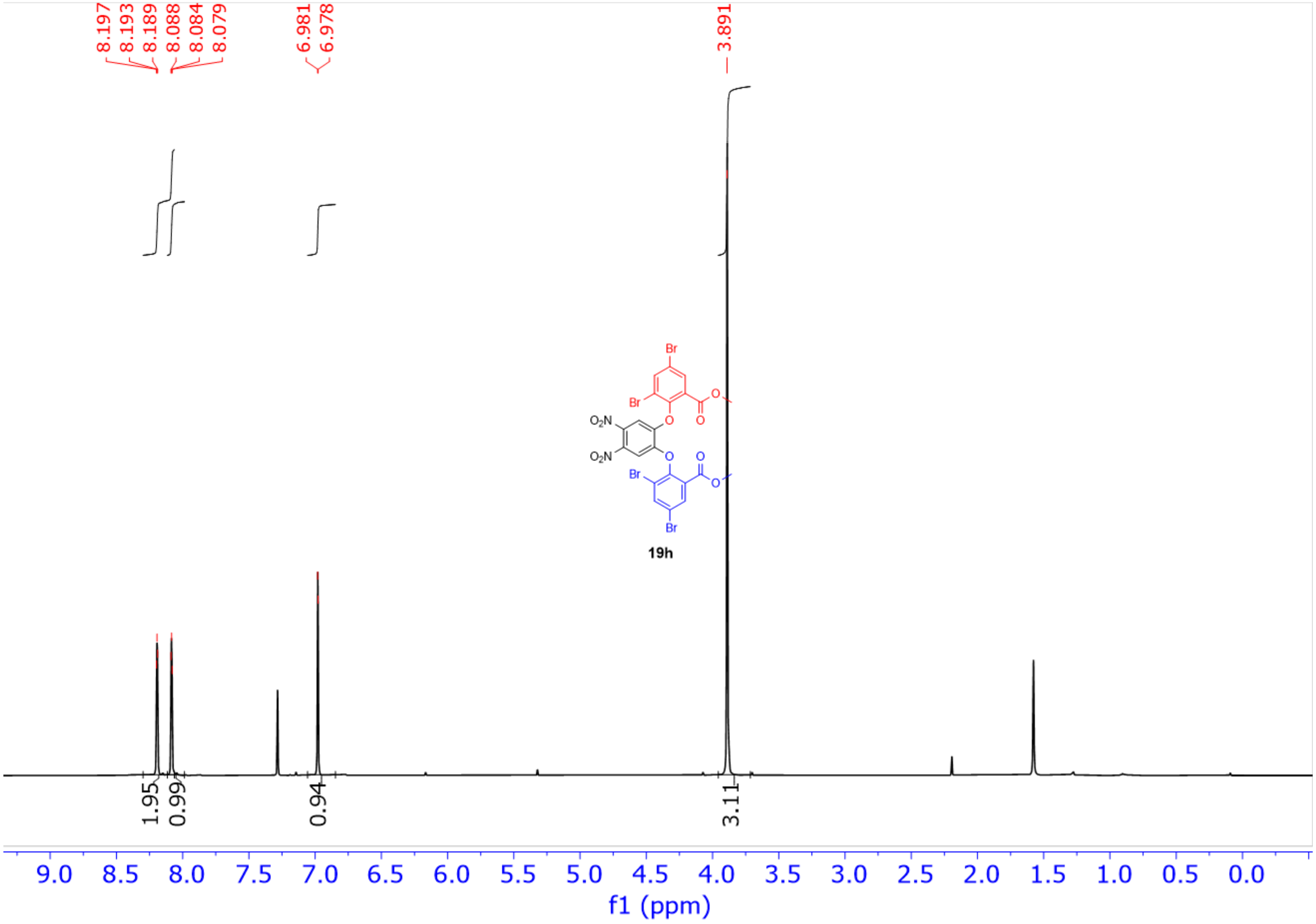

19h

$^{13}C\{1H\}$ NMR (100 MHz, $CDCl_3$)

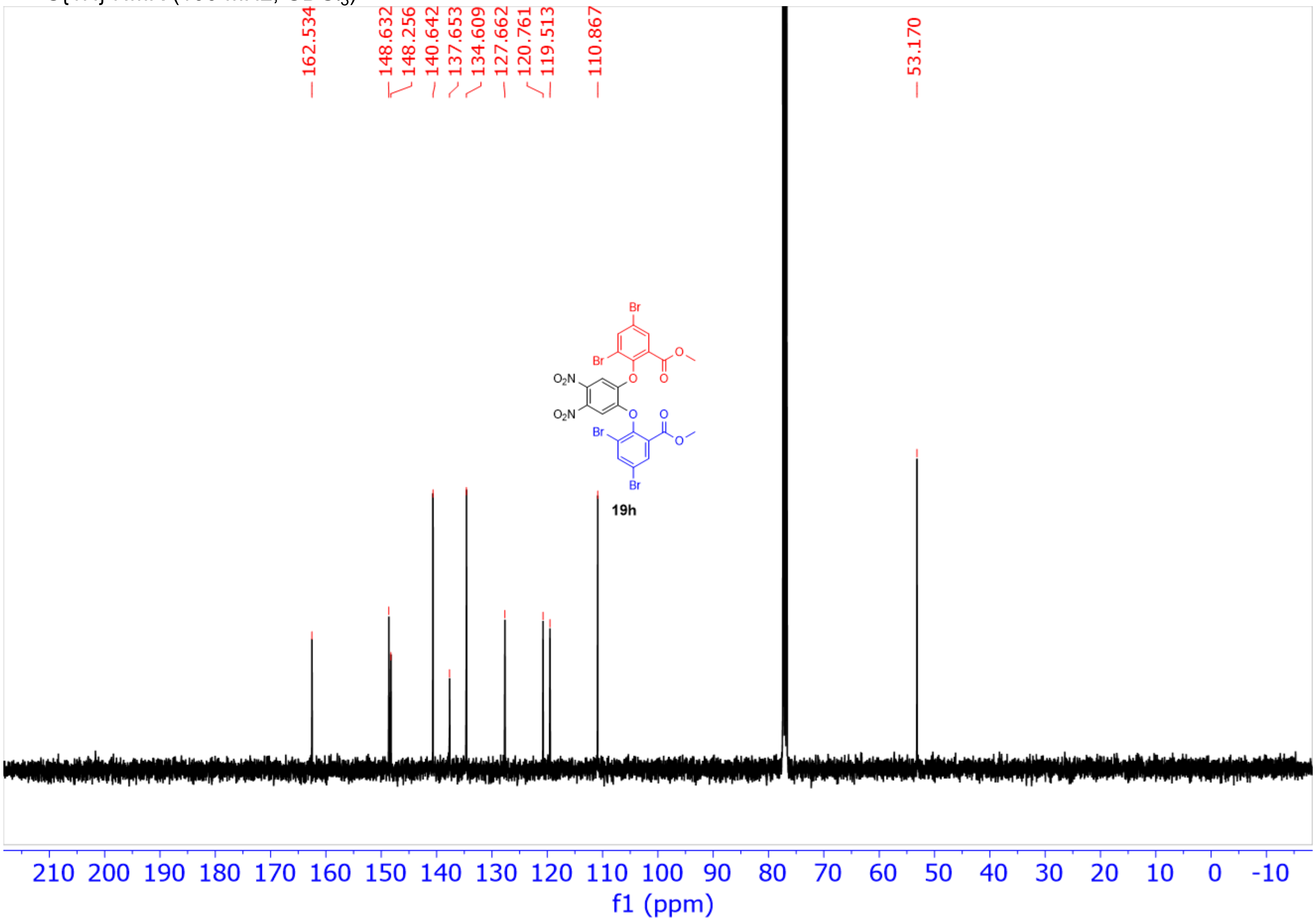

^{1}H NMR (400 MHz, $(CD_3)_2CO$)

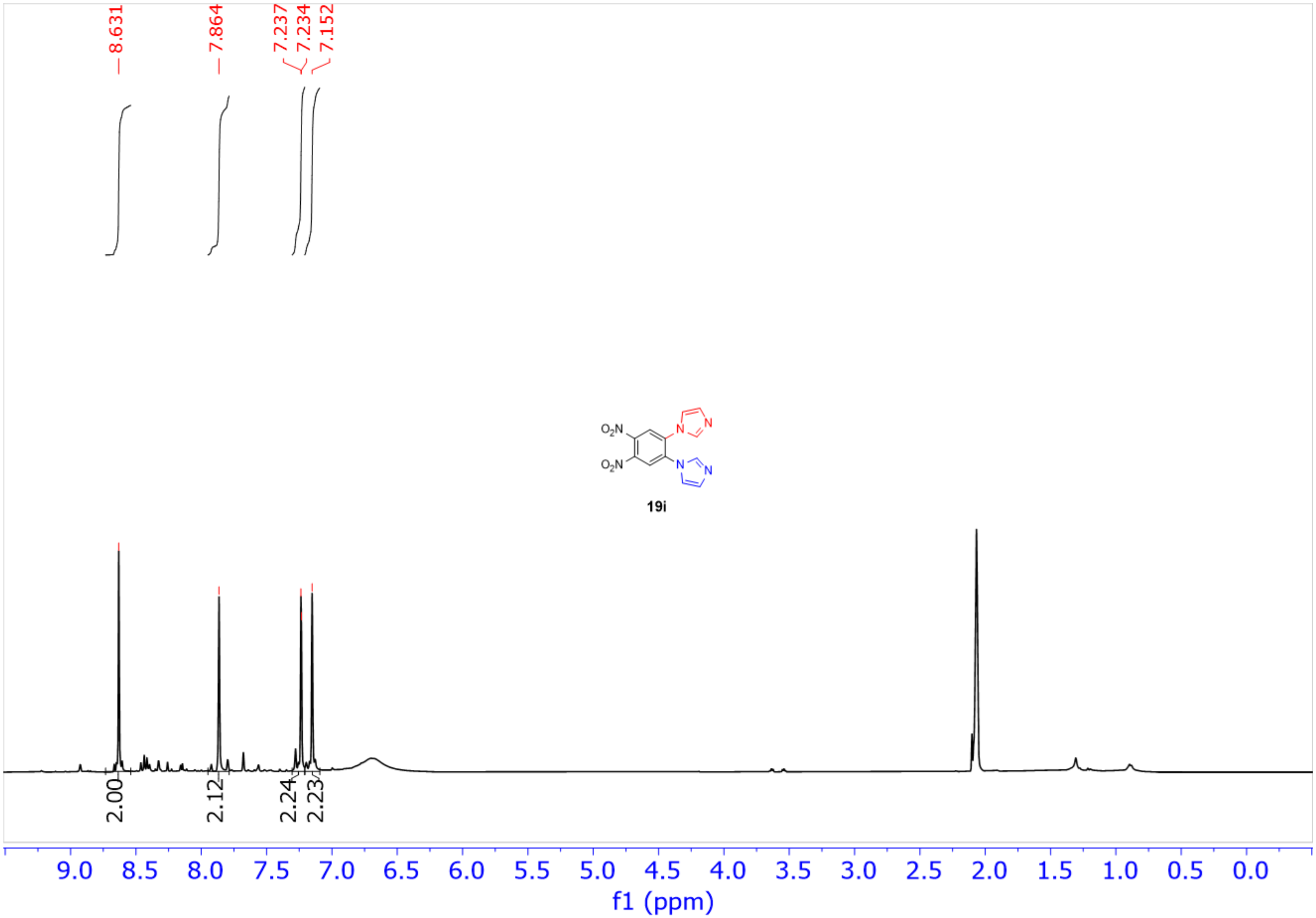

$^{13}C\{1H\}$ NMR (100 MHz, $(CD_3)_2CO$)

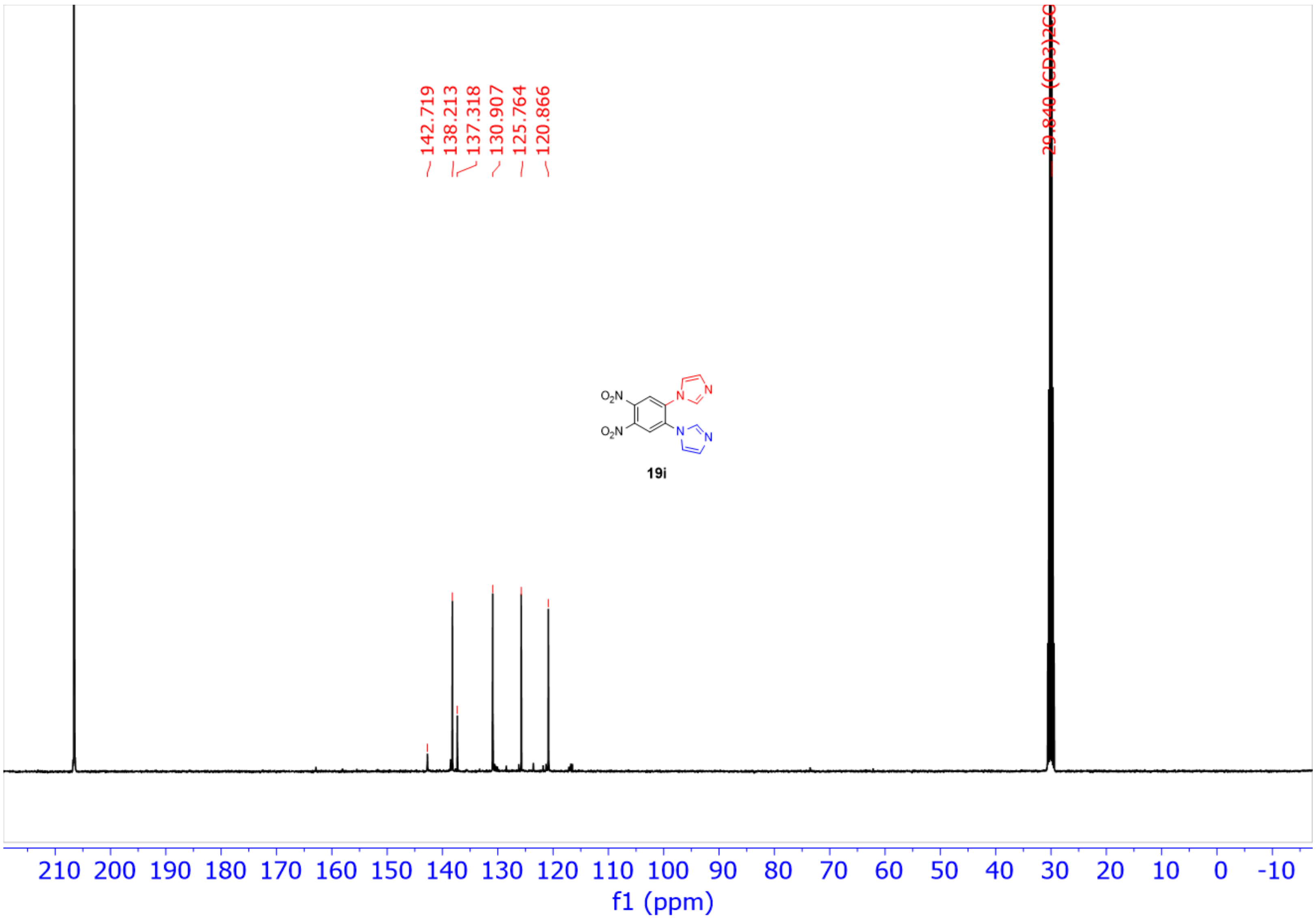

^{1}H NMR (400 MHz, $CDCl_3$)

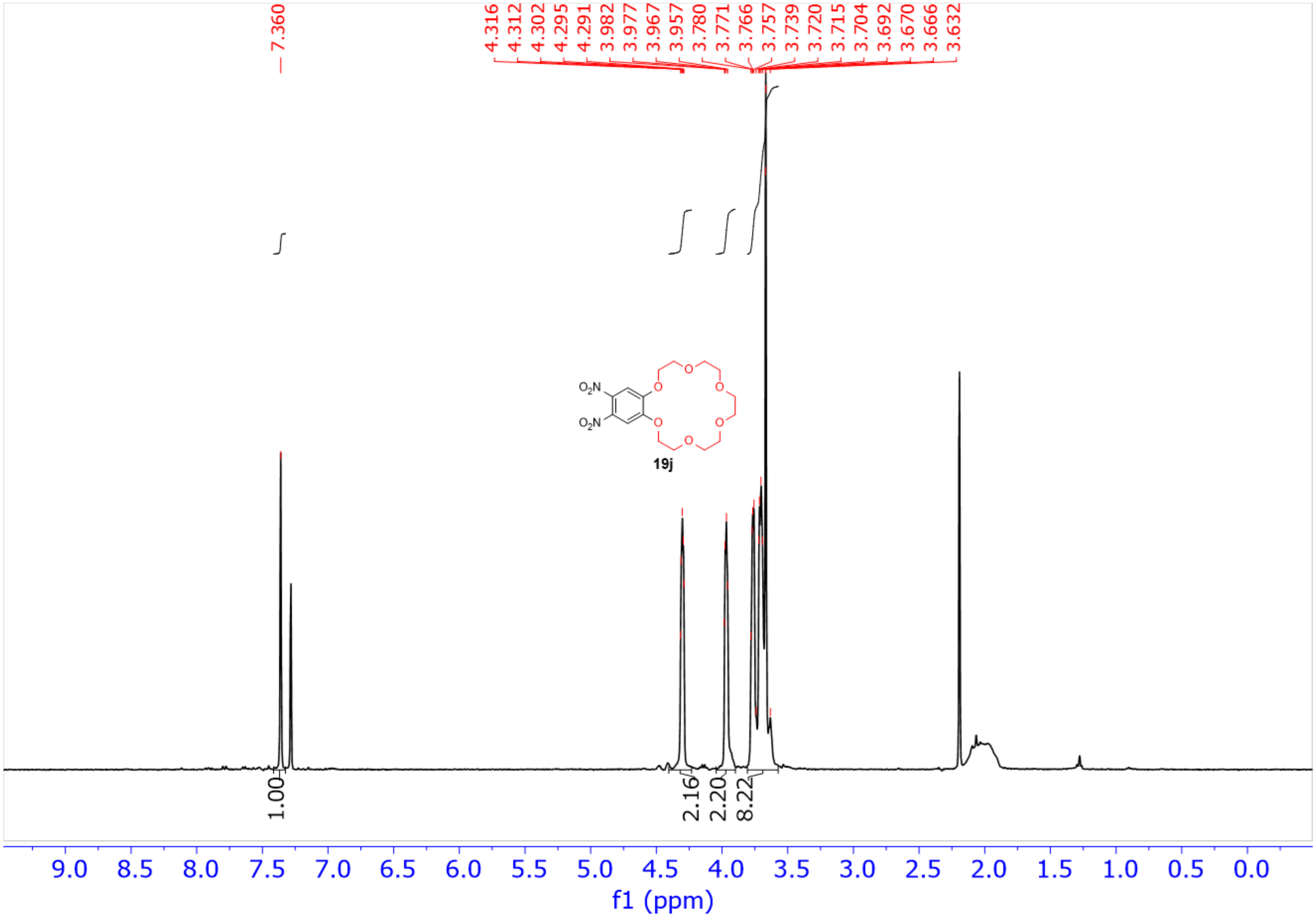

^1H NMR (400 MHz, $CDCl_3$)

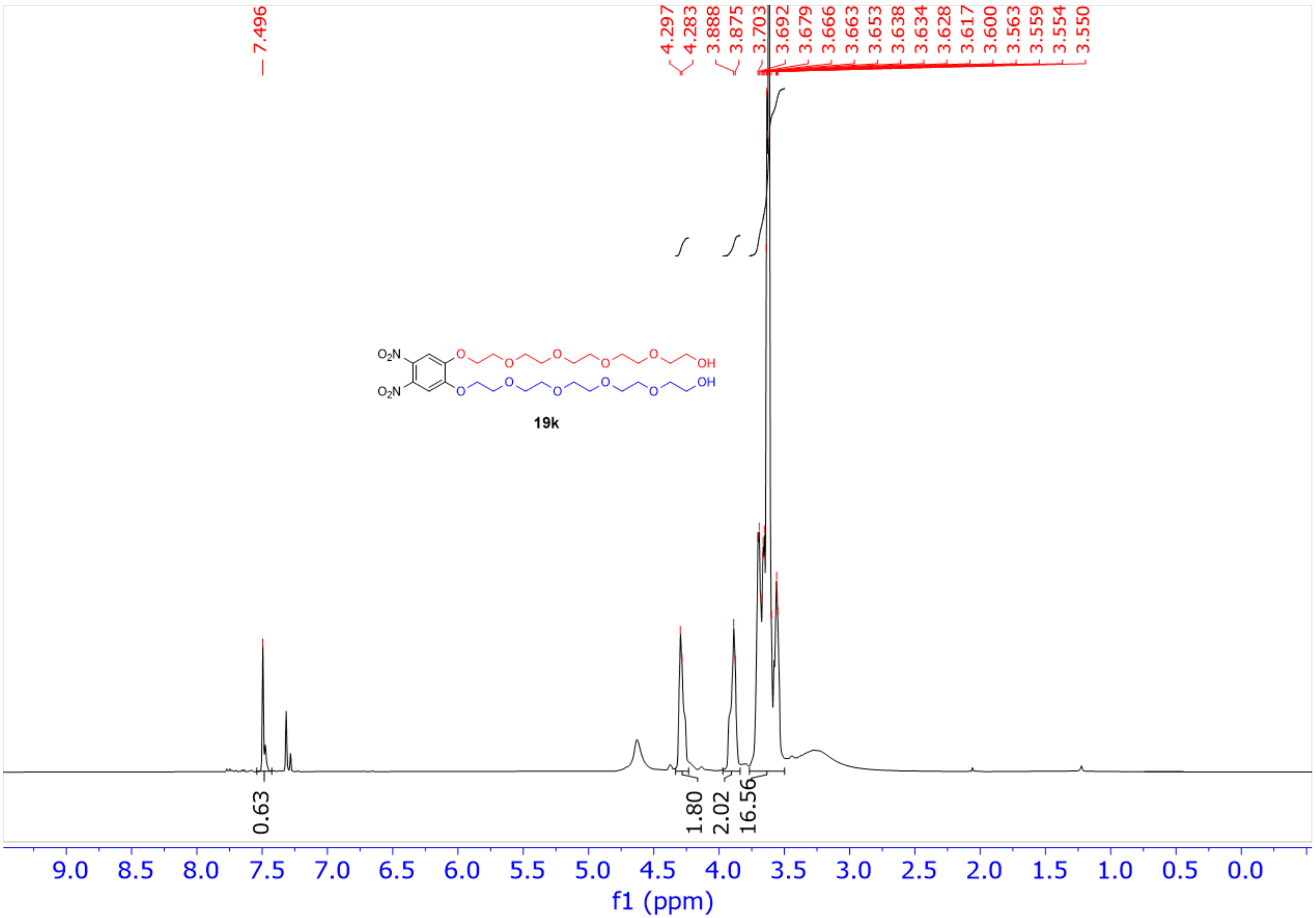

$^{13}C\{1H\}$ NMR (100 MHz, $CDCl_3$)

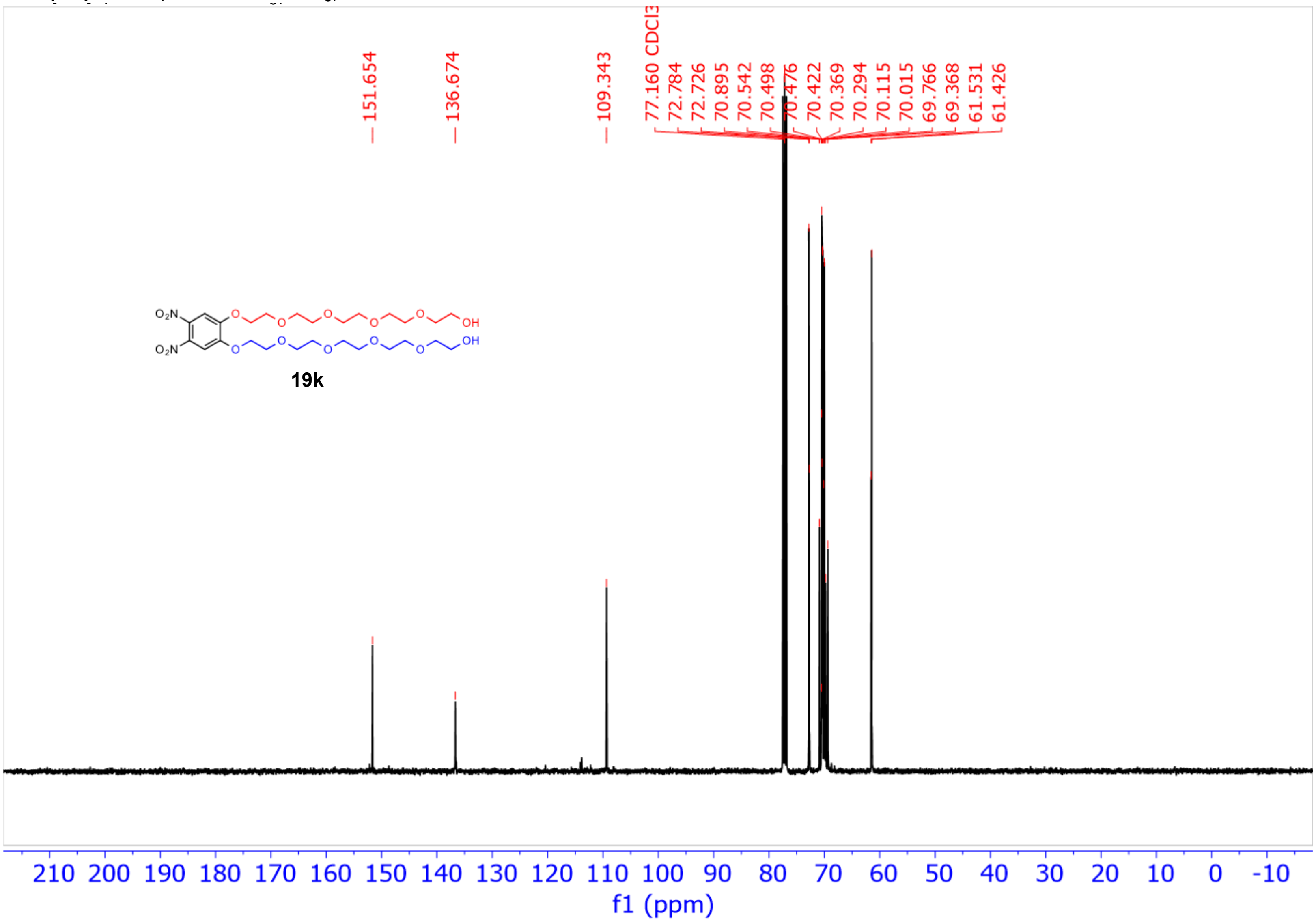

Supplement: Supplementary file 1 [file jo5c00538_si_001.pdf]
